# A revision of the *minor* species group in the millipede genus *Nannaria* Chamberlin, 1918 (Diplopoda, Polydesmida, Xystodesmidae)

**DOI:** 10.3897/zookeys.1030.62544

**Published:** 2021-04-13

**Authors:** Jackson C. Means, Derek A. Hennen, Paul E. Marek

**Affiliations:** 1 Virginia Tech, Department of Entomology, Blacksburg, Virginia 24061, USA Virginia Tech Blacksburg United States of America

**Keywords:** Appalachia, gonopod, micro-range endemic, short-range endemic, spatulate, twisted-claw

## Abstract

Millipedes in the family Xystodesmidae (Polydesmida) are often referred to as “colorful, flat-backed millipedes” for their bright aposematic coloration and tendency to form Müllerian mimicry rings in the Appalachian region. However, there are many species of Xystodesmidae that do not display colorful warning patterns, and instead have more cryptic appearances. Perhaps for this reason, groups such as the genus *Nannaria* have remained understudied, despite containing a large number of undescribed species. Before his death in 2012, R. L. Hoffman worked on a revision of the genus *Nannaria*, and synthesized material and drawings since 1949. Here the work is continued, inferring a molecular phylogeny of the Nannariini (*Nannaria* + *Oenomaeapulchella*), and revealing two clades within the genus. One clade is named the *minor* species group, and the second is the *wilsoni* species group. This revision, using a molecular phylogenetic framework, is the basis for descriptions of 35 new species in the *minor* species group. A multi-gene molecular phylogeny is used to make taxonomic changes in the taxon. Eleven putative species of *Nannaria* are also illustrated and discussed. Additionally, detailed collection, natural history and habitat notes, distribution maps, and a key to species of the *Nannariaminor* species group are provided. These items are synthesized as a basis for a revision of the genus, which hopefully will aid conservation and evolutionary investigations of this cryptic and understudied group.

## Introduction

**Figure 1.**
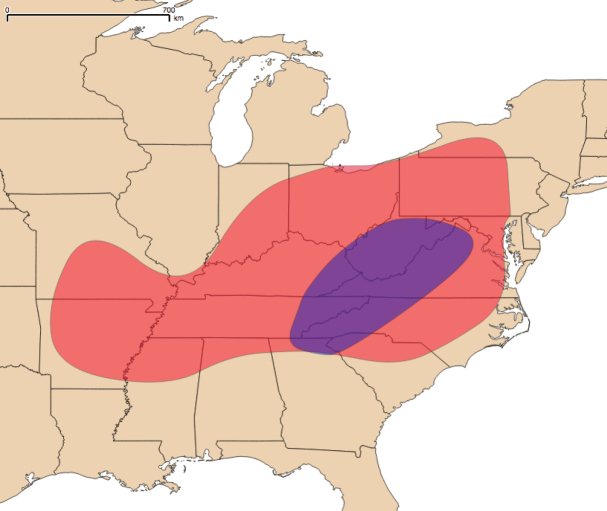
The geographic distribution of *Nannaria* Chamberlin, 1918. Red: *minor* species group; Purple: *wilsoni* species group.

The genus *Nannaria* Chamberlin, 1918 (Polydesmida, Xystodesmidae) inhabits the eastern United States, from western Arkansas and Missouri to just outside of Rochester, New York in the north, to the coast of Virginia and to central Mississippi in the south (Fig. 1). Despite this extensive range, the group as a whole is poorly known, and only two studies in the last 40 years have specifically addressed taxonomic changes to *Nannaria* (Hennen and Shelley 2015; Shelley and Smith 2018). Furthermore, genetic techniques are still rarely applied in modern diplopod systematics, and *Nannaria* has been included only twice in a molecular phylogeny (Marek and Bond 2006; Means et al. 2021). While these works shed light on the placement of *Nannaria* within the Xystodesmidae, their analyses relied on limited taxon sampling of the tribe. While the affinities of the Nannariini [*Nannaria* + *Oenomaeapulchella* (Bollman, 1889)] to the other members of the family have been shown, their taxon sampling scheme omitted most nannariine species. Here, we use six genes (16S, 28S, EF1a, rpb1, COI, and fbox) to construct a molecular phylogeny of the Nannariini, and include 139 *Nannaria* specimens, *O.pulchella* and 12 taxa in the Rhysodesmini, to resolve the phylogenetic relationships between the genera of Nannariini. Additionally, we describe 35 new species, and demonstrate that *Nannaria* contains two clades, one of which, the *wilsoni* species group, is to be revised in a subsequent publication.

### History of the Nannariini and diagnostic characters

**Figure 2.**
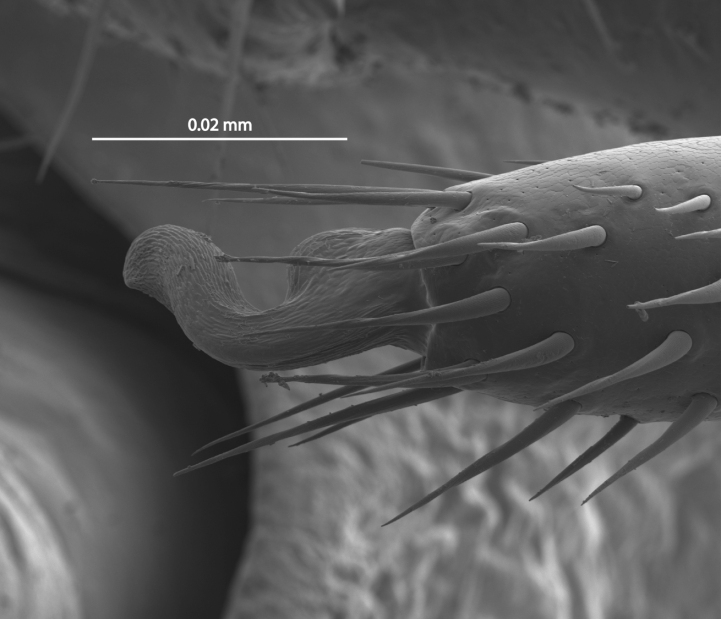
Scanning electron microscope image of a spatulate, twisted pregonopodal claw of a male *Nannariaericacea* Hoffman, 1949.

The tribe Nannariini has received little attention since being proposed in 1964 by R. L. Hoffman, and as such does not have the same nomenclatural peregrinations of some animal taxa (Hoffman 1964). The species within the Nannariini have been placed in five different genera over the years (*Mimuloria* Chamberlin, 1928, *Fontaria* Gray, 1832, *Castanaria* Causey, 1950, *Nannaria*, and *Polydesmus* Latreille, 1802), with the most recent change by Means et al. (2021) synonymizing *Mimuloria* with *Nannaria* for the current count of two genera within the Nannariini. The oldest of these two, *Nannaria*, was first proposed by Chamberlin (1918), and he included within it the previously described *Fontariatennesseensis* Bollman, 1888, as well as three new species, *Nannariamedia* Chamberlin, 1918, *N.infesta* Chamberlin, 1918 and *N.minor* Chamberlin, 1918. Two of these species, *N.infesta* and *N.media*, have recently been shown to belong to separate genera (Means et al. 2021), now *Howellariainfesta* (Chamberlin, 1918) and *Borariastricta* (Brölemann, 1896), respectively. Chamberlin’s (1918) description of *Nannaria* was brief, and while he stated that he would define the genus in greater detail later, Chamberlin did not revisit the task. Additionally, neither Chamberlin (1918) nor Bollman (1888) provided illustrations of the gonopods, the external male reproductive structures traditionally used for specific and generic differentiation in millipede taxonomy, for the above four species; therefore *Nannaria* had remained a poorly defined genus for 31 years until Hoffman (1949) finally provided a brief diagnosis of the genus. Williams and Hefner (1928) later stated that “it is not deemed necessary to make these more technical divisions” in reference to Chamberlin’s split of *Fontaria* into three genera, *Mimuloria*, *Nannaria*, and *Apheloria* Chamberlin, 1921 (Chamberlin 1918, 1921, 1928); however, they themselves did not propose an alternative arrangement. Nor did they formalize a synonymy. Hoffman’s (1949) diagnosis merely mentioned that *Nannaria* have laterally positioned repugnatorial pores, gonopods with paired processes, and are “olive to black with keels [paranota, or lateral extensions of the body ring] pink”. [Note that color may confound diagnosing species in the Xystodesmidae (Marek and Bond 2006), and it is now known that species with distinctly different color patterns may be the same species or population of millipede.] Perhaps due to their presence in *Oenomaeapulchella* and absence in *H.infesta* and *B.stricta*, Hoffman (1964) did not list the distinct spatulate pregonopodal claws of *Nannaria* as a diagnostic feature of the tribe (see Fig. 2). Hoffman (1964) also omitted from his diagnosis the subcoxal sternal spines that are characteristic of the tribe, though again this may be due to the lack of sternal modifications in *H.infesta* and *B.stricta*.

**Figure 3.**
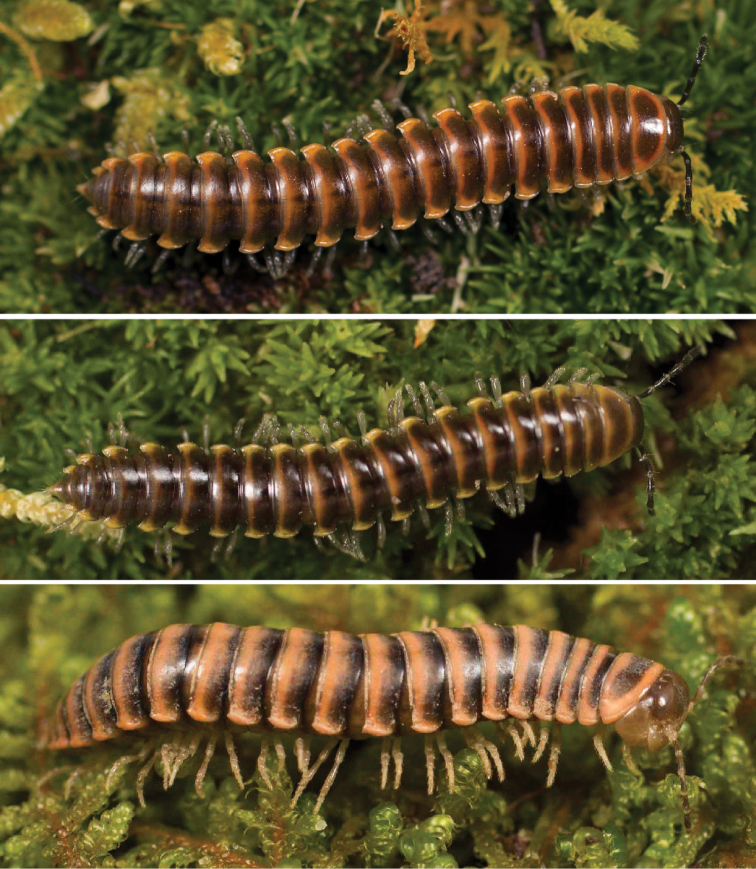
Examples of the coloration of *Oenomaeapulchella* Hoffman, 1964.

Fifteen years later, spurred by the discovery of an unusual male xystodesmid from Georgia which conformed somatically to the female type specimens of *Fontariapulchella* Bollman, 1889 collected in Tennessee, Hoffman (1964) proposed the tribe Nannariini to include *Nannaria* and the new genus *Oenomaea* Hoffman, 1964 (Fig. 3). Hoffman (1964) also synonymized the genus *Mimuloria* under *Nannaria*, a change which would later be reversed by Hennen and Shelley (2015), and again synonymized by Means et al. (2021). Hoffman (1964) provided a diagnosis of the tribe, which included characters such as the spatulate pregonopodal claws, the subcoxal sternal spines and the conical coxal projections on the fourth leg pair. The three characters which he provided to differentiate the genera *Oenomaea* and *Nannaria* are the strongly twisted pregonopodal claws of *Nannaria* and the acutely projecting paranotal corners and the flattened, plate-like solenomerite (tip of the acropodite) of *Oenomaea* (Hoffman 1964).

Nearly a half-century later, Hennen and Shelley (2015) revived *Mimuloria* as a separate genus, synonymizing *Nannariadepalmai* (Causey 1950a) under *Mimuloriacastanea* (McNeill, 1887) and describing two new species including one subspecies. Hennen and Shelley (2015) suggested that the *Nannaria* species with complex gonopods be placed within *Oenomaea* or in a new genus, due to their gonopods having solenomeres, the structure which carries the seminal groove after a bifurcation near the tip of the acropodite; however, they did not formally make this change. The changes that Hennen and Shelley’s (2015) publication made to the Nannariini were based entirely on morphological characters, primarily the terminal acropodite ornamentation found in some *Nannaria* (see Figs 22, 36, and 37). This was the major character that both *Mimuloria* and *Castanaria* were based upon when they were described by Chamberlin (1928) and Causey (1950a), and Hoffman (1964: 33) stated that “I find this character to be a mutable one, and more suitable for the distinction of a species-group than a separate genus.” *Mimuloria* was thereafter synonymized under *Nannaria* by Means et al. (2021), after their six-gene (16S, 12S, tRNAval, COI, EF1a, and 28S) molecular phylogeny found that species of *Mimuloria* were in multiple locations within *Nannaria*.

Shelley and Smith (2018) moved the family Eurymerodesmidae into the Xystodesmidae, based on a hypothesized sister group relationship with the Nannariini. Due to the priority of the Eurymerodesmini, the Nannariini were reduced to a subtribe, the Nannariina. This change was made in the absence of molecular evidence, and based solely on the presence of stout setae along the inner margin of the gonopods. Based on molecular phylogenetics, Means et al. (2021) found that the Eurymerodesmini are members of the Xystodesmidae, and with the tribe Euryurini, are sister to the Nannariini; the tribe Nannariini was therefore revived as a separate tribe distinct from the Eurymerodesmini and Euryurini.

**Figure 4.**
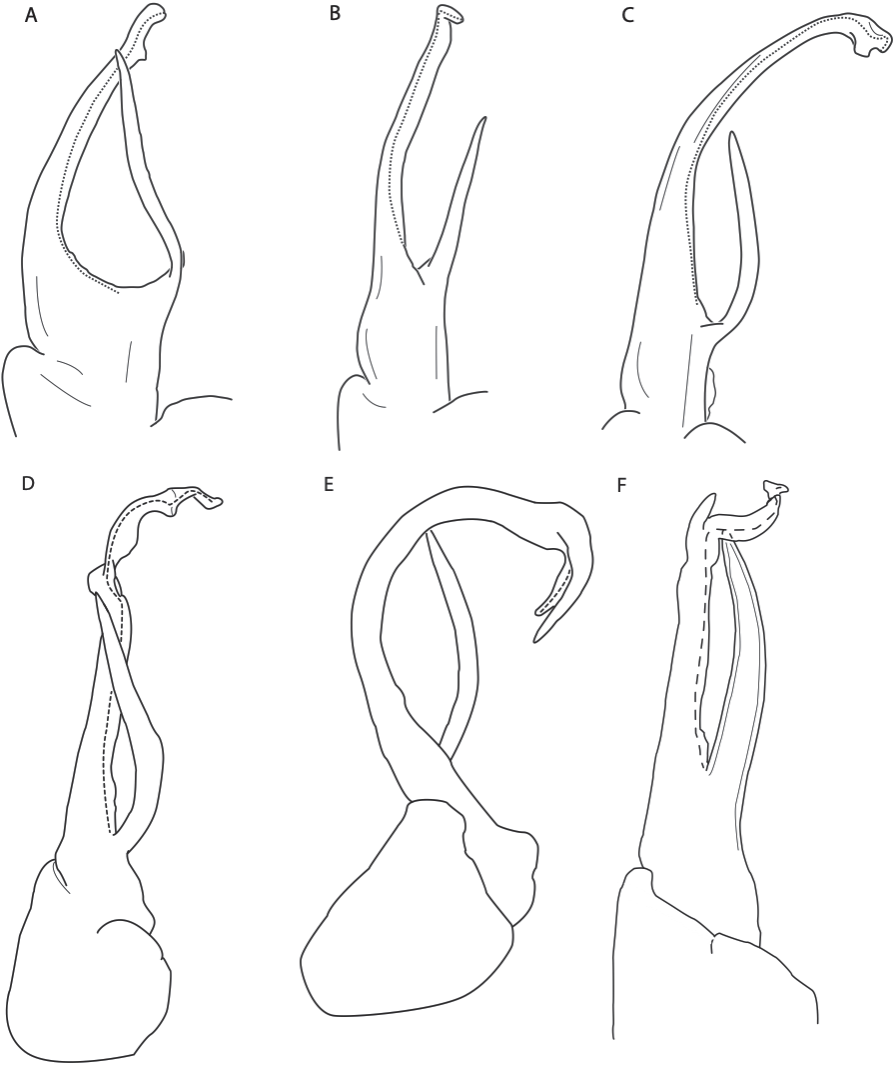
Examples of the *minor* species group (**A–C**) and *wilsoni* species group (**D–F**) gonopods, anterior view, setae removed for clarity **A***Nannariaminor* Chamberlin, 1918 **B***N.terricola* Williams & Hefner, 1928 **C***N.scholastica* sp. nov. **D***N.austricola* Hoffman, 1950 **E***N.shenandoa* Hoffman, 1949 **F***N.ericacea* Hoffman, 1949.

A considerable breadth of gonopodal variation exists amongst populations of *Nannaria* and has been a great challenge to the solely morphology-based taxonomy of the group. Hoffman (1964, 1999) and Hennen and Shelley (2015) have remarked that there exists a wide variety of gonopodal forms in *Nannaria*, which either indicates a highly variable massive genus or multiple genera. The most obvious divide is between those with simple, stick-like gonopods (Fig. 4A–C, the *minor* species group after the generotype *N.minor*) and those with complex, twisting, and often adorned gonopods (Fig. 4D–F, the *wilsoni* species group after *N.wilsoni* Hoffman, 1949, a species found around Blacksburg, Virginia). While Hoffman (1964) commented on the variety of gonopodal forms in the *Nannaria* on multiple occasions, even going so far as to suggest that there may exist up to 200 species within the genus, he never pointed out this divide between simple and complex gonopods in his publications or his personal notes. Here we present a six-gene molecular phylogeny of the Nannariini which reveals multiple clades within the *minor* species group, and 35 new species.

## Materials and methods

### Specimen collection and preservation

Collection sites were selected based on a combination of factors, including: availability of suitable habitat, location of type localities, information from R. L. Hoffman’s notes on *Nannaria*, and natural history collections from the Virginia Museum of Natural History and the North Carolina State Museum—repositories with large holdings of nannariine specimens. Collection methods followed that of Means et al. (2015). Briefly, adult and juvenile *Nannaria* were collected by hand with the use of a millipede rake or gardening claw, often by removing leaf litter and digging 2–3 cm beneath the soil. For the 12 species of Rhysodesmini used in this study, collection methods were similar, though adults were often found under leaf litter, above the soil.

Adult and juvenile millipedes were brought back to the lab for identification, photography, DNA and RNA collection, preservation, and, in the case of juveniles, housed in terraria until maturation. Live millipedes were photographed with a Canon EOS 6D digital camera with a 50 mm macro lens to record color and habitus morphology. Legs from rings 8–18 were removed using forceps from the left side of adults and placed in either RNA*later* (Qiagen, Hilden, Germany) for the preservation of DNA and RNA, or 100% EtOH for the preservation of DNA. Preserved legs were stored at -80 °C for archival storage. Specimens sans legs were given a unique specimen code, stored in 70% isopropanol and deposited in the Virginia Tech Insect Collection (**VTEC**; https://collection.ento.vt.edu). Type material deposition location is noted in each species description under Materials Examined, and includes the VTEC, the Field Museum of Natural History (**FMNH**), and the Virginia Museum of Natural History (**VMNH**). Institutions which provided specimen loans are as follows: Florida State Collection of Arthropods (**FSCA**), North Carolina Museum of Natural Sciences (**NCSM**), Museum fur Naturkunde, Berlin (**MFN**), Smithsonian National Museum of Natural History (**NMNH**), and the VMNH. Institution abbreviations mentioned in the text include The Academy of Natural Sciences of Philadelphia (**ANSP**) and the United States National Museum (USNM; now NMNH, see above). The abbreviation “leg.” (*legit* = ‘has collected’) follows the name(s) of the collector(s) of the specimen.

### Primer design

The transcriptomes of *Nannariahokie* Means, Hennen & Marek, 2021 (*minor* species group) and *Nannariaericacea* (*wilsoni* species group) sequenced by Means et al. (2021) were used for gene exploration and primer development as described by Means et al. (2021). Twenty-seven gene region candidates (Suppl. material 1) were blasted (Altschul et al. 1990) against both transcriptomes and eleven were chosen for primer design and testing based on the following criteria: presence in both *Nannaria* transcriptomes, gene region length > 400 base pairs, and > 3% gene region variation between transcriptomes.

### DNA extraction and phylogenetics

DNA was extracted and purified using a Qiagen DNeasy tissue kit and stored at -20 °C. For *Nannaria*, we used 4–6 legs per specimen and eluted in 50 ul of Qiagen DNeasy Buffer AE due to their small size (average 25 mm in length); while for larger bodied Xystodesmidae, such as the outgroup *Pachydesmus* Cook, 1895, we used three legs and 100 µl of Buffer AE. For legs stored in 100% EtOH, these were air-dried at room temperature (22 °C) prior to DNA extraction with the same protocol above. Six gene fragments were amplified for each specimen: cytochrome c oxidase subunit I gene (COI), elongation factor-1 alpha gene (EF1a), 28S ribosomal RNA gene (28S), large ribosomal RNA gene (16S), F-box protein (fbox), and DNA-directed RNA polymerase II subunit RPB1 (RPB1). Amplifications were cleaned, quantified, and then sequenced using an Applied Biosystems 3730 DNA Analyzer.

We used the programs phred and phrap within the Mesquite (Version 3.5) module Chromaseq (Version 1.31) for base-calling, generation of sequence contigs and sequence trimming (Maddison and Maddison 2010; Ewing et al. 1998). Individual genes were aligned with five iterations each in PRANK (Version 140603) and with the default HKY model with empirical base frequencies, kappa=2, the -F option and iterative guide trees (Löytynoja and Goldman 2005). Aligned sequences were then concatenated in Mesquite, partitioned by gene, codon site (for protein-coding genes), and intron/exon boundaries (for EF1a and RPB1). The concatenated matrix was then exported to PartitionFinder 2 (Version 2.1.1) as .phy and .cfg files for determination of best-fit models of nucleotide site substitution for each partition (Table 1). Partition groupings were determined using the ‘greedy’ scheme and a Bayesian Information Criterion (BIC) model selection method (Lanfear et al. 2012). For the phylogenetic analysis, the concatenated matrix was exported from Mesquite for MrBayes (Ronquist et al. 2005), and the best-fit partition block from PartionFinder 2 was included in the nexus file (Lanfear et al. 2012). In MrBayes (Version 3.2.6), we ran two simultaneous pairs of hot and cold MCMC chains, with a 25% burn-in, for 210 million generations. Sampling occurred every 100 generations until the standard deviation of split frequencies (SDSF) reached < 0.01, indicating that the chains had converged on a set of consensus topologies (Ronquist et al. 2005). The MrBayes commands “sumt” with “contype=allcompat” were used to summarize the posterior distribution of trees into a consensus tree while displaying frequencies of all bipartitions. Individual gene trees were estimated in MrBayes to assess separate gene histories. Nucleotide base composition and frequency, as well as sequence heterogeneity, were assessed in IQ-TREE 2 (Version 2.0.4, Minh et al. 2020), excluding non-Nannariini taxa. Sequence heterogeneity was measured using χ^2^ tests of stationarity, with the null hypothesis of homogeneity (Table 2).

### Morphological character scoring, illustrations, and distribution mapping

For each species, gonopods were dissected under a Leica M125 stereomicroscope (Leica Microsystems, Wetzlar, Germany), and photographed using a Canon 6D camera with a 65 mm MP-E macro lens mounted on a Passport II Portable Digital Imaging System (Visionary Digital, Charlottesville, VA). Gonopods were photographed at three angles (anterior, posterior, and medial) and photographs were taken at 4× zoom, every 0.08 mm, for an average of 8–15 images per angle. These images were then focus stacked to create a composite photograph using the program Helicon Focus (Helicon, Kharkiv, Ukraine). Gonopod photographs were then traced in Adobe Illustrator CC 2018–2021 using the pen tool (Adobe, San Jose, CA). Specimens were then scored for gonopodal, cyphopodal and somatic characters in a 48-character matrix adapted from Marek and Bond (2006) for *Nannaria* (Suppl. material 6). All measurements were recorded in millimeters and the following six areas were measured according to Marek (2010): body length (BL), collum width (CW), intergenal width (IW), interantennal socket width (ISW), body ring 11 width (B11W), and body ring 11 height (B11H). Latitude and longitude coordinates were obtained for literature records by georeferencing collection data in Google Earth (Google LLC, Mountain View, CA). Distribution maps were generated using SimpleMappr (Shorthouse 2010) and edited in Adobe Illustrator CC 2018–2021. Distributions represented by a single collection locality are denoted as “N/A” below.

### Species delimitation

For the delimitation of species, we used a combination of morphological and molecular criteria as implemented in Marek (2010) and Means et al. (2021). Briefly, a representative specimen from a sampled population needed to display overt morphological dissimilarity from previously known species, and/or be placed in the concatenated molecular phylogeny in an un-hypothesized location, to be considered an undescribed species. There exists a paucity of somatic features which vary between *Nannaria* species and populations, however gonopodal variation between species can be pronounced. Differences in gonopod morphology have dictated the establishment of a majority of species in the Diplopoda, and we are confident that a combination of gonopodal variation and molecular evidence is reliable criteria for species delimitation. We accepted paraphyletic species where multiple populations of a single species were paraphyletic with respect to another species and possessed overt gonopodal differences. Specimens which displayed gonopod morphology that was sufficiently different from known species of *Nannaria*, but for which we did not have genetic material, were designated as *incertae sedis*. While we include illustrations of *incertae sedis* specimens to aid in future taxonomic work on *Nannaria*, we refrain from elevating these indeterminate specimens to species. For this revision we use the higher classification for the Xystodesmidae and *Nannaria* as outlined in Means et al. (2021).

**Figure 5.**
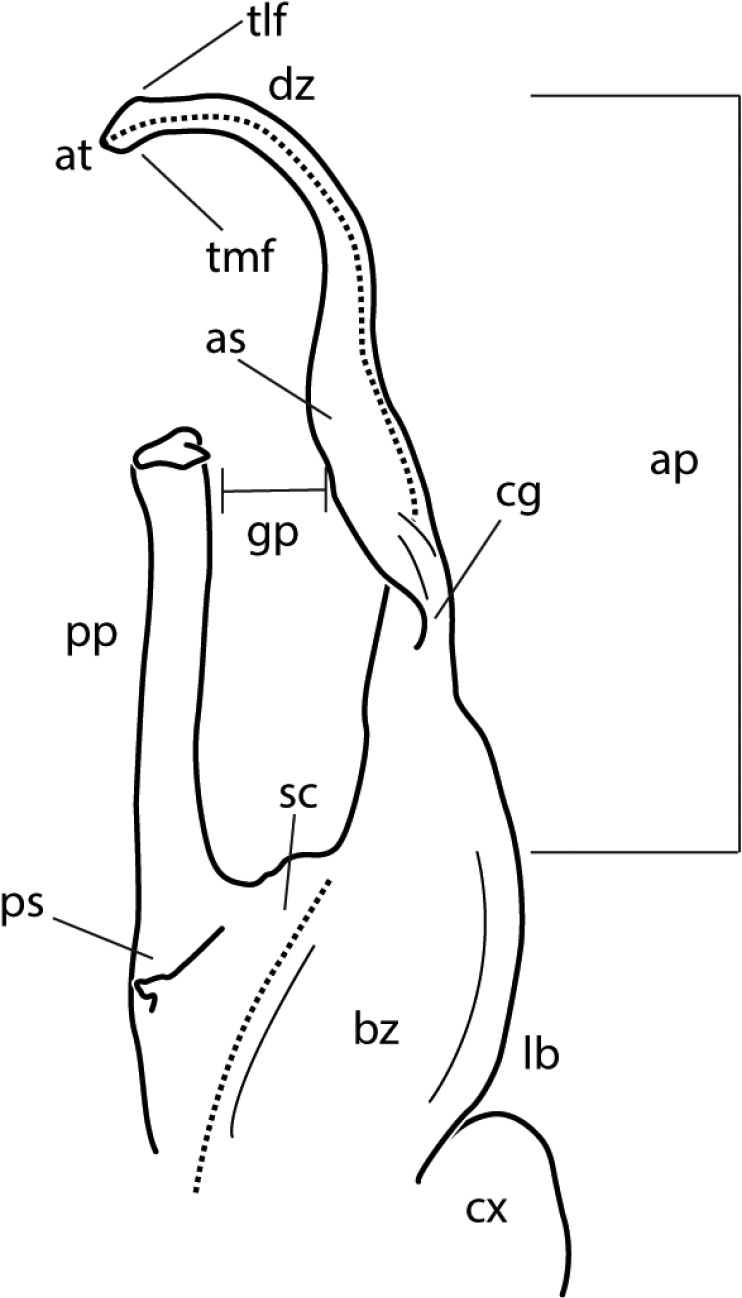
*Nannariacingulata* sp. nov. telopodite, posterior view. Abbreviations: ap = acropodite; as = acropodite swelling; at = acropodite tip; bz = basal zone; cg = cingulum; cx = coxa; dz = distal zone; gp = gap between pp & ac; lb = basal zone lateral bulge; pp = prefemoral process; ps = prefemoral spine; sc = seminal canal; tlf = tip lateral flange; tmf = tip medial flange.

### Morphological terminology

Morphological terms used to describe gonopods in *Nannaria* are as follows:

1. The coxa, defined as the basal segment of the gonopod [gonopod = a leg which has been modified into a structure for sperm transfer (Fig. 5, cx)].
2. The gonopodal telopodite, defined as the area distal to the coxa (Fig. 5).
3. The basal zone lateral bulge, defined as the lateral edge of the base of the gonopod which is occasionally pronounced in size (Fig. 5, lb)
4. The gonopod basal zone, defined as the area between the coxa and the acropodite (Fig. 5, bz).
5. The prefemoral spine, a new term, defined as the process arising at the base of the prefemoral process, often as a sharp spine, but occasionally reduced to a ridge, bulge, or completely absent (Fig. 5, ps).
6. The seminal canal, the linear trough that carries sperm during copulation, indicated in illustrations as a dashed line (Fig. 5, sc).
7. The prefemoral process, defined as the smaller of the two branches of the telopodite, and which does not carry the seminal canal (Fig. 5, pp).
8. The cingulum, defined as a fold or linear depression, found only in Nannaria cingulata sp. nov. (Fig. 5, cg).
9. The acropodite, which carries the prostatic groove (Fig. 5, ap), a distal subregion of the telopodite that does not include the prefemur.
10. The ‘gap’ between the prefemoral process and the acropodite (Fig. 5, gp).
11. The acropodite swelling, which is defined as present or absent, is a swelling of the region distal to the basal zone (Fig. 5, as).
12. The distal zone, defined as the area distal to the major inflection point of the gonopod (Fig. 5, dz).
13. The tip lateral flange, a flange which is occasionally present on the upper portion of the acropodite tip (Fig. 5, tlf).
14. The tip medial flange, a flange which is occasionally present on the underside of the acropodite tip (Fig. 5, tmf).
15. The acropodite tip, defined as the terminal portion of the acropodite (Fig. 5, at).

## Results

### Specimen collection

**Figure 6.**
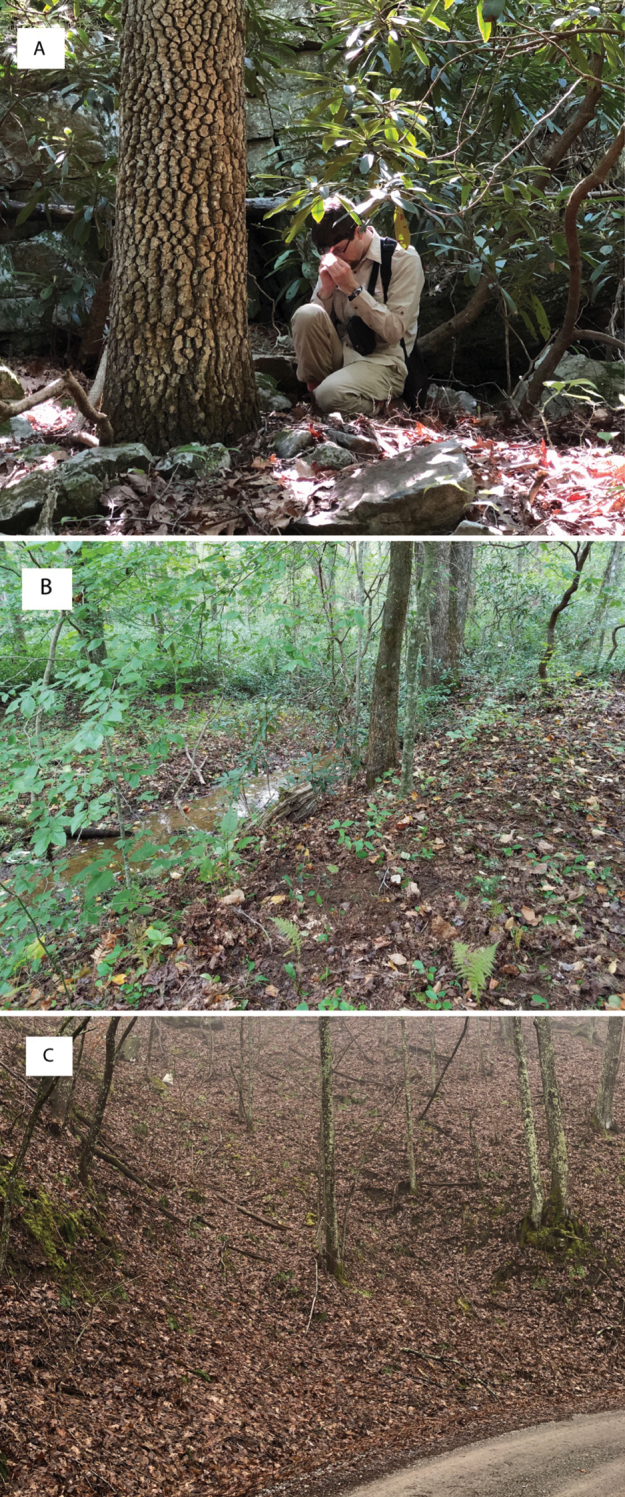
Examples of *Nannaria* Chamberlin, 1918 habitat **A** broadleaved forest and *Rhododendron* cove interface **B** riparian corridor **C** steep mesic hillside.

In total, 935 *Nannaria* specimens were collected between 2014 and 2018 throughout the eastern United States. Areas of suitable habitat included broad leaved forests and hemlock groves, typically near a body of water or riparian area, and *Rhododendron* coves (Fig. 6). Immature *Nannaria* appeared to display a preference for molting underneath *Rhododendron*, while adult *Nannaria* were readily found near the edges of *Rhododendron* groves, but rarely within the actual grove. Additionally, sloped hillsides near streams with dark, loamy soil and sparse root mats were often productive sites to encounter *Nannaria* specimens. A .kmz file of *Nannariaminor* species group distributions for use in programs such as Google Earth (Alphabet Inc., Mountain View, California) is provided as Suppl. material 8.

### Primer design

Of the 27 candidate gene regions from the *Nannaria* transcriptomes, eleven were selected for primer design, and ultimately three newly developed gene regions were then used in phylogenetic analyses, based on amplification success (> 90%) and sequencing success (> 90% contig rate): large subunit ribosomal RNA gene (16S), fbox domain (fbox), RNA polymerase II largest subunit (RPB1). These three gene regions were then combined with the three other gene regions standardly used in millipede molecular phylogenetics: COI, 28S, and EF1a (see below).

**Table 1.** Partitions used in MrBayes phylogenetic analysis of molecular data. PIC = Parsimony Informative Characters.

Partition	Gene region	Best-fit models	PIC
1	16S	GTR+I+G	368
2	CO1 (3^rd^ CP)	GTR+I+G	154
3	CO1 (1^st^ CP)	GTR+I+G	34
4	CO1 (2^nd^ CP)	GTR+G	5
5	fbox (1^st^ CP), 28S	GTR+I+G	121
6	fbox (3^rd^ CP), EF1a (3^rd^ CP)	GTR+G	63
7	EF1a (1^st^ & 2^nd^ CPs), RPB1 (1^st^ & 2^nd^ CPs)	K80 +I+G	40
8	EF1a (intron)	HKY+G	69
9	fbox (2^nd^ CP)	JC+I	10
10	RPB1 (intron & 3^rd^ CP)	HKY+I+G	128

### Sequence alignment and phylogenetic inference

**Figure 7.**
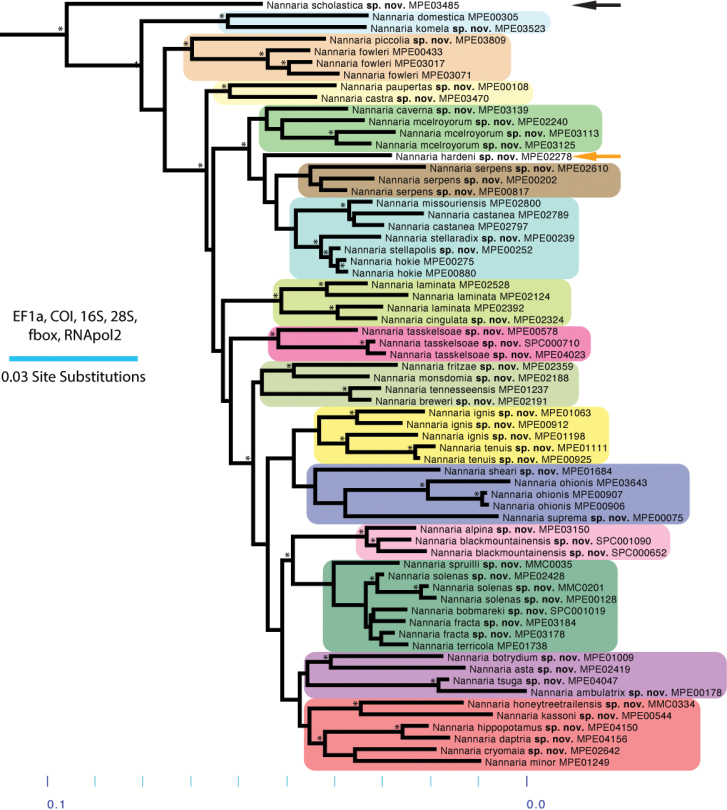
Molecular phylogeny of the *minor* species group. Asterisks indicate a posterior probability of > 0.95. Colored boxes and arrows indicate clades: black arrow, *Nannariascholastica* sp. nov.; sky blue box, *N.domestica* clade; orange box, *N.fowleri* clade; light yellow box, *N.paupertas* clade; green box, *N.mcelroyorum* clade; burnt orange arrow, *N.hardeni* sp. nov.; brown box, *N.serpens* clade; turquoise box, *N.castanea* clade; lime green box, *N.laminata* clade; dark pink box, *N.tasskelsoae* clade; camo green box, *N.tennesseensis* clade; yellow box, *N.ignis* clade; blue box, *N.ohionis* clade; light pink box, *N.blackmountainensis* clade; forest green box, *N.terricola* clade; purple box, *N.ambulatrix* clade; red box, *N.minor* clade.

The concatenated matrix contained six genes, 152 taxa, and a total length of 5,354 bp, as follows: 16S (1–1016), COI (1017–1553), 28S (1554–3178), EF1a (3179–3879), fbox (3880–4298), and RPB1 (4299–5354; for a list of taxa and NCBI accession numbers for specific gene regions see Suppl. material 2). The six genes were divided in PartitionFinder into ten partitions (Table 1). Of the 5,354 characters, 3,787 were constant, 992 were parsimony-informative, and 575 characters were variable and parsimony-uninformative. Observed mean base pair composition for the 5,354 characters was *A* = 0.235, *C* = 0.197, *G* = 0.263, *T* = 0.303, and for each gene fragment as given in Table 2. For each gene region, nucleotide frequency was homogeneous across taxa (*P* < 0.05, df = 3), with no taxa failing the χ^2^ test of stationarity (Table 2). Mean uncorrected percent difference of COI sequences between *Nannaria* species in the *minor* group was 9.8% (maximum: 14%, minimum: 0.0%, standard deviation: 1.8%). In the MrBayes analysis, likelihood values converged, as indicated by the average standard deviation of split frequencies reaching < 0.01, after 210 million generations, and one quarter of the generated trees were then discarded as burnin. A topology was generated from the six-gene concatenated matrix, with 94 of the 151 nodes having posterior probabilities ≥ 0.95. Due to the lack of possible gonopod-based identification, 33 female specimens were pruned from the *minor* species group tree post-analysis, and a tree with only *minor* species group specimens that are identifiable and diagnosable is presented in Fig. 7. To view the unpruned tree including outgroups, the *wilsoni* group and *minor* group female specimens, see Suppl. material 3.

**Table 2.** Nucleotide base frequencies and average gap/ambiguity (%) by gene.

Gene region	A	C	G	T	Av. % Gap/Ambiguity
16S	0.27	0.07	0.23	0.42	30.72
COI	0.19	0.14	0.25	0.42	0.74
EF1a	0.26	0.23	0.25	0.26	24.48
28S	0.15	0.29	0.36	0.20	7.96
fbox	0.23	0.27	0.26	0.23	0.01
Rpb1	0.31	0.18	0.23	0.29	6.94

### Taxonomic notes

The Nannariini was recovered as a monophyletic group (posterior probability, *pp* = 1), with *Oenomaeapulchella* sister to *Nannaria* (*pp* = 1; Suppl. material 3). Eurymerodesmidae was not recovered as sister to the Nannariini, however in light of recent genetic findings using a much greater sample of Eurymerodesmidae and Xystodesmidae, this result is likely due to low taxon sampling of other members of the subfamily here (Means et al. 2021). The genus *Nannaria* is split between two clades, the *wilsoni* and *minor* species groups (*pp* = 1), a relationship that is also recovered in five of the six individual gene trees (Suppl. material 4). The *wilsoni* species group contains many hypothesized undescribed species that will be described in the second part of the revision of *Nannaria*. The *minor* group species was made up of 18 divergent clades representing new species (Fig. 7), including the taxa *Nannariascholastica* sp. nov. and *Nannariahardeni* sp. nov., and a clade consisting entirely of indeterminate female specimens shown in Suppl. material 3 (Murphy clade, eggplant purple box). The *minor* group clades possess a variety of support values (average *pp* = 0.64; Figs 7, 113; Suppl. material 3). Three of the 45 species were paraphyletic (*Nannariaignis* sp. nov., *Nannariafracta* sp. nov., and *Nannarialaminata* Hoffman, 1949); however, we opted to recognize these taxa due to divergent gonopodal morphology and meeting our species delimitation criteria.

### Color

Of 171 *Nannaria* specimens recorded for color, 148 (86.5%) had orange, pink, or red paranota with a dark brown or black background (as in Fig. 89B), while only 23 (13.5%) had white paranota (as in Fig. 89A). Twenty-three (15.5%) of the 148 orange/pink/red *Nannaria* also had metatergal stripes (as in Fig. 97).

### *Nannariaminor* species group diagnoses

The following are diagnoses of 54 species based on our species delimitation criteria (see Materials and Methods). Diagnoses are provided as paragraphs that include a suite of characters that differentiate the species from the most morphologically similar *minor* group species and the sympatric or geographically closest known *wilsoni* group species. Descriptions are provided as a scored morphological matrix for rapid comparisons of species (Suppl. material 5 and 6). Distribution statuses are provided based on three categories, wide-range endemic (WRE): distribution area > 10,000 km^2^; short-range endemic (SRE): distribution area < 10,000 km^2^ and > 1,000 km^2^; and micro-range endemic (MRE): distribution area < 1,000 km^2^ (Harvey 2002; Harvey et al. 2011; Means and Marek 2017). Overall, *Nannaria* species in the *minor* group have restricted distributions, with 91% falling into the SRE or MRE categories. Species descriptions are grouped by clade, and clades are organized as they appear in the molecular phylogeny (Fig. 7).

### *Nannaria* Taxonomy

#### Class Diplopoda de Blainville in Gervais, 1844

**Infraclass Helminthomorpha Pocock, 1887**

**Order Polydesmida Leach, 1815**

**Family Xystodesmidae Cook, 1895**

**Subfamily Rhysodesminae Brolemann, 1916**

##### Tribe Nannariini Hoffman, 1964

###### Genus Nannaria

Taxon classificationAnimaliaPolydesmidaXystodesmidae

Chamberlin, 1918

AF4D94B6-E963-540C-84D0-18D918524C03

####### Type species.

*Nannariaminor* Chamberlin, 1918, by original designation.

####### Other taxa included.

61 species.

####### Diagnosis.

Members of the genus *Nannaria* are distinct from other genera of the subfamily Rhysodesminae, except the monotypic genus *Oenomaea*, by the twisted spatulate shape of the pregonopodal claws in males (Fig. 2) vs. bisinuately curved shape in other genera of Rhysodesminae. *Nannaria* vary from the contribal *Oenomaea* by the more rounded paranota in *Nannaria* (vs. more acute and hook-like in *Oenomaea*) and the unique gonopodal characters of *Oenomaea*, as discussed in Hoffman (1964).

####### Short description.

Small-bodied (17.3–38.5 mm BL, 2.9–6.0 mm B11W) ‘flat-backed’ xystodesmid millipedes. ***Color***: paranota red, orange, white, or (rarely) yellow—rarely connected by concolorus metatergal stripes. Background drab, ranging from pale tan to deep black. ***Male exoskeletal characters***: sterna with paramedian lobes between 4^th^ pair of legs; caudal margins of 8–18 sterna modified into subcoxal spines. Pregonopodal tarsal claws spatulate [(Fig. 2)—note: the twisted spatulate shape of the pregonopodal claws in males is a unique autapomorphy and uniformly present in the tribe Nannariini (*Oenomaeapulchella* and *Nannaria* species)]. Postgonopodal tarsal claws bisinuate. ***Male gonopodal characters***: gonopods variable, often crossing, when viewed in situ; co-planar with coxae, lacking torsion, generally simple in comparison to other Rhysodesminae—though some species display elaborate curvature and ornamentation. Gonopods never bulky as in some Apheloriini. Prefemoral process present. Prostatic groove originating at cannula, terminating at acropodite tip. Acropodite, when viewed ventrally, c- or r-shaped. Gonopodal aperture lacking modified sternum. Gonocoxae connected by membrane and muscle.

####### Distribution.

*Nannaria* can be found in much of the eastern United States (Fig. 1): western Arkansas east to just outside Philadelphia (Pennslyvania), north to western New York, and south to central Mississippi. The southeastern edge of the distribution follows the Appalachian Mountains closely, though specimens from central North Carolina and Mississippi suggest that populations may eventually be found south of the Appalachians in states such as Texas and Florida. Likewise, scattered populations may exist in central and southern Illinois, as seen in neighboring Indiana and Missouri.

####### Etymology.

Derived from the Greek word *nannos*, for dwarf, referring to the comparatively small size of this genus within the Xystodesmidae.

##### Key to the *minor* species group of *Nannaria* Chamberlin, 1918

**Table:** 

1	Gonopods fairly straight, parallel for most of length, rarely crossing when viewed in situ (Fig. 8A)	**2**
–	Gonopods curved, often crossing when viewed in situ (Fig. 8B)	**10**
2 (1)	Prefemoral basal spine fused with prefemoral process for entire length (Fig. 86B). Ohio	***N.terricola*** (Figs 86, 87)
–	Prefemoral basal spine separate from prefemoral process for part of length (Fig. 88A)	**3**
3 (2)	Prefemoral basal spine large, pronounced (Fig. 57A)	**4**
–	Prefemoral basal spine small, reduced (Fig. 88C)	**5**
4 (3)	Prefemoral basal spine pointed, sharp (Fig. 80C). Acropodite with small, shelf-like medial flange just before tip (Fig. 80C, red arrow). Telopodite basal zone with pronounced medial swelling (Fig. 80A, red circle). Virginia – Kentucky border	***N.fracta* sp. nov.** (Figs 80, 81)
–	Prefemoral basal spine pointed, blunt (Fig. 57A). Distal zone much reduced, rounded, with small, lobed lateral flange (Fig. 57A, red triangle). Prefemur with large, laminate prefemoral process, curving medially, crossing acropodite and expanding before tip. Knox Co., Tennessee	***N.monsdomia* sp. nov.** (Figs 57, 58)
5 (4)	Prefemoral proccess arising from prefemoral spine, dorsomedially (Fig. 88A). Distal zone short, directed dorsomedially, with large, triangular lateral flange (Fig. 88A, red arrow). Telopodite basal zone large, subequal to length of acropodite. Southwestern Virginia	***N.ambulatrix* sp. nov.** (Figs 88, 89)
–	Prefemoral process arising from prefemur (Fig. 54A)	**6**
6 (5)	Prefemoral process sinuous, serpentine (Fig. 69A, B). Distal zone short, directed dorsomedially, with large, triangular lateral flange (Fig. 69A, red arrow). Virginia – Tennessee border	***N.suprema* sp. nov.** (Figs 69, 70)
–	Prefemoral process variable, though never sinuous, serpentine	**7**
7 (6)	Prefemoral process laminate (Fig. 54A). Distal zone much reduced, with rounded, simple tip. Acropodite with medial swelling. Acropodite appearing as straight line in medial view. Knox Co., Tennessee	***N.equalis*** (Fig. 54)
–	Prefemoral process acicular (Fig. 100A)	**8**
8 (7)	Gonopod basal zone with slight lateral bulge (Fig. 100A). Acropodite tip with prominent lobed lateral flange (Fig. 100A, red arrow). Acropodite swollen medially before apex (Fig. 100A, red triangle). Greene Co., Tennessee	***N.hippopotamus* sp. nov.** (Figs 100, 101)
–	Gonopod basal zone without lateral bulge (Fig. 85A)	**9**
9 (8)	Gonopod basal zone height, when viewed anteriorly, subequal to half length of acropodite. Acropodite straight. Distal zone short, rectangular in composition when viewed anteriorly, bent medially at 90° angle with slight cephalically-directed upturn at terminal edge (Fig. 82A, red arrow). Southwestern Virginia, southern West Virginia	***N.solenas* sp. nov.** (Figs 82–84)
–	Gonopod basal zone height, when viewed anteriorly, greater than half length of acropodite. Acropodite straight, curving medially at nearly 90° angle at apex. Acropodite tip with small, rounded lateral flange (Fig. 85C, red arrow). Prefemur with straight, acicular prefemoral process and reduced prefemoral spine (Fig. 85C, red triangle). Wise Co. Virginia	***N.spruilli* sp. nov.** (Fig. 85)
10 (1)	Prefemoral basal spine absent (Fig. 71B)	**11**
–	Prefemoral basal spine present (Fig. 90B)	**16**
11 (10)	Prefemoral basal spine fused with prefemoral process for entire length (Fig. 38A). Acropodite long, curving medially. Distal zone with medial and lateral flanges at 90° angle to solenomere (Fig. 38B, red arrow). Acropodite with small medial flange near apex (Fig. 38A, red triangle). Missouri	***N.missouriensis*** (Figs 38, 39)
–	Prefemoral basal spine separate from prefemoral process for some part of length (Fig. 88A)	**12**
12 (11)	Prefemoral process thin, acicular (Fig. 110A). Distal zone short, simple, curving smoothly posteriorly, with a crochet hook-like appearance (Fig. 110A, red triangle). Prefemoral spine lacking. Pennsylvania	***N.oblonga*** (Fig. 110)
–	Prefemoral process variable, though never thin, acicular	**13**
13 (12)	Prefemoral process laminate (Fig. 54A)	**14**
–	Prefemoral process simple, curving (Fig. 71A). Acropodite tip with small, triangular lateral flange (Fig. 71A, red arrow). Telopodite basal zone with slight lateral bulge (Fig. 71A, red triangle). Gap between prefemoral process and acropodite greater than width of acropodite basal zone. Pulaski Co. Kentucky	***N.alpina* sp. nov.** (Figs 71, 72)
14 (13)	Prefemoral process curving medially, when viewed anteriorly (Fig. 20A). Distal zone curving dorsally, with tip directed caudally (Fig. 20B). Acropodite tip with lateral flange (Fig. 20A, red arrow). Acropodite with slight twist and swelling at midpoint (Fig. 20C, red triangle). Mercer Co., West Virginia	***N.castra* sp. nov.** (Figs 20, 21)
–	Prefemoral process variable, though never curving medially, when viewed anteriorly	**15**
15 (14)	Prefemoral process straight, when viewed anteriorly (Fig. 108A). Putnam Co., Tennessee	***N.rhysodesmoides*** (Fig. 108)
–	Prefemoral process curves laterally, when viewed anteriorly (Fig. 9A). Prefemoral process with slight constriction at base (Fig. 9C, red triangle). Prefemoral spine lacking. Distal zone twisted dorsolaterally (Fig. 9). Tip directed dorsolaterally, flattened, with enlarged acuminate flange on inner margin (Fig. 9B, red arrow). Rockbridge Co., Virginia	***N.scholastica* sp. nov.** (Figs 9, 10)
16 (10)	Prefemoral basal spine fused with prefemoral process for entire length (Fig. 38A)	**17**
–	Prefemoral basal spine separate from prefemoral process for some part of length (Fig. 88A)	**23**
17 (16)	Prefemoral basal spine reduced to a rounded bulge (Fig. 92C)	**18**
–	Prefemoral basal spine rectangular, shelf-like (Fig. 52C)	**19**
18 (17)	Prefemoral process tip directed medially (Fig. 92A). Prefemoral process long thin, bent 90° and directed medially at half-way point (Fig. 92A, red arrow). Prefemoral spine reduced and fused with prefemoral process for entire length (Fig. 92C, red triangle). Bland and Tazewell Cos., Virginia, Randolph Co. West Virginia	***N.botrydium* sp. nov.** (Figs 92, 93)
–	Prefemoral process tip directed dorsally (Fig. 63B). Prefemoral spine reduced to slight swelling at base of prefemoral process (Fig. 63C, red triangle). Acropodite tip rounded with small, rounded lateral flange (Fig. 63A, red arrow). Acropodite with medial swelling before apex. Bland Co., Virginia	***N.tenuis* sp. nov.** (Figs 63, 64)
19 (17)	Prefemoral process simple, curving medially (Fig. 52A). Prefemoral tip bent dorsally (Fig. 52B). Prefemoral spine fused to prefemoral process throughout length, forming ridge along base of prefemoral process (Fig. 52C, red triangle). Acropodite distal zone with large, lobed lateral flange (Fig. 52A, red arrow). Southern West Virginia, Southwestern Virginia, Northeastern Tennessee	***N.breweri* sp. nov.** (Figs 52, 53)
–	Prefemoral process variable, though never curving simple, medially	**20**
20 (19)	Prefemoral process acicular (Fig. 59A)	**21**
–	Prefemoral process variable, though never acicular	**22**
21 (20)	Gonopod basal zone height, when viewed anteriorly, subequal to half length of acropodite (Fig. 73A). Acropodite curving dorsomedially with pronounced medial swelling (Fig. 73A). Acropodite tip with large, hooked lateral flange (Fig. 73A, red arrow). Prefemoral spine reduced and fused to prefemoral process, forming small ridge (Fig. 73B, red triangle). Cumberland Co., Tennessee, East to confluence of Tennessee, Kentucky, North Carolina and Virginia	***N.blackmountainensis* sp. nov.** (Figs 73, 74)
–	Gonopod basal zone height, when viewed anteriorly, greater than half length of acropodite (Fig. 59A). Acropodite distal zone heavily reduced, with small, triangular lateral flange (Fig. 59B, red arrow). Prefemoral spine fused to prefemoral process throughout length (Fig. 59A, red triangle). Eastern Tennessee	***N.tennesseensis*** (Figs 59, 60)
22 (20)	Prefemoral process laminate (Fig. 67A). Prefemoral process curving dorsomedially (Fig. 67B). Prefemoral spine fused with prefemoral process, reduced to shelf-like ridge at base of prefemoral process (Fig. 67C, red triangle). Acropodite distal zone curving medially, with tip directed caudally (Fig. 67A, red arrow). Mercer Co., West Virginia	***N.sheari* sp. nov.** (Figs 67, 68)
–	Prefemoral process sinuous (Fig. 13A). Prefemoral spine reduced and fused with prefemoral process, forming basal ridge (Fig. 13C, red triangle). Acropodite semi-circular, curving dorsomedially throughout, giving appearance of slight swelling just basal to tip in posterior view (Fig. 13C, red arrow). Carroll Co., Virginia, Surry Co., North Carolina	***N.komela* sp. nov.** (Figs 13, 14)
23 (16)	Prefemoral basal spine large, pronounced (Fig. 57A)	**24**
–	Prefemoral basal spine small, reduced (Fig. 88C)	**43**
24 (23)	Prefemoral basal spine rectangular, shelf-like (Fig. 40A). Acropodite long and curving medially before apex. Acropodite tip with small, triangular lateral flange (Fig. 40A, red arrow). Telopodite basal zone height ca. 1/2 length of acropodite. Roanoke Co., Virginia	***N.stellapolis* sp. nov.** (Figs 40, 41)
–	Prefemoral basal spine variable, though never rectangular, shelf-like	**25**
25 (24)	Prefemoral basal spine pointed, blunt (Fig. 57A)	**26**
–	Prefemoral basal spine variable, though never pointed, blunt	**28**
26 (25)	Prefemoral process arising from prefemur (110A). Prefemoral process straight, laminate, separated widely from projected, blunt prefemoral spine (Fig. 94A, red triangle). Acropodite distal zone short, directed dorsomedially, with large, lobed lateral flange (Fig. 94A, red arrow). Sullivan Co. Tennessee	***N.tsuga* sp. nov.** (Figs 94, 95)
–	Prefemoral process arising dorsomedially from prefemoral spine (Fig. 102A)	**27**
27 (26)	Prefemoral process stout (Fig. 102A). Acropodite tip blunt, with small, triangular lateral flange (Fig. 102A, red arrow). Acropodite swollen before apex (Fig. 102A, red triangle). Telopodite basal zone height > 1/3 length of acropodite. Lee Co. Virginia	***N.honeytreetrailensis* sp. nov.** (Figs 102, 103)
–	Prefemoral process thin, sinuous (Fig. 112A). Meigs Co. Tennessee	***N.sigmoidea*** (Fig. 112)
28 (25)	Prefemoral process arising from the top of the prefemoral spine (Fig. 32A)	**29**
–	Prefemoral process variable, though never arising from the top of the prefemoral spine	**32**
29 (28)	Prefemoral process sinuous (Fig. 112A). Acropodite apex with distinct constriction (Fig. 96A, red arrow). Tip expanded distally, with small lobed lateral and medial flanges. Acropodite with expanded medial flange (Fig. 96A, red triangle). Morgan Co. Tennessee	***N.cryomaia* sp. nov.** (Figs 96, 97)
–	Prefemoral process straight, acicular (Fig. 32A)	**30**
30 (29)	Prefemoral process straight, when viewed anteriorly, arising from top of prefemoral spine (Fig. 75A). Prefemoral spine large, projecting, acicular. Acropodite curving medially before apex (Fig. 65). Southeastern Ohio, northwestern West Virginia	***N.ohionis*** (Figs 65, 66)
–	Prefemoral process curves medially, when viewed anteriorly (Fig. 32A)	**31**
31 (30)	Gonopod basal zone height, when viewed anteriorly, subequal to half-length of acropodite (Fig. 73A). Acropodite long, thin, curving medially. Distal zone with medial flange at 135° to solenomere, and lateral flange at 90° angle to solenomere (Fig. 32B, red triangle). Prefemoral process small, thin, curving medially, arising from top of projected, stout prefemoral spine (Fig. 32A, red arrow). Mississippi north to Indiana, west to Arkansas and Missouri	***N.castanea*** (Figs 32, 33)
–	Gonopod basal zone height, when viewed anteriorly, less than half length of acropodite (Fig. 35A). Acropodite long and curving ventromedially. Distal zone bent ventroposteriorly, with laminate flange encircling tip forming a hood-like structure around dorso-posteriorly projected, laminate solenomere — laminate flange partially obscuring solenomere when viewed laterally (Fig. 35A, red arrow). Acropodite with small medial flange near apex (Fig. 35C, red triangle). Prefemoral process small, thin, curving medially, arising from top of projected acuminate prefemoral spine. Southwestern Virginia	***N.hokie*** (Figs 35–37)
32 (28)	Prefemoral process arising from prefemur (Fig. 98A)	**33**
–	Prefemoral process arising dorsomedially from prefemoral spine (Fig. 102A)	**36**
33 (32)	Prefemoral process simple, curving medially (Fig. 98A). Prefemoral spine prominent, claw-like, curving cephalically. Acropodite tip blunt, with small, triangular lateral and medial flanges (Fig. 98A, red arrows). Acropodite swollen before apex (Fig. 98A, red triangle). Greene Co., Tennessee	***N.daptria* sp. nov.** (Figs 98, 99)
–	Prefemoral process variable, though never simple, curving medially	**34**
34 (33)	Prefemoral process sinuous, bending ventrally before curving cephalolaterally, arising dorsomedially from large, sharp prefemoral spine (Fig. 61B). Distal zone of acropodite curving dorsomedially (Fig. 61B, red arrow). Tip of acropodite with small, rounded lateral flange (Fig. 61A, red triangle). Bland and Wythe Cos., Virginia	***N.ignis* sp. nov.** (Figs 61, 62)
–	Prefemoral process variable, though never sinuous, bending ventrally	**35**
35 (34)	Prefemoral process curves medially, when viewed anteriorly (Fig. 109A). Prefemoral spine acicular and paralleling prefemoral process (Fig. 109B, red arrow). Acropodite tip simple, blunt. Central North Carolina	***N.conservata*** (Fig. 109)
–	Prefemoral process straight, acicular (Fig. 28A). Prefemoral spine sharp, tooth-like, widely separated from prefemoral process (Fig. 28A, red arrow). Acropodite curving medially before apex, distal zone reduced. Henry and Pittsylvania Cos., Virginia	***N.hardeni* sp. nov.** (Figs 28, 29)
36 (32)	Prefemoral process stout, acicular, arising dorsomedially from projected cephalically-curving prefemoral spine (Fig. 90A). Acropodite distal zone curving dorsomedially, tip rounded, directed caudally with small lateral flange (Fig. 90A). Acropodite with slight swelling on inner margin (Fig. 90B, red triangle) and dimple on outer margin (Fig. 90A, red arrow). Bland Co., Virginia	***N.asta* sp. nov.** (Figs 90, 91)
–	Prefemoral process variable, though never stout, acicular	**37**
37 (36)	Prefemoral process sinuous (Fig. 24A)	**38**
–	Prefemoral process simple, curving in a single arc (Fig. 104A)	**42**
38 (37)	Prefemoral process curves medially, when viewed anteriorly (Fig. 26A)	**39**
–	Prefemoral process curves laterally, when viewed anteriorly (Fig. 61A)	**41**
39 (38)	Prefemoral process tip directed ventrally (Fig. 26B)	**40**
–	Prefemoral process tip directed cephalically (Fig. 111). Prefemoral process long, thin, curving medially, arising from sharp projected prefemoral spine (Fig. 111A, red arrow). Acropodite simple, curving medially throughout with extremely short, blunt distal zone. Rutherford Co., North Carolina	***N.rutherfordensis*** (Fig. 111)
40 (39)	Basal zone width, when viewed anteriorly, subequal to space between acropodite and prefemoral process at greatest divide (Fig. 30A). Acropodite gradually tapering towards tip, with small, lobed medial flange (Fig. 26A, red triangle). Tip directed medially, rounded and simple. Prefemoral process thin, sinuous, acicular. Prefemoral spine small, medially directed, with secondary hump proximal to acropodite base (Fig. 26C, red arrow). Western West Virginia, northeastern Kentucky	***N.mcelroyorum* sp. nov.** (Figs 26, 27)
–	Basal zone width, when viewed anteriorly, wider than space between acropodite and prefemoral process at greatest divide (Fig. 24A). Acropodite curving medially before bending ventromedially at a nearly 90° angle (Fig. 22B, red triangle). Distal zone short, bent dorsomedially at 90° angle. Acropodite shaft swollen before apex, with medial flange, and sinuous region. Prefemoral process curving ventromedially, coplanar (when viewed medially) with acropodite. Prefemoral spine prominent, acicular. Montgomery Co., Virginia	***N.paupertas* sp. nov.** (Figs 22, 23)
41 (38)	Prefemoral process tip directed ventrally (Fig. 24A). Prefemoral process arising from large, pronounced prefemoral spine (Fig. 24B, red arrow). Acropodite linear, bent medially forming 130° angle with telopodite basal zone. Tip simple, rectangular, bent medially. Carter Co., Kentucky	***N.caverna* sp. nov.** (Figs 24, 25)
–	Prefemoral process tip directed cephalically (Fig. 30A). Prefemoral spine enlarged, cephalically directed. Acropodite with slightly swollen medial area (Fig. 30C, red triangle), and small, lobed medial flange (Fig. 30A, red arrow) before apex. Tip directed medially, blunt and simple. Distal zone greatly reduced. Southwestern Virginia, northeastern North Carolina	***N.serpens* sp. nov.** (Figs 30, 31)
42 (37)	Prefemoral process tip directed medially (Fig. 104A). Prefemoral process paralleling curve of acropodite, arising dorsolaterally from pronounced curving prefemoral spine. Acropodite apex with distinct constriction (Fig. 104A, red arrow), tip sharp, with acuminate, triangular lateral flange. Acropodite with expanded, laminate medial flange (Fig. 104A, red triangle). Campbell Co., Tennessee	***N.kassoni* sp. nov.** (Figs 104, 105)
–	Prefemoral process tip directed ventrally (Fig. 42A). Prefemoral process arising from enlarged prefemoral spine. Acropodite distal zone short, directed medially with small triangular lateral flange (Fig. 42A, red triangle). Floyd and Pulaski Cos., Virginia	***N.stellaradix* sp. nov.** (Figs 42, 43)
43 (23)	Prefemoral basal spine pointed, sharp (Fig. 45C)	**44**
–	Prefemoral basal spine variable, though never pointed, sharp	**52**
44 (43)	Prefemoral process arising from prefemur (Fig. 45A)	**45**
–	Prefemoral process arising dorsomedially from prefemoral spine (Fig. 15A)	**50**
45 (44)	Prefemoral process sinuous (Fig. 45B)	**46**
–	Prefemoral process variable, though never sinuous	**47**
46 (45)	Prefemoral process curves laterally when viewed anteriorly (Fig. 45A). Acropodite shaft swollen before apex, with cingulum (Fig. 45A, red rectangle). Prefemoral spine greatly reduced to small, tooth-like projection (Fig. 45C, red oval). Acropodite distal zone short, bent dorsomedially at 45° angle. Tip blunt with small, lobed lateral flange (Fig. 45C, red arrow). Telopodite basal zone with lateral bulge (Fig. 45A, red triangle). West Virginia panhandle and eastern Virginia	***N.cingulata* sp. nov.** (Figs 45–47)
–	Prefemoral process fairly straight when viewed anteriorly (Fig. 19A), arising from medial side of shelf-like prefemoral spine (Fig. 19C, red arrow). Acropodite simple, without cingulum or modifications. Distal zone short, simple, at nearly 90° angle to acropodite. Border region between central West Virginia and Virginia	***N.simplex*** (Fig. 19)
47 (45)	Prefemoral process acicular (Fig. 34A)	**48**
–	Prefemoral process laminate and sinuous (Fig. 48A). Prefemoral spine reduced to small, acuminate projection (Fig. 48C, red arrow). Acropodite semi-circular, curving dorsomedially with abrupt 90° curve after apex. Acropodite tip simple, directed posteriorly, terminating in sharp, claw-like point. Central to southwestern Virginia, southeastern West Virginia	***N.laminata*** (Figs 48, 49)
48 (47)	Prefemoral process straight, when viewed anteriorly, arising from small, blunt prefemoral spine (Fig. 34A). Acropodite distal zone with medial and lateral flanges at 90° angle to solenomere (Fig. 34A, red arrow). Acropodite with small medial flange near apex (Fig. 34A, red triangle). Newton Co., Arkansas	***N.davidcauseyi*** (Fig. 34)
–	Prefemoral process otherwise	**49**
49 (48)	Prefemoral process curving medially, when viewed anteriorly (Fig. 55A). Prefemoral spine reduced to thorn-like structure, arising from prefemoral process, directed ventrally (Fig. 55B, red triangle). Acropodite distal zone short, rounded. Floyd Co., Georgia	***N.fritzae* sp. nov.** (Figs 55, 56)
–	Prefemoral process curves laterally, when viewed anteriorly (Fig. 106A). Prefemoral spine small, curving cephalically (Fig. 106B, red triangle). Acropodite distal zone extremely short, blunt. Northern border of Tennessee and North Carolina, and Loudon Co., Tennessee	***N.minor*** (Figs 106, 107)
50 (44)	Prefemoral process sinuous, arising from base of stout, tooth-like prefemoral spine, paralleling medial curve of acropodite (Fig. 15A). Acropodite distal zone short, curving medially forming 130° angle with acropodite (Fig. 15). Tip and distal zone simple, rectangular, blunt. Northeastern Virginia and eastern West Virginia north to southwestern New York	***N.fowleri*** (Figs 15, 16)
–	Prefemoral process otherwise	**51**
51 (50)	Prefemoral process stout, curving laterally (Fig. 17A). Prefemoral spine stout, tooth-like (Fig. 17C, red arrow). Alleghany and Rockbridge Cos., Virginia	***N.piccolia* sp. nov.** (Figs 17, 18)
–	Prefemoral process simple, curving medially (Fig. 50A). Prefemoral process arising dorsally from prefemoral spine (Fig. 50A, red arrow), paralleling medial curve of acropodite. Prefemoral spine large, curving cephalically. Eastern West Virginia	***N.tasskelsoae* sp. nov.** (Figs 50, 51)
52 (43)	Prefemoral basal spine reduced to rounded bulge (Fig. 78C). Prefemoral process dorsomedially curving. Acropodite tip with prominent triangular lateral flange curving abruptly at 90° angle towards tip (Fig. 78A, red triangle). Acropodite with laminate medial flange just proximal to tip. Davidson and Marshall Cos. Tennessee	***N.dilatata*** (Figs 78, 79)
–	Prefemoral basal spine rectangular in conformation, shelf-like (Fig. 75C)	**53**
53 (52)	Prefemoral process long, acicular (Fig. 75A). Prefemoral spine pronounced, sharp, partially fused to prefemoral process forming a ridge (Fig. 75C, red triangle). Acropodite acicular, bending abruptly medially at 90° angle at tip. Distal zone quadrate. Acropodite with small, shelf-like medial flange just before tip (Fig. 17C, red arrow). Telopodite basal zone with slight medial swelling (Fig. 75A, red circle). Harlan and Leslie Cos., Kentucky	***N.bobmareki* sp. nov.** (Figs 75, 76)
–	Prefemoral process large, laminate and sinuous (Fig. 11A). Prefemoral spine reduced to small, rectangular shelf. Acropodite semi-circular, gently curving dorsomedially throughout. Acropodite tip with small, triangular lateral flange (Fig. 11A, red triangle). Tip terminating in sharp, caudally-directed point. Watauga Co., North Carolina	***N.domestica*** (Figs 11, 12)

**Figure 8.**
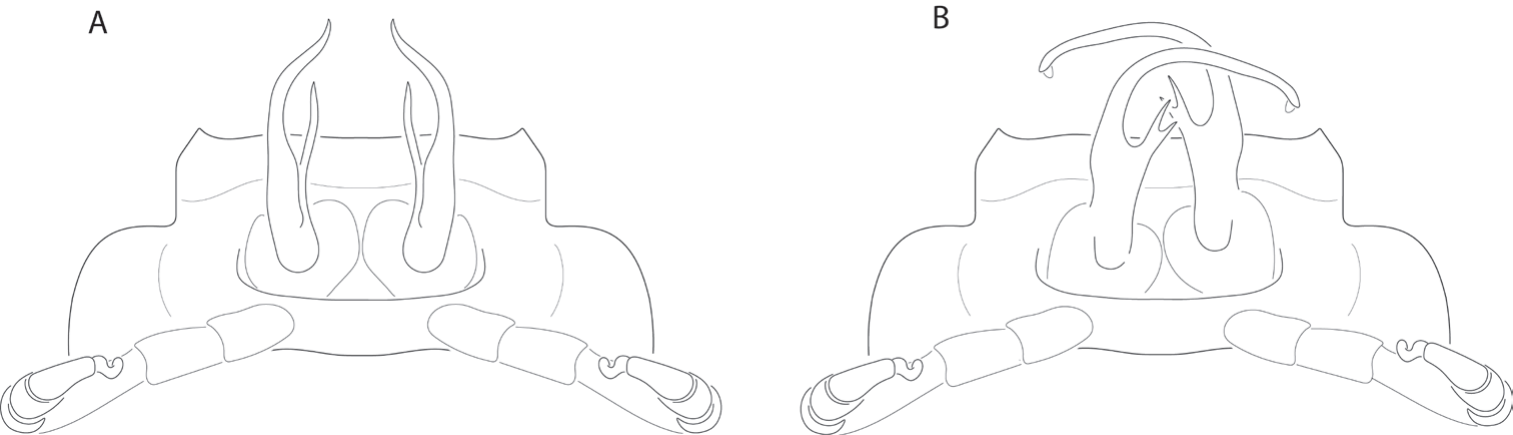
Two general types of gonopods in the *minor* species group of *Nannaria* Chamberlin, 1918 **A***Nannariaambulatrix* sp. nov. (non-type ♂, VTEC, MPE03794), fairly straight gonopods which do not cross in situ **B***N.hokie* Means, Hennen & Marek, 2021 (paratype ♂, VMNH, MPE00886), more highly curved gonopods which do cross in situ.

##### *minor* species group diagnosis

Species in the *minor* species group differ from species in the *wilsoni* species group based on the presence of the following characters (best viewed with ≥ 20× magnification): acropodite often highly setaceous throughout, not with setation ending at midpoint as in the *wilsoni* species group. Acropodite anterior bend lacking twist as seen in the *wilsoni* species group. Prefemoral process often with prefemoral spine, lacking in the *wilsoni* species group. Without cyphopod receptacle modification and expanded 2^nd^ coxae of females, as is present in the *wilsoni* species group.

Species in the *minor* species group may be confused in field with *Gyalostethusmonticolens* (Chamberlin, 1951), *Boraria* Chamberlin, 1943, *Howellaria* Hoffman, 1950, and *Idaloria* Marek, Means & Hennen, 2021, due to similarity in body length (average 27 mm) and relatively simple acropodites. However, the *minor* species group differs based on the presence of the following characters (best viewed with ≥ 20× magnification): Males with spatulate pregonopodal claws (Fig. 2), not bisinuate or uncinate. Acropodite typically thin, long and continually curving; if straight, lacking S-shaped acropodite tip as in *G.monticolens*. Acropodite tip rarely directed cephalically. With triangular sternal projections, not lacking as in *Gyalostethus* Hoffman, 1965. Color in life without yellow paranotal spots as in *Borariastricta*, or tan/brown as in *G.monticolens*.

##### *scholastica* clade

**Components.***Nannariascholastica* sp. nov. Based on the molecular phylogeny (Fig. 7), *Nannariascholastica* sp. nov. is sister to the remaining species of the *minor* species group. In combination with morphological characters detailed in the below diagnosis, *N.scholastica* sp. nov. is a member of its own monotypic clade (Fig. 113). The basal placement of this species is not entirely surprising, as it has simple gonopods, yet lacks a prefemoral spine, therefore only partially conforming with the rest of the *minor* group. The geographic isolation of this species is worrisome for its conservation. Hoffman had only one specimen (a male) in the VMNH collection, from the campus of Washington and Lee University (WLU). JCM and DAH discovered an abundant and seemingly healthy population during a visit to WLU, however repeated collection trips by the authors, Hoffman, and collaborators in the surrounding mountains have revealed no additional populations. This species may be a relict that is in threat of extinction should the small forest that it now occupies be destroyed or altered.

**Distribution.** The campus of WLU (Fig. 114, black dot).

###### Nannaria scholastica sp. nov.

Taxon classificationAnimaliaPolydesmidaXystodesmidae

279E35D1-ED5E-5137-998F-7E2362A21180

http://zoobank.org/97C73812-E51C-4BCB-9D97-9F145A01590A

**Figure 9.**
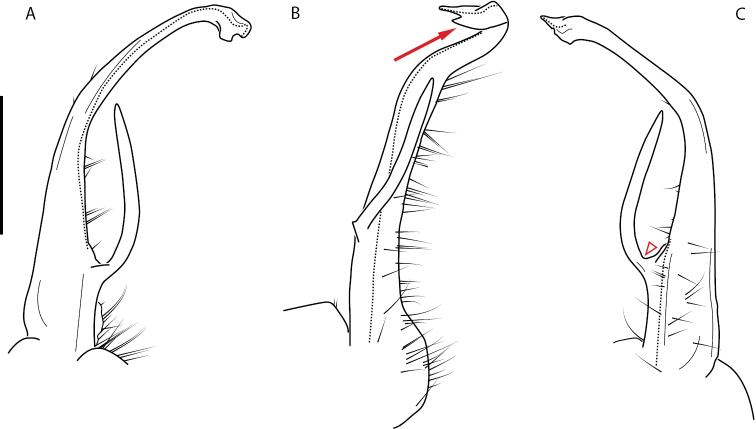
*Nannariascholastica* sp. nov. paratype ♂ (VMNH, NAN0306) left gonopod **A** anterior view **B** medial view; red arrow indicates medial flange **C** posterior view; red triangle indicates slight prefemoral process constriction. Scale bar: 0.5 mm.

**Figure 10.**
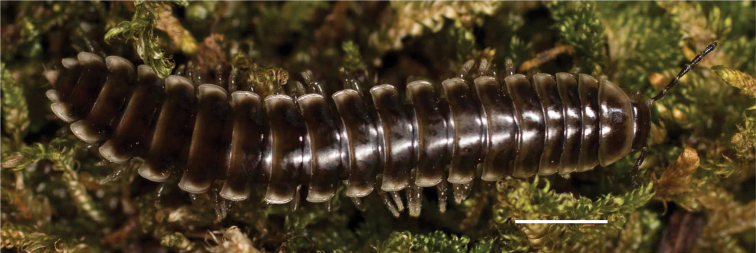
*Nannariascholastica* sp. nov. holotype ♂ (VTEC, MPE03485) coloration. Scale bar: 4.0 mm.

Figs 9 , 10 Vernacular name: “The Scholarly Twisted-Claw Millipede”

####### Material examined.

***Holotype*:** United States – **Virginia** • ♂; Rockbridge County, Lexington, Washington and Lee University campus, hillside beside community gardens; 37.7956°N, -79.4427°W; elev. 330 m; 14 Nov. 2017; hand collected; J. Means, D. Hennen leg.; VTEC MPE03485.

***Paratypes*:** United States – **Virginia** • 4 ♂♂; same collection data as holotype; VTEC MPE03486, 3498, 3500, 3517 • 3 ♂♂; same collection data as holotype; VMNH MPE03516, 3518, 3625 • 1 ♀; same colletion data as holotype; VTEC MPE03499 • 1 ♂; Rockbridge County, W & L campus Lexington; 37.7956°N, -79.4427°W; 20 Nov. 1948; R. Hoffman leg.; VMNH NAN0306. For detailed collection data see Suppl. material 7.

####### Diagnosis.

Adult males of *Nannariascholastica* sp. nov. are distinct from other *Nannaria* and the nearby *N.shenandoa*, based on the following combination of characters: ***Gonopods*.** Gonopodal acropodite gently curving ventromedially before apex, not straight as in *N.terricola*. Distal zone twisted dorsolaterally (Fig. 9). Tip directed dorsolaterally, flattened, with enlarged acuminate flange on inner margin (Fig. 9B, red arrow), not with folds, grooves, flanges as *N.shenandoa*. Telopodite lacking swollen basal zone of *N.terricola* and heavily reduced basal zone of *N.shenandoa*. Prefemur with curved, saber-like prefemoral process, not straight as in *N.terricola*. Prefemoral process (when viewed medially) not crossing the acropodite as in *N.shenandoa*. Prefemoral process slightly constricted at base (Fig. 9C, red triangle), one-half length of acropodite. Prefemur lacking prefemoral spine. ***Color*.** Tergites with white paranotal spots (Fig. 10). Dark brown background. Dorsum of collum smooth with white margin.

####### Measurements.

♂ holotype (VTEC, MPE03485): BL = 32.5, CW = 4.2, IW = 2.1, ISW = 0.9, B11W = 5.4, B11H = 3.2; ♀ paratype (VTEC, MPE03499): BL = 34.2, CW = 3.9, IW = 2.5, ISW = 1.0, B11W = 5.4, B11H = 3.8.

####### Variation.

No known variation.

####### Distribution.

Known only from the type locality (Virginia: Rockbridge County, Suppl. material 7; Figs 114, 126). Distribution area: N/A; status: MRE.

####### Ecology.

Individuals of *N.scholastica* sp. nov. were collected from a mesic mixed pine-deciduous forest composed of maple, tuliptree, black cherry, beech, and white pine. They were found under leaf litter on top of the soil. *Nannariascholastica* sp. nov. was found along with an undescribed species of *Nannaria* in the *wilsoni* species group; however, the two species had ostensibly stratified themselves in two longitudinal strips, with the *wilsoni* species at the top of the hill, and *N.scholastica* sp. nov. ca. 10 m lower down on the slope. Similar specific elevational spacing has been observed in *N.serpens* sp. nov. and *N.stellaradix* sp. nov.; however, in this case the *minor* group *N.serpens* sp. nov. and *N.stellaradix* sp. nov. were found from the higher elevation areas of the hill, while the undescribed *wilsoni* group species were found at the bottom of the hill.

####### Etymology.

This species is named for its discovery on the campus of an academic institution, Washington and Lee University. The specific name *scholastica* is Latin for academic, and is a feminized adjective.

####### Type locality.

United States, Virginia, Rockbridge County, Lexington, Washington and Lee University campus hillside beside community gardens, 37.7956°N, -79.4427°W.

##### *domestica* clade

**Components.***Nannariadomestica* Shelley, 1975, *N.komela* sp. nov., and a female from the I-77 NC Welcome Center. Members of the *domestica* clade share a curving, semi-circular acropodite and laminate prefemoral process. Surprisingly, *N.domestica* and *N.komela* sp. nov. are not closely related to *N.laminata*, despite *N.laminata* also having circular gonopods with laminate prefemoral processes. Due to the simplicity of these forms, however, it is likely that similarity between the *N.domestica* clade and *N.laminata* is due to convergence, rather than a shared evolutionary history. Based on the molecular phylogeny (Fig. 7) the female from the I-77 NC Welcome Center would appear to be the same species as *N.komela* sp. nov.

**Distribution.** the *domestica* clade is known from a small area in southwestern Virginia and northwestern North Carolina (Fig. 114).

###### Nannaria domestica

Taxon classificationAnimaliaPolydesmidaXystodesmidae

Shelley, 1975

C104EE0A-F2D7-50FA-A6BB-71441F1C625C

**Figure 11.**
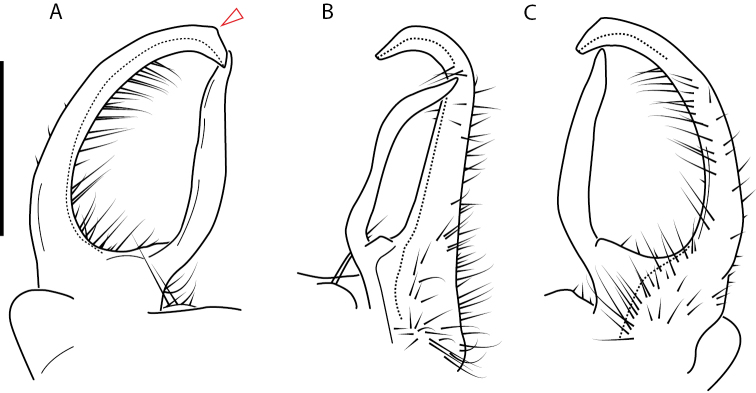
*Nannariadomestica* ♂ (VTEC, MPE00305) left gonopod **A** anterior view; red triangle indicates lateral flange **B** medial view **C** posterior view. Scale bar: 0.5 mm.

**Figure 12.**
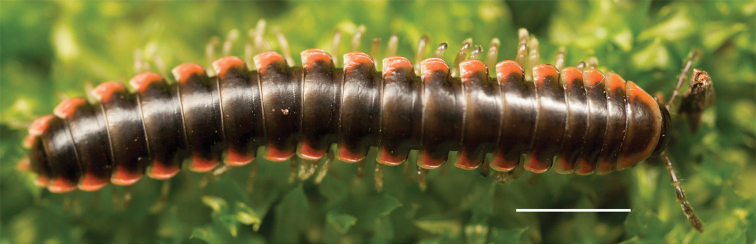
*Nannariadomestica* ♂ (VTEC, MPE00305) coloration. Scale bar: 4.0 mm.

Figs 11 , 12 Vernacular name: “The Domestic Twisted-Claw Millipede”

Nannaria domestica Shelley, 1975: 186, figs 10–12. Hoffman 1999: 366. Shelley 2000: 196. Marek et al. 2014: 36. Means et al. 2021: S69.

####### Material examined.

United States – **North Carolina** • 1 ♂; Watauga County, Cliff Dwellers Inn, side of road, slope; 36.1386°N, -81.6694°W; elev. 1018 m; 25 Apr. 2015; hand collected; J. Means leg.; VTEC MPE00305 • 6 ♀♀; same collection data as preceding; VTEC MPE00303, 310–313, 315 • 1 ♂; Watauga County, Blowing Rock, Goforth Rd., 0.5 N US 321; 36.1293°N, -81.6619°W; 11 Oct. 1975; R. Shelley leg.; NCSM NAN0510; 3 ♂♂, 1 ♀; same collection data as preceding; 8 Sep. 1973; NCSM NAN0525. For detailed collection data see Suppl. material 7.

####### Diagnosis.

Adult males of *N.domestica* are distinct from other *Nannaria* and the nearby *N.blackmountainensis* sp. nov. based on the following combination of characters: ***Gonopods*.** Gonopodal acropodite semi-circular, gently curving dorsomedially throughout, not with abrupt 90° angle after apex as in *N.laminata* or pre-apex swelling as in *N.blackmountainensis* sp. nov. Acropodite tip with small, triangular lateral flange (Fig. 11A, red triangle), not hooked as in *N.blackmountainensis* sp. nov. and not simple, lacking flange as in *N.laminata*. Tip terminating in sharp, caudally-directed point, not blunt, rectangular as in *N.blackmountainensis* sp. nov. Height of telopodite basal zone ca. 1/4 length of acropodite, not > 1/3 length of acropodite as in *N.laminata* and ca. 1/2 length as in *N.blackmountainensis* sp. nov. Prefemoral process large, laminate and serpentine with prefemoral spine reduced to small, rectangular shelf, not fused with prefemoral process as in *N.blackmountainensis* sp. nov. ***Color*.** Tergites with orange paranotal spots (Fig. 12). Dark brown background. Dorsum of collum smooth with orange margin.

####### Measurements.

♂ (VTEC, MPE00305): BL = 28.6, CW = 3.3, IW = 1.8, ISW = 0.8, B11W = 4.2, B11H = 2.7; ♀ (VTEC, MPE00312): BL = 31.9, CW = 3.9, IW = 1.8, ISW = 0.9, B11W = 4.9, B11H = 3.5.

####### Variation.

No known variation

####### Distribution.

Known only from Blowing Rock, Watauga County, North Carolina (Suppl. material 7; Fig. 126). Distribution area: N/A; status: MRE.

####### Ecology.

Individuals of *N.domestica* have been collected from under leaf litter in mesic broadleaved forests composed of oak, maple, and rhododendron.

####### Etymology.

Shelley (1975) gives no etymology for this name, however it is reasonable to assume that the name refers to the type specimen being collected from a residence.

####### Type locality.

United States, North Carolina, Watauga County, Blowing Rock, from the yard of residence on Goforth Road, 0.5 miles north U.S. highway 321.

####### Notes.

In the original paper, Shelley (1975: 186) examined and designated the holotype male (NCSM 1007) collected by himself on October 16, 1971.

###### Nannaria komela sp. nov.

Taxon classificationAnimaliaPolydesmidaXystodesmidae

D0571FB1-4862-50FC-A14C-6998C32EA548

http://zoobank.org/DB1B3BA2-0240-4EF7-B387-AEB0C7AC62E1

**Figure 13.**
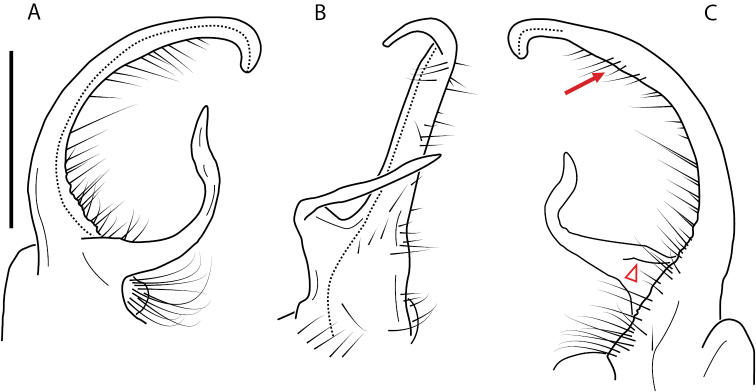
*Nannariakomela* sp. nov. paratype ♂ (VMNH, NAN0295) left gonopod **A** anterior view **B** medial view **C** posterior view; red arrow indicates dorsomedial bend giving appearance of medial swelling; red triangle indicates prefemoral spine fused with prefemoral process. Scale bar: 0.5 mm.

**Figure 14.**
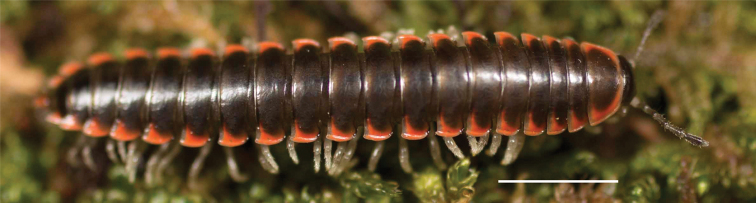
*Nannariakomela* sp. nov. holotype ♂ (VTEC, MPE03523) coloration. Scale bar: 4.0 mm.

Figs 13 , 14 Vernacular name: “The Lambsburg Twisted-Claw Millipede”

####### Material examined.

***Holotype*:** United States – **Virginia** • ♂; Carroll County, Lambsburg, Lambsburg Rd., Hawks State Forest, hillside above Turkey Creek; 36.6062°N, -80.7720°W; elev. 767 m; 7 Oct. 2017; hand collected; J. Means, D. Hennen leg.; VTEC MPE03523.

***Paratypes*:** United States – **Virginia** • 1 ♂; same collection data as holotype; VTEC MPE03536 • 1 ♂; same collection data as holotype; VMNH MPE03537 • 1 ♀; same collection data as holotype; VTEC MPE03699 • 1 ♀; same collection data as holotype; VMNH MPE03700 • 1 ♂; Carroll County, crest of Blue Ridge at end of VA. Hy. 716, ca. 3 miles W of Lambsburg; 36.5852°N, -80.8155°W; 3 June 1986; R. Hoffman, R. Highton leg.; VMNH NAN0295.

####### Other material.

United States – **North Carolina** • 1 ♀; Surry County, gully behind North Carolina Welcome Center; 36.5605°N, -80.7469°W; elev. 421 m; 24 June 2017; hand collected; J. Means leg.; VTEC MPE02891 • 1 ♂; Surry County, 8NW Dobson, 1338, 2^nd^ bridge, 0.5 S 1421; 36.4719°N, -80.8355°W; 11 Aug. 1975; R. Shelley, J. Clamp leg.; NCSM NAN0506 • 4 ♂♀; Surry County, along 1328, 1.8 mi from int. of 1325 on hillside in dry stream bed, 11.2 mi W Dobson; 36.4181°N, -80.9283°W; 7 Sep. 1978; W. Jones leg.; NCSM NAN0512; SCAU – **Virginia** • 1 ♂; Russell County, 4200’ Beartown Mtn.; 36.9360°N, -81.8859°W; 16 Oct. 1976; C. Rushin leg.; VMNH NAN0141. For detailed collection data see Suppl. material 7.

####### Diagnosis.

Adult males of *Nannariakomela* sp. nov. are distinct from other *Nannaria* and the nearby *N.wilsoni*, based on the following combination of characters: ***Gonopods*.** Gonopodal acropodite semi-circular, curving dorsomedially throughout, with appearance of slight swelling before apex in posterior view (Fig. 13C, red arrow), not with corkscrew before apex as in *N.wilsoni*. Tip simple, rounded, curving dorsally. Tip not sharp, with small triangular lateral flange as in *N.domestica*, or with laminate expansion and tooth-like lateral flange as in *N.wilsoni*. Height of telopodite basal zone reduced, ca. 1/4 length of acropodite, not ca. 1/6 as in *N.wilsoni*. Prefemoral process serpentine, curving ventrally. Prefemoral process not laminate as in *N.domestica*, or long, curving medially as in *N.wilsoni*. Prefemoral spine reduced and fused with prefemoral process, forming basal ridge (Fig. 13C, red triangle), not separate as in *N.domestica*. ***Color*.** Tergites with orange paranotal spots (Fig. 14). Black background. Dorsum of collum smooth with orange margin.

####### Measurements.

♂ holotype (VTEC, MPE03523): BL = 28.1, CW = 3.6, IW = 2.2, ISW = 0.8, B11W = 4.6, B11H = 3.0; ♀ paratype (VTEC, MPE03699): BL = 33.3, CW = 3.8, IW = 2.0, ISW = 1.0, B11W = 5.0, B11H = 3.3.

####### Variation.

The specimen from Beartown Mountain (VMNH NAN0141) has a slightly less serpentine prefemoral process, and a more pronounced prefemoral spine.

####### Distribution.

Known from southwestern Virginia and northwestern North Carolina (Virginia: Carroll and Russell counties; North Carolina: Surry County, Suppl. material 7; Fig. 126). Distribution area: 70 km^2^; status: MRE.

####### Ecology.

Individuals of *Nannariakomela* sp. nov. have been collected from mesic deciduous forests dominated by oak, maple, tuliptree, and rhododendron. They are often found under 1–2 cm moist soil on hillsides.

####### Etymology.

The specific epithet is an arbitrary combination of letters from the Greek *kome*, meaning ‘village,’ and *melon*, meaning ‘sheep,’ which refers to the town of Lambsburg, Virginia nearby to the type locality. It is to be treated as a noun in apposition.

####### Type locality.

United States, Virginia, Carroll County, Lambsburg, Lambsburg Rd., Hawks State Forest, hillside above Turkey Creek, 36.6062°N, -80.7720°W.

##### *fowleri* clade

**Components.***Nannariafowleri* Chamberlin, 1947, *N.piccolia* sp. nov., *N.simplex* Hoffman, 1949, and a female specimen from Mill Gap, VA. Members of the *fowleri* clade have simple gonopods that have a gradually curving acropodite with the tip bent at a 90° to the main branch. Hoffman labeled the specimens he collected around Longdale Mines, Virginia, as topotypes of *N.simplex*, however, after inspecting the *N.simplex* type material we do not agree that these forms are representatives of the same species. *Nannariasimplex* has a long, thin prefemoral process, while *N.piccolia* sp. nov. specimens have short, stout, claw-like prefemoral processes (Fig. 17). Unfortunately, while JCM visited the type locality of *N.simplex* (McGraw’s Gap, Virginia) three times, he was unable to recover specimens of *N.simplex* to test this relationship with molecular data (instead only finding an undescribed species in the *wilsoni* group, despite McGraw’s Gap being the type locality for both *N.simplex* and *N.ericacea*). However, due to the close geographic proximity and the shared gonopodal characters listed above, *N.simplex* is ostensibly within the *fowleri* clade.

**Distribution.** the *fowleri* clade extends from western Virginia, to eastern West Virginia, to western Maryland (The Allegheny Mountains), up through central Pennsylvania and into eastern New York (Fig. 114).

###### Nannaria fowleri

Taxon classificationAnimaliaPolydesmidaXystodesmidae

Chamberlin, 1947

32F6936F-B108-5197-AA9C-DFBED5CA3568

**Figure 15.**
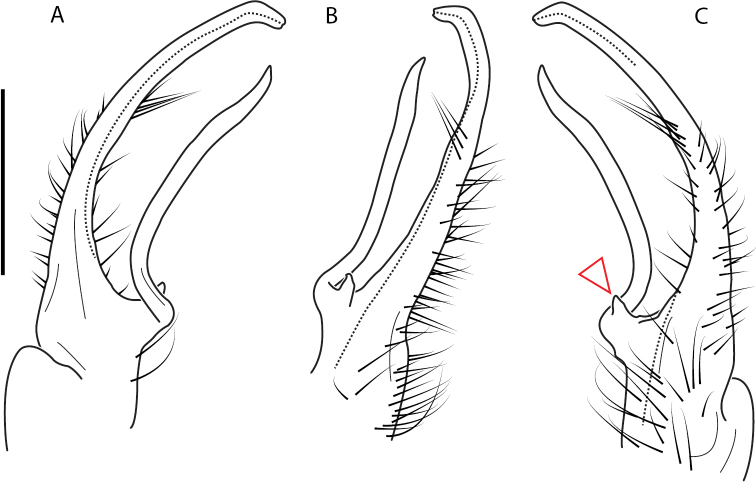
*Nannariafowleri* Chamberlin, 1947, ♂ (VTEC, MPE03017) left gonopod **A** anterior view **B** medial view **C** posterior view; red triangle indicates stout, tooth-like prefemoral spine. Scale bar: 0.5 mm.

**Figure 16.**
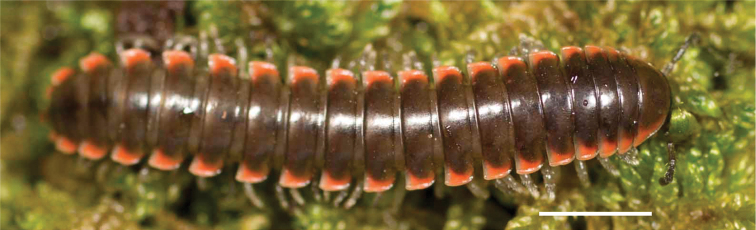
*Nannariafowleri* Chamberlin, 1947, ♂ (VTEC, MPE03017) coloration. Scale bar: 4.0 mm.

Figs 15 , 16 Vernacular name: “Fowler’s Twisted-Claw Millipede”

Nannaria fowleri Chamberlin, 1947: 29, fig. 14. Chamberlin and Hoffman 1958: 40. Shelley 1988: 1656. Hoffman 1999: 366. Shelley 2002: 1872. Marek et al. 2014: 37. Means et al. 2021: S70.
Nannaria cayugae Chamberlin, 1949: 4, fig. 3. Chamberlin and Hoffman 1958: 40. Shelley 1988: 1656.

####### Material examined.

United States – **Maryland** • 1 ♂; Allegany County, E of Flinstone, 1600 ft. summit of Town Hill Mtn.; 39.6903°N, -78.4067°W; 19 May 1958; L. Hubricht leg.; VMNH NAN0352 • 1 ♂; Allegany County, Cumberland, Knobby Mountain; 39.6527°N, -78.7626°W; 29 March; K. Holbrook; NCSM NAN0463; SCAU – **New York** • 1 ♂; Cattaraugus County, Allegany St. Pk., Anderson Trail; 42.0023°N, -78.8357°W; 10 May 1958; Muchmore leg.; VMNH NAN0347 • 1 ♂; McKean County, 8 Oct. 1959; VMNH NAN0348 • 1 ♂; Monroe County, Mendon Ponds Park; 42.9988°N, -77.5641°W; 17 Apr. 1958; Muchmore leg.; VMNH NAN0344; SCAU – **Pennsylvania** • 1 ♂; Centre County, Toftrees State Game Lands; 40.9680°N, -77.9154°W; 1993; T. McCoy leg.; NCSM NAN0451 • 3 ♂♂; Clinton County, Rauchtown, Ravensburg State Park, bank of Rauchtown Run, north of restrooms beside playfield; 41.1034°N, -77.2435°W; elev. 368 m; 18 Sep. 2017; hand collected; J. Means & D. Hennen leg.; VTEC MPE03071, 3094, 3906 • 10 ♀♀; same collection data as preceding; VTEC MPE03072, MPE03095–102, 3907 • 1 ♂; Franklin County, Caledonia State Park, Thaddeus Stevens Trail; 39.9079°N, -77.4777°W; elev. 287 m; 15 Sep. 2017; hand collected; J. Means, D. Hennen leg.; VTEC MPE03017 • 1 ♂; Franklin County, low woods, near creek 1.8 miles S of Caledonia; 39.7359°N, -77.4967°W; 8 June 1956; L. Hubricht leg.; VMNH NAN0345 • 1 ♂; Franklin County, road to Cowants Gap State Park; 39.9957°N, -77.9154°W; 11 Apr.1959; R. Mighton leg.; VMNH NAN0349 • 2 ♂♂; Potter County, Patterson Park, Susquehanna State Forest, 10 mi SSE of Coudersport, roadside logs; 41.6406°N, -77.9471°W; 20 Aug. 1971; W. Shear leg.; VMNH NAN0050 • 1 ♂; Potter County, Austin Dam ruins; 41.6944°N, -78.1197°W; 12 June; W. Shear leg.; VMNH NAN0342 • 1 ♂; Potter County, Denton Hill State Park, 14 mi. E of Coudersport, beech-birch-maple litter; 41.7685°N, -77.8334°W; 22 Aug. 1971; W. Shear leg.; VMNH NAN0037 • 1 ♂; Potter County, Rock Ridge Road, 2 mi from Lyman Run State Park, wet birch logs near spring; 41.6609°N, -77.8864°W; 23 Aug. 1971; W. Shear leg.; VMNH NAN0033; SCAU – **Virginia** • 5 ♂♂♀♀; Bath County, GWNF Long Spring Run above Little Back Creek; 38.2213°N, -79.8387°W; 24 July 1992; J. Pagels, D. Kobuszewski leg.; VMNH NAN0651 • 1 ♂; same collection data as preceding; 11 Sep. 1992; D. Kobuszewski leg.; VMNH NAN0654 • 2 ♂♂; same collection data as preceding; 17 July 1992; J. Pagels leg.; VMNH NAN0659 • 1 ♂; Bath County, “F” GWNF H’Town; 37.9993°N, -79.8315°W; 1 July 1992; J. Pagels leg.; VMNH NAN0653 • 4 ♂♂; Highland County, Locust Spring Rec. Area, 8 mi. NW of Hightown; 38.5152°N, -79.6899°W; elev. 1158 m; 23 May 1973; R. Hoffman leg.; VMNH NAN0343 • 1 ♂; same collection data as preceding; 28 Apr. 1972; R. Hoffman, Knight leg.; VMNH NAN0346 • 1 ♂; Highland County, GWNF west bank, Laurel Fork (between Bear Hollow & Newman Rd.); 38.2862°N, -79.7468°W; 17 May – 15 June 1993; VMNH NAN0658; SCAU – **West Virginia** • 2 ♀♀; Pendleton County, old road headed up to Spruce Knob; 38.6947°N, -79.5146°W; elev. 1010 m; 24 May 2015; hand collected; M. Kasson leg.; VTEC MPE00433, 446 • 1 ♂; Pendleton County, Spruce Knob; 38.7308°N, -79.4913°W; elev. 1112 m; 2016; C. Stauder leg.; VTEC MPE01816. For detailed collection data see Suppl. material 7.

####### Diagnosis.

Adult males of *N.fowleri* are distinct from other *Nannaria* and the sympatric *N.shenandoa*, based on the following combination of characters: ***Gonopods*.** Gonopodal acropodite gradually curving medially before apex, not straight as in *N.shenandoa*. Distal zone short, bent medially forming 130° angle with acropodite (Fig. 15). Telopodite with basal zone > 1/4 length of prefemoral process. Tip and distal zone simple, rectangular, blunt, < 1/8 length of acropodite, not large, curving with flange as in *N.shenandoa*. Prefemoral process arising from base of prefemoral spine, paralleling medial curve of acropodite (Fig. 15A), not directed laterally and crossing acropodite when viewed medially as in *N.shenandoa* or arising from top of prefemoral spine as in *N.tasskelsoae* sp. nov. Prefemur with stout, tooth-like prefemoral spine (Fig. 15C, red triangle). ***Color*.** Tergites with orange paranotal spots (Fig. 16). Dark brown background. Dorsum of collum smooth with orange margin. Chamberlin (1947) described the holotype as having yellow paranota, but his description was from a preserved specimen which had likely lost its color due to its preservation in alcohol.

####### Measurements.

♂ (VTEC, MPE03017): BL = 25.1, CW = 3.7, IW = 2.3, ISW = 0.8, B11W = 4.6, B11H = 2.9; ♀ (VTEC, MPE03098): BL = 28.8, CW = 3.7, IW = 2.3, ISW = 0.8, B11W = 4.4, B11H = 3.1.

####### Variation.

A male taken from Cumberland, Maryland (NCSM NAN0463), has a slight acropodite medial swelling, which has not been observed in other individuals of *N.fowleri*.

####### Distribution.

*Nannariafowleri* has a larger distribution than most *minor* species group *Nannaria*, perhaps due to post-glacial northward expansion from the eastern Appalachians (Virginia, West Virginia) north into Maryland, Pennsylvania and New York (Virginia: Bath and Highland counties; West Virginia: Pendleton and Pocahontas counties; Maryland: Allegany County; Pennsylvania: Centre, Clinton, Franklin, McKean, and Potter counties; New York: Cattaraugus and Monroe counties; Suppl. material 7; Fig. 127). Distribution area: 43,054 km^2^; status: WRE.

####### Ecology.

Individuals of *N.fowleri* have been collected from mesic hardwood forests composed of maple, hemlock, and scattered pine, often buried 1–2 cm under dark, sandy soil.

####### Etymology.

Chamberlin (1947) gave no etymology for the name *fowleri* but it is reasonable to assume that the name refers to one of the collectors of the holotype, H. W. Fowler.

####### Type locality.

United States, Maryland, Garrett County, Jennings.

####### Notes.

In the original description, Chamberlin (1947: 29) examined and designated the holotype male (ANSP Type no. 9951) collected in July 1907 by W. Stone and H. W. Fowler.

###### Nannaria piccolia sp. nov.

Taxon classificationAnimaliaPolydesmidaXystodesmidae

7885A47A-54DB-5453-BAA1-8A6C54B1AA0B

http://zoobank.org/5CF65D66-4448-4ADA-9C6E-086B1614C70B

**Figure 17.**
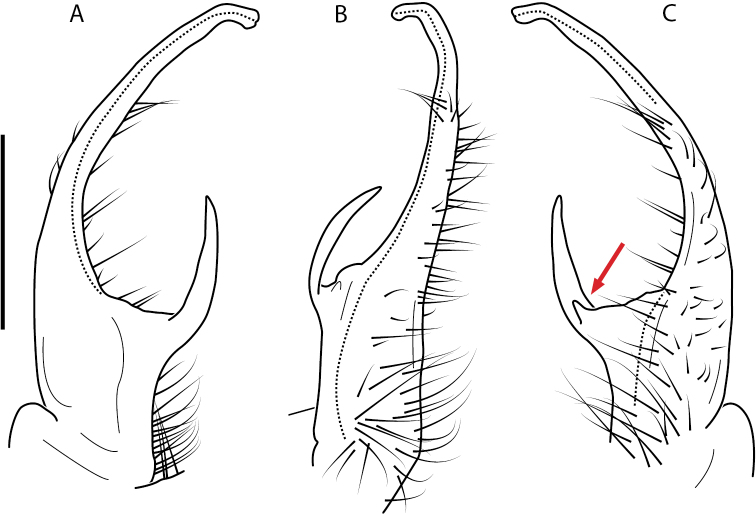
*Nannariapiccolia* sp. nov. holotype ♂ (VTEC, MPE03809) left gonopod **A** anterior view **B** medial view **C** posterior view; red arrow indicates stout, tooth-like prefemoral spine. Scale bar: 0.5 mm.

**Figure 18.**
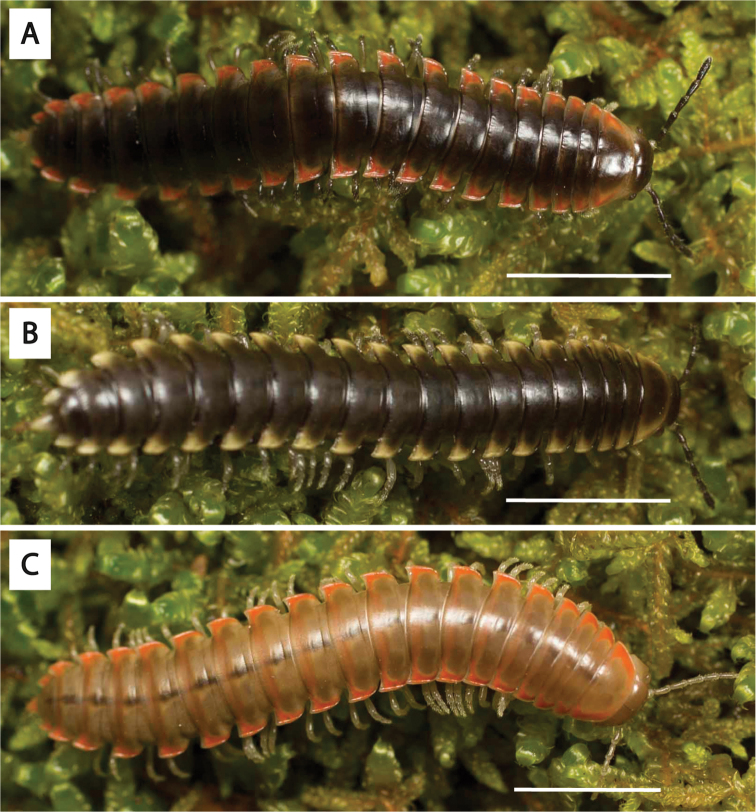
*Nannariapiccolia* sp. nov. coloration, **A** holotype ♂ (VTEC, MPE03809) orange paranotal spots **B** paratype ♂ (VTEC, MPE03812) white paranotal spots **C** paratype ♀ (VTEC, MPE03811) orange paranotal spots with orange stripes. Scale bar: 4.0 mm.

Figs 17 , 18 Vernacular name: “The Lichen-Loving Twisted-Claw Millipede”

####### Material examined.

***Holotype*:** United States – **Virginia** • ♂; Rockbridge County, 1.5 air miles NW of Collierstown, Lake Robertson Recreation Area, Mountain Trail, hillside near Hawks Creek stream crossing; 37.8065°N, -79.6152°W; elev. 457 m; 20 Feb. 2018; hand collected; J. Means, D. Hennen leg.; VTEC MPE03809.

***Paratypes*:** United States – **Virginia** • 1 ♂; same collection data as holotype; VTEC MPE03812 • 1 ♂; same collection data as holotype; VMNH MPE03816 • 2 ♀♀; same collection data as holotype; VTEC MPE03811, 17 • 1 ♀; same collection data as holotype; VMNH MPE03818.

####### Other material.

United States – **Virginia** • 1 ♂; Alleghany County, Longdale Mines; 37.8083°N, -79.6834°W; 15 Sep. 1948; R. Hoffman leg.; VMNH NAN0645 • 1 ♂; Rockbridge County, Rockbridge Alum Springs, 8 mi SW Goshen; 37.9086°N, -79.6123°W; 1 June 1970; Newman leg.; VMNH NAN0647. For detailed collection data see Suppl. material 7.

####### Diagnosis.

Adult males of *Nannariapiccolia* sp. nov. are distinct from other *Nannaria* and the sympatric *N.shenandoa*, based on the following combination of characters: ***Gonopods*.** Gonopodal acropodite gradually curving medially before apex, not straight as in *N.shenandoa*. Distal zone short, bent medially forming 130° angle with acropodite (Fig. 17). Telopodite basal zone > 1/3 length of acropodite, not < 1/3 as in *N.fowleri* and *N.shenandoa*. Tip and distal zone simple, rectangular, blunt, < 1/8 length of acropodite, not large, curving with flange as in *N.shenandoa*. Prefemoral process short, laterally curved, arising dorsomedially from prefemoral spine, not long, serpentine, paralleling curve of acropodite as in *N.fowleri*, or crossing over acropodite as in *N.shenandoa*. Space between prefemoral process and acropodite wider than telopodite basal zone, not thinner as in *N.fowleri*. Prefemur with stout, tooth-like prefemoral spine (Fig. 17C, red arrow). ***Color*.** Tergites with either orange or white paranotal spots (Fig. 18A, B) and occasionally orange paranotal spots with orange stripes (Fig. 18 C). Brown to black background. Dorsum of collum smooth with either orange or white margin, depending on color morph.

####### Measurements.

♂ holotype (VTEC, MPE03809): BL = 26.4, CW = 3.9, IW = 2.4, ISW = 0.8, B11W = 5.0, B11H = 2.9; ♀ paratype (VTEC, MPE03811): BL = 36.5, CW = 4.3, IW = 2.7, ISW = 0.9, B11W = 5.5, B11H = 4.0

####### Variation.

No known variation.

####### Distribution.

Known from a small area in western Virginia (Virginia: Allegany and Rockbridge counties, Suppl. material 7; Fig. 126). Distribution area: 35 km^2^; status: MRE.

####### Ecology.

Individuals of *Nannariapiccolia* sp. nov. have been collected from mesic deciduous forests dominated by oak, maple, and hickory, typically found under leaf litter on hillsides.

####### Etymology.

Named as a gesture of goodwill to the community of lichen specialists, in honor of the lichen species *Piccolianannaria* (Tuck.) Lendemer & Beeching. The specific epithet is a noun in apposition.

####### Type locality.

United States, Virginia, Rockbridge County, 1.5 air miles NW of Collierstown, Lake Robertson Recreation Area, Mountain Trail, hillside near Hawks Creek stream crossing, 37.8065°N, -79.6152°W.

###### Nannaria simplex

Taxon classificationAnimaliaPolydesmidaXystodesmidae

Hoffman, 1949

EA91233D-58E6-5E23-B5E3-98691AACE4CC

**Figure 19.**
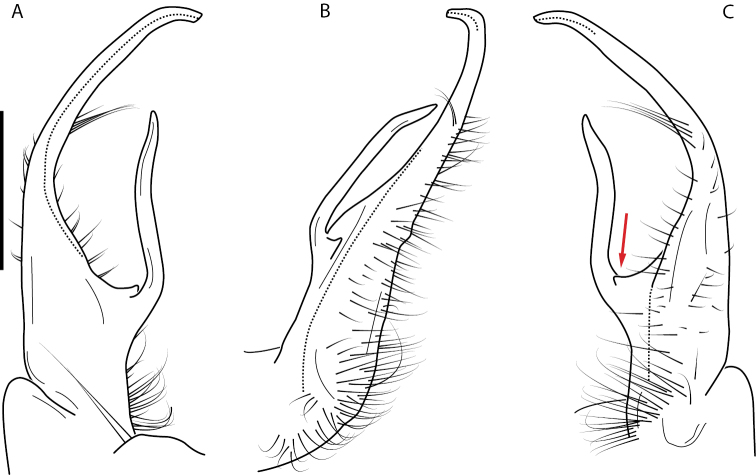
*Nannariasimplex* Hoffman, 1949, holotype ♂ left gonopod (NMNH #1807) **A** anterior view **B** medial view **C** posterior view, red arrow indicates shelf-like prefemoral spine. Scale bar: 0.5 mm.

Fig. 19 Vernacular name: “The Simple Gonopod Twisted-Claw Millipede”

Nannaria simplex Hoffman, 1949: 384, figs 13, 14. Chamberlin and Hoffman 1958: 42. Hoffman 1999: 368. Marek et al. 2014: 38. Means et al. 2021: S72.

####### Material examined.

***Holotype*:** United States – **Virginia** • ♂; Alleghany County, McGraw Gap, 3 miles northwest of Clifton Forge; [37.8586°N, -79.8660°W]; 19 June 1947; R. Hoffman leg.; NMNH #1807.

####### Other material.

United States – **Virginia** • 2 ♂♂; Augusta County, 5 mi SW of Reddish Knob, FS 85, “mature site” pitfalls; 38.4102°N, -79.3066°W; 28 May 1988; K. Buhlmann leg.; VMNH NAN0353 • 1 ♂; Bath County, Jewel Hole Hollow, off FR 141, just E of upper storage reservoir, S of Paddy Knob; 38.2427°N, -79.7955°W; elev. 914 m; 10 June 1997; MeShean Project leg.; VMNH NAN0646 • 23 ♂♀; Rockingham County, Shenandoah Mountain, DF site off Va. 924, ca. 0.5 mi E of WVA state line, jct with FS 85 (the “8 year old stand” of *Plethodonpunctatus* study); 38.4783°N, -79.2136°W; 17 June 1988; K. Buhlmann leg.; VMNH NAN0350; SCAU – **West Virginia** • 1 ♂; Pendleton County, Moyers, ca. 5 mi. SE of Franklin; 38.5154°N, -79.3621°W; 5 May 1961; D. Whitehead leg.; VMNH NAN0351. For detailed collection data see Suppl. material 7.

####### Diagnosis.

Adult males of *Nannariasimplex* are distinct from other *Nannaria* and the sympatric *wilsoni* species group, *N.ericacea*, based on the following combination of characters: ***Gonopods*.** Gonopodal acropodite gently curving medially basal to apex, not nearly straight basal to apex as in *N.ericacea*. Distal zone short, simple, at nearly 90° bend to acropodite — not serpentine, with prominent lateral flange as in *N.ericacea*. Telopodite basal zone height ca. 1/2 length of acropodite, not < 1/3 length as in *N.fowleri* and *N.ericacea*. Prefemur with thin, somewhat serpentine prefemoral process arising from medial side of prefemoral spine, directed caudally—not paralleling acropodite as in *N.fowleri*, or large, crossing under acropodite distal zone as in *N.ericacea*. Prefemoral spine shelf-like (Fig. 19C, red arrow)—not pronounced and tooth-like as in *N.fowleri*, or lacking as in *N.ericacea*. ***Color*.**Hoffman (1949) described *N.simplex* as having “reddish pink” tergites with a black background. Collum completely black.

####### Measurements.

♂ holotype (NMNH, #1807): BL = 29.4, CW = 3.9, IW = 2.6, ISW = 0.9, B11W = 4.5, B11H = 3.2.

####### Variation.

No known variation.

####### Distribution.

Known from the border of Virginia and West Virginia near the Shenandoah Mountains (Virginia: Alleghany, Augusta, Bath, and Rockingham counties; West Virginia: Pendleton County; Suppl. material 7; Fig. 127). Distribution area: 1,213 km^2^; status: SRE.

####### Ecology.

Hoffman (1949) provides little ecological information, other than mentioning that the type specimen was found under hemlock bark. The authors JCM and DAH have visited the type locality, McGraw Gap, on multiple occasions and the habitat is composed of a mesic mixed hardwood and pine forest dominated by oak, maple, tuliptree, rhododendron, pine and hemlock, with an extensive fern understory.

####### Etymology.

Hoffman (1949) gave no etymology for his choice of specific name but it is reasonable to assume that the name refers to the relatively simple form of the gonopods.

####### Type material.

United States, Virginia, Alleghany County, McGraw Gap, 3 miles northwest of Clifton Forge; [37.8586°N, -79.8660°W].

####### Notes.

In the original description, Hoffman (1949: 385) examined and designated the holotype male (NMNH #1807) collected on June 19, 1947 by R. Hoffman.

##### *paupertas* clade

**Components.** – *Nannariacastra* sp. nov., *N.paupertas* sp. nov., a female from Raleigh, North Carolina and a female from Carroll County, Virginia. Members of the *paupertas* clade share a medially curved prefemoral process and caudally directed acropodite tip. The division of *N.castra* sp. nov. and *N.sheari* sp. nov. (from Camp Creek Falls State Park and Brush Creek Preserve, respectively) into two separate clades was highly unexpected. Both specimens have medially curved prefemoral processes, with caudally directed acropodite tips, and the Camp Creek specimen does not share a sinuous region with *N.paupertas* sp. nov., a feature which we expected to unite *N.paupertas* sp. nov. with *N.cingulata* sp. nov. Furthermore, *N.castra* sp. nov. and *N.sheari* sp. nov. specimens were collected from localities only ~ 8 km apart. However, gonopods of *N.paupertas* sp. nov. and *N.castra* sp. nov. do share some morphological characteristics (see above) and therefore the similarities between the *N.castra* sp. nov. and *N.sheari* sp. nov. specimens are likely due to convergence. The development of a sinuous region in *N.paupertas* sp. nov. and a cingulum in *N.cingulata* sp. nov. is also likely attributable to convergence. The female from Raleigh, North Carolina may be *N.conservata*, as that is the only known species from that area. However, without a male, and in light of the complex nature of clade distributions within the *minor* group, it is possible that this female specimen represents an additional undescribed species. Collection of a male of this species, as well as the species from Carroll County, Virginia, will help resolve relationships within this clade.

**Distribution.** – the *paupertas* clade extends from central North Carolina, through southwestern Virginia and southern West Virginia (Fig. 114).

###### Nannaria castra sp. nov.

Taxon classificationAnimaliaPolydesmidaXystodesmidae

3B89A684-B23B-591D-8A69-300330109B40

http://zoobank.org/A35BF7A1-2D72-4670-AE27-4276CD168523

**Figure 20.**
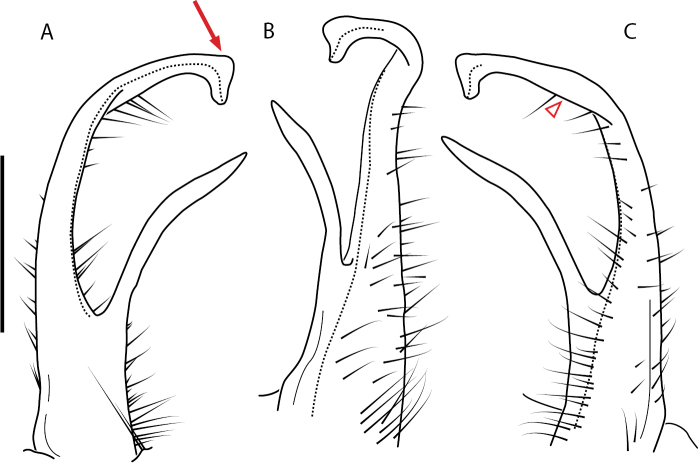
*Nannariacastra* sp. nov. holotype ♂ (VTEC, MPE03470) left gonopod **A** anterior view; red arrow indicates lateral flange **B** medial view **C** posterior view; red triangle indicates acropodite midpoint twist and swelling. Scale bar: 0.5 mm.

**Figure 21.**
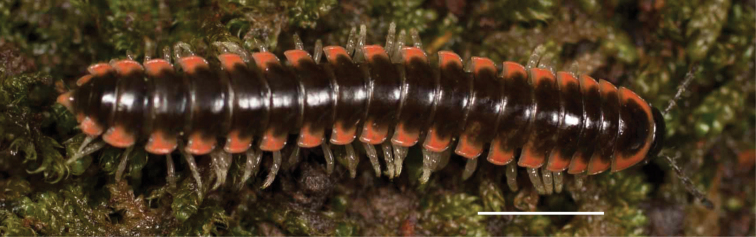
*Nannariacastra* sp. nov. holotype ♂ (VTEC, MPE03470) coloration. Scale bar: 4.0 mm.

Figs 20 , 21 Vernacular name: “The Camp Creek Twisted-Claw Millipede”

####### Material examined.

***Holotype*:** United States – **West Virginia** • ♂; Mercer County, Camp Creek State Park; 37.5147°N, -81.1297°W; elev. 616 m; 12 Nov. 2017; hand collected; J. Means leg.; VTEC MPE03470.

***Paratypes*:** United States – **West Virginia** • 2 ♀♀; same collection data as holotype; VTEC MPE03476, 3482 • 3 ♂♂; same collection data as holotype; VTEC MPE03475, 3477, 3478 • 3 ♂♂; same collection data as holotype; VMNH MPE03480, 81, 83 • 1 ♀; same collection data as holotype; VMNH MPE03479.

####### Other material.

United States – **West Virginia** • 2 ♀♀; Mercer County, Camp Creek State Park, Farley Branch; 37.5082°N, -81.1370°W; elev. 657 m; 21 July 2005; hand collected; P. Marek, C. Spruill leg.; VTEC SPC000749, 750 • 1 ♂; Mercer County, Camp Creek State Park, Nash Fork Hollow; 37.5233°N, -81.1311°W; 36 Mar. 1968; W. Shear leg.; VMNH NAN0014. For detailed collection data see Suppl. material 7.

####### Diagnosis.

Adult males of *Nannariacastra* sp. nov. are distinct from other *Nannaria* and the nearby *N.aenigma* Means, Hennen & Marek in Means et al. 2021, based on the following combination of characters: ***Gonopods*.** Gonopodal acropodite relatively straight before curving medially at half-way point, not continually curving as in *N.paupertas* sp. nov. Distal zone curving dorsally, with tip directed caudally (Fig. 20B)—not curving ventromedially with tip directed dorsomedially as in *N.paupertas* sp. nov., or tip directed medially as in *N.aenigma*. Acropodite tip with lateral flange (Fig. 20A, red arrow). Acropodite with slight twist and swelling at midpoint (Fig. 20C, red triangle), lacking medial flange of *N.paupertas* sp. nov., or lateral flange of *N.aenigma*. Telopodite basal zone thin, with slight lateral bulge, height subequal to 1/2 length of acropodite, not wide as in *N.paupertas* sp. nov., or > 1/6 length as in *N.aenigma*. Prefemur with dorsomedially curving prefemoral process (Fig. 20B), not ventromedially curving as in *N.paupertas* sp. nov., or straight as in *N.aenigma*. Prefemoral spine absent, not prominent, acicular as in *N.paupertas* sp. nov. ***Color*.** Tergites with orange paranotal spots (Fig. 21). Black background. Dorsum of collum smooth with orange margin.

####### Measurements.

♂ holotype (VTEC, MPE03470): BL = 28.5, CW = 3.6, IW = 1.8, ISW = 0.7, B11W = 4.2, B11H = 2.5. ♀ paratype (VTEC, MPE03476): BL = 27.8, CW = 3.6, IW = 2.0, ISW = 0.9, B11W = 4.5, B11H = 3.2.

####### Variation.

No known variation.

####### Distribution.

Known only from the type locality, Camp Creek State Park (West Virginia: Mercer County; Suppl. material 7; Fig. 126). Distribution area: > 1 km^2^; status: MRE.

####### Ecology.

Individuals of *N.castra* sp. nov. were found in a mesic hardwood and rhododendron forest on top of hardpacked soil surrounding the entrance parking lot to Camp Creek State Park, and along the road leading towards the park campground. The majority of individuals were walking on top of the soil beneath leaf litter.

####### Etymology.

This species is named for its type locality. The specific name a noun in apposition from the Latin *castra* for ‘camp.’

####### Type locality.

United States, West Virginia, Mercer County, Camp Creek State Park, 37.5147°N, -81.1297°W.

###### Nannaria paupertas sp. nov.

Taxon classificationAnimaliaPolydesmidaXystodesmidae

B6B89965-FD2F-5B99-B1E2-44B94C237BB4

http://zoobank.org/C8B741BA-38E7-4472-9364-3A0F4EB52DAB

**Figure 22.**
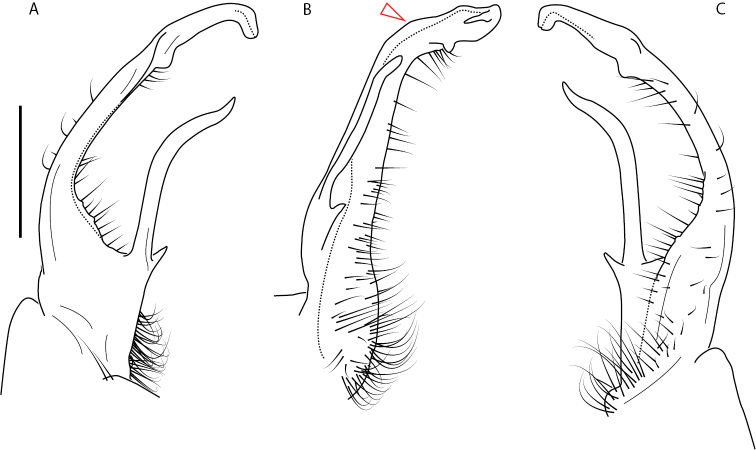
*Nannariapaupertas* sp. nov. holotype ♂ (VTEC, MPE00108) left gonopod **A** anterior view **B** medial view; red triangle indicates acropodite ventromedial bend **C** posterior view. Scale bar: 0.5 mm.

**Figure 23.**
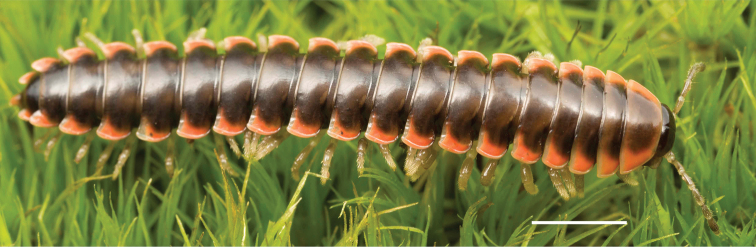
*Nannariapaupertas* sp. nov. holotype ♂ (VTEC, MPE00108) coloration. Scale bar: 4.0 mm.

Figs 22 , 23 Vernacular name: “The Poverty Creek Twisted-Claw Millipede”

####### Material examined.

***Holotype*:** United States – **Virginia** • ♂; Montgomery County, Blacksburg, Pandapas Pond, Poverty Creek Trail near forest access rd. 708; 37.2678°N, -80.4852°W; elev. 648 m; 7 July 2014; hand collected; J. Means leg.; VTEC MPE00108.

***Paratype*:** United States – **Virginia** • 1 ♀; same collection data as holotype; VMNH MPE00109.

####### Other material.

United States – **Virginia** • 1 ♂; Montgomery County, Pandapas Pond, NW of Blacksburg, Poverty Creek Trail; 37.2805°N, -80.4720°W; elev. 651 m; hand collected; D. Hennen leg.; VTEC MPE02084 • 1 ♂; Montgomery County, gully on north slope of Brush Mtn. off forest service road 208; 37.2764°N, -80.4837°W; elev. 693 m; 9 May 2015; J. Means leg.; VTEC MPE00359 • 1 ♂; Montgomery County, Pandapas Pond, in bottomland below horse trail parking lot; 37.2824°N, -80.4485°W; elev. 738 m; hand collected; J. Means leg.; VTEC MPE00834 • 1 ♀; same collection data as preceding; VTEC MPE00836. For detailed collection data see Suppl. material 7.

####### Diagnosis.

Adult males of *Nannariapaupertas* sp. nov. are distinct from other *Nannaria* and the sympatric *N.ericacea*, based on the following combination of characters: ***Gonopods*.** Gonopodal acropodite gently curving medially before bending ventromedially at a nearly 90° angle (Fig. 22B, red triangle), not straight as in *N.ericacea* or curving dorsomedially as in *N.cingulata* sp. nov. Distal zone short, bent dorsomedially at 90° angle, not laminate, curving medially with flanges as in *N.ericacea* or bent dorsomedially at 45° as in *N.cingulata* sp. nov. Acropodite tip directed dorsomedially, without flanges, not laminate and serpentine, directed medially as in *N.ericacea*. Telopodite basal zone height > 1/3 length of acropodite, not greatly reduced as in *N.ericacea* or with lateral bulge as in *N.cingulata* sp. nov. Acropodite shaft swollen before apex, with medial flange, and sinuous region, not with lateral flange as in *N.ericacea* or cingulum as in *N.cingulata* sp. nov. Prefemur with prefemoral process curving ventromedially, not ventrally as in *N.cingulata* sp. nov. and *N.ericacea*. Prefemoral process (when viewed medially) coplanar with acropodite, not crossing acropodite as in *N.ericacea*. Prefemoral process 2/3 length of acropodite, not subequal as in *N.ericacea* or 1/2 length as in *N.cingulata* sp. nov. Prefemoral spine prominent, acicular, not reduced as in *N.cingulata* sp. nov. or absent as in *N.ericacea*. ***Color*.** Tergites with orange paranotal spots (Fig. 23). Dark brown background. Dorsum of collum smooth with orange margin.

####### Measurements.

♂ holotype (VTEC, MPE00108): BL = 30.3, CW = 4.1, IW = 2.1, ISW = 0.9, B11W = 4.8, B11H = 3.1; ♀ paratype (VMNH, MPE00109): BL = 31.7, CW = 4.0, IW = 2.2, ISW = 1.0, B11W = 5.1, B11H = 3.6.

####### Variation.

A dead male collected from Craig County is morphologically similar to *N.paupertas* sp. nov., with the exception of a prefemoral process which is straight for most of its length before bending nearly 90° ventrally, and a highly reduced prefemoral spine. Genetic sequences of a sympatric female did not support conspecificity with *N.paupertas* sp. nov. (Suppl. material 7), and therefore the Craig County population may represent a separate species and is not included in *N.paupertas* sp. nov.

####### Distribution.

Known only from a small area around Pandapas Pond (an impoundment of Poverty Creek) in Montgomery County, Virginia (Virginia: Montgomery County, Suppl. material 7; Fig. 126). Distribution area: 2 km^2^; status: MRE.

####### Ecology.

Individuals of *N.paupertas* sp. nov. have been collected from the side of walking trails in mesic, broadleaved forests, composed of oak, maple, ferns, and scattered pine, under deciduous leaf litter adjacent to rhododendron groves.

####### Etymology.

This species is named for the Poverty Creek Trail by which it was first collected. The specific name is derived from the Latin *paupertas*, meaning poverty, and is a noun in apposition.

####### Type locality.

United States, Virginia, Montgomery County, Blacksburg, Pandapas Pond, Poverty Creek Trail near forest access rd. 708, 37.2678°N, -80.4852°W.

##### Murphy clade

**Components.** female specimens from Mattaponi Wildlife Management Area, Virginia, the Appalachian Trail crossing of Virginia highway 621, and Dr. Brian Murphy’s property near Blacksburg, Virginia. This clade is unfortunately completely comprised of female specimens and therefore little in the way of conclusions can be drawn as to its characteristics. The specimens from Dr. Brian Murphy’s property and the AT crossing of Virginia highway 621 are likely representatives of the same undescribed species, while the specimen from Mattaponi Wildlife Management Area may represent a second undescribed species.

##### *mcelroyorum* clade

**Components.***Nannariacaverna* sp. nov. and *N.mcelroyorum* sp. nov. The *mcelroyorum* clade is characterized by a simple, medially directed acropodite tip, and a prefemoral process arising dorsomedially from the prefemoral spine. We had expected *N.mcelroyorum* sp. nov. and *N.caverna* sp. nov. to fall into separate clades based on the differing prefemoral processes. However, the form of the prefemoral process varies between species in several clades recovered in the molecular phylogeny (Fig. 7), suggesting that the prefemoral process may not be as taxonomically informative as we had previously believed.

**Distribution.** – the *mcelroyorum* clade extends from southwestern West Virginia into northeastern Kentucky (Fig. 114).

###### Nannaria caverna sp. nov.

Taxon classificationAnimaliaPolydesmidaXystodesmidae

9D604702-6D95-5AD5-865D-B4B414B09236

http://zoobank.org/41468A49-456A-41C0-B973-D66E8B757919

**Figure 24.**
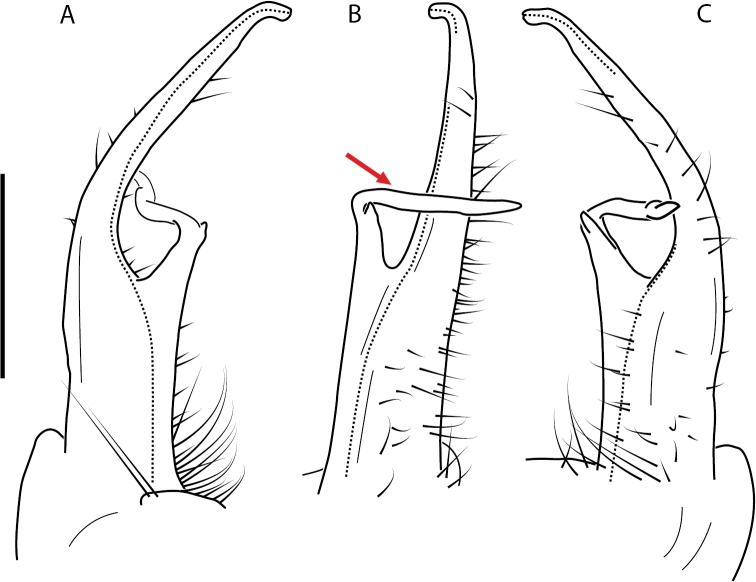
*Nannariacaverna* sp. nov. holotype ♂ (VTEC, MPE03139) left gonopod **A** anterior view **B** medial view; red arrow indicates ventrally directed prefemoral process **C** posterior view. Scale bar: 0.5 mm.

**Figure 25.**
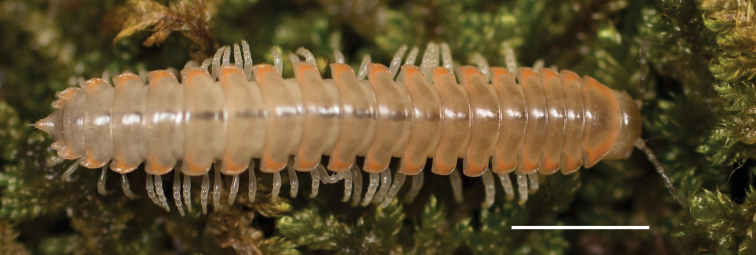
*Nannariacaverna* sp. nov. holotype ♂ (VTEC, MPE03139) coloration. Scale bar: 4.0 mm.

Figs 24 , 25 Vernacular name: “The Carter Caves Twisted-Claw Millipede”

####### Material examined.

***Holotype*:** United States – **Kentucky** • ♂; Carter Co., Carter Caves State Park; 38.3738°N, -83.1142°W; 24 Sep. 2017; hand collected; J. Means, D. Hennen leg.; VTEC MPE03139.

***Paratypes*:** United States – **Kentucky** • 1 ♂; same collection data as holotype; VTEC MPE03157 • 1 ♀; same collection data as holotype; VTEC MPE03158 • 1 ♂; same collection data as holotype; VMNH MPE03160 • 1 ♀; same collection data as holotye; VMNH MPE03159. For detailed collection data see Suppl. material 7.

####### Diagnosis.

Adult males of *Nannariacaverna* sp. nov. are distinct from other *Nannaria* and nearby *N.shenandoa*, based on the following combination of characters: ***Gonopods*.** Gonopodal acropodite linear, bent medially forming 130° angle with telopodite basal zone bent medially at tip, not continually curving throughout as *N.serpens* sp. nov. and *N.shenandoa*. Tip simple, rectangular, without laminate flanges as in *N.shenandoa*. Telopodite basal zone tall, > 1/2 length of acropodite, not < 1/2 as in *N.serpens* sp. nov. and *N.shenandoa*. Prefemur with sinuous, ventrally directed prefemoral process, bent at a 90° angle, arising from large, pronounced prefemoral spine (Fig. 24B, red arrow), not directed cephalically as in *N.serpens* sp. nov., or curving laterally as in *N.shenandoa*. ***Color*.** Tergites with light orange paranotal spots (Fig. 25). Light grey background, likely due to teneral condition of specimens. Collum smooth with orange margin.

####### Measurements.

♂ holotype (VTEC, MPE03139): BL = 24.7, CW = 3.8, IW = 2.5, ISW = 0.8, B11W = 4.2, B11H = 2.9. ♀ paratype (VMNH, MPE03159): BL = 25.7, CW = 3.2, IW = 2.2, ISW = 1.2, B11W = 4.0, B11H = 2.9.

####### Variation.

No known variation.

####### Distribution.

Known only from the type locality (Kentucky: Carter Caves State Park; Suppl. material 7; Fig. 127). Distribution area: N/A; status: MRE.

####### Ecology.

Individuals of *N.caverna* sp. nov. were collected as they emerged from hardpacked soil on the edge of a creek running through Carter Caves State Park. The surrounding forest was made up of hardwood trees and rhododendron thickets.

####### Etymology.

This species is named for its type locality. The specific name is a noun in apposition from the Latin *caverna* for cave.

####### Type locality.

United States, Kentucky, Carter Co., Carter Caves State Park, 38.3738°N, -83.1142°W.

###### Nannaria mcelroyorum sp. nov.

Taxon classificationAnimaliaPolydesmidaXystodesmidae

772940C1-8E93-5467-A734-20AD0C3F704E

http://zoobank.org/37EFC360-6A08-41DD-848F-2C5C97C60E70

**Figure 26.**
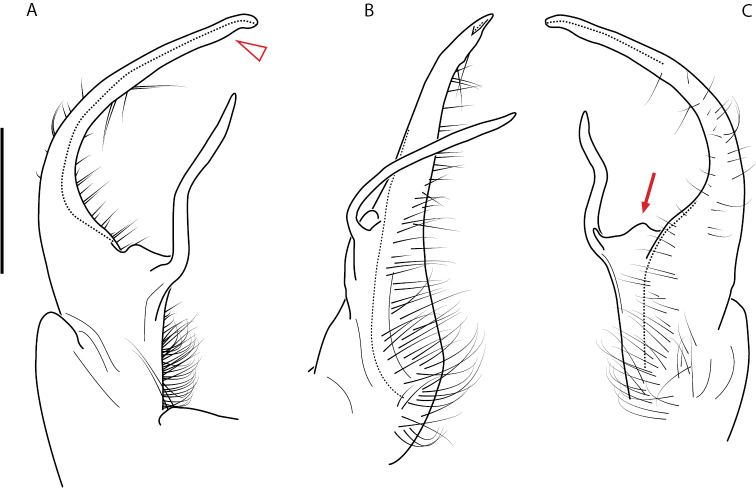
*Nannariamcelroyorum* sp. nov. holotype ♂ (VTEC, MPE03113) left gonopod **A** anterior view; red triangle indicates medial flange **B** medial view **C** posterior view; red arrow indicates prefemoral spine secondary hump. Scale bar: 0.5 mm.

**Figure 27.**
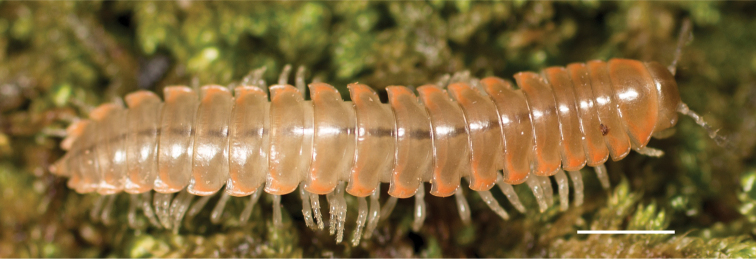
*Nannariamcelroyorum* sp. nov. non-type ♂ (VTEC, MPE03125) coloration. Scale bar: 4.0 mm.

Figs 26 , 27 Vernacular name: “The McElroy Twisted-Claw Millipede”

####### Material examined.

***Holotype*:** United States – **West Virginia** • ♂; Wayne County, Barboursville, Beech Fork State Park, Lost Trail near Moxley Branch Campground; 38.3047°N, -82.3512°W; elev. 194 m; 23 Sep. 2017; hand collected; J. Means and D. Hennen leg.; VTEC MPE03113.

***Paratypes*:** United States – **West Virginia** • 3 ♂♂; same collection data as holotype; VTEC MPE03116, 3118, 3120 • 4 ♂♂; same collection data as holotype; VMNH MPE03135, 3151, 3154, 3156 • 3 ♀♀; same collection data as holotype; VTEC MPE03117, 3119, 3121 • 4 ♀♀; same collection data as holotype; VMNH MPE03122, 3136, 3137, 3155.

####### Other material.

United States – **Kentucky** • 1 ♂; Boyd County, Fannin Park, off Bramble Drive, hillside across road from park; 38.3475°N, -82.6866°W; elev. 219 m; 24 Sep. 2017; J. Means, D. Hennen leg.; VTEC MPE03125; SCAU – **West Virginia** • 3 ♂♂; Boone County, Julian, along Big Pinnacle Road; 38.1802°N, -81.8384°W; elev. 223 m; 23 Sep. 2017; hand collected; J. Means, D. Hennen leg.; VTEC MPE03262, 63, 3289 • 2 ♀♀; same collection data as preceding; VTEC MPE03290, 91 • 7 ♂♂; same collection data as preceding; VTEC MPE02240, 2257, 2258, 2260–62, 3727 • same collection data as preceding; 5 ♀♀; same collection data as preceding;18 Nov. 2016; VTEC MPE02248–50, 3728, 3729. For detailed collection data see Suppl. material 7.

####### Diagnosis.

Adult males of *Nannariamcelroyorum* sp. nov. are distinct from other *Nannaria* and the nearby *N.shenandoa*, based on the following combination of characters: ***Gonopods*.** Gonopodal acropodite gently curving medially throughout, not straight as in *N.shenandoa*. Acropodite gradually tapering towards tip, with small lobed medial flange (Fig. 26A, red triangle), lacking swollen medial area as in *N.serpens* sp. nov. Tip directed medially, rounded and simple, not with folds, grooves, flanges as in *N.shenandoa*. Distal zone greatly reduced, not enlarged and curving caudolaterally as in *N.shenandoa*. Prefemur with thin, sinuous, acicular prefemoral process, not curved, saber-like as in *N.shenandoa*. Prefemoral spine small, medially directed, with secondary hump proximal to acropodite base (Fig. 26C, red arrow), not enlarged, cephalically directed as in *N.serpens* sp. nov., or lacking as in *N.shenandoa*. Telopodite basal zone height ca. 1/2 length of acropodite, not > 1/2 length of acropodite as in *N.serpens* sp. nov. or ca. 1/5 length as in *N.shenandoa*. ***Color*.** Tergites with light orange paranotal spots and faint orange stripes (Fig. 27). Light brown background. Dorsum of collum smooth with orange margin.

####### Measurements.

♂ paratype (VTEC, MPE03116; holotype too damaged for measurement): BL = 33.9, CW = 4.7, IW = 2.3, ISW = 0.9, B11W = 5.4, B11H = 3.6; ♀ paratype (VTEC, MPE03117): BL = 33.2, CW = 4.4, IW = 2.5, ISW = 1.3, B11W = 5.9, B11H = 4.3.

####### Variation.

Individuals of *N.mcelroyorum* sp. nov. from the Boone County, West Virginia site have prefemoral processes ca. 2/3 the length of those of the holotype.

####### Distribution.

Known from a linear area in western West Virginia and Eastern Kentucky (Kentucky: Boyd County; West Virginia: Boone and Wayne counties, Suppl. material 7; Fig. 127). Distribution area: 105 km^2^; status: MRE.

####### Ecology.

Individuals of *N.mcelroyorum* sp. nov. were collected from mesic deciduous forests dominated by oak, maple, laurel, and some pine, often under 1–2 cm soil.

####### Etymology.

This species is named for Clint, Justin, Travis, and Griffin McElroy from Huntington, West Virginia, who provided endless hours of emotional support to JCM and DAH during field collections through their podcasts, ‘The Adventure Zone’ and ‘My Brother, My Brother, and Me’. The specific name is a plural genitive derived as a patronym.

####### Type locality.

United States, West Virginia, Wayne County, Barboursville, Beech Fork State Park, Lost Trail near Moxley Branch Campground, 38.3047°N, -82.3512°W.

##### *hardeni* clade

**Components.** – *Nannariahardeni* sp. nov. Based on the molecular phylogeny (Fig. 7) and the combination of morphological characters detailed in the below diagnosis, *N.hardeni* sp. nov. has no sister taxon and is in a monotypic clade (Fig. 113). Due to shared morphological characters between *N.hardeni* sp. nov. and *N.monsdomia* sp. nov., including a large, rounded prefemoral spine, the proximity of the prefemoral process to the acropodite and the wide separation between the prefemoral spine and process, we had expected the two species to be closely related. Instead, *N.hardeni* sp. nov. is sister to the *serpens* and *castanea* clades, and appears to be yet another example of morphological convergence confusing attempts to infer evolutionary relatedness through gonopod morphology alone.

**Distribution.***N.hardeni* sp. nov. is known only from southern Virginia (Fig. 128).

###### Nannaria hardeni sp. nov.

Taxon classificationAnimaliaPolydesmidaXystodesmidae

B4B26CD2-BA77-5CD9-A431-C54E1D970B76

http://zoobank.org/A276BC4F-2CB8-4822-9A23-5A368B6CE830

**Figure 28.**
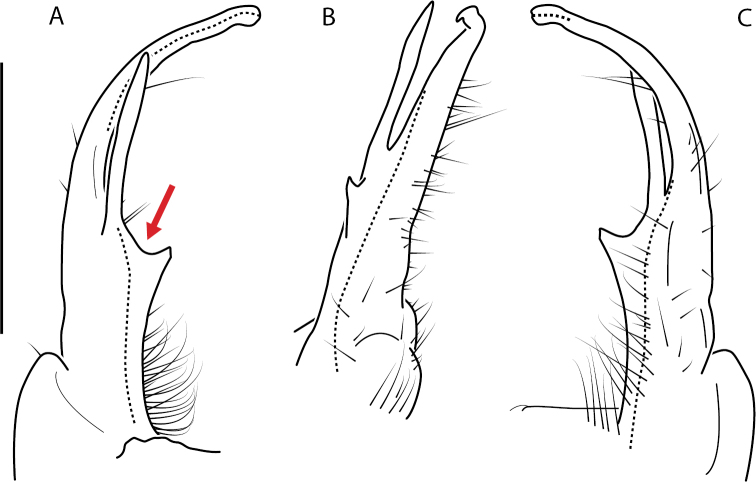
*Nannariahardeni* sp. nov. holotype ♂ (VMNH, MPE02278) left gonopod **A** anterior view; red arrow indicates wide separation between prefemoral process and prefemoral spine **B** medial view **C** posterior view. Scale bar: 0.5 mm.

**Figure 29.**
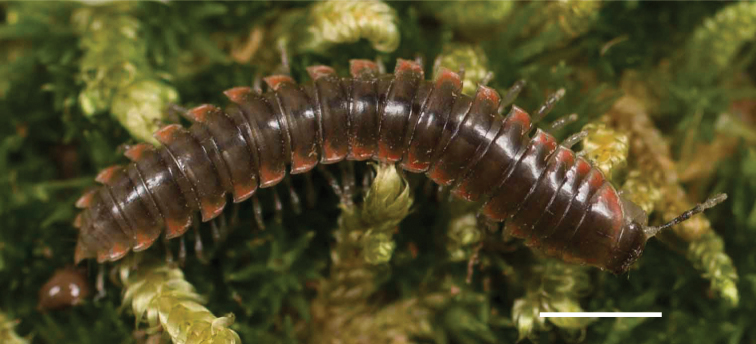
*Nannariahardeni* sp. nov. holotype ♂ (VMNH, MPE02278) coloration. Scale bar: 4.0 mm.

Figs 28 , 29 Vernacular name: “Curt Harden’s Twisted-Claw Millipede”

####### Material examined.

***Holotype*:** United States – **Virginia** • ♂; Pittsylvania County, Angler’s Park; 36.5577°N, -79.3515°W; elev. 134 m; 23 Dec. 2016; C. W. Harden leg.; VMNH MPE02278.

***Paratypes*:** United States – **Virginia** • 1 ♂; same collection data as holotype; VTEC MPE02282 • 1 ♂; same collection data as holotype; VMNH MPE02283 • 1 ♀; same collection data as holotype; VTEC MPE02279 • 1 ♀; same collection data as holotype; VMNH MPE02281.

####### Other material.

United States – **Virginia** • 1 ♂; Henry County, vicinity of Figsboro; 36.7861°N, -79.8586°W; 9 Nov. 1980; R. Hoffman leg.; VMNH NAN0247 • 1 ♂; same collection data as preceding; 15 Nov. 1981; VMNH NAN0249 • 1 ♂; Henry County, Martinsville, VMNH; 36.6761°N, -79.8771°W; 2 Nov. 1992; J. Anderson, VMNH surveys leg.; VMNH NAN0270 • 1 ♂; same collection data as preceding; Dec. 1997; VMNH NAN0271 • 1 ♂; same collection data as preceding; 19 Dec. 1996; VMNH survey leg.; VMNH NAN0273 • 1 ♂; Martinsville, VMNH on sidewalk to Annex; 36.6761°N, -79.8771°W; 12 Nov. 1992; J. Anderson leg.; VMNH NAN0272 • 1 ♂; Martinsville, Dundee Lane; 36.6892°N, -79.8727°W; 5 Jan. 1990; P. Carter leg.; VMNH NAN0274 • 1 ♂; near Martinsville, Dupont prop.; 36.6680°N, -79.8922°W; 16 Oct. 1995; J. Anderson leg.; VMNH NAN0275 • 2 ♂♂; same collection data as preceding; 23 Oct. 1995; VMNH NAN0276 • 8 ♂♂; Pittsylvania County, Danville, Angler’s Park; 36.5500°N, -79.3500°W; elev. 126 m; 29 Oct. 2017; hand collected; C. Harden leg.; VTEC MPE03734–40, 4239 • 3 ♀♀; same collection data as preceding; VTEC MPE03741–3 • 18 ♂♂; Pittsylvania County, Lacy Farm DF site, 3 mi. ENE of Axton; 36.6760°N, -79.6621°W; 29 Mar. 1992; VMNH survey leg.; VMNH NAN0245 • 8 ♂♂; same collection data as preceding; 21 Dec. 1992; VMNH NAN0246 • 10 ♂♂; same collection data as preceding; 23 Apr. 1992; VMNH NAN0248 • 2 ♂♂; same collection data as preceding; 15 May 1992; VMNH NAN0250 • 8 ♂♂; Lacy Farm, ca 5 mi. ENE of Axton, pitfall trap; 36.6870°N, -79.6286°W; 13 May 1992; VMNH survey leg.; VMNH NAN0251; 28 ♂♂; Lacy Farm DF site, ca 4 mi. ENE of Axton; 36.6815°N, -79.6454°W; 13 Nov. 1992; VMNH survey leg.; VMNH NAN0252. For detailed collection data see Suppl. material 7.

####### Diagnosis.

Adult males of *Nannariahardeni* sp. nov. are distinct from other *Nannaria* and the nearby *N.wilsoni*, based on the following combination of characters: ***Gonopods*.** Gonopodal acropodite (Fig. 28) gently curving medially before apex, distal zone reduced, not laminate, curving caudally with flanges as in *N.wilsoni* or with lobed lateral flange as in *N.monsdomia* sp. nov. Telopodite basal zone height > 1/2 length of acropodite, not < 1/4 length of acropodite as in *N.wilsoni*. Tip simple, directed medially, without lateral flange as in *N.monsdomia* sp. nov., and not directed caudally with lateral flange as in *N.wilsoni*. Prefemur with straight, acicular prefemoral process, not laminate, curving medially as in *N.monsdomia* sp. nov. or *N.wilsoni*. Prefemoral process not crossing acropodite as in *N.wilsoni* or *N.monsdomia* sp. nov. Prefemoral process length subequal to height of telopodite basal zone, not greater than height of telopodite basal zone as in *N.monsdomia* sp. nov. and *N.wilsoni*. Prefemoral spine widely separated from prefemoral process (Fig. 28A, red arrow) and sharp, tooth-like, not rounded as in *N.monsdomia* sp. nov. or lacking as in *N.wilsoni*. ***Color*.** Tergites with orange paranotal spots (Fig. 29). Dark brown background. Dorsum of collum smooth with orange margin.

####### Measurements.

♂ holotype (VMNH, MPE02278): BL = 17.3, CW = 2.5, IW = 1.6, ISW = 0.6, B11W = 2.9, B11H = 1.9; ♀ paratype (VMNH, MPE02281): BL = 17.6, CW = 2.1, IW = 1.8, ISW = 0.6, B11W = 2.9, B11H = 1.9.

####### Variation.

No known variation.

####### Distribution.

Known from southern Virginia, near the North Carolina border (Virginia: Henry and Pittsylvania counties; Suppl. material 7; Fig. 128). Distribution area: 374 km^2^; status: MRE.

####### Ecology.

Individuals of *N.hardeni* sp. nov. have been found in young, disturbed mesic hardwood forests under rocks, often in fern thickets. Interestingly, *N.hardeni* sp. nov. has also been collected from IPM sticky traps from inside the Virginia Museum of Natural History in Martinsville, Virginia.

####### Etymology.

This species is named after its collector, Curt W. Harden. The specific name is a genitive noun derived as a patronym.

####### Type locality.

United States, Virginia, Pittsylvania County, Angler’s Park, 36.5577°N, -79.3515°W.

##### *serpens* clade

**Components.** – *Nannariaserpens* sp. nov. Based on the molecular phylogeny (Fig. 7) and the morphological characters detailed in the below diagnosis, including the presence of a serpentine prefemoral process and enlarged prefemoral spine, coupled with a slight medial swelling before the acropodite apex, *N.serpens* sp. nov. is in a monotypic clade (Fig. 113).

**Distribution.***N.serpens* sp. nov. is known solely from southwestern Virginia and northwestern North Carolina (Fig. 126).

###### Nannaria serpens sp. nov.

Taxon classificationAnimaliaPolydesmidaXystodesmidae

B84A3715-F652-5724-8ABB-1D27CEDBB7BD

http://zoobank.org/33C19D59-CF22-4713-A660-D1A2FC2DE146

**Figure 30.**
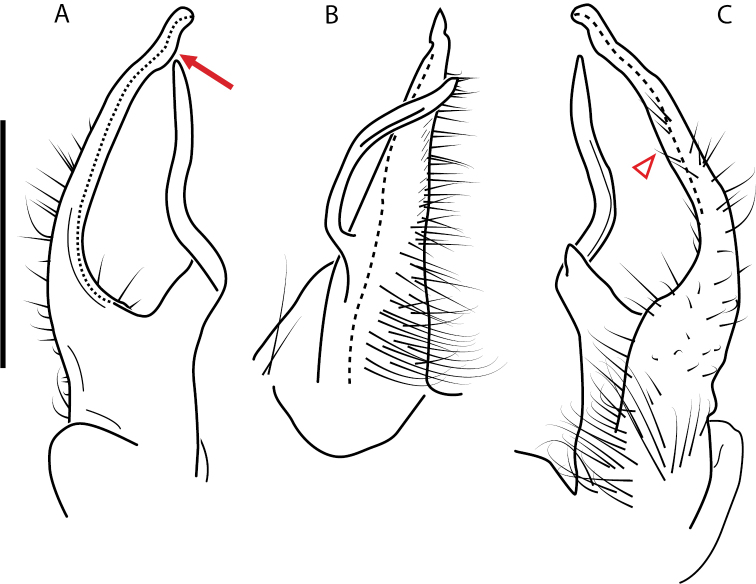
*Nannariaserpens* sp. nov. holotype ♂ (VTEC, MPE00202) left gonopod **A** anterior view; red arrow indicates medial flange **B** medial view **C** posterior view; red triangle indicates slight medial acropodite swelling. Scale bar: 0.5 mm.

**Figure 31.**
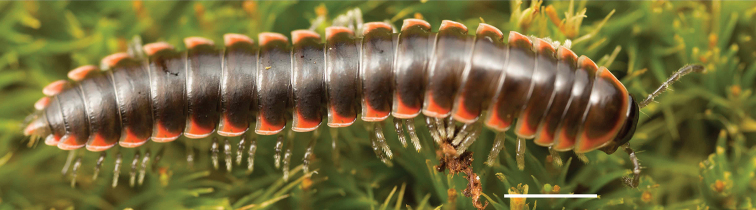
*Nannariaserpens* sp. nov. holotype ♂ (VTEC, MPE00202) coloration. Scale bar: 4.0 mm.

Figs 30 , 31 Vernacular name: “The Serpentine Twisted-Claw Millipede”

####### Material examined.

***Holotype*:** United States – **Virginia** • ♂; Carroll County, Dugspur, side of hill southeast of Blacksnake Meadery; 36.7747°N, -80.5420°W; elev. 822 m; 17 Sep. 2014; hand collected; J. Means leg.; VTEC MPE00202.

***Paratypes*:** United States – **Virginia** • 1 ♂; same collection data as holotype; VMNH MPE00210.

####### Other material.

United States – **North Carolina** • 1 ♂; Ashe County, Three-Top Mtn., 2 miles SE of Creston; 36.4144°N, -81.6003°W; elev. 1219 m; 23 July 1963; R. Hoffman, Carico leg.; VMNH NAN0206 • 2 ♂♂; Avery County, Grandfather Mtn.; 36.1096°N, -81.8114°W; C. M. leg.; VMNH NAN0205; SCAU – **Virginia** • 1 ♂; Carroll County, Blacksnake Meadery, bottomland near creek at entrance; 36.7760°N, -80.5446°W; elev. 767 m; 14 Sep. 2014; hand collected; J. Means leg.; VTEC MPE00274 • 1 ♀; same collection data as preceding; VTEC MPE00204 • 1 ♂; Floyd County, on hill edge by side of road across from Rocky Knob Picnic Area; 36.8132°N, -80.3495°W; elev. 970 m; 18 Sep. 2015; hand collected; J. Means, P. Marek, K. Lawler, P. Shorter, V. Wong leg.; VTEC MPE00817 • 1 ♀; same collection data as preceding; VTEC MPE03717 • 6 ♂♂; Floyd County, 6 mi SE Willis, Felker’s property, Rt. 726, Sorex study; 36.8009°N, -80.3988°W; 7 June 1995; J. Anderson leg.; VMNH NAN0083 • 3 ♂♂; same collection data as preceding; 20 June 1995; VMNH survey leg.; VMNH NAN0087 • 2 ♂♂; same collection data as preceding; 7 June 1995; J. Anderson leg.; VMNH, NAN0089 • 12 ♂♂; same collection data as preceding; 4 June 1993; VMNH NAN0207 • 4 ♂♂; Floyd County, Buffalo Mountain N.A.P. south slope site, berleseate; 36.7958°N, -80.4772°W; elev. 1067 m; 9 Aug. – 6 Sep. 2000; berlese; Joint Survey leg.; VMNH NAN0084 • 1 ♂; same collection data as preceding; 23 Oct. 1996; VMNH survey leg.; VMNH NAN0088 • 1 ♂; same collection data as preceding; 18 Aug.1991; VMNH NAN0090 • 6 ♂♂; same collection data as preceding; 6 Sep.– 3 Oct. 2000; VMNH NAN0091 • 1 ♂♂; same collection data as preceding; 12 Jan. 1986; R. Hoffman leg.; VMNH NAN0092 • 6 ♂♂; same collection data as preceding; 10 Oct. – 8 Nov.; VMNH survey leg.; VMNH NAN0093 • 30 ♂♂; same collection data as preceding; 23 June – 15 July 2001; VMNH NAN0094, 95 • 9 ♂♂; same collection data as preceding; 23 Nov. 2000; Joint Survey; VMNH NAN0096 • 24 ♂♂; same collection data as preceding; 23 Aug. – 29 Sep. 2001; VMNH survey leg.; VMNH NAN0097 • 15 ♂♂; same collection data as preceding; 31 Oct. 2001; VMNH NAN0098 • 5 ♂♂; same collection data as preceding; 19 Aug. 1992; VMNH NAN0208 • 3 ♂♂; same collection data as preceding; 1 Apr. 1993; VMNH NAN0209 • 1 ♂; same collection data as preceding; 3 Sep. 1967; R. Hoffman leg.; VMNH NAN0296 • 1 ♂; Lee County, Powell Mountain at Va. Hy. 70, near crest; 36.6211°N, -83.1062°W; 5 June 1986; R. Hoffman leg.; VMNH NAN0153 • 2 ♂♂; Patrick County, Rock Castle Creek, Rock Castle Gorge Trail, Rocky Knob Recreation Center; 36.7858°N, -80.3717°W; elev. 839 m; 21 Sep. 2015; hand collected; J. Means, P. Marek leg.; VTEC MPE00830, 833 • 1 ♂; Patrick County, side of hill by Fred Clifton Park; 36.7181°N, -80.3246°W; elev; 869 m; hand collected; J. Means, P. Shorter; leg.; VTEC MPE02610 • 2 ♀; same collection data as preceding; VTEC MPE02629, 2630 • 3 ♂♂; Rockbridge County, vicinity of Natural Bridge, in dry limestone woods; 37.63°N, -79.5433°W; 14 May 1989; R. Hoffman leg.; VMNH NAN0193. For detailed collection data see Suppl. material 7.

####### Diagnosis.

Adult males of *Nannariaserpens* sp. nov. are distinct from other *Nannaria* and the sympatric *N.wilsoni*, based on the following combination of characters: ***Gonopods*.** Gonopodal acropodite gently curving medially, not strongly curving medially as in *N.wilsoni*. Telopodite basal zone height < 1/2 length of acropodite, not heavily reduced as in *N.wilsoni*. Acropodite with slightly swollen medial area (Fig. 30C, red triangle) and small lobed medial flange (Fig. 30A, red arrow) basal to apex. Tip directed medially, blunt and simple, not with folds, grooves, flanges as in *N.wilsoni*. Distal zone greatly reduced, not enlarged and curving caudomedially as in *N.wilsoni*. Prefemur with sinuous, cephalically-directed prefemoral process, not curving medially as in *N.wilsoni*. Prefemoral spine enlarged, cephalically directed, lacking secondary hump as in *N.mcelroyorum* sp. nov. ***Color*.** Tergites with bright orange paranotal spots (Fig. 31). Dark brown background. Dorsum of collum smooth with orange margin.

####### Measurements.

♂ holotype (VTEC, MPE00202): BL = 30.9, IW = 2.4, ISW = 0.8, B11W = 4.7, B11H = 2.9; ♀ paratype (VTEC, MPE00204): BL = 29.4, CW = 3.4, IW = 1.8, ISW = 0.8, B11W = 4.4, B11H = 3.0.

####### Variation.

No known variation.

####### Distribution.

Known from southwestern Virginia and northwestern North Carolina, with disjunct populations ca. 112 km northeast at Natural Bridge State Park, and ca. 230 km southwest at Powell Mountain (North Carolina: Ashe and Avery counties; Virginia: Carroll, Floyd, Lee, Patrick and Rockbridge counties, Suppl. material 7; Fig. 126). Distribution area: 2,280 km^2^; status: SRE.

####### Ecology.

Individuals of *N.serpens* sp. nov. have been collected from mesic hardwood forests composed of oak, maple, tuliptree, sassafras, and rhododendron, often found under leaves on wooded hillsides.

####### Etymology.

This species is named for both the serpentine nature of its prefemoral process, and for its type locality, Blacksnake Meadery. The specific name is derived from the Latin *serpens* meaning snake, and is a noun in apposition.

####### Type locality.

United States, Virginia, Carroll County, Dugspur, side of hill southeast of Blacksnake Meadery, 36.7747°N, -80.5420°W.

##### *castanea* clade

**Components.***Nannariacastanea* (McNeill, 1887), *N.davidcauseyi* Causey, 1950, *N.hokie*, *N.missouriensis* Chamberlin, 1928, *N.stellapolis* sp. nov., *N.stellaradix* sp. nov. and a female from Poor Mountain, Virginia. Members of the *castanea* clade have a distinctly long, curving acropodite with a slight medial swelling before the acropodite apex and a medial flange at the tip which carries the prostatic groove, though this flange is much reduced in *N.stellapolis* sp. nov. and *N.stellaradix* sp. nov. No specimens of *N.davidcauseyi* were collected for this study, however due to close geographic proximity and the combination of shared gonopodal characters, including those typical for the clade mentioned above, we suggest that *N.davidcauseyi* belongs in the *castanea* clade.

**Distribution.** The *castanea* clade falls out into two distinct groups, one at the western extents of the Nannariini range and the other in southwestern Virginia. The *castanea* clade therefore has one of the largest distributions of all *Nannaria* clades, extending from southwestern Virginia, to central and northern Mississippi, and north into Arkansas, Indiana, and Missouri (Fig. 114). We expect that additional sampling of areas between these two geographic groups, such as Claytor Lake State Park, Natchez Trace State Park and throughout Tennessee, as well as male specimens from Poor Mountain, Virginia, will reveal additional forms of the *castanea* clade, and help explain its extensive geographic distribution.

###### Nannaria castanea

Taxon classificationAnimaliaPolydesmidaXystodesmidae

(McNeill, 1887)

272CC2A3-D33D-560E-AEE5-374413C5D905

**Figure 32.**
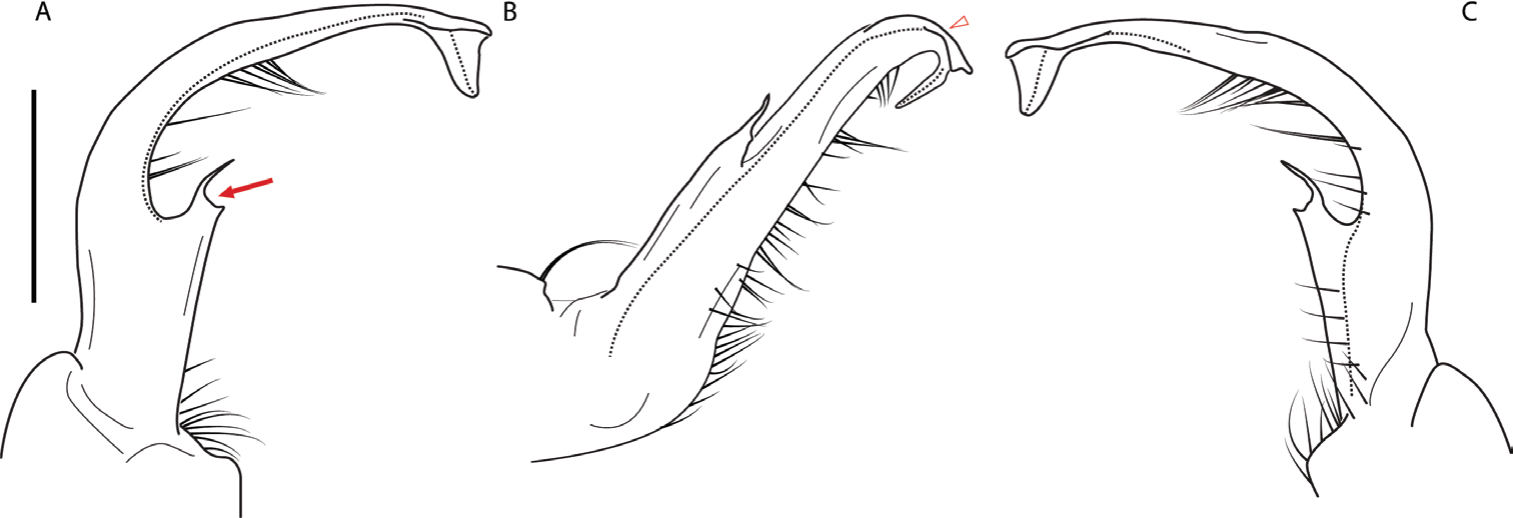
*Nannariacastanea* lectotype ♂ (NMNH, Type #38) left gonopod **A** anterior view; red arrow indicates projected, stout prefemoral spine **B** medial view; red triangle indicates distal zone medial and lateral flanges **C** posterior view. Scale bar: 0.5 mm.

**Figure 33.**
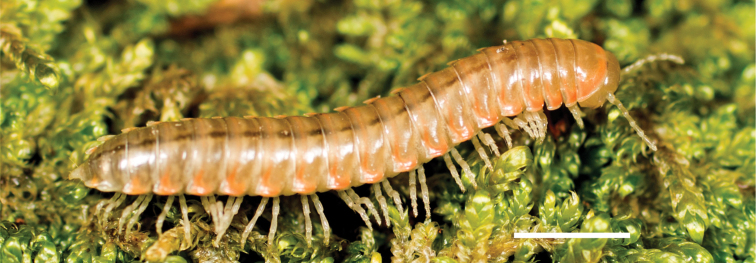
*Nannariacastanea* (McNeill, 1887) ♀ (VTEC, MPE02789) coloration. Scale bar: 4.0 mm.

Figs 32 , 33 Vernacular name: “The Bloomington Twisted-Claw Millipede”

Polydesmus castaneus McNeill, 1887: 329, fig. 8
Fontaria castanea : Bollman 1893: 123.
Mimuloria castanea : Chamberlin 1928: 155. Causey 1952: 8, fig. 6c; 1955: 30. Chamberlin and Hoffman 1958: 37. Hennen and Shelley 2015: 6, figs 3, 9–14.
Nannaria castanea : Chamberlin 1949: 4. Hoffman 1999: 365–366. Marek et al. 2014: 36. Means et al. 2021:17, S68.
Castanaria castanea : Causey 1950a: 1.
Castanaria depalmai : Causey 1950a: 1–3, fig. 1.
Mimuloria depalmai : Causey 1952: 9. Chamberlin and Hoffman 1958: 37–38.
Nannaria depalmai : Hoffman 1999: 366. Marek et al. 2014: 36.

####### Material examined.

***Lectotype* (here designated)**: United States – **Indiana** • ♂; Monroe County, Bloomington; NMNH Type #38.

***Paralectotype*:** United States – **Indiana** • 1 ♀; same collection data as holotype; NMNH Type #38.

####### Other material.

United States – **Arkansas** • 11 ♀♀; Carroll County, Eureka Springs, Lake Leatherwood, Hyde Trail south of lake; 36.4307°N, -93.7576°W; elev. 317 m; 17 May 2017; hand collected; J. Means, D. Hennen, V. Wong leg.; VTEC MPE02765–70, MPE02797–99, MPE03766, MPE03767 • 1 ♂; Fulton County, Hwy. 62; 24 Apr. 1952; N. Causey leg.; VMNH NAN0196; SCAU – **Mississippi** • 1 ♂; Choctaw County, 4.5 mi. S. of Ackerman, upland woods; 33.2809°N, -89.1690°W; 2 Dec. 1961; L. Hubricht leg.; VMNH NAN0238 • 3 ♀♀; Tishomingo County, Burnsville, Divide Wildelife Management Area, forest beside pulloff on road; 34.8051°N, -88.3063°W; elev. 159 m; 14 May 2017; hand collected; J. Means, D. Hennen, V. Wong leg.; VTEC MPE02789–91 • 1 ♂; Tishomingo County, wooded hillside 1.6 miles W of Burnsville; 34.8388°N, -88.3298°W; 27 Feb. 1961; L. Hubricht leg.; VMNH NAN0241; SCAU – **Missouri** • 1 ♂; Crawford County, 5 miles W of Berryman; 37.9188°N, -91.1871°W; 2 Apr. 1955; R. Crabill leg.; VMNH NAN0242. For detailed collection data see Suppl. material 7.

####### Diagnosis.

Adult males of *Nannariacastanea* are distinct from other *Nannaria* based on the following combination of characters: ***Gonopods*.** Gonopodal acropodite gently curving medially, not curving ventromedially before apex as in *N.hokie*. Distal zone with medial flange at 135° to solenomere, and lateral flange at 90° angle to solenomere (Fig. 32B, red triangle), not encircling tip as in *N.hokie*. Acropodite tip curving caudally. Acropodite robust, simple; not thin, with small medial flange near apex as in *N.hokie*. Telopodite basal zone height ca. 1/2 length of acropodite, not ca. 1/3 length as in *N.hokie*. Prefemoral process small, thin, curving medially, arising from top of projected, stout prefemoral spine (Fig. 32A, red arrow), not arising from thin, projected prefemoral spine as in *N.hokie*. ***Color*.** Tergites with orange paranotal spots (Fig. 33) and occasionally faint orange stripes (Fig. 33). Dark to light brown background. Dorsum of collum smooth with orange margin.

####### Measurements.

Lectotype ♂ (NMNH, Type #38): CW = 3.5, IW = 2.2, ISW = 0.8. BL, B11W and B11H were unmeasurable due to the poor quality of the specimen.

####### Variation.

*Castanariadepalmai* was synonymized with *N.castanea* by Hennen and Shelley (2015) without justification and the relationship between *C.depalmai* and *N.castanea* has not been tested using molecular evidence. Morphological differences between the two are extremely slight, however, so it is likely that the synonymy of *C.depalmai* and *N.castanea* will be supported by molecular phylogenetics. Interestingly, there are six specimens of *N.castanea* collected from two localities in Mississippi by Leslie Hubricht in 1961 that Hennen and Shelley (2015) were apparently unaware of and did not mention in their treatment of *Mimuloria*. The northern Mississippi specimen is remarkably similar in form to *N.missouriensis*, however specimens collected by the authors are genetically more similar to *N.castanea* from Arkansas than *N.missouriensis*, and we therefore identify the two Mississippi populations as *N.castanea*, awaiting more detailed investigations.

####### Distribution.

Known from central Indiana west to Missouri and south into Arkansas and Mississippi (Indiana: Monroe County; Missouri: Jefferson, Crawford, Dent, Wayne, Wright counties; Arkansas: Carroll, Searcy, Stone, Fulton counties; Mississippi: Tishomingo, Choctaw counties; Suppl. material 7; Fig. 129). Distribution area: 113,102 km^2^; status: WRE.

####### Ecology.

Individuals of *N.castanea* were collected from both mesic broadleaved and xeric semi-evergreen forests, often beneath 1–2 cm of soil.

####### Etymology.

McNeill (1887) gave no etymology for his choice of *castaneus* but he mentioned the dark chestnut coloration of the species, suggesting that his choice of name may have been derived from the Latin *castaneus* for ‘of the color of chestnuts.’

####### Type locality.

United States, Indiana, Monroe County, Bloomington.

####### Notes.

In the original description, McNeill (1887: 329) stated that he examined three specimens but did not designate a holotype, and no lectotype has been designated by subsequent authors. Therefore, we consider two type specimens we examined as syntypes and here we designate a lectotype male. There is no information on who collected the type material and when. The label of “NMNH Type #38” was presumeably added by an unknown individual after the specimens were deposited at the NMNH.

###### Nannaria davidcauseyi

Taxon classificationAnimaliaPolydesmidaXystodesmidae

Causey, 1950

3A7F611E-2AAD-5328-931F-180198BE83A1

**Figure 34.**
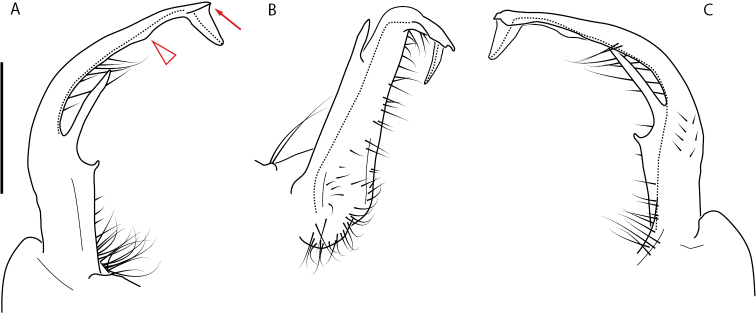
*Nannariadavidcauseyi* Causey, 1950 ♂ (VMNH, NAN0537) left gonopod **A** anterior view; red triangle indicates small acropodite medial flange, red arrow indicates medial flange at apex **B** medial view **C** posterior view. Scale bar: 0.5 mm.

Fig. 34 Vernacular name: “David Causey’s Twisted-Claw Millipede”

Nannaria davidcauseyi Causey, 1950b: 194, figs 3, 4. Hoffman 1999: 366. Marek et al. 2014: 36. Means et al. 2021: S69.
Mimuloria davidcauseyi : Chamberlin and Hoffman 1958: 37. Hennen and Shelley 2015: 10, figs 4–8, 16, 17.

####### Material examined.

United States – **Arkansas** • 1 ♀; Newton County, Dismal Hollow; 35.8506°N, -93.2701°W; 2 Nov. 2013; pitfall trap; M. Skvarla leg.; NCSM NAN0539 • 2 ♂♂; Newton County, Steel Creek, Buffalo Nt River; 36.0380°N, -93.3402°W; 7 Nov. 2013; pitfall trap; M. Skvarla leg.; NCSM NAN0537. For detailed collection data see Suppl. material 7.

####### Diagnosis.

Adult males of *N.davidcauseyi* are distinct from other *Nannaria* and the nearby *N.castanea* based on the following combination of characters: ***Gonopods*.** Gonopodal acropodite gently curving medially, and distal zone with medial and lateral flanges at 90° angle to solenomere, not with medial flange at 135° angle to solenomere as in *N.castanea* (Fig. 34A, red arrow). Acropodite with small medial flange near apex, not simple lacking flange as in *N.castanea* (Fig. 34A, red triangle). Telopodite basal zone height 1/2 length of acropodite. Prefemoral process pronounced, acicular, arising distantly from small blunt prefemoral spine, not from top of projected prefemoral spine as in *N.castanea*. ***Color*.**Hennen and Shelley (2015) described specimens of *N.davidcauseyi* as having clear paranota with irregular orange spots internally, and orange/pink collum margins.

####### Measurements.

♂ (VMNH, NAN0537): BL = 23.3, CW = 3.0, IW = 1.5, ISW = 0.6, B11W = 3.8, B11H = 2.2.

####### Variation.

Causey (1950b: 194) mentions that the male paratype of *N.davidcauseyi* has variation in the shape of both the prefemoral spine and the “keel at the end of the main blade” referring to the caudally-directed acropodite tip.

####### Distribution.

Known only from the northwest corner of Arkansas (Arkansas: Johnson and Newton counties, Fig. 129). Distribution area: 131 km^2^; status: MRE.

####### Ecology.

Causey (1950b) describes the habitat in which she and her husband found *N.davidcauseyi* as an eastern-facing hillside in an oak-hickory woodland. Two individuals of *N.davidcauseyi* were collected from Steel Creek Campground in Arkansas by M. Skvarla (NAN0537) who listed oak, hickory, American beech, and eastern red cedar as the primary tree species in the area (Skvarla et al. 2015).

####### Etymology.

Causey (1950b) gave no explanation of her choice in name for this species, however it is presumed that this species is named after the author’s husband and its collector, Dr. David Causey.

####### Type locality.

United States, Arkansas, Newton County, about three miles northwest of Jasper.

####### Notes.

In the original description Causey (1950b: 194) designates a male holotype and one male and two immature paratypes collected by Dr. David Causey on 25 August 1950. Hennen and Shelley (2015: 10) refer to a female paratype and one immature paratype, which would suggest that Causey’s original identification of the female specimen as an immature was incorrect.

###### Nannaria hokie

Taxon classificationAnimaliaPolydesmidaXystodesmidae

Means, Hennen & Marek, 2021

11ACA8B4-D458-5F9B-BD8E-B3A44CF71419

**Figure 35.**
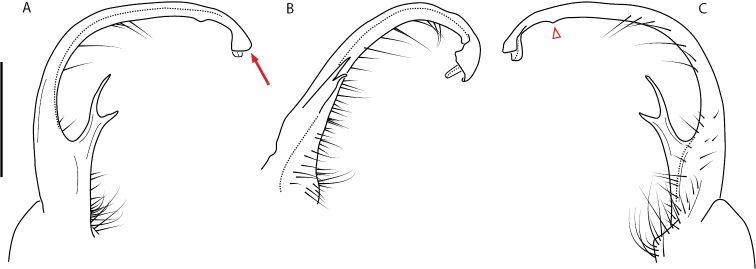
*Nannariahokie* Means, Hennen & Marek in Means et al. 2021, paratype ♂ (VTEC, MPE00880) left gonopod. **A** anterior view; red arrow indicates hood-like flange partially obscuring view of projected solenomere **B** medial view **C** posterior view; red triangle indicates medial flange. Scale bar: 0.5 mm.

**Figure 36.**
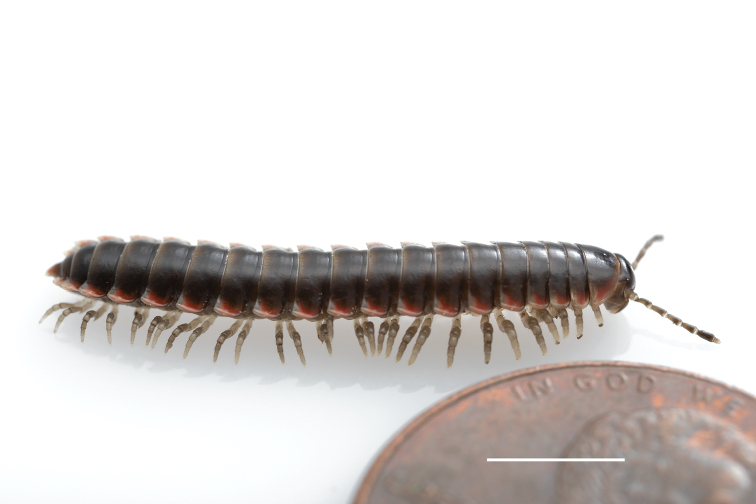
*Nannariahokie* Means, Hennen & Marek in Means et al. 2021, holotype ♂ (VTEC, MPE04803) coloration. Scale bar: 4.0 mm.

**Figure 37.**
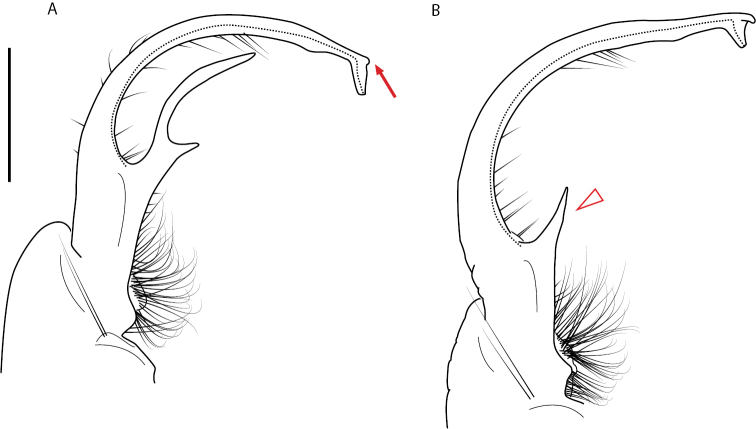
*Nannariahokie* Means, Hennen & Marek, 2021 variation **A** Floyd County, Virginia non-type ♂ (VMNH, NAN0003; red arrow indicates reduced lateral and medial flanges) **B** Pulaski County, Virginia non-type ♂ (VTEC, MPE00253); red triangle indicates prefemoral process lacking prefemoral spine. Scale bar: 0.5 mm.

Figs 35 , 36 , 37 Vernacular name: “The Virginia Tech Twisted-Claw Millipede”

Nannaria hokie Means, Hennen & Marek, in Means et al. 2021: 4, 5, 7, 10, 17–19, 24, S70.

####### Material examined.

***Holotype*:** United States – **Virginia** • ♂; Montgomery County, Blacksburg, Virginia Tech campus, Stadium Woods; 37.2216°N, -80.4159°W; elev. 637 m; 17 Apr. 2019; hand collected; P. Marek leg.; VTEC MPE04803.

***Paratypes*:** United States – **Virginia** • 5 ♂♂; Montgomery County, Blacksburg, Virginia Tech campus, south side of Duck Pond; 37.2250°N, -80.4276°W; elev. 625 m; 13 Oct. 2015; hand collected; D. Hennen leg.; VTEC MPE00880, 882, 883, VMNH, MPE00884, 886 • 2 ♀♀; same collection data as preceding; VTEC MPE00881, 885.

####### Other material.

United States – **Virginia** • 4 ♂♂; Floyd County, along Goose Creek, ca. 3 mi. N of Simpsons, 1850 ft.; 37.0815°N, -80.2055°W; 9 Oct. 1971; R. Hoffman, Knight leg.; VMNH NAN0005, 0244 • 10 ♂♂; Montgomery County, Golden Hills Disc Golf course, in gully; 37.1729°N, -80.4078°W; elev. 628 m; 8 Nov. 2014; hand collected; J. Means, D. Hennen, P. Marek leg.; VTEC MPE00266, 275, 280, 283–89 • 5 ♂♂; Montgomery County, Blacksburg, Virginia Tech, patch of woods between Grove Ln. and Duck Pond; 37.2247°N, -80.4260°W, elev. 628 m; 9 Nov. 2017; hand collected; J. Means, D. Hennen, G. Schiermeyer leg.; VTEC MPE03464–68 • 1 ♂; Montgomery County, Blacksburg, end of Valley View Drive near quarry; 37.2218°N, -80.3894°W; elev. 671 m; 13 Sep. 2019; hand collected; J. Means, D. Hennen, P. Marek, F. Vasquez, I. Huerta leg.; VTEC MPE04998 • 2 ♂♂; Montgomery County, Gateway Trail, 0.5 mi in from main trail by stream; 37.2506°N, -80.4610°W; elev. 642 m; 26 Sep. 2014; hand collected; J. Means, P. Marek, E. Francis, K. Lawler, N. Zegler leg.; VTEC MPE00227, 229 • 4 ♂♂; Montgomery County, Blacksburg, 803 Airport Rd.; 37.2125°N, -80.4053°W; elev. 581 m; 3 Mar. 2018; hand collected; J. Means, P. Marek, G. Schiermeyer, C. Hall leg.; VTEC MPE03829, 3830, 3833, 3835 • 4 ♀♀; same collection data as preceding; VTEC MPE03831, 3832, 3834, 3836 • 1 ♂; Montgomery County, Blacksburg, 505 Fairview Ave., ex puddle of water at base of driveway in neighborhood; 37.212°N, -80.40663°W; elev. 640 m; 13 Mar. 2020; hand collected; P. Marek leg.; VTEC MPE05027 • 1 ♂; Montgomery County, Blacksburg, jct. Airport and Fairview Rds.; 37.2122°N, -80.4060°W; elev. 634 m; 19 Feb. 2018; hand collected; P. Marek leg.; VTEC MPE03808 • 1 ♀; Montgomery County, Blacksburg, Virginia Tech campus, southern end of Duck Pond; 37.2251°N, -80.4272°W; elev. 623 m; 8 June 2017; G. Scheirmeyer leg.; VTEC MPE03705 • 1 ♂; Montgomery County, Blacksburg; 37.1849°N, -80.4087°W; 28 Oct. 1956; R. Hoffman leg.; VMNH NAN0234 • 20 ♂♂; Montgomery County, south Blacksburg, nr jct. Rt. 775 and US 460, under stones in grassy field; 37.1849°N, -80.4087°W; 3 Nov. 1956; R. Hoffman leg.; VMNH NAN0320, 321 • 1 ♂; Montgomery County; Oct. 1950; R. Hoffman leg.; VMNH NAN0235 • 5 ♂♂; Montgomery County, Blacksburg, Trillium Vale; 37.2294°N, -80.4141°W; 10 Oct. 1950; R. Hoffman leg.; VMNH NAN0243 • 6 ♂♂; Pulaski County, Highland Farm, bottom of hill in dry stream bed; 37.1775°N, -80.6456°W; elev. 574 m; 27 Dec. 2014; hand collected; J. Means, K. Lawler leg.; VTEC MPE00253–55, 259–61 • 3 ♀♀; same collection data as preceding; MPE00262–64 • 1 ♂; Smyth County, 7 mi SE of Chilhowie, NW slope of Iron Mtn.; 36.7100°N, -81.6188°W; 4 May 1964; R. Hoffman leg.; VMNH NAN0003. For detailed collection data see Suppl. material 7.

####### Diagnosis.

Adult males of *Nannariahokie* are distinct from other *Nannaria* and the sympatric *N.ericacea*, based on the following combination of characters: ***Gonopods*.** Gonopodal acropodite long and curving ventromedially before apex, not medially as in *N.castanea* or straight as in *N.ericacea*. Distal zone bent ventroposteriorly, with laminate flange encircling tip forming a hood-like structure around dorso-posteriorly projected. Acropodite solenomere laminate, partially obscuring solenomere when viewed laterally (Fig. 35A, red arrow). Distal zone lacking flat laminate flange, at 90° angle to solenomere as in *N.castanea* or laminate, sinuous, directed ventro-cephalically as in *N.ericacea*. Acropodite with small medial flange near apex (Fig. 35C, red triangle), not with lateral projection as in *N.ericacea*. Telopodite basal zone height ca. 1/3 length of acropodite, not ca. 1/2 length as in *N.castanea*, or < 1/5 as in *N.ericacea*. Prefemoral process small, thin, curving medially, arising from top of projected acuminate prefemoral spine, not large, laminate as in *N.ericacea* or straight as in *N.castanea*. ***Color*.** Tergites with orange paranotal spots (Fig. 36). Dark brown background. Dorsum of collum smooth with orange margin.

####### Measurements.

♂ paratype (VTEC, MPE00880): BL = 18.0, CW = 2.9, IW = 2.3, ISW = 0.8, B11W = 3.5, B11H = 2.2; ♀ paratype (VTEC, MPE00881): BL = 22.9, CW = 3.1, IW = 1.6, ISW = 0.8, B11W = 3.9, B11H = 2.4.

####### Variation.

Little morphological variation exists between individuals collected from Montgomery County, Virginia; however, populations in Floyd and Pulaski counties, Virginia display a level of variation that suggests a closer comparison of these populations may be warranted. Both the Floyd and Pulaski County specimens have a much reduced acropodite “hood” (Fig. 37A, red arrow), and the Pulaski County specimens have highly variable prefemoral processes, including forms with and without a prefemoral spine (Fig. 37B, red triangle).

####### Distribution.

Known only from southwestern Virginia, primarily in the vicinity of Blacksburg, though a single specimen collected from Smyth County, Virginia extends the distribution of the species considerably (Virginia: Floyd, Montgomery, Pulaski, and Smyth counties; Suppl. material 7, Fig. 126). Distribution area: 1,668 km^2^; status: SRE.

####### Ecology.

Individuals of *N.hokie* have been collected from mesic hardwood forests, composed of maple, oak, tuliptree, beech, spicebush, and witch hazel, under deciduous leaf litter, logs, and fallen bark. The Pulaski County specimens were all collected from a 1 m^2^ section of a dry creek, taken from under 1–2 cm hard packed soil on the bank of the creek.

####### Etymology.

Means et al. (2021) named *N.hokie* for its type locality, the campus of Virginia Tech. A *hokie* is a popular term for a member of the Virginia Tech community.

####### Type locality.

United States, Virginia, Montgomery County, Blacksburg, Virginia Tech campus, Stadium Woods, 37.2216°N, -80.4159°W.

####### Notes.

In the original publication Means et al. (2021: 17) designated a male holotype (VTEC MPE04803), five male paratypes (VTEC MPE00880, 882, 883; VMNH MPE00884, 886) and two female paratypes (VTEC MPE00881, 885) from the multiple locations on the campus of Virginia Tech collected by the authors from various dates in 2015 and 2019.

###### Nannaria missouriensis

Taxon classificationAnimaliaPolydesmidaXystodesmidae

Chamberlin, 1928

226B16BC-43D8-5542-AA3F-94D223F89B67

**Figure 38.**
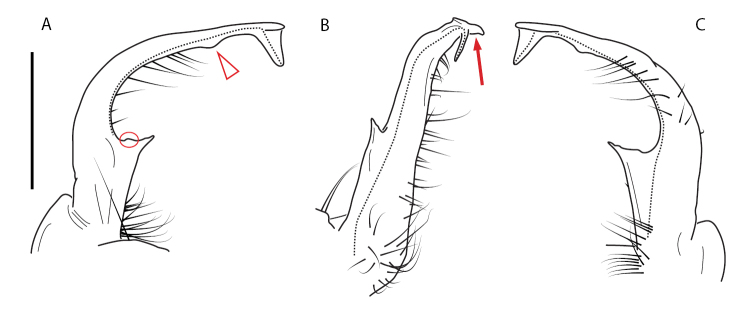
*Nannariamissouriensis* Chamberlin, 1928, holotype ♂ (NMNH, P-6) left gonopod **A** anterior view; red triangle indicates small medial flange on acropodite; red circle indicates reduced prefemoral process **B** medial view; red arrow indicates medial and lateral flanges on tip at 90° to acropodite **C** posterior view. Scale bar: 0.5 mm.

**Figure 39.**
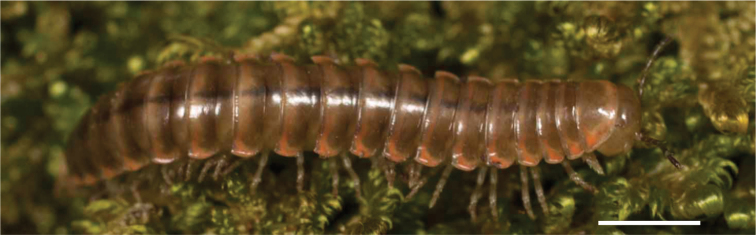
*Nannariamissouriensis* Chamberlin, 1928 ♀ (VTEC, MPE02800) coloration. Scale bar: 4.0 mm.

Figs 38 , 39 Vernacular name: “The Missouri Twisted-Claw Millipede”

Mimuloria missouriensis Chamberlin, 1928: 155; Chamberlin and Hoffman 1958: 37; Hennen and Shelley 2015: 9, fig. 15.
Nannaria missouriensis : Hoffman, 1964: 33; Hoffman 1999: 367. Marek et al. 2014: 37. Means et al. 2021: S70–S71.

####### Material examined.

***Holotype*:** United States – **Missouri** • ♂ St. Charles; 1926; M. J. Brown leg.; NMNH P-6.

***Paratypes*:** United States – **Missouri** • 3 ♂♂; same collection data as holotype; NMNH P-9 • 3 ♀♀; same collection data as holotype; NMNH P-15 • 1 ♀; [Allotype] same collection data as holotype; NMNH IC.

####### Other material.

United States – **Missouri** • 1 ♂, 1 ♀; Cole County, Jefferson Cirty; 38.5767°N, -92.1735°W; 1 Oct. 1944; W. Dowdy leg.; NCSM NAN0535 • 1 ♂, 1 ♀; Morgan County, Versailles; 38.4314°N, -92.8410°W; 1 Apr. 1959; J. Brooks leg.; NCSM NAN0536 • 1 ♀; St. Charles County, Weldon Spring Conservation Area, Lost Valley Trail; 38.6706°N, -90.7515°W; elev. 164 m; 18 May 2017; hand collected; J. Means, D. Hennen, V. Wong; VTEC MPE02800. For detailed collection data see Suppl. material 7.

####### Diagnosis.

Adult males of *Nannariamissouriensis* are distinct from other *Nannaria*, and the nearby *N.castanea*, based on the following combination of characters: ***Gonopods*.** Gonopodal acropodite gently curving medially. Distal zone with medial and lateral flanges at 90° angle to solenomere (Fig. 38B, red arrow), not with medial flange at 135° angle to tip as in *N.castanea*. Acropodite with small medial flange near apex (Fig. 38A, red triangle), not lacking as in *N.castanea*. Telopodite basal zone height ca. 1/3 length of acropodite, not ca. 1/2 length as in *N.castanea*. Prefemoral process reduced to small bump arising from top of projected, stout prefemoral spine (Fig. 38A, red circle), not projected, acicular as in *N.castanea*. ***Color*.** Tergites with orange paranotal spots (Fig. 39) and occasionally faint orange stripes (Fig. 39). Dark to light brown background. Dorsum of collum smooth with orange margin.

####### Measurements.

♂ holotype (NMNH): BL = N/A, CW = 2.9, IW = 1.7, ISW = 0.7, B11W = N/A, B11H = N/A; ♀ paratype (NMNH, IC): BL = 25.4, CW = 1.6, IW = 1.7, ISW = 0.7, B11W = 3.4, B11H = 2.3.

####### Variation.

As Hennen and Shelley (2015) noted, there is some slight variation in the prefemoral process and spine between populations of *N.missouriensis*.

####### Distribution.

Known from central and eastern Missouri (Missouri: Cole, Morgan, Phelps, and St. Charles counties, Suppl. material 7; Fig. 129). Distribution area: 10, 977 km^2^; status: WRE.

####### Ecology.

Individuals of *N.missouriensis* have been collected from mesic deciduous forests dominated by pawpaw, maple, ironwood, and oak, often found under 1–2 cm dark, crumbly soil on hillsides.

####### Etymology.

Chamberlin (1928) gave no etymology for *Nannariamissouriensis*, but it is reasonable to assume that the specific name is in reference the state of Missouri.

####### Type locality.

United States, Missouri, St. Charles.

####### Note.

In the original publication, Chamberlin (1928: 155) mentions eight type specimens, one of which he designated as the holotype (NMNH P-6). Chamberlin (1928) did not mention the sex of the paratypes, but we examined the type material and found three male and four female paratypes, one of which (NMNH IC) was labeled as an allotype. Type specimens were collected in 1926 by M. J. Brown.

###### Nannaria stellapolis sp. nov.

Taxon classificationAnimaliaPolydesmidaXystodesmidae

CB3C73FC-A863-5D21-B540-4D6C0803C380

http://zoobank.org/1C878FE9-4057-4633-81E4-2231D93D7380

**Figure 40.**
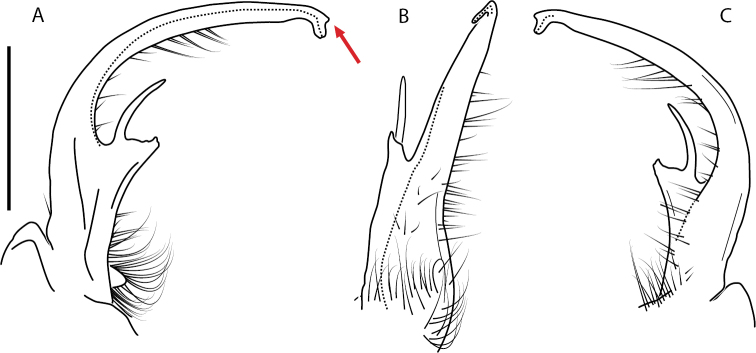
*Nannariastellapolis* sp. nov. holotype ♂ (VTEC, MPE00252) left gonopod **A** anterior view; red arrow indicates lateral flange **B** medial view **C** posterior view. Scale bar: 0.5 mm.

**Figure 41.**
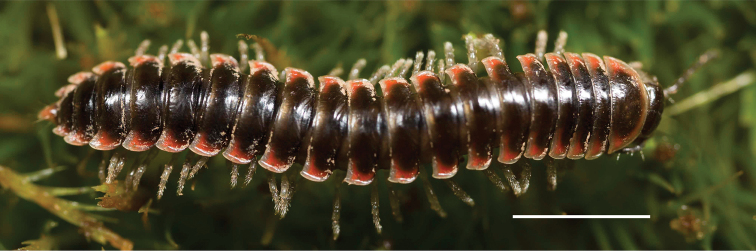
*Nannariastellapolis* sp. nov. holotype ♂ (VTEC, MPE00252) coloration. Scale bar: 4.0 mm.

Figs 40 , 41 Vernacular name: “The Star City Twisted-Claw Millipede”

####### Material examined.

***Holotype*:** United States – **Virginia** • ♂; Roanoke County, Roanoke Mountain picnic area off Blue Ridge Parkway; 37.2309°N, -79.9502°W; elev. 449 m; 25 Oct.2014; J. Means leg.; VTEC MPE00252.

***Paratype*:** United States – **Virginia** • 1 ♂; same collection data as holotype; VMNH MPE00258. For detailed collection data see Suppl. material 7.

####### Diagnosis.

Adult males of *Nannariastellapolis* sp. nov. are distinct from other *Nannaria* and the nearby *N.wilsoni*, based on the following combination of characters: ***Gonopods*.** Gonopodal acropodite long and curving medially before apex, not ventromedially as in *N.hokie*. Acropodite tip with small, triangular lateral flange (Fig. 40A, red arrow), not encircling tip forming a hood-like structure as in *N.hokie*. Acropodite simple, lacking triangular medial flange of *N.hokie*. Acropodite without heavily sclerotized laminate corkscrew as in *N.wilsoni*. Telopodite basal zone height ca. 1/2 length of acropodite, not < 1/3 length as in *N.hokie* or ca. 1/6 length as in *N.wilsoni*. Telopodite with small basal zone lateral bulge. Prefemoral process small, thin, curving medially and arising from top of large, projected, shelf-like prefemoral spine—not arising from sharp, acuminate prefemoral spine as in *N.hokie*, and not subequal to length of acropodite as in *N.wilsoni*. ***Color*.** Tergites with orange/red paranotal spots (Fig. 41). Dark brown background. Dorsum of collum smooth with orange anterior margin.

####### Measurements.

♂ holotype (VTEC, MPE00252): BL = 24.2, CW = 3.3, IW = 1.7, ISW = 0.7, B11W = 3.7, B11H = 2.3.

####### Variation.

No known variation.

####### Distribution.

Known only from the type locality (Virginia: Roanoke County, Suppl. material 7; Fig. 126). Distribution area: N/A; status: MRE.

####### Ecology.

Individuals of *N.stellapolis* sp. nov. were found in mesic broadleaved forests composed of oak, walnut, and maple, under 1–2 cm of hard packed soil on the bank of a dry creek bed.

####### Etymology.

This species is named for Star City, the common nickname of Roanoke city. The specific name is a noun in apposition derived from the Latin *stella*-, star, and Greek *polis*, city.

####### Type locality.

United States, Virginia, Roanoke County, Roanoke Mountain picnic area off Blue Ridge Parkway, 37.2309°N, -79.9502°W.

###### Nannaria stellaradix sp. nov.

Taxon classificationAnimaliaPolydesmidaXystodesmidae

3F288E87-B5BC-5BC5-AACE-32266D3D2755

http://zoobank.org/8AA670CB-8404-494A-B46D-0EC1415B3867

**Figure 42.**
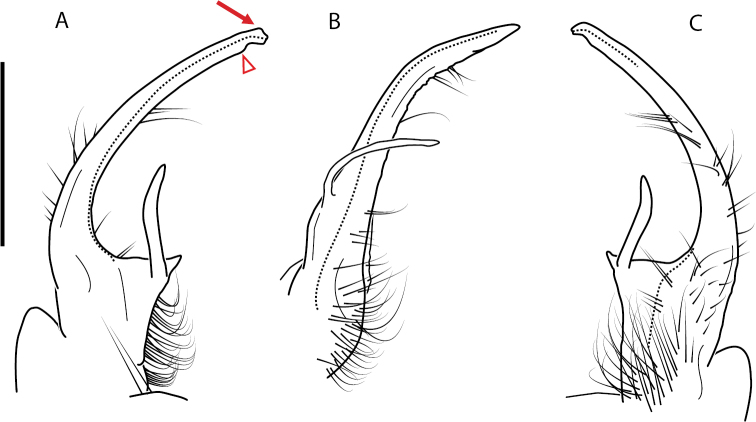
*Nannariastellaradix* sp. nov. holotype ♂ (VTEC, MPE00233) left gonopod **A** anterior view; red arrow indicates lateral flange; red triangle indicates small medial flange **B** medial view **C** posterior view. Scale bar: 0.5 mm.

**Figure 43.**
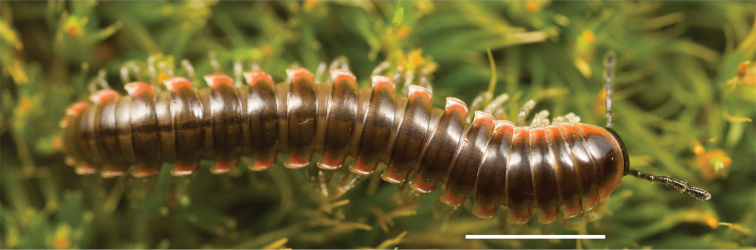
*Nannariastellaradix* sp. nov. holotype ♂ (VTEC, MPE00233) coloration. Scale bar: 4.0 mm.

**Figure 44.**
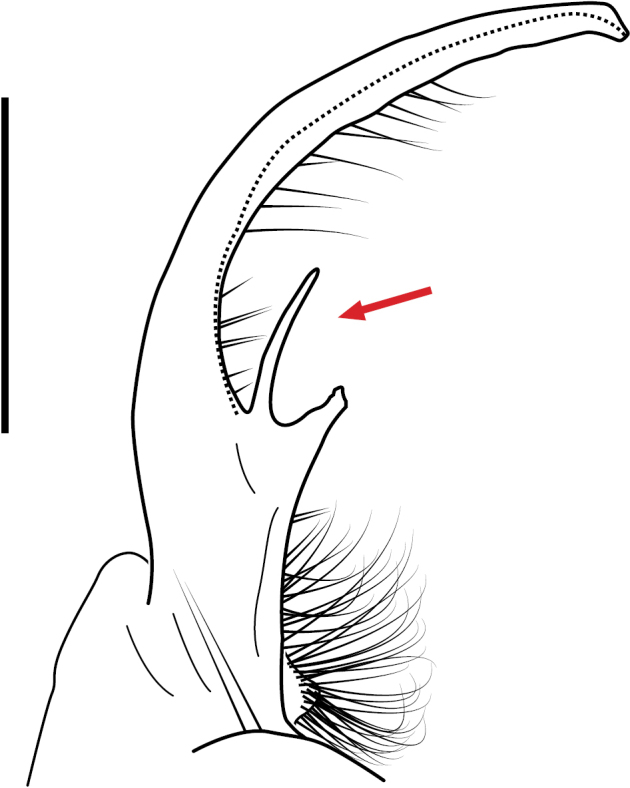
*Nannariastellaradix* sp. nov. non-type ♂ (VMNH, NAN0165) from Pulaski County, Virginia. Red arrow indicates prefemoral process arising from the top of the prefemoral spine. Scale bar: 0.5 mm.

Figs 42 , 43 , 44 Vernacular name: “Starroot’s Twisted-Claw Millipede”

####### Material examined.

***Holotype*:** United States – **Virginia** • ♂; Montgomery County, Riner, 222 Milky way NW, in wetland below lake; 36.9662°N, -80.4179°W; elev. 773 m; 17 Oct. 2014; hand collected; J. Means leg.; VTEC MPE002331.

***Paratype*:** United States – **Virginia** • 1 ♂; same collection data as holotype; VMNH MPE00241 • 3 ♀♀; same collection data as holotype; VTEC MPE00238–240 • 2 ♀♀; same collection data as holotype; VMNH MPE00242, 244.

####### Other material.

United States – **Virginia** • 1 ♂; Floyd County, 2 mi. SW of Copper Valley; 36.9702°N, -80.5425°W; 15 Oct. 1974; R. Hoffman leg.; VMNH NAN0146 • 1 ♂; Floyd County, Big Indian Creek, ca. 2 mi. S of Copper Valley; 36.9615°N, -80.5171°W; 16 Apr. 1970; B. Coombs leg.; VMNH NAN0147 • 1 ♂; Pulaski County, Powhatan Scout Reservation, ca. 1 mi. E. of Macks Creek P.O.; 36.9645°N, -80.6626°W; 6 Mar. 1976; R. Hoffman leg.; VMNH NAN0165. For detailed collection data see Suppl. material 7.

####### Diagnosis.

Adult males of *Nannariastellaradix* sp. nov. are distinct from other *Nannaria* and the sympatric *N.wilsoni*, based on the following combination of characters: ***Gonopods*.** Gonopodal acropodite continually curving medially before apex. Distal zone short, directed medially with small triangular lateral flange (Fig. 42A, red arrow), not directed caudally with enveloping lateral and medial flanges as in *N.hokie*, or enlarged, laminate, as in *N.wilsoni*. Acropodite with small laminate medial flange (Fig. 42A, red triangle). Basal zone > 1/3 length of acropodite, not < 1/5 as in *N.wilsoni*. Prefemur with enlarged prefemoral spine. Prefemur with small, ventrally curving prefemoral process arising dorsomedially from spine, not from top of spine as in *N.hokie*, or long, medially curving, paralleling acropodite as in *N.wilsoni*. ***Color*.** Tergites with light orange paranotal spots (Fig. 43). Dark brown background. Dorsum of collum smooth with orange and white margin.

####### Measurements.

♂ holotype (VTEC, MPE00233): BL = 23.8, CW = 2.9, IW = 1.8, ISW = 0.8, B11W = 3.7, B11H = 2.6; ♀ paratype (VTEC, MPE00238): BL = 25.4, CW = 3.4, IW = 2.2, ISW = 0.9, B11W = 3.9, B11H = 2.8.

####### Variation.

Three populations from Pulaski and Floyd counties in Virginia (VMNH, NAN0146, 147, 165) show some morphological variation (Fig. 44) that may indicate a separate species; however, without molecular evidence, specific recognition is not warranted. Notable variations in these populations include a prefemoral process arising from the top of the prefemoral spine (Fig. 44, red arrow) and the lack of an acropodite medial flange.

####### Distribution.

Known from a linear area in southern Virginia (Virginia: Floyd and Pulaski counties, Suppl. material 7; Fig. 126). Distribution area: 11 km^2^; status: MRE.

####### Ecology.

Individuals of *N.stellaradix* sp. nov. were collected from mesic mixed hardwood and pine habitats predominately composed of maple and white pine; individuals were collected from under leaf litter.

####### Etymology.

This species is named after the owner of the property from which the holotype was found, an artist named Starroot. The specific name is a noun in apposition derived from the Latin *stella*-, star, and -*radix*, root.

####### Type locality.

United States, Virginia, Montgomery County, Riner, 222 Milky way NW, in wetland below lake, 36.9662°N, -80.4179°W.

##### *laminata* clade

**Components.***Nannariacingulata* sp. nov. and *N.laminata*. Members of the *laminata* clade share gonopodal characters, including a blunt acropodite tip and pronounced prefemoral spine. The molecular phylogeny (Fig. 7) would suggest that this clade is either one highly variable species, where eastern and northern forms have developed a cingulum, a basal bulge and a straight acropodite (*N.cingulata* sp. nov.) or, except for *N.cingulata* sp. nov., a fairly morphologically uniform but genetically diverse group of species. Sequencing of additional specimens throughout the range of the *laminata* clade will help illuminate which of the above scenarios are accurate, however, due to the number of morphological apomorphies found in *N.cingulata* sp. nov. specimens, we feel confident in naming *N.cingulata* sp. nov. as a separate species from *N.laminata*.

**Distribution.** The *laminata* clade extends from much of Virginia and the D.C. metro area into eastern West Virginia (Fig. 114).

###### Nannaria cingulata sp. nov.

Taxon classificationAnimaliaPolydesmidaXystodesmidae

778B1B0F-86BC-58FB-AB68-038B18C70DA0

http://zoobank.org/E1CC5FC5-266A-4B04-886B-22587B78B4C4

**Figure 45.**
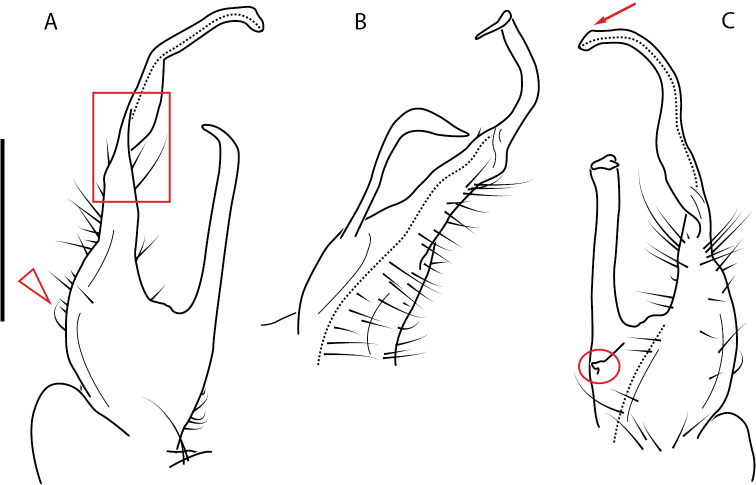
*Nannariacingulata* sp. nov. holotype ♂ (VTEC, MPE02324) left gonopod **A** anterior view; red triangle indicates basal lateral bulge; red rectangle indicates cingulum **B** medial view **C** posterior view; red arrow indicates small lateral flange on acropodite tip; red oval indicates reduced prefemoral spine. Scale bar: 0.5 mm.

**Figure 46.**
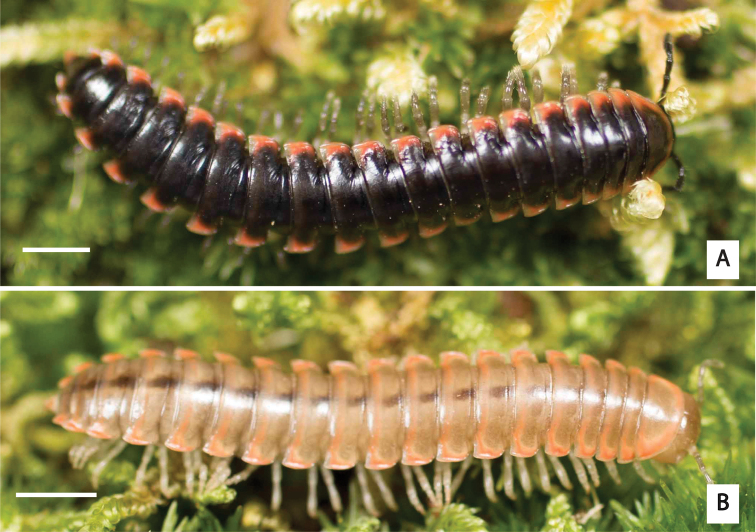
*Nannariacingulata* sp. nov. coloration **A** holotype ♂ (VTEC, MPE02324) orange paranota **B** non-type ♀ (VTEC, MPE01881) faint orange stripes. Scale bars: 4.0 mm.

**Figure 47.**
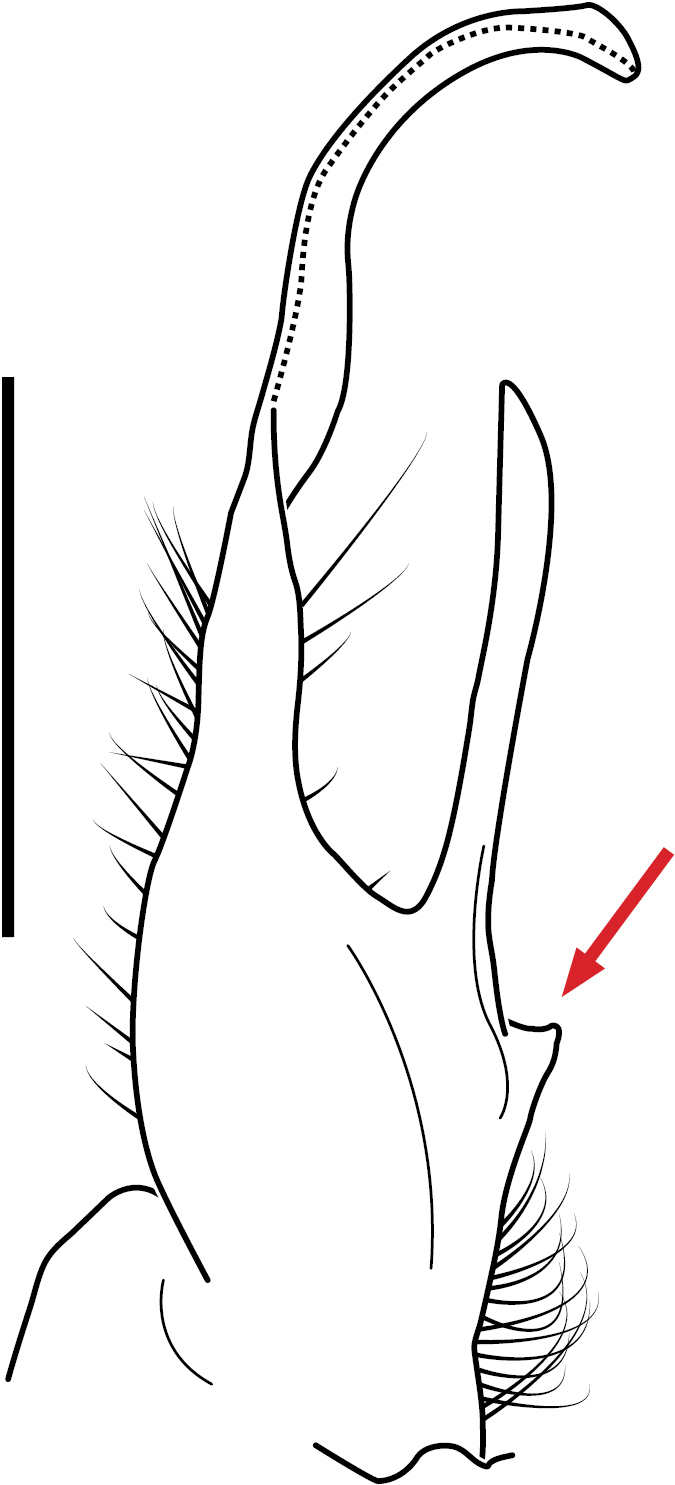
*Nannariacingulata* sp. nov. (VMNH, NAN0041) from Prince Edward Co., Virginia. Red arrow indicates projected prefemoral spine. Scale bar: 0.5 mm.

Figs 45 , 46 , 47 Vernacular name: “The Swamp-Dwelling Twisted-Claw Millipede”

####### Material examined.

***Holotype*:** United States – **Virginia** • ♂; Shenandoah County, Massanutten Trail nr. Signal Knob parking off Route 678; 38.9332°N, -78.3207°W; elev. 250 m; 24 Feb. 2017; hand collected; C. Harden leg.; VTEC MPE02324.

***Paratypes*:** United States – **Virginia** • 11 ♂♂; same collection data as holotype; VTEC MPE02325-2335 • 11 ♂♂; same collection data as holotype; VMNH MPE02336-45, 3668 •1 ♀; Clarke County, nr. Mount Weather; 39.0717°N, -77.9120°W; elev. 326 m; 22 June 2017; hand collected; J. Means, D. Hennen leg.; VTEC MPE01881.

####### Other material.

United States – **Virginia** • 1 ♂; Chesterfield County, Pocahontas State Park; 37.3679°N, -77.5755°W; 11 May 2002; A. Evans leg.; VMNH NAN0221 • 1 ♂; Clarke County, 1.7 miles by road N of Ashby Gap, U.S. Rt. 50 crossing of Blue Ridge; 39.0133°N, -77.9622°W; 10 May 1958; W. Highton leg.; VMNH NAN0227 • 2 ♂; Fairfax County, Hemlock Overlook Regional Park challenge course; 38.7666°N, -77.4104°W; elev. 101 m; 8 Apr. 2017; hand collected; P. Marek, C. Hall leg.; VTEC MPE02438, 2447 • 2 ♂; Fauquier County, Sky Meadows State park, near junction of North Ridge Trail and Appalachian Trail; 38.9850°N, -77.9900°W; elev. 549 m; 21 Apr. 2017; hand collected; C. Harden leg.; VTEC MPE02473, 77 • 2 ♂♂; Frederick County, Albin, ca. 3 mi. W of Winchester on VA. Hy 679; 39.2216°N, -78.1988°W; 10 Apr. 1990; LFCC class leg.; VMNH NAN0225 • 1 ♂; Prince Edward County, Hampden-Sydney College; 37.2381°N, -78.4606°W; 20 April 1983; J. White leg.; VMNH NAN0041 • 3 ♂♂; NAN0230 Prince George County, Lanham; 38.9669°N, -76.8622°W; 1 June; H. Loomis leg.; VMNH NAN0230 • 1 ♂; Shenandoah County, Massanuten Trail ca. 2.5 mi from Signal Knob parking lot by Rte. 698; 38.9324°N, -78.3249°W; elev. 260 m; 24 Feb. 2017; hand collected; C. Harden, G. Chapman leg.; VTEC MPE02348 • 2 ♂♂; Shenandoah County, George Washington National Forest near Eliz. Furn. Group Cmpgrd; 38.9310°N, -78.3220°W; elev. 233 m; 26 Feb. 2017; hand collected; C. Harden leg.; VTEC MPE03437, 03438 • 3 ♂♂; Shenandoah County, George Washington National Forest, Botts Trail, south of Elizabeth Furnace; 38.9240°N, -78.3280°W; elev. 250 m; 21 Apr. 2017; hand collected; C. Harden leg.; VTEC MPE02475, 76, 3673 • 2 ♂♂; Shenandoah County, dry creak near swamp just past Elizabeth Furnace Campground; 38.9122°N, -78.3326°W; elev. 248 m; 22 Apr. 2018; hand collected; J. Means, N. Larson leg.; VTEC MPE03899, 03900 • 1 ♀; same collection data as preceding; VTEC, MPE03901 • 1 ♂; Suffolk County, Magnolia, US Hys. 58-460; 36.7458°N, -76.5408°W; 23 Feb. 1954; R. Ragot leg.; VMNH NAN0229 • 1 ♂; Warren County, Smithsonian Conservation Biology Institute, High Knob Mountain; 38.8930°N, -78.1370°W; elev. 396 m; 13 Mar. 2017; hand collected; C. Harden leg.; VTEC MPE02463 • 6 ♂♂; Warren County, Racetrack Hill, gravel road/field; 38.8915°N, -78.1680°W; elev. 305 m; 4 Apr. 2017; hand collected; C. Harden leg.; VTEC MPE02453–56, 2786, 2787 • 1 ♂; Warren County, SCBI, High Knob Mountain; 38.8930°N, -78.1370°W; elev. 390 m; 6 Mar. 2017; hand collected; C. Harden leg.; VTEC MPE03744 • 1 ♂; Warren County, upper reaches of Stokes Branch 0.17 mi N of dirt forest road terminus, off Rte 613 nr jct w/608; 38.9156°N, -78.2994°W; elev. 265 m; 12 Feb. 2018; hand collected; C. Harden leg.; VTEC MPE03885 • 26 ♂♂; Warren County, 2 mi SE Front Royal, DF site at NZP-CRC, oak woods on hillside; 38.8975°N, -78.1683°W; 1 May 1996; VMNH Survey leg.; VMNH NAN0232 • 4 ♂; York County, Cheatham Annex NSD Jones Mill Pond; 37.2850°N, -76.6416°W; 17 Nov. 1989; Buhlmann, Pague leg.; VMNH NAN0222 • 1 ♂; same collection data as preceding; 2 Feb. 1997; S. Roble leg.; VMNH NAN0223 • 1 ♂; same collection data as preceding; 2 Nov. 1989; K. Buhlmann leg.; VMNH, NAN0224 • 2 ♂♂; same collection data as preceding; 20 Feb. 1990; VDNH survey leg.; VMNH NAN0228 • 1 ♂; same collection data as preceding; 17 Nov. 1989; K. Buhlmann leg.; VMNH NAN0359; SCAU – **West Virginia** • 2 ♂♂, 2 ♀♀; Berkeley County, Sleepy Creek Hunt Area; 39.5057°N, -78.1695°W; 6 May 1968; P. Martinat leg.; NCSM NAN0449, 450. For detailed collection data see Suppl. material 7.

####### Diagnosis.

Adult males of *Nannariacingulata* sp. nov. are distinct from other *Nannaria* and the sympatric *N.shenandoa*, based on the following combination of characters: ***Gonopods*.** Gonopodal acropodite gently curving medially before bending dorsomedially, not ventromedially as in *N.paupertas* sp. nov. and *N.shenandoa*. Distal zone short, bent dorsomedially at 45°, not bent dorsomedially at 90° as in *N.paupertas* sp. nov., or curving posterolaterally as in *N.shenandoa*. Acropodite tip blunt with small, lobed lateral flange (Fig. 45C, red arrow), not with large, pointed flange as in *N.shenandoa*. Telopodite basal zone height < 1/2 length of acropodite with lateral bulge (Fig. 45A, red triangle), not greatly reduced as in *N.shenandoa*, or lacking lateral bulge as in *N.paupertas* sp. nov. Acropodite shaft swollen before apex, with cingulum (Fig. 45A, red rectangle), not simple as in *N.shenandoa*, or with medial flange as in *N.paupertas* sp. nov. Prefemur with prefemoral process straight for first 3/4, and bent ventrally at 90° in last 1/4, not bent ventromedially as in *N.paupertas* sp. nov., or curving laterally as in *N.shenandoa*. Prefemoral process ca. 1/2 length of acropodite, not 2/3 length as in *N.paupertas* sp. nov. or *N.shenandoa*. Prefemoral spine greatly reduced to small, tooth-like projection (Fig. 45C, red oval), not prominent, acicular as in *N.paupertas* sp. nov., or absent as in *N.shenandoa*. ***Color*.** Tergites with orange paranotal spots (Fig. 46A) and occasionally faint orange stripes (Fig. 46B). Dark to light brown background. Dorsum of collum smooth with orange margin.

####### Measurements.

♂ holotype (VTEC, MPE02324): BL = 24.7, CW = 3.9, IW = 2.1, ISW = 0.8, B11W = 4.4, B11H = 2.9. ♀ paratype (VTEC, MPE01881): BL = 28.6, CW = 3.9, IW = 2.7, ISW = 0.9, B11W = 4.8, B11H = 3.3.

####### Variation.

The only *Nannariacingulata* sp. nov. individual known from Prince Edward Co., Virginia has a cephalically directed prefemoral process and a pronounced prefemoral spine (Fig. 47, red arrow).

####### Distribution.

*Nannariacingulata* sp. nov. has a more extensive range than most species in the *minor* species group and can be found from eastern West Virginia south to central and southeastern Virginia, and Maryland in the Washington D.C. metropolitan area (West Virginia: Berkley County; Virginia: Frederick, Clarke, Shenandoah, Warren, Fairfax, Chesterfield, Prince Edward, Gloucester counties, and the City of Suffolk; Suppl. material 7; Fig. 128). Distribution area: 28,825 km^2^; status: WRE.

####### Ecology.

The majority of *Nannariacingulata* sp. nov. specimens collected by C. Harden have been taken from mesic hardwood forests at night and were found walking on top of leaf litter and on man-made paths, or were collected using pitfall traps. Several museum specimens of *N.cingulata* sp. nov. lack ecological notes.

####### Etymology.

This species is named for the presence of a cingulum, a linear groove and possible point of flexion, on its gonopod, a characteristic unique within *Nannaria*. The specific name is a feminized adjective derived from the Latin *cingulatus* for belted.

####### Type locality.

United States, Virginia, Shenandoah County, Massanutten Trail nr. Signal Knob parking off Route 678, 38.9332°N, -78.3207°W.

###### Nannaria laminata

Taxon classificationAnimaliaPolydesmidaXystodesmidae

Hoffman, 1949

E76C06AC-8983-55BE-918A-94BDDEEE05AE

**Figure 48.**
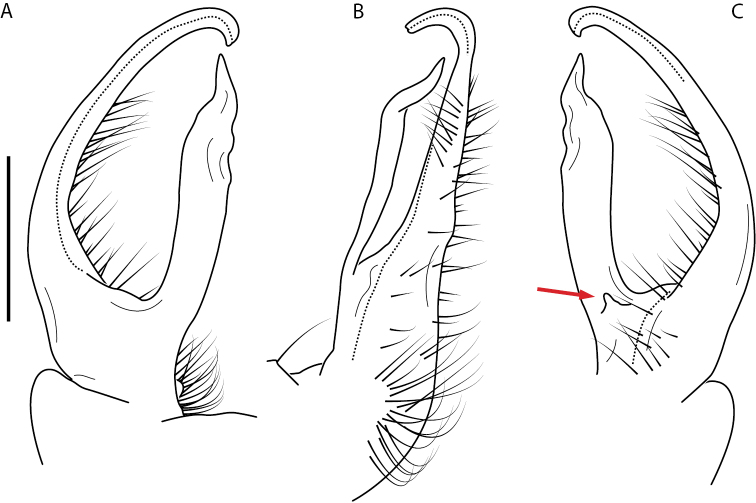
*Nannarialaminata* holotype ♂ (NMNH, Type #1806) left gonopod **A** anterior view **B** medial view **C** posterior view; red arrow indicates reduced, acuminated prefemoral spine. Scale bar: 0.5 mm.

**Figure 49.**
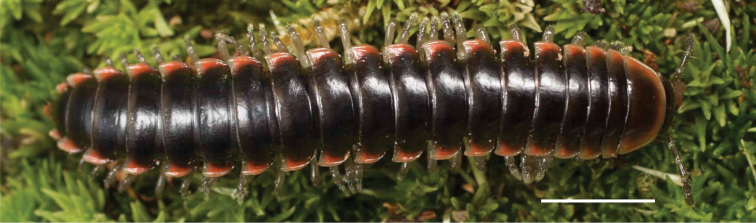
*Nannarialaminata* non-type ♂ (VTEC, MPE02392) coloration. Scale bar: 4.0 mm.

Figs 48 , 49 Vernacular name: “The Laminate Twisted-Claw Millipede”

Nannaria laminata Hoffman, 1949: 383, figs 11, 12. Chamberlin and Hoffman 1958: 41. Hoffman 1999: 367. Marek et al. 2014: 37. Means et al. 2021: S70.

####### Material examined.

***Holotype*:** United States – **West Virginia** • ♂; Mercer County, ravine beside U.S. Route 460, ca. 2 miles south of Glen Lyn, Virginia; [37.3550°N, -80.8982°W]; 12 July 1947; H. H. Hobbs, C. M. Wilson leg.; NMNH Type #1806.

####### Other material.

United States – **Virginia** • 1 ♂; Amherst County, Tarjacket Ridge FS 1167, 3500’; 37.7665°N, -79.1863°W; 13 Nov. 1999; VMNH Survey leg.; VMNH NAN0332 • 1 ♂; Augusta County, across creek from Barnwood Cabin, where old road flattens out by small stream; 37.89643°N, -79.0007°W; elev. 828 m; 18 Mar. 2017; J. Means leg.; VTEC MPE02401 • 1 ♀; same collection data as preceding; VTEC MPE03679 • 1 ♂; Augusta County, far corner of upper Sherando Lake, bank side by marsh; 37.9139°N, -79.0207°W; elev. 607 m; 19 Mar. 2017; J. Means leg.; VTEC MPE02392 • 1 ♂; Bedford County, Flattop Mtn., Peaks of Otter nr MP 82.8; 37.4498°N, -79.5834°W; 27 Oct. 1986; J. Mitchell leg.; VMNH NAN0330 • 3 ♂♂; Bland County, Hamilton’s Cave, ca. 6 km ENE of Mechanicsville, rich wooded hillside; 37.7032°N, -78.1627°W; 16 May 1980; R. Hoffman leg.; VMNH NAN0150 • 2 ♂♂; Botetourt County, Harkening Hill, Blue Ridge Parkway; 37.4577°N, -79.6175°W; 21 Oct. 1989; J. Mitchell leg.; VMNH NAN0333 • 1 ♂; Botetourt County, North Creek, 1–3 mi. east of Arcadia; 37.5411°N, -79.5866°W; 13 Oct. 1973; R. Hoffman leg.; VMNH NAN0334 • 1 ♂; Campbell County, 4 miles NW of Rustburg; 37.3176°N, -79.1526°W; 14 Oct. 1950; L. Hubricht leg.; VMNH NAN0331 • 1 ♂; Cumberland County, clearcut north DF site 2 ca 2 km SSW of Columbia; 37.7361°N, -78.1714°W; 17 May 1990; J. Mitchell leg.; VMNH NAN0253 • 3 ♂♂; same collection data as preceding; Nov. 1989; VMNH NAN0259 • 1 ♂; same collection data as preceding; 2 Apr. 1990; VMNH NAN0261 • 1 ♂; same collection data as preceding; 3 Dec. 1989; VMNH NAN0263 • 1 ♂; same collection data as preceding; 15 Feb. 1990; VMNH NAN0264 • 19 ♂♂; same collection data as preceding; 19 Oct. 1989 VMNH NAN0266 • 7 ♂♂; same collection data as preceding; 16 Nov. 1989; VMNH NAN0267 • 11 ♂♀; same collection data as preceding; Nov. 1989; VMNH NAN0361 • 6 ♂♂; same collection data as preceding; 1 May 1990; VMNH NAN0255 • 1 ♂; Cumberland County, hardwood site 1 (north), 2 km SSW of Columbia; 37.7361°N, -78.1715°W; 1 May 1990; J. Mitchell leg.; VMNH NAN0254 • 24 ♂♂; same collection data as preceding; 17 Mar. 1990; VMNH NAN0268 • 1 ♂; Cumberland County, hardwood site 4 (south), 7 km SSW of Columbia; 37.6953°N, -78,1959°W; 5 Oct. 1989; J. Mitchell; VMNH NAN0257 • 3 ♂♂; Cumberland County, hardwood site 2 – N 2 km SSW Columbia; 37.7361°N, -78.1714°W; VMNH NAN0258 • 2 ♂♂; Cumberland County, pinewoods DF site 5, ca. 5.5 km SSW of Columbia; 37.7048°N, -78.1889°W; 17 May 1990; J. Mitchell leg.; VMNH NAN0532 • 1 ♂; Giles County, Jefferson National Forest, Cascades Day Use Area, within a few hundred meters of the start of the Cascades Trail, near parking lot; 37.3500°N, -80.5835°W; elev. 544 m; 21 Oct. 2015; hand collected; D. Hennen leg.; VTEC MPE00891 • 1 ♂; Giles County, side of Upper Trail on way to Cascades; 37.3613°N, -80.5875°W; elev. 799 m; 18 Feb. 2018; hand collected; J. Means leg.; VTEC MPE03807 • 1 ♂; Giles County, Jefferson National Forest past Mountain Lake Biological Station, past Mini Ball Hill on Rt 613; 37.4209°N, -80.5093°W; elev. 1062 m; 3 Oct. 2016; hand collected; J. Means, D. Hennen, V. Wong leg.; VTEC MPE02124 • 2 ♀♀; same collection data as preceding; VTEC MPE02136, 2142 • 1 ♂; Prince Edward County, Farmville, Price Drive; 37.3019°N, -78.3922°W; 28 June 1988; W. Shear leg.; VMNH NAN0256 • 1 ♂; Prince Edward County, 13 mi S Farmville, dug up 12 cm under surface of clay; 37.1134°N, -78.3922°W; 22 Feb. 1975; W. Shear leg.; VMNH NAN0260 • 1 ♂; Prince Edward County, pitfall trap, old field at Rice; 37.2750°N, -78.2916°W; 17 June 1981; R. Bellinger leg.; VMNH NAN0265; SCAU – **West Virginia** • 1 ♀; Mercer County, just over the WV state line on 460, ca. 2 miles SW of Glen Lyn on 219/8; 37.3452°N, -80.9105°W; elev. 507 m; 12 Nov. 2017; hand collected; J. Means leg.; VTEC MPE03474 • 1 ♂; Monroe County, 5.8 air miles east of Linside, up logging road; 37.4749°N, -80.5673°W; elev. 934 m; 27 Apr. 2017; hand collected; J. Means, D. Hennen, P. Marek, P. Shorter, V. Wong leg.; VTEC MPE02520 • 3 ♀♀; same collection data as preceding; VTEC, MPE02578, 2579, 3675 • 2 ♂♂; Monroe County, along logging road off VA rt 613, down in bowl and on each side of dirt and gravel road; 37.4717°N, -80.5620°W; elev. 1107 m; 27 Apr. 2016; hand collected; J. Means, D. Hennen, P. Marek, P. Shorter, V. Wong leg.; VTEC MPE02528, 2561. For detailed collection data see Suppl. material 7.

####### Diagnosis.

Adult males of *N.laminata* are distinct from other *Nannaria* and the nearby *N.solenas* sp. nov. and *N.wilsoni*, based on the following combination of characters: ***Gonopods*.** Gonopodal acropodite semi-circular, curving dorsomedially with abrupt 90° curve after apex, not straight before abrupt 90° curve as in *N.solenas* sp. nov. or with laminate corkscrew before apex as in *N.wilsoni*. Acropodite tip simple, directed posteriorly, not directed anteriorly as in *N.solenas* sp. nov. and not with triangular lateral flange as in *N.wilsoni*. Tip terminating in sharp claw-like point, not blunt point as in *N.solenas* sp. nov. and *N.wilsoni*. Height of telopodite basal zone > 1/3 length of acropodite, not ca. 1/2 length as in *N.solenas* sp. nov., or ca. 1/5 as in *N.wilsoni*. Prefemoral process large, laminate and serpentine, with prefemoral spine reduced to small, acuminate projection (Fig. 48C, red arrow), not lacking as in *N.wilsoni*. ***Color*.** Tergites with red paranotal spots (Fig. 49). Black background. Dorsum of collum smooth with red margin.

####### Measurements.

♂ holotype (NMNH, Type #1806): BL = 30.5, CW = 4.0, IW = 2.2, ISW = 0.9, B11W = 4.9, B11H = 3.4.

####### Variation.

*Nannarialaminata* occupies a relatively large geographic area, > 10,000 km^2^, and displays a fair amount of morphological variation; however, the general shape of the acropodite and laminate prefemoral process remain constant throughout. Variation is seen in the sharpness and curve of the acropodite tip; some individuals display a more medially directed, blunt tip than the holotype. Additionally, the width of the prefemoral process (when viewed anteriorly or posteriorly) varies widely between individuals.

####### Distribution.

Known from a horizontal strip from central Virginia to just north of the border into West Virginia (Virginia: Amherst, Augusta, Bedford, Botetourt, Campbell, Cumberland, Giles, and Prince Edward counties; West Virginia: Mercer and Monroe counties, Suppl. material 7; Fig. 126). Distribution area: 9,297 km^2^; status: SRE.

####### Ecology.

Individuals of *N.laminata* have been collected from mesic deciduous forests dominated by maple, oak, rhododendron, and some pine, often under 1–2 cm soil and/or leaf litter.

####### Etymology.

Hoffman (1949) gives no explanation for the name *laminata* in his description of the species, however it is reasonable to assume that the name refers to the laminate prefemoral process, the major distinguishing morphological character.

####### Type locality.

United States, West Virginia, Mercer County, ravine beside U.S. Route 460, ca. 2 miles south of Glen Lyn, Virginia; [37.3550°N, -80.8982°W].

####### Notes.

In the original publication, Hoffman (1949: 384) designates a male holotype (NMNH Type #1806) collected by H. Hobbs and C. Wilson on July 12, 1947.

##### *tasskelsoae* clade

**Components.***Nannariatasskelsoae* sp. nov. Based on the molecular phylogeny (Fig. 7) *N.tasskelsoae* sp. nov. is in a monotypic clade (Fig. 113). The *tasskelsoae* clade can be diagnosed by the combination of morphological characters detailed in the below diagnosis, including an enlarged prefemoral spine from which arises a medially curving prefemoral process which parallels the medially curving, simple acropodite.

**Distribution.** the *tasskelsoae* clade is known only from eastern West Virginia (Fig. 127).

###### Nannaria tasskelsoae sp. nov.

Taxon classificationAnimaliaPolydesmidaXystodesmidae

135F2CAA-E8ED-56E6-BD10-EF5806D25F6C

http://zoobank.org/F3B40E44-28D4-4353-BF02-78B6398996D6

**Figure 50.**
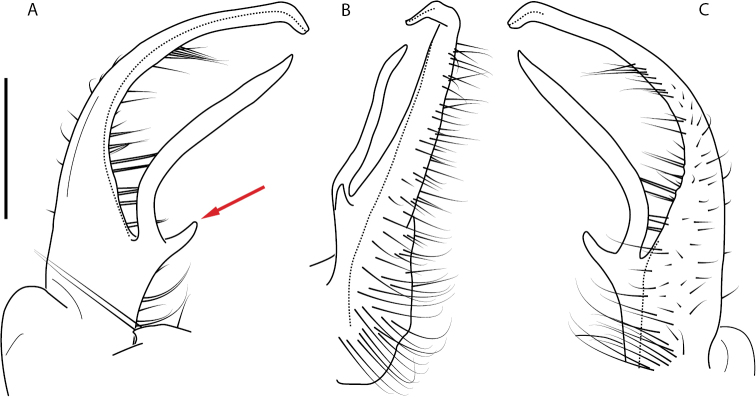
*Nannariatasskelsoae* sp. nov. holotype ♂ (VTEC, SPC000710) left gonopod **A** anterior view; red arrow indicates pronounced, curving prefemoral spine **B** medial view **C** posterior view. Scale bar: 0.5 mm.

**Figure 51.**
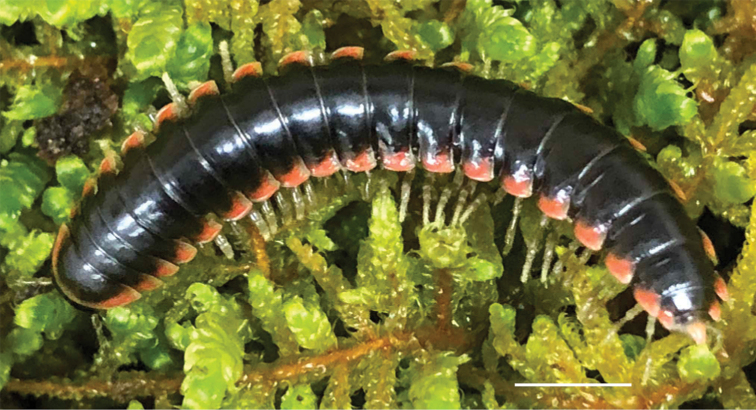
*Nannariatasskelsoae* sp. nov. non-type ♂ (VTEC, MPE04021) coloration. Scale bar: 4.0 mm.

Figs 50 , 51 Vernacular name: “Tass Kelso’s Twisted-Claw Millipede”

####### Material examined.

***Holotype*:** United States – **West Virginia** • ♂; Greenbrier Co., Monongahela National Forest, Summit Lake Campground; 38.2490°N, -80.4437°W; elev. 1062 m; 20 July 2005; hand collected; P. Marek, C. Spruill leg.; VTEC SPC000710.

***Paratypes*:** United States – **West Virginia** • 1 ♂; same collection data as holotype; FMNH SPC000711 • 1 ♀; same collection data as holotype; VTEC SPC000712 • 1 ♀; same collection data as holotype; VMNH SPC000713 • 1 ♂; Pocahontas Co., Droop Mountain Battlefield State Park, near Little Levels between cemetery, lookout, and park area; 38.1101°N, -80.2733°W; elev. 942 m; 1 June 2015; hand collected; C. Hall leg.; VTEC MPE00578 • 1 ♀; same collection data as preceding; VTEC MPE00580.

####### Other material.

United States – **West Virginia** • 4 ♂♂; Greenbrier County, Summit Lake Campground, small swift stream hollow downhill from campsite #20; 38.2498°N, -80.4455°W; elev. 1056 m; 19 May 2018; hand collected; C. Harden, G. Chapman leg.; VTEC MPE04022, 23, 31, 32 • 5 ♂♂; Greenbrier County, Kate’s Mountain summit, Greenbrier State Forest; 37.7374°N, -80.3324°W; 20 Apr. 1971; W. Shear leg.; VMNH NAN0042 • 1 ♂; Greenbrier County, Second Ck, 1 mi E, 1.4 mi S Ft. Spring; 37.7162°N, -80.5388°W; 12 Oct. 2000; W. Arnold leg.; VMNH NAN0079 • 1 ♂; Greenbrier County, White Sulfur Springs, Kate’s Mountain summit; 37.7622°N, -80.3013°W; 20 Apr. 1968; W. Shear leg.; VMNH NAN0082 • 1 ♂; Monroe County, Laurel Creek Cave, Greenville; 37.5440°N, -80.6818°W; 16 Apr. 1972; W. Shear leg.; VMNH NAN0040 • 1 ♂; Nicholas County, Monongahela NF, Woodbine Picnic Area; 38.2897°N, -80.5353°W; elev. 646 m; 10 May 2011; Ashley Bailey leg.; VTEC SPC001100 • 1 ♀; Nicholas County, Monongahela NF, FR-76 (E side), 5.15 rd km N WV-39/55; 38.2728°N, -80.5250°W; elev. 806 m; 20 July 2005; hand collected; P. Marek, C. Spruill leg.; VTEC SPC000715 • 1 ♂; Pocahontas County, Kennison Mountain Trail, ca.0.5 mi from trailhead on Hwy 39; 38.1906°N, -80.2858°W; elev. 1261 m; 19 May 2018; hand collected; C. Harden, G. Chapman leg.; VTEC MPE04021 • 1 ♀; Pocahontas County, Watoga State Park, Dragon Draft Trail; 38.1190°N, -80.1540°W; elev. 739 m; 18 July 2005; hand collected; P. Marek, C. Spruill leg.; VTEC SPC000689 • 2 ♀♀; Pocahontas County, Watoga State Park, Riverside Campground; 38.1119°N, -80.1783°W; elev. 636 m; 19 July 2005; hand collected; P. Marek, C. Spruill leg.; VTEC SPC000694, 695 • 1 ♂; Pocahontas County, Cranberry Glades Natural Area, Monongahela Nat. Forest; 38.2000°N, -80.2716°W; 20 May 1967; W. Shear leg.; VMNH NAN0016 • 1 ♂; Pocahontas County, Hills Creek Falls, Monongahela Nat. Forest; oak-hickory-beech-maple transition; 38.1761°N, -80.3365°W; 20 May 1967; W. Shear leg.; VMNH NAN0021 • 2 ♂♂; same collection data as preceding; 8 July 1967; W. Shear leg.; VMNH NAN0022 • 1 ♂; same collection data as preceding; 18 May 1968; W. Shear leg.; VMNH NAN0023 • 1 ♂; same collection data as preceding; 24 Sep. 1972; W. Shear leg.; VMNH NAN0039 • 2 ♂♂; same collection data as preceding; 8 May 1971; W. Shear leg.; VMNH NAN0044 • 10 ♂♂; same collection data as preceding; 5 May 1970; VMNH NAN0078 • 1 ♂; Pocahontas County, Droop Mtn State Park; 38.1150°N, -80.2694°W; 30 Apr. 1967; W. Shear leg.; VMNH NAN0024 • 1 ♀; Randolph County, Kumbrabow State Forest, off Kumbrabow Rd. near Mill Creek; 38.6550°N, -80.0713°W; elev. 922 m; 1 June 2015; hand collected; C. Hall leg.; VTEC MPE00585 • 1 ♀; Randolph County, Gatewood CpGrnd; 37.7622°N, -80.3013°W; elev. 1322 m; 20 Apr. 1968; W. Shear leg.; VMNH NAN0080 • 1 ♂; Randolph County, between 1–2 miles W of Bear Heaven Picnic Area on road to Bickle’s Knob nr. Alpena; 38.9367°N, -79.7029°W; 12 June 1986; Highton, Barry leg.; VMNH NAN0152 • 3 ♂♂; Randolph County, along US 33, 2.1 W Alpena; 38.9101°N, -79.6900°W; 23 Aug. 1978; R. Shelley, C. Withrow leg.; NCSM NAN0486 • 3 ♂♂; Summers County, 9 miles SE of Bellepoint on W. Va. Hy. 12; 37.5385°N, -80.7897°W; 22 Sep. 1962; R. Hoffman leg.; VMNH NAN0149 • 1 ♂; Tucker County, Backbone Mtn.; 39.1470°N, -79.5700°W; elev. 1067 m; 30 June 1968; W. Muchmore leg.; VMNH NAN0148 • For detailed collection data see Suppl. material 7.

####### Diagnosis.

Adult males of *Nannariatasskelsoae* sp. nov. are distinct from other *Nannaria* and the sympatric *N.shenandoa*, based on the following combination of characters: ***Gonopods*.** Gonopodal acropodite gently curving medially before apex, not straight as in *N.shenandoa*. Distal zone short, bent medially forming 130° angle with acropodite (Fig. 50). Telopodite basal zone ca. 1/3 length of acropodite, not < 1/3 as in *N.fowleri* and *N.shenandoa*. Acropodite tip and distal zone simple, rectangular, blunt, < 1/8 length of acropodite, not large, curving with flange as in *N.shenandoa*. Prefemoral process arising from top of prefemoral spine (Fig. 50A, red arrow), paralleling medial curve of acropodite, not arising from bottom of prefemoral spine as in *N.fowleri*, or curving laterally as in *N.shenandoa*. Prefemoral spine large, curving cephalically, not stout, tooth-like as in *N.fowleri*. ***Color*.** Tergites with orange paranotal spots (Fig. 51). Black background. Dorsum of collum smooth with orange margin.

####### Measurements.

♂ holotype (VTEC, SPC000710): BL = 30.5, CW = 3.9, IW = 2.4, ISW = 0.9, B11W = 5.0, B11H = 3.3; ♀ paratype (VTEC, SPC000712): BL = 34.4, CW = 4.3, IW = 2.2, ISW = 1.1, B11W = 5.3, B11H, 4.1.

####### Variation.

The most notable variation amongst individuals of *N.tasskelsoae* sp. nov. is found in the southern range of the species’ distribution. One specimen from Greenville, West Virginia (NAN0040) has a sudden lateral curve in the tip of its prefemoral process, forming a cat-claw like bend, while another specimen from near Forest Hill, West Virginia (NAN0149) has a laminate medial flange that is distal to the acropodite apex. Additional variation between populations is minimal, and is primarily found in the degree to which the prefemoral process parallels the acropodite and the size of the prefemoral spine.

####### Distribution.

*Nannariatasskelsoae* sp. nov. has a linear distribution running the length of the eastern edge of West Virginia (West Virginia: Randolph, Nicholas, Greenbrier, Monroe, Pocahontas, Summers, and Tucker counties; Suppl. material 7; Fig. 127). Distribution area: 3,623 km^2^; status: SRE.

####### Ecology.

Specimens of *N.tasskelsoae* sp. nov. were collected from mesic hardwood forests composed of beech, black cherry, oak, sugar maple, tuliptree, and yellow birch. Several specimens were found on hillsides, including those from Summit Lake Campground which were active on top of leaf litter at night.

####### Etymology.

This species is named after Dr. Tass Kelso, systematic botanist. Dr. Kelso was mentor to the discoverer of this species, Charity Hall. The specific name is a genitive noun derived as a matronym.

####### Type locality.

United States, West Virginia, Greenbrier Co., Monongahela National Forest, Summit Lake Campground; 38.2490°N, -80.4437°W.

##### *tennesseensis* clade

**Components.***Nannariabreweri* sp. nov., *N.equalis* Chamberlin, 1949, *N.fritzae* sp. nov., *N.monsdomia* sp. nov., *N.tennesseensis*, and a female specimen from Dungannon, Virginia. The *tennesseensis* clade represents one of the more heterogeneous *Nannaria* clades, with a variety of gonopod morphologies and a widespread geographic distribution, including the most southern species of the *minor* group, *N.fritzae* sp. nov. There are a few generally shared gonopodal characters, however, including a blunt acropodite tip and a medially directed prefemoral process, with the exception of *N.monsdomia* sp. nov. and *N.equalis* (Fig. 113). No specimens of *N.equalis* were collected for this study, however due to close geographic proximity and shared gonopodal characters, such as a blunt acropodite tip and a prominent prefemoral spine (as seen in *N.monsdomia* sp. nov.), we place *N.equalis* within the *tennesseensis* clade.

**Distribution.** The *tennesseensis* clade extends from northwestern Georgia, into eastern Tennessee, and southwestern Virginia (Fig. 114).

###### Nannaria breweri sp. nov.

Taxon classificationAnimaliaPolydesmidaXystodesmidae

322087E4-CCE4-5F21-A3EA-44431481F35A

http://zoobank.org/6B041BC2-5674-4133-993F-FFCDCAC54252

**Figure 52.**
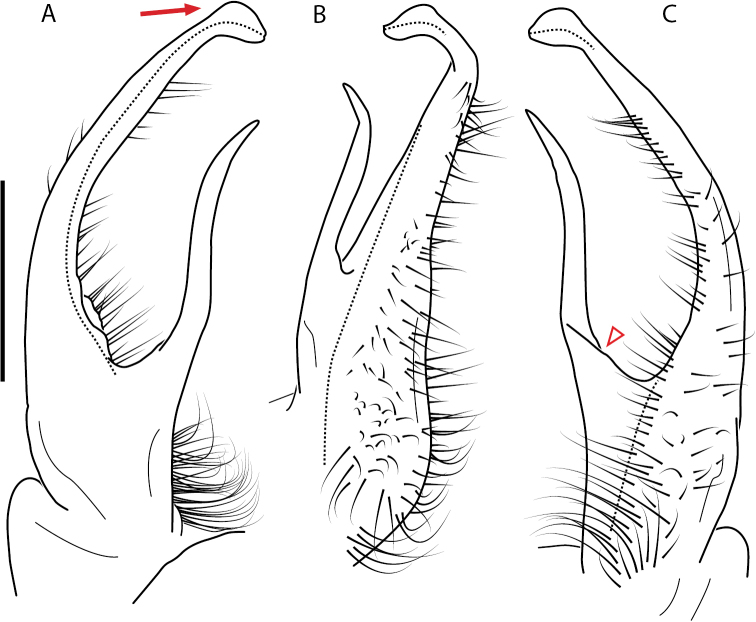
*Nannariabreweri* sp. nov. holotype ♂ (VTEC, MPE02191) left gonopod **A** anterior view; red arrow indicates lateral flange **B** medial view **C** posterior view; red triangle indicates prefemoral spine fused to the prefemoral process. Scale bar: 0.5 mm.

**Figure 53.**
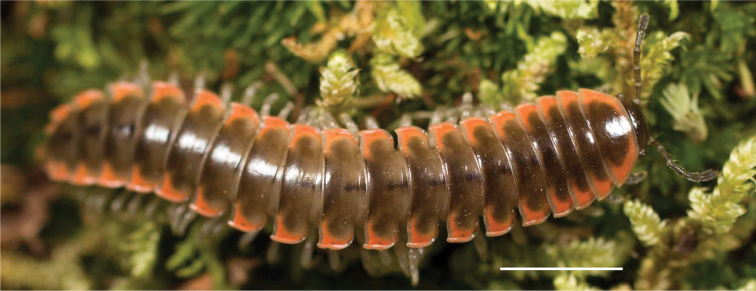
*Nannariabreweri* sp. nov. holotype ♂ (VTEC, MPE02191) coloration. Scale bar: 4.0 mm.

Figs 52 , 53 Vernacular name: “Michael Brewer’s Twisted-Claw Millipede”

####### Material examined.

***Holotype*:** United States – **Tennessee** • ♂; Hamblen County, Morristown, Panther Creek State Park, in gully below parking lot at Spoone Recreation Area, near Ore Mine Trail; 36.2167°N, -83.4055°W; elev. 373 m; 9 Oct. 2016; hand collected; J. Means and D. Hennen leg.; VTEC MPE02191.

***Paratypes*:** United States – **Tennessee** • 1 ♂; same collection data as for holotype; VTEC MPE02198 • 2 ♂♂; same collection data as for holotype; VMNH MPE02205, 3730 • 5 ♀♀; same collection data as for holotype; VTEC MPE02197, 2202–4, 3719 • 4 ♀♀; same collection data as for holotype; VMNH MPE03720, 3731–3.

####### Other material.

United States – **Tennessee** • 1 ♂; Grainger County, 6.8 miles S of Rutledge; 36.1822°N, -83.5150°W; 18 May 1956; Lund, Keeton, R. Hoffman leg.; VMNH NAN0324 • 2 ♀♀; Hamblen County, Panther Creek State Park, Seven Sinkholes Trail; 36.2160°N, -83.4057°W; elev. 343 m; 14 Oct. 2007; hand collected; P. Marek, L. Swafford, M. Brewer, C. Hall, K. Bader leg; VTEC SPC001167, 1179 • 5 ♂♂; Hamblen County, Cherokee Lake Bluff, 3.5 miles N of Morristown; 36.2646°N, -83.2950°W; 19 May 1961; L. Hubricht leg.; VMNH NAN0309; SCAU – **Virginia** • 1 ♂; Tazewell County, Burkes Garden, on Garden Mountain; 37.0809°N, -81.4145°W; elev. 1158 m; 27 Oct. 1970; R. Hoffman leg.; VMNH NAN0001 • 1 ♂; same collection data as preceding; 16 Oct. 1966; A. Q. Field Trip leg; VMNH NAN0004 • 1 ♂; same collection data as preceding; 14 Apr. 1965; Herp Class leg.; VMNH NAN0008 • Tazewell County, Burkes Garden, Station Spring Creek; 37.0809°N, -81.4145°W; elev. 1219 m; 23 Aug. 1981; R. Hoffman leg.; VMNH NAN0006 • 1 ♂; Tazewell County, Burkes Garden, NW slope Beartown Mountain; 36.9361°N, -81.8861°W; elev. 1341 m; 12 July 1971; R. Hoffman leg.; VMNH NAN0007 • Tazewell County, Burkes Garden; 37.0980°N, -81.3411°W; 20 March 1954; R. Hoffman leg.; VMNH, NAN0009 • 2 ♂♂; same collection data as preceding; 30 March 1954; VMNH NAN0070 • 2 ♂♂; Tazewell County, Burkes Garden, Cassell Farm; 37.0980°N, -81.3411°W; 26 April 1981; R. Hoffman leg.; VMNH NAN0142 • SCAU – **West Virginia** • 1 ♂; Mercer County, Concord College campus; 37.4245°N, -81.0060°W; 14 Nov. 1966; W. Shear leg.; VMNH NAN0036. For detailed collection data see Suppl. material 7.

####### Diagnosis.

Adult males of *Nannariabreweri* sp. nov. are distinct from other *Nannaria* and the nearby *N.scutellaria* Causey, 1942, based on the following combination of characters: ***Gonopods*.** Acropodite curving medially throughout, not sinuous, only curving slightly medially as in *N.tennesseensis* and not straight, with 90° medial bend at apex as in *N.scutellaria*. Distal zone with large, lobed lateral flange (Fig. 52A, red arrow), not with small, triangular lateral flange as in *N.tennesseensis*, or medial flange as in *N.scutellaria*. Tip directed medially, not cephalically as in *N.tennesseensis*. Telopodite basal zone height < 1/2 length of acropodite, not > 1/2 as in *N.tennesseensis* or ca. 1/4 length as in *N.scutellaria*. Prefemur with medially curving prefemoral process, not straight as in *N.tennesseensis*. Prefemoral tip bent dorsally (Fig. 52B). Prefemoral spine fused to prefemoral process throughout length, forming ridge along base of prefemoral process (Fig. 52C, red triangle). ***Color*.** Tergites with orange paranotal spots (Fig. 53). Brown background. Collum smooth with orange margin.

####### Measurements.

♂ holotype (VTEC, MPE02191): BL = 29.9, CW = 4.8, IW = 2.3, ISW = 0.9, B11W = 5.4, B11H = 3.6; ♀ paratype (VTEC, MPE02203): BL = 35.9, CW = 4.5, IW = 2.5, ISW = 1.0, B11W = 5.8, B11H = 4.4.

####### Variation.

No known variation.

####### Distribution.

Known from a linear area from southern West Virginia to northeastern Tennessee (Tennessee: Grainger and Hamblen counties; West Virginia: Mercer County; Virginia: Russell and Tazewell counties, Suppl. material 7; Fig. 126). Distribution area: 635 km^2^; status: MRE.

####### Ecology.

Individuals of *N.breweri* sp. nov. have been collected from mesic, broadleaved forests composed of pawpaw, maple, spicebush, oak, and elm, often under deciduous leaf litter and/or beneath 1–2 cm dark soil.

####### Etymology.

This species is named for its co-collector and West Virginian, Dr. Michael Brewer (Suppl. material 7). The specific name is a genitive noun derived as a patronym.

####### Type locality.

United States, Tennessee, Hamblen County, Morristown, Panther Creek State Park, in gully below parking lot at Spoone Recreation Area, near Ore Mine Trail, 36.2167°N, -83.4055°W.

###### Nannaria equalis

Taxon classificationAnimaliaPolydesmidaXystodesmidae

Chamberlin, 1949

18A65F98-3CB7-56BC-8C5E-1E693A77AEAE

**Figure 54.**
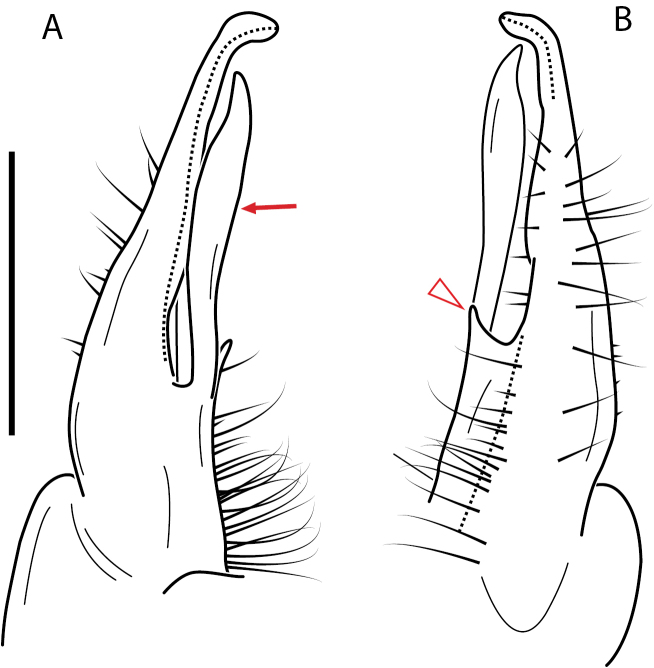
*Nannariaequalis* ♂ (VMNH, NAN0335) left gonopod **A** anterior view; red arrow indicates large, straight prefemoral process **B** posterior view; red triangle indicates sharp prefemoral spine. Scale bar: 0.5 mm.

Fig. 54 Vernacular name: “The Knoxville Twisted-Claw Millipede”

Nannaria equalis Chamberlin, 1949: 4, fig. 4. Chamberlin and Hoffman 1958: 40. Hoffman 1999: 366. Marek et al. 2014: 37. Means et al. 2021: S69–S70.

####### Material examined.

United States – **Tennessee** • 1 ♂; Knox County, site 2 mesic cove hardwoods; from label: 17.233902E 3993447N; 10 May 2005; J. Sevier leg.; VMNH NAN0335. For detailed collection data see Suppl. material 7.

####### Diagnosis.

Adult males of *N.equalis* are distinct from other *Nannaria* and the nearby *N.monsdomia* sp. nov. and *N.scutellaria* based on the following combination of characters: ***Gonopods*.** Gonopodal acropodite straight before apex, not slightly curving before apex as in *N.monsdomia* sp. nov. Distal zone much reduced, rounded, simple, not with small, lobed lateral flange as in *N.monsdomia* sp. nov., or bent at 90° with pronounced medial flange as in *N.scutellaria*. Acropodite with medial swelling, lacking in *N.scutellaria*. Telopodite basal zone height ca. 1/2 length of acropodite, not > 2/3 length as in *N.monsdomia* sp. nov. or < 1/3 length as in *N.scutellaria*. Prefemur with large, straight prefemoral process (Fig. 54A, red arrow), not curving medially, crossing acropodite, and expanding before tip, as in *N.monsdomia* sp. nov., or curving laterally as in *N.scutellaria*. Prefemoral spine sharp, curving laterally (Fig. 54B, red triangle), not widely separated from prefemoral process and rounded, as in *N.monsdomia* sp. nov., or lacking as in *N.scutellaria*. Acropodite appearing as straight line in medial view, therefore the medial view was not illustrated. ***Color*.** Chamberlin reports that the paranota of *Nannariaequalis* are yellow in color, though this would be unique in the *Nannaria* and has not been confirmed by the authors. It is likely Chamberlin was referring to a preserved specimen whose color had faded in alcohol.

**Figure 55.**
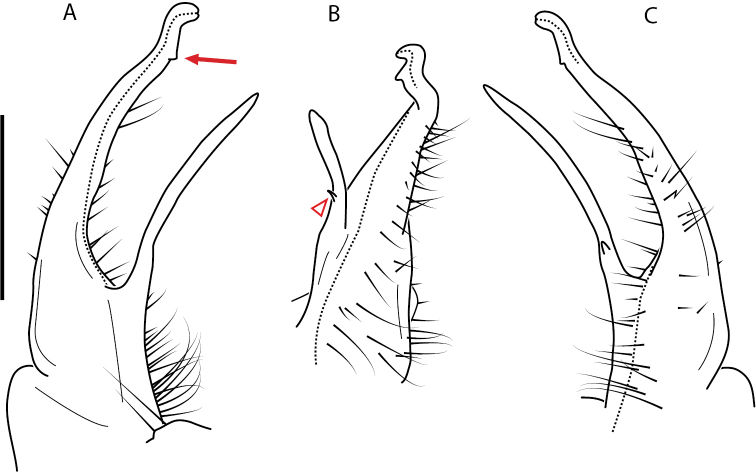
*Nannariafritzae* sp. nov. ♂ holotype (VTEC, MPE02359) left gonopod **A** anterior view; red arrow indicates tooth-like medial flange **B** medial view; red triangle indicates small, thorn-like prefemoral spine **C** posterior view. Scale bar: 0.5 mm.

####### Measurements.

♂ (VMNH, NAN0335): BL = 27.2, CW = 4.0, IW = 1.7, ISW = 0.9, B11W = 4.7, B11H = 2.8.

####### Variation.

No known variation.

####### Distribution.

Known only from the type locality (Tennessee: Knox County; Suppl. material 7; Fig. 126). Distribution area: N/A; status: MRE.

####### Ecology.

Chamberlin (1949) gives no information on the ecology of *N.equalis*.

####### Etymology.

Chamberlin (1949) provided no etymology for the name *equalis*, though it is reasonable to assume that it is in reference to the prefemoral process nearly equaling in length the acropodite, as Chamberlin mentions in his description.

####### Type locality.

United States, Tennessee, Knox County, Knoxville.

####### Notes.

In the original publication Chamberlin (1949: 4) designated a male holotype and female paratype (originally as an “allotype”).

###### Nannaria fritzae sp. nov.

Taxon classificationAnimaliaPolydesmidaXystodesmidae

834069AA-15D9-5AD5-BD10-A6938565EB9F

http://zoobank.org/735CBEDE-9F71-4971-9601-BCE0ADF3F53B

**Figure 56.**
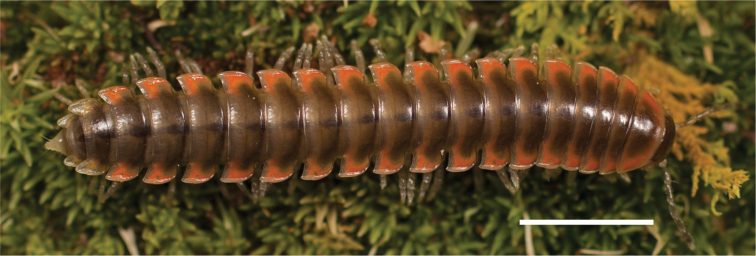
*Nannariafritzae* sp. nov. ♂ holotype (VTEC, MPE02359) coloration. Scale bar: 4.0 mm.

Figs 55 , 56 Vernacular name: “Kathlyn Fritz’s Twisted-Claw Millipede”

####### Material examined.

***Holotype*:** United States – **Georgia** • ♂ Chattooga County, hillside by Lake Marvin Rd.; 34.5628°N, -85.0681°W; elev. 385 m; 5 Mar. 2017; hand collected; J. Means, K. Means leg.; VTEC MPE02359.

***Paratypes*:** United States – **Georgia** • 1 ♂; same collection data as holotype; VTEC MPE02377 • 1 ♂; same collection data as holotype; VMNH MPE02378.

####### Diagnosis.

Adult males of *Nannariafritzae* sp. nov. are distinct from other *Nannaria* and the nearby *Nannaria* sp. nov. ‘Amicolola’ (*wilsoni* species group) based on the following combination of characters. ***Gonopods*.** Gonopodal acropodite gently curving medially throughout, not straight with abrupt medial bend at apex as in *Nannaria* sp. nov. ‘Amicolola.’ Distal zone short, rounded, not serpentine as in *Nannaria* sp. nov. ‘Amicolola.’ Acropodite with small, tooth-like medial flange near apex (Fig. 55A, red arrow). Prefemur with long acicular prefemoral process bent at base and directed medially—not curving laterally, crossing acropodite, as in *N.minor* or not large, laminate as in *Nannaria* sp. nov. ‘Amicolola.’ Prefemoral spine reduced to thorn-like structure, arising from prefemoral process, directed ventrally (Fig. 55B, red triangle)—not curving cephalically as in *N.minor*. Telopodite basal zone ca. 1/2 length of acropodite, not < 1/2 as in *N.minor* or > 1/4 as in *Nannaria* sp. nov. ‘Amicolola.’ ***Color*.** Tergites with orange paranotal spots (Fig. 56), reduced pigmentation of paranotal spots of terminal segments. Light brown background. Dorsum of collum smooth with orange and white margin.

####### Measurements.

♂ holotype (VTEC, MPE02359): BL = 28.2, CW = 3.8, IW = 2.3, ISW = 0.8, B11W = 4.6, B11H = 3.0.

####### Variation.

No known variation.

####### Distribution.

Known only from the type locality (Georgia: Chattooga County; Suppl. material 7; Fig. 126). Distribution area: N/A; status: MRE.

####### Ecology.

Individuals of *N.fritzae* sp. nov. were collected on a hillside in a mesic hardwood forest dominated by oak and maple. Specimens were found under leaf litter in a dry creek bed near the base of an oak tree.

####### Etymology.

This species is named for its co-collector, Kathlyn Fritz Means. The name is a genitive noun derived as a matronym.

####### Type locality.

United States, Georgia, Chattooga County, hillside by Lake Marvin Rd.; 34.5628°N, -85.0681°W.

###### Nannaria monsdomia sp. nov.

Taxon classificationAnimaliaPolydesmidaXystodesmidae

CDE5B836-9982-5E33-834C-48D4BAFDF8E1

http://zoobank.org/B78C1537-424E-42E7-9100-9385966C69D9

**Figure 57.**
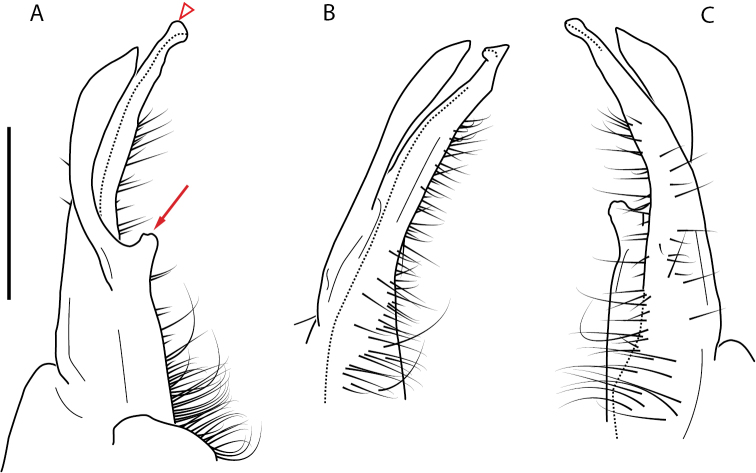
*Nannariamonsdomia* sp. nov. holotype ♂ (VTEC, MPE02188) left gonopod **A** anterior view; red triangle indicates small lateral flange on tip; red arrow indicates wide separation between prefemoral process and rounded prefemoral spine **B** medial view **C** posterior view. Scale bar: 0.5 mm.

**Figure 58.**
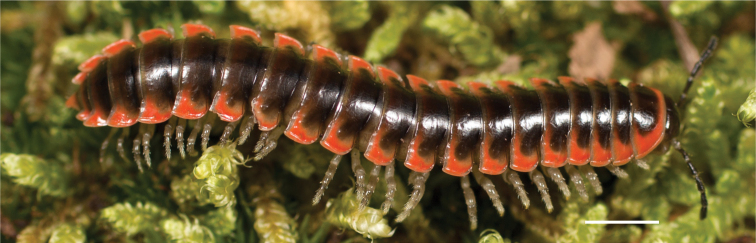
*Nannariamonsdomia* sp. nov. holotype ♂ (VTEC, MPE02188) coloration. Scale bar: 4.0 mm.

Figs 57 , 58 Vernacular name: “The House Mountain Twisted-Claw Millipede”

####### Material examined.

***Holotype*:** United States – **Tennessee** • ♂; Knox County, Mascot, House Mountain State Natural Area, along dried up stream beside Hogskin Rd.; 36.1032°N, -83.7642°W; elev. 357 m; 8 Oct. 2016; hand collected; J. Means, D. Hennen leg. VTEC MPE02188.

***Paratypes*:** United States – **Tennessee** • 2 ♂♂; same collection data as holotype; VTEC MPE02186, 2199 • 2 ♂; same collection data as holotype; VMNH MPE02201, 3712 • 2 ♀♀; same collection data as holotype; VTEC MPE02187, 2189 • 2 ♀♀; same collection data as holotype; VMNH MPE02196, 2200 • 1 ♂; Knox County, Mascot, House Mountain State Natural Area, under large strips of oak bark on top of soil at top of hill; 36.1043°N, -83.7633°W; elev. 360 m; 8 Oct. 2016; hand collected; J. Means, D. Hennen leg.; VMNH MPE03677 • 2 ♀♀; same collection data as preceding; VMNH MPE02180, 2192 For detailed collection data see Suppl. material 7.

####### Diagnosis.

Adult males of *Nannariamonsdomia* sp. nov. are distinct from other *Nannaria* and the nearby *N.scutellaria*, based on the following combination of characters. ***Gonopods*.** Gonopodal acropodite slightly curving medially basal to apex, not straight basal to apex as in *N.scutellaria*. Distal zone much reduced, rounded, with small, lobed lateral flange (Fig. 57A, red triangle)—not simple as in *N.hardeni* sp. nov., or bent at 90° with pronounced medial flange as in *N.scutellaria*. Telopodite basal zone height > 2/3 length of acropodite, not < 1/3 length as in *N.scutellaria*. Prefemur with large, laminate prefemoral process, curving medially, crossing acropodite and expanding before tip, not straight, acicular as in *N.hardeni* sp. nov., or curving laterally as in *N.scutellaria*. Prefemoral process length greater than height of telopodite basal zone, not subequal as in *N.hardeni* sp. nov. Prefemoral spine widely separated from prefemoral process (Fig. 57A, red arrow) and rounded, not sharp or tooth-like as in *N.hardeni* sp. nov., or lacking as in *N.scutellaria*. ***Color*.** Tergites with bright orange paranotal spots and faint orange stripes (Fig. 58). Dark brown background. Dorsum of collum smooth with orange margin.

####### Measurements.

♂ holotype (VTEC, MPE02188): BL = 30.7, CW = 4.2, IW = 2.2, ISW = 0.9, B11W = 4.2, B11H = 3.1; ♀ paratype (VTEC, MPE02187): BL = 33.5, CW = 4.1, IW = 2.5, ISW = 1.1, B11W = 5.3, B11H = 3.9.

####### Variation.

No known variation.

####### Distribution.

Known only from the type locality, Suppl. material 7; Fig. 126. Distribution area: N/A; status: MRE.

####### Ecology.

Individuals of *N.monsdomia* sp. nov. were collected from a mesic hardwood forest dominated by beech, maple, magnolia, pawpaw, and poison ivy. All specimens were found under 1–4 cm of hardpacked soil at the edge of boulders near a dried up, rocky stream.

####### Etymology.

The specific epithet is an arbitrary combination of letters from the Latin *mons*, meaning mountain, and *domus*, meaning house, which refers to the type locality House Mountain State Natural Area. It is to be treated as a noun in apposition.

####### Type locality.

United States, Tennessee, Knox County, Mascot, House Mountain State Natural Area, along dried up stream beside Hogskin Rd., 36.1032°N, -83.7642°W.

###### Nannaria tennesseensis

Taxon classificationAnimaliaPolydesmidaXystodesmidae

(Bollman, 1888)

2B87C0A9-6887-5748-81C9-1ED64CB72686

**Figure 59.**
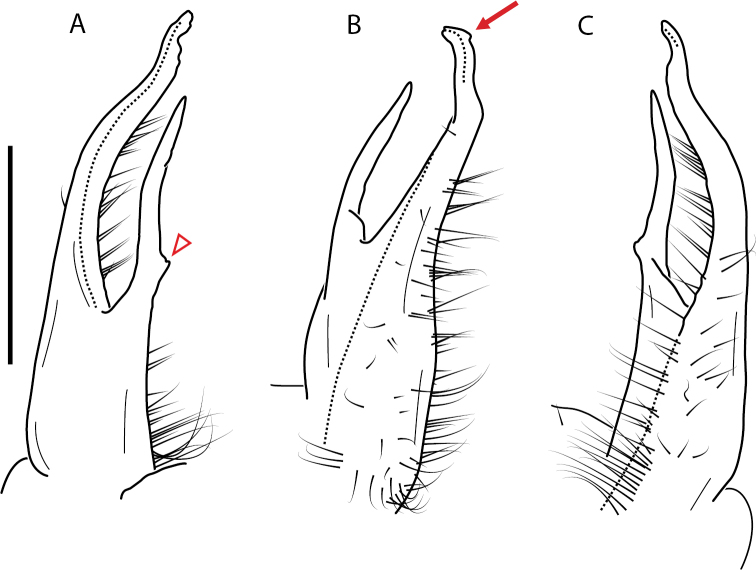
*Nannariatennesseensis* ♂ (VTEC, MPE01245) left gonopod **A** anterior view; red triangle indicates prefemoral spine fused to the prefemoral process **B** medial view; red arrow indicates lateral flange **C** posterior view Scale bar: 0.5 mm.

**Figure 60.**
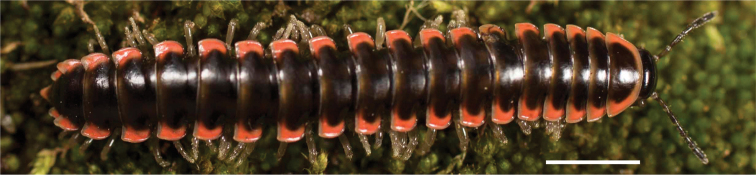
*Nannariatennesseensis* ♂ (VTEC, MPE01245) coloration. Scale bar: 4.0 mm.

Figs 59 , 60 Vernacular name: “The Tennessee Twisted-Claw Millipede”

Fontaria tennesseensis Bollman, 1888: 340. Bollman 1893: 91. Attems 1898: 263. Attems 1938: 199.
Nannaria tennesseensis : Chamberlin and Hoffman 1958: 42. Hoffman 1999: 368. Marek et al. 2014: 38. Means et al. 2021: S73.

####### Material examined.

***Syntype*:** United States – **Tennessee** • ♀; Jefferson County, Mossy Creek; C. Branner leg.; NMNH No. 203.

####### Other material.

United States – **Tennessee** • 1 ♂; Blount County, on dirt rd. off 2422 on Blount-Sevier co. line, 14 E Maryville; 35.7450°N, -83.7188°W; 11 Oct. 1978; R. Shelley, W. Jones leg.; NCSM NAN0484 • 6 ♂♂; Blount County, GSMNP, Hwy 129/Lake Chilhowee Site 2; 35.5472°N, -84.01277°W; 14 Oct. 2006; hand collected; B. Snyder leg.; Bruce Snyder Pers. Coll., B+3, B+9, B+15 • 2 ♂♂ and 1 ♀; same collection data as preceding; 27 October 2006; Bruce Snyder Pers. Coll. • 1 ♂; Jefferson County, Cherokee Dam Campground, roped off road (gravel) next to parking lot by tent camping; 36.1515°N, -83.5170°W; elev. 345 m; 24 May 2016; hand collected; J. Means, D. Hennen leg.; VTEC MPE01237 • 2 ♀♀; same collection data as preceding; VTEC MPE01315, 131 • 1 ♂; Jefferson County, Mossy Creek Wildlife Viewing Area, south end of Cherokee Reservoir, followed road past parking area, turned into a dirt road; 36.1301°N, -83.5056°W; elev. 336 m; 25 May 2016; hand collected; J. Means, D. Hennen leg.; VTEC MPE01245 • 3 ♀♀; same collection data as preceding; VTEC MPE01290–92 • 1 ♂; Jefferson County, on unnumb. rd. off US 411, 16 air mi SE Jefferson City; 35.9543°N, -83.2928°W; 10 Oct. 1978; R. Shelley, W. Jones leg.; NCSM NAN0471 • 1 ♀; Sevier County, Great Smoky Mountains National Park, Ramsey Cascades Trail; 35.7032°N, -83.3536°W; elev. 691 m; 24 Apr. 2019; hand collected; D. Hennen leg.; VTEC MPE04825 • 2 ♂♂; Sevier County, Great Smoky Mountains National Park, forest beside Greenbrier Rd.; 35.7257°N, -83.4020°W; elev. 471 m; 26 Apr. 2019; hand collected; D. Hennen leg.; VTEC MPE04812, 4828 • 3 ♀♀; same collection data as preceding; VTEC MPE04813–15. For detailed collection data see Suppl. material 7.

####### Diagnosis.

Adult males of *Nannariatennesseensis* are distinct from other *Nannaria* and the nearby species *N.scutellaria*, based on the following combination of characters: ***Gonopods*.** Gonopodal acropodite sinuous, curving slightly medially, not curving medially as in *N.breweri* sp. nov. and not straight with 90° medial bend at apex as in *N.scutellaria*. Distal zone heavily reduced, with small triangular lateral flange (Fig. 59B, red arrow)—without large lobed lateral flange as in *N.breweri* sp. nov., or medial flange as in *N.scutellaria*. Acropodite tip directed cephalically, not medially as in *N.breweri* sp. nov. or *N.scutellaria*. Telopodite basal zone long, > 1/2 length of acropodite, not < 1/2 as in *N.breweri* sp. nov. or ca. 1/4 length as in *N.scutellaria*. Prefemur with straight prefemoral process, not medially curving as in *N.breweri* sp. nov. Prefemoral spine fused to prefemoral process throughout length (Fig. 59A, red triangle). ***Color*.** Tergites with sherbet orange paranotal spots (Fig. 60). Brown background. Dorsum of collum smooth with orange and white caudal margin.

####### Measurements.

♂ (VTEC, MPE01245): BL = 29.2, CW = 3.2, IW = 1.8, ISW = 0.7, B11W = 4.2, B11H = 2.5; ♀ (VTEC, MPE01291): BL = 34.8, CW = 3.9. IW = 2.5, ISW = 1.2, B11W = 4.7. B11H = 3.5.

####### Variation.

No known variation.

####### Distribution.

Known from eastern Tennessee in the vicinities of Jefferson City and the Great Smoky Mountains National Park (Tennessee: Blount, Jefferson, and Sevier counties; Suppl. material 7; Fig. 126). Distribution area: 1,440 km^2^; status: SRE.

####### Ecology.

Individuals of *N.tennesseensis* have been collected from a variety of habitats, including mesic mixed hemlock and hardwood forests primarily composed of oak, maple, tuliptree, hemlock, and rhododendron, and semi-xeric hardwood forests composed of oak and maple. In the former habitats, specimens were often found under leaf litter and/or under 1–2 cm of dark, loose soil; in the latter habitats, specimens were found walking on top of hard packed soil, under thin oak leaf litter.

####### Etymology.

Bollman (1888) gave no etymology for the name *tennesseensis*, but it is reasonable to assume that the specific name refers to the state in which it is found.

####### Type locality.

United States, Tennessee, Jefferson County, Mossy Creek.

####### Notes.

In the original publication Bollman (1888: 340) inspected two lots of specimens, Nos. 203 & 388. No. 203 contained just one specimen, a female syntype, while No. 388 consisted of five specimens, two males and three females. Bollman (1888: 340) notes that he deposited two of the lot No. 388 specimens at the USNM (now NMNH) though he does not mention the sex of these specimens. Bollman (1888: 340) mentioned two “types”, one from each of the lots. The type from lot No. 388 was deposited in Bollman’s private collection, and its current whereabouts are unknown. Both lots were collected by C. Branner on unspecified dates.

##### *ignis* clade

**Components.***Nannariaignis* sp. nov., *N.tenuis* sp. nov., and a female from Mercer Co., West Virginia (Fig. 113). Members of the *ignis* clade share gonopodal characters, including a wide division between the prefemoral process and acropodite, and a small lateral flange near the acropodite apex. Beyond these characters, however, there is a high degree of variability in the shape and curve of the prefemoral process and the width of the trunk. Additionally, the members of the *ignis* clade occupy a very small geographic range, ~ 500 square km. This suggests that this clade either represents a closely related group of species which have not dispersed widely or one isolated, morphologically variable species which may be in the process of speciating. Based on a combination of morphological and molecular evidence, we feel confident in naming *N.ignis* sp. nov. and *N.tenuis* sp. nov. as separate species, however additional sampling, including in and around Mercer Co., West Virginia, will help elucidate relationships within the *ignis* clade.

**Distribution.** the *ignis* clade extends from southeastern West Virginia into southwestern Virginia (Fig. 114).

###### Nannaria ignis sp. nov.

Taxon classificationAnimaliaPolydesmidaXystodesmidae

C435A97F-2AFD-5B69-AD23-3D71411F8197

http://zoobank.org/12052629-9913-403E-BE70-4EB35E719A9E

**Figure 61.**
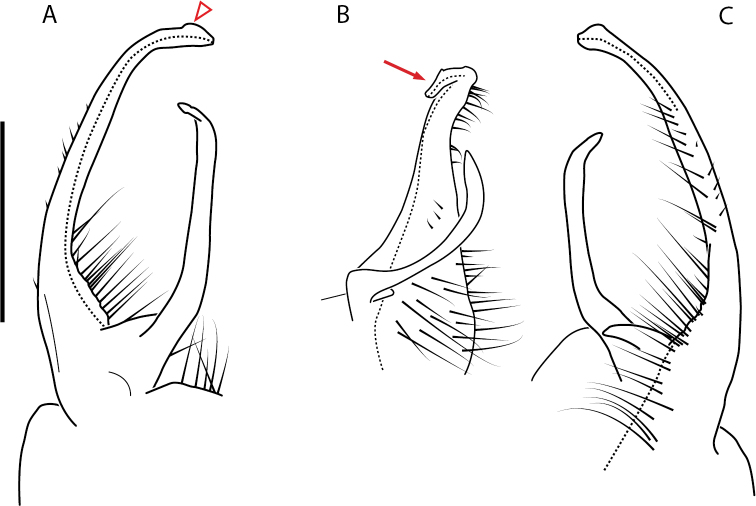
*Nannariaignis* sp. nov. holotype ♂ (VTEC, MPE01063) left gonopod **A** anterior view; red triangle indicates small lateral flange **B** medial view; red arrow indicates dorsomedially curving acropodite tip **C** posterior view. Scale bar: 0.5 mm.

**Figure 62.**
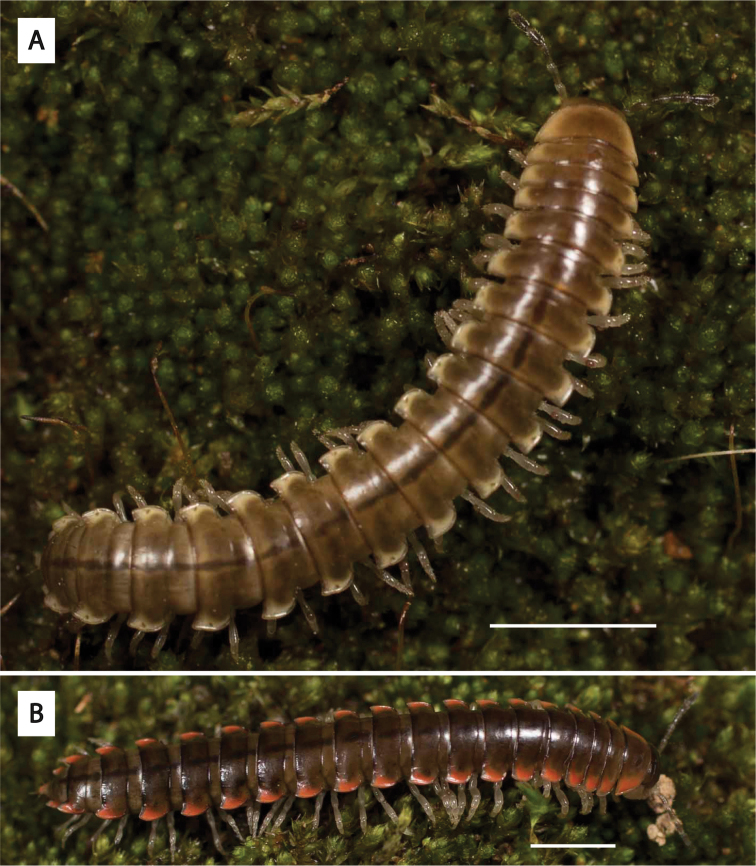
*Nannariaignis* sp. nov. coloration **A** non-type ♂ (VTEC, MPE01198) white paranota **B** non-type ♂ (VTEC, MPE01202) orange paranota. Scale bars: 4.0 mm.

Figs 61 , 62 Vernacular name: “The Dragon-Headed Twisted-Claw Millipede”

####### Material examined.

***Holotype*:** United States – **Virginia** • ♂; Bland County, base of Big Walker Mountain off powerline access road; 37.0383°N, -81.1090°W; elev. 828 m; 28 Mar. 2016; hand collected; J. Means, P. Marek, A. Prewitt leg.; VTEC MPE01063 •

***Paratype*:** United States – **Virginia** • 1 ♀; same collection data as holotype; VMNH MPE01064.

####### Other material.

United States – **Virginia** • 1 ♀; Bland County, 0.5 mi. down road on south side of Little Walker Mtn. near powerline; 37.0273°N, -81.0933°W; elev. 871 m; 6 Jan. 2016; hand collected; J. Means, D. Hennen, R. Jean, P. Marek leg.; VTEC MPE00917 • 2 ♂♂; Bland County, uphill of road, under mixed pine and hardwood ca. 0.3 m from power line on north side of LWM; 37.0302°N, -81.0977°W; elev. 879 m; 8 Jan. 2016; hand collected; J. Means, D. Hennen, P. Marek, R. Jean leg.; VTEC MPE00921, 922 • 1 ♂; Bland County; 37.0324°N, -81.0947°W; elev. 827 m; 21 Nov. 2016; hand collected; J. Means, P. Marek, A. Prewitt leg.; VTEC MPE01037 • 2 ♂♂; Bland County, next to rock face, outcrop going down from access road; 37.0478°N, -81.1155°W; elev. 1184 m; 17 May 2016; hand collected; D. Hennen, P. Shorter, D. Krall, A. Prewitt leg.; VTEC MPE01198, 1202 • 1 ♂; Bland County, Big Walker Mtn., 2 mi E of Sharon Spgs.; 37.0585°N, -81.0988°W; 28 July 1962; R. Hoffman leg.; VMNH NAN0010 • 2 ♂♂; Wythe County; 37.0257°N, -81.0901°W; elev. 838 m; Jan. 2016; hand collected; J. Means, D. Hennen, R. Jean, P. Marek leg.; VTEC MPE00912, 918 • 1 ♂; Wythe County; 37.0315°N, -81.0950°W; elev. 841 m; 21 Mar. 2016; hand collected; J. Means, P. Marek, A. Prewitt leg.; VTEC MPE01036. For detailed collection data see Suppl. material 7.

####### Diagnosis.

Adult males of *Nannariaignis* sp. nov. are distinct from other *Nannaria* and the sympatric *N.aenigma* based on the following combination of characters: ***Gonopods*.** Gonopodal acropodite gently curving medially before apex, not strongly curved as in *N.ohionis* Loomis & Hoffman, 1948. Distal zone curving dorsomedially (Fig. 61B, red arrow), not medially as in *N.ohionis* or *N.aenigma*. Tip with small, rounded lateral flange (Fig. 61A, red triangle),not simple as in *N.ohionis*, or serpentine as in *N.aenigma*. Telopodite basal zone reduced, ca. 1/4 length of acropodite, not > 1/3 length as in *N.ohionis*, or ca. 1/6 length as in *N.aenigma*. Prefemur with serpentine prefemoral process, bending ventrally before curving cephalolaterally (Fig. 61B), not straight, acicular as in *N.ohionis* or *N.aenigma*. Prefemoral process ca. 3/4 length of acropodite, not ca. 1/2 length as in *N.aenigma*. Prefemoral process arising dorsomedially from large, sharp prefemoral spine, not arising from top of prefemoral spine as in *N.ohionis*. ***Color*.** Tergites with white (Fig. 62A) or orange (Fig. 62B) paranotal spots. Dark brown background. Dorsum of collum smooth with either white or orange margin, depending on color morph.

####### Measurements.

♂ holotype (VTEC, MPE01063): BL = 25.3, CW = 3.2, IW = 2.3, ISW = 0.8, B11W = 3.9, B11H = 2.5; ♀ paratype (VMNH, MPE01064): BL = 27.6, CW = 3.6, IW = 1.9, ISW = 0.9, B11W = 4.6, B11H = 3.2.

####### Variation.

Individuals of *N.ignis* sp. nov. from the ridge of Big Walker Mountain have slightly wider, more laminate prefemoral processes, and a more pronounced lateral flange at the tip of the acropodite.

####### Distribution.

Known only from a small area on the border of Bland and Wythe counties in southwestern Virginia, and on both Big and Little Walker Mountains (Virginia: Bland and Wythe counties; Suppl. material 7; Fig. 126). Distribution area: 3.5 km^2^; status: MRE.

####### Ecology.

Individuals of *N.ignis* sp. nov. were collected from mesic broadleaved forests composed of oak, maple, laurel, and some pine.

####### Etymology.

This species is named for the shape of its acropodite in the medial view, which resembles a dragon’s head and neck. The specific name is a noun in apposition derived from the Latin *ignis* meaning fire.

####### Type locality.

United States, Virginia, Bland County, base of Big Walker Mountain off powerline access road, 37.0383°N, -81.1090°W.

###### Nannaria tenuis sp. nov.

Taxon classificationAnimaliaPolydesmidaXystodesmidae

D7C7F6D8-E412-5D00-AE9E-E4639923955F

http://zoobank.org/8280982C-467A-4445-B146-53014CFAB831

**Figure 63.**
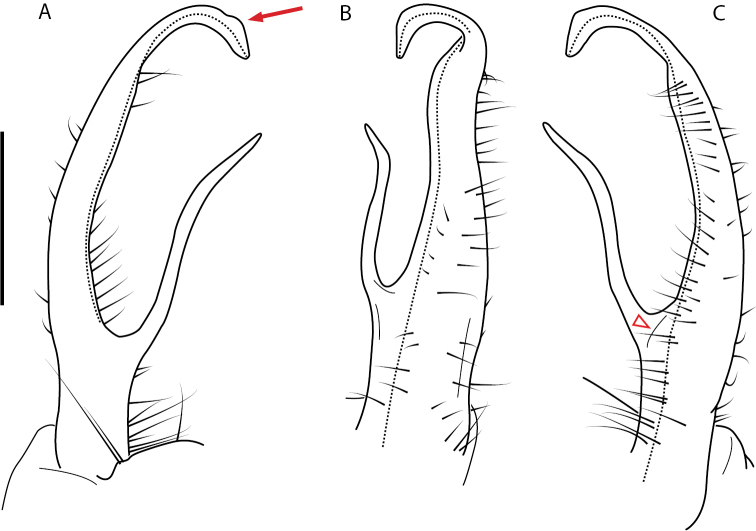
*Nannariatenuis* sp. nov. ♂ holotype (VTEC, MPE00990) left gonopod **A** anterior view; red arrow indicates small lateral flange **B** medial view **C** posterior view; red triangle indicates reduced prefemoral spine. Scale bar: 0.5 mm.

**Figure 64.**
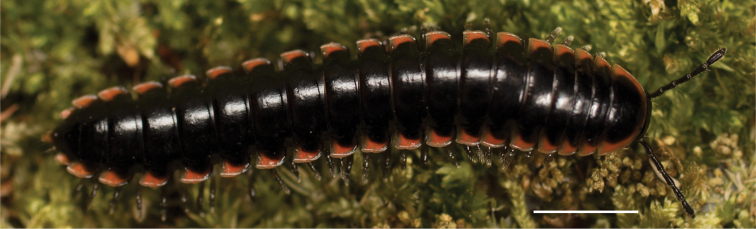
*Nannariatenuis* sp. nov. ♂ non-type (VTEC, MPE00991) coloration. Scale bar: 4.0 mm.

Figs 63 , 64 Vernacular name: “The Svelte Twisted-Claw Millipede”

####### Material examined.

***Holotype*:** United States – **Virginia** • ♂; Bland Co., 1.0 km southeast of Bastian; 37.1453°N, -81.1417°W; elev. 728 m; 2 Feb. 2016; hand collected; J. Means, P. Marek, T. Price leg.; VTEC MPE00990.

***Paratype*:** United States – **Virginia** • ♀; Bland County, 1.6 km southeast of Bastian; 37.1407°N, -81.1394°W; elev. 797 m; 15 Jan. 2016; hand collected; J. Means, P. Marek, V. Wong, T. Price leg.; VMNH MPE00925.

####### Other material.

United States – **Virginia** • 1 ♂; Bland county, in gully, side of hill, by creek near power line; 37.1537°N, -81.1450°W; elev. 712 m; 8 Feb. 2016; hand collected; J. Means, P. Marek, T. Price, Kyle leg.; VTEC MPE00991 • 3 ♀♀; Bland County; 37.1193°N, -81.1357°W; elev. 874; 1 Apr. 2016; J. Means, P. Marek, A. Prewitt leg.; VTEC MPE01087–89 • 1 ♀; Bland County; 37.1184°N, -81.1360°W; elev. 861 m; 1 Apr. 2016; J. Means, P. Marek, A. Prewitt leg.; VTEC MPE01095 • 1 ♂; Bland County; 37.1159°N, -81.1363°W; elev. 890 m; 1 Apr. 2016; hand collected; J. Means, P. Marek, A. Prewitt leg.; VTEC MPE01086 • 1 ♂; Bland County; 37.1149°N, -81.1347°W; elev. 864 m; 1 Apr. 2016; hand collected; J. Means, P. Marek, A. Prewitt leg.; VTEC MPE01106 • 1 ♀; Bland County; 37.1134°N, -81.1339°W; elev. 842 m; 1 Apr. 2016; hand collected; J. Means, P. Marek, A. Prewitt leg.; VTEC MPE01109 • 1 ♀; Bland County; 37.1200°N, -81.1360°W; elev. 894 m; 7 Apr. 2016; hand collected; J. Means, P. Marek, A. Prewitt, Tyler, Renea leg.; VTEC MPE01115 • 1 ♀; Bland County; 37.1218°N, -81.1356°W; elev. 889 m; 7 Apr. 2016; hand collected; J. Means, P. Marek, A. Prewitt, Tyler, Renea leg.; VTEC MPE01114 • 1 ♂; Bland County; 37.1234°N, -81.1355°W; elev. 889 m; 7 Apr. 2016; hand collected; J. Means, P. Marek, A. Prewitt, Tyler, Renea leg.; VTEC MPE01111 • 2 ♀♀; same collection data as preceding; VTEC MPE01112, 1113 • 1 ♂; Bland County, off AT down from the road; 37.1369°N, -81.1372°W; elev. 939 m; 20 May 2016; hand collected; J. Means, P. Shorter, D. Krall leg.; VTEC MPE01319 • 1 ♀; same collection data as preceding; VTEC MPE01320 • 1 ♂; Bland County, small creek with forest on either side, gully; 37.1449°N, -81.1412°W; elev. 748 m; 20 May 2016; hand collected; J. Means, P. Shorter, D. Krall leg.; VTEC MPE01317 • 1 ♀; same collection data as preceding; VTEC, MPE01318. For detailed collection data see Suppl. material 7.

####### Diagnosis.

Adult males of *Nannariatenuis* sp. nov. are distinct from other *Nannaria* and the sympatric *N.aenigma*, based on the following combination of characters: ***Gonopods*.** Gonopodal acropodite gently curving medially before apex, distal zone curving dorsomedially, with caudally directed tip, not medially directed as in *N.ignis* sp. nov. Tip rounded with small lateral flange (Fig. 63A, red arrow), not thin and serpentine, lacking flanges as in *N.aenigma*. Acropodite with medial swelling before apex, not simple as in *N.ignis* sp. nov. and *N.aenigma*. Telopodite basal zone thin and elongate, > 1/3 length of acropodite, not ca. 1/4 length of acropodite as in *N.ignis* sp. nov., or ca. 1/6 length as in *N.aenigma*. Prefemur with dorsomedially curving prefemoral process, not serpentine, curving ventrally and bending cephalo-laterally at tip as in *N.ignis* sp. nov., or straight, acicular as in *N.aenigma*. Prefemoral spine reduced to slight swelling at base of prefemoral process (Fig. 63C, red triangle), not large, projected as in *N.ignis* sp. nov. ***Color*.** Tergites with orange paranotal spots (Fig. 64). Black background. Dorsum of collum smooth with caudal orange margin.

####### Measurements.

♂ holotype (VTEC, MPE00990): BL = 30.6, CW = 4.1, IW = 2.4, ISW = 0.9, B11W = 5.1, B11H = 3.3; ♀ paratype (VMNH, MPE00925): BL = 35.4, CW = 3.9, IW = 2.6, ISW = 1.0, B11W = 5.1, B11H = 3.6.

####### Variation.

No known variation.

####### Distribution.

Known only from southwestern Virginia (Virginia: Bland County; Suppl. material 7; Fig. 126). The distribution of *N.tenuis* sp. nov. is restricted to the area just southeast of Bastian, Virginia. Distribution area: < 1 km^2^; status: MRE.

####### Ecology.

Individuals of *N.tenuis* sp. nov. were collected in winter and spring from mesic broadleaved forests, were found under hardwood leaf litter, and were occasionally beneath 1–2 cm of soil. Two individuals, including the female holotype, were found under ca. 7 cm of snow when temperatures were below freezing, suggesting that there ostensibly exists some cold tolerance in *Nannaria*.

####### Etymology.

This species is named for its strikingly thin telopodite basal zone. The specific name is an adjective derived from the Latin *tenuis*, meaning thin.

####### Type locality.

United States, Virginia, Bland Co., 1.0 km southeast of Bastian; 37.1453°N, -81.1417°W.

##### *ohionis* clade

**Components.***Nannariaohionis*, *N.sheari* sp. nov., *N.suprema* sp. nov., and female specimens from Stone Mtn. State Park in North Carolina and The Blue Hole, Tennessee (Fig. 114; Supplemental Material 2). Members of the *ohionis* clade share gonopodal characters, including an acropodite tip with a small lateral flange. The *ohionis* clade is closely related to the *ignis* clade, and may represent one, larger group, as evidenced by the low PP (< 0.70) separating the two clades. We expect that further sampling and genetic evidence may prove this group to be polyphyletic, perhaps uniting *N.sheari* sp. nov. with the closely related *ignis* clade, or discovering transitional forms between these species.

**Distribution.** the *ohionis* clade extends from southeastern Ohio south into West Virginia, southwestern Virginia, northeastern Tennessee, and northwestern North Carolina (Fig. 114).

###### Nannaria ohionis

Taxon classificationAnimaliaPolydesmidaXystodesmidae

Loomis & Hoffman, 1948

07D9E006-DA45-5990-99D8-2818A2E73C44

**Figure 65.**
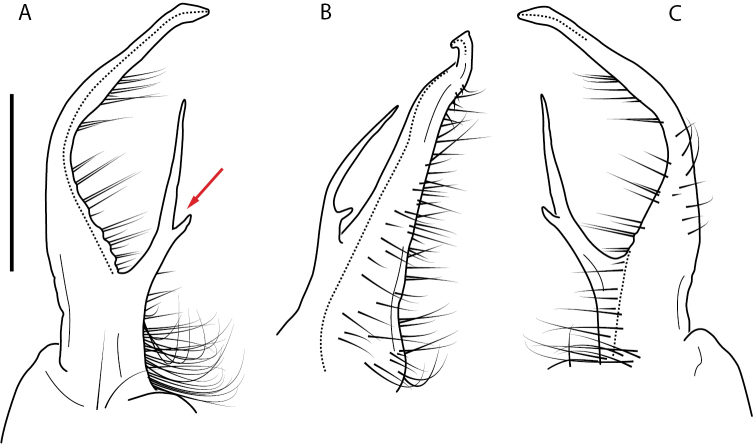
*Nannariaohionis* Loomis & Hoffman, 1948 neotype ♂ (VTEC, MPE00906) left gonopod. **A** anterior view; red arrow indicates prefemoral process arising from top of prefemoral spine **B** medial view **C** posterior view. Scale bar: 0.5 mm.

**Figure 66.**
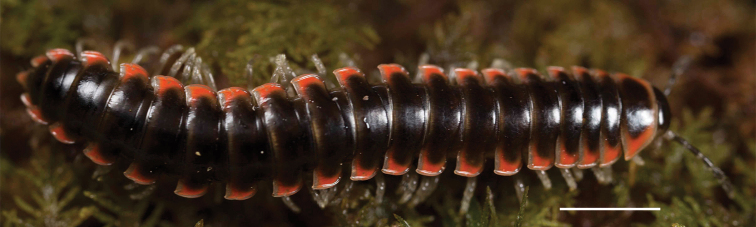
*Nannariaohionis* Loomis & Hoffman, 1948 neotype ♂ (VTEC, MPE00906) coloration. Scale bar: 4.0 mm.

Figs 65 , 66. Vernacular name: “The Southern Ohio Twisted-Claw Millipede”

Fontaria castanea : Williams & Hefner, 1928: 106, fig. 9b
Nannaria ohionis Loomis & Hoffman, 1948: 53. Hoffman 1999: 367. Marek et al. 2014: 37. Means et al. 2021: S71–S72.
Mimuloria ohionis : Causey 1952: 8. Chamberlin and Hoffman 1958: 38.

####### Material examined.

***Neotype* (here designated)**: United States – **Ohio** • ♂; Athens County, Coolville, Hennen Ln.; 39.2107°N, -81.8421°W; elev. 242 m; 25 Nov. 2015; hand collected; D. Hennen leg.; VTEC MPE00906.

####### Other material.

United States – **Ohio** • 1 ♂; Hocking County, Hocking Hills State Park, Cantwell Cliffs; 39.5420°N, -82.5755°W; elev. 270 m; 22 Oct. 2016; hand collected; D. Hennen leg.; VTEC MPE02270 • 2 ♂♂; same collection data as preceding; 29 Sep. 1963; FAC leg.; VMNH NAN0026 • 2 ♂♂; same collection data as preceding; 15 May 1961; J. Crites leg.; VMNH NAN0326, 327 • 5 ♀; Hocking County, Crane Hollow Nature Preserve, in hollow behind Ellis House; 39.4913°N, -82.5797°W; elev. 290 m; 15 June 2016; hand collected; J. Means, D. Hennen leg.; VTEC MPE01707–11 • 1 ♂; Lawrence County, Pedro, Lake Vesuvius Rec Area, hillside across street from Roadside Group Picnic Shelter; 38.6064°N, -82.6289°W; elev. 195 m; 6 July 2017; hand collected; D. Hennen leg.; VTEC MPE03678 • 1 ♂; Meigs County, Morgan’s Cave, under rocks; 39.0874°N, -81.9971°W; 5 Mar. 1964; L. Carr leg.; VMNH NAN0169 • 1 ♂; Monroe County, Sardis, Narrows Run Rd.; 39.6150°N, -80.9369°W; elev. 219 m; 7 Oct. 2018; L. Hughes leg.; VTEC MPE04611 • 2 ♂♂; Washington County, Little Hocking Nature Trail, next to Little Hocking Elementary School, off Newbury Road; 39.2583°N, -81.7044°W; elev. 195 m; 27 Dec. 2016; hand collected; D. Hennen leg.; VTEC MPE02271, 2273 • 1 ♂; Washington County, Hune Bridge, hillside above parking lot; 39.5092°N, -81.2508°W; elev. 210 m; 19 Aug. 2017; hand collected; D. Hennen, K. Lustofin, M. Spring leg.; VTEC MPE03003 • 1 ♀; same collection data as preceding; VTEC MPE03004 • 2 ♂♂; Washington County, Marietta, Washington County Career Center; 39.4307°N, -81.5003°W; elev. 263 m; 28 Nov. 2015; hand collected; D. Hennen leg.; VTEC MPE00907; SCAU – **West Virginia** • 1 ♂; Wood County, Waverly, Mountwood Park, 1100 Volcano Rd., north-facing hillside near parking lot; 39.2420°N, -81.2991°W; elev. 292 m; 25 Nov. 2017; hand collected; D. Hennen leg.; VTEC MPE03643. For detailed collection data see Suppl. material 7.

####### Diagnosis.

Adult males of *Nannariaohionis* are distinct from other *Nannaria*, the sympatric *N.terricola*, and the nearby *N.shenandoa*, based on the following combination of characters: ***Gonopods*.** Gonopodal acropodite gently curving medially before apex (Fig. 65), not straight or slightly curved as in *N.terricola*, or strongly curved as in *N.shenandoa*. Distal zone and tip short, simple, bending medially, not bending dorsally as in *N.terricola* or large with flanges, curving posterolaterally as in *N.shenandoa*. Telopodite basal zone height ca. 1/3 length of acropodite, not ca. 1/2 as in *N.terricola*, or ca. 1/4 as in *N.shenandoa*. Prefemur with straight, acicular prefemoral process, not curving laterally as in *N.shenandoa*. Prefemoral process arising from top of prefemoral spine, not from the prefemur as in *N.terricola* and *N.shenandoa* (Fig. 65A, red arrow). Prefemoral spine large and projecting, acicular, –not reduced, shelf-like, fused with prefemoral process as in *N.terricola*. ***Color*.** Tergites with orange paranotal spots (Fig. 66). Background variable between individuals, varying from dark brown to black. Dorsum of collum smooth with orange margin.

####### Measurements.

♂ neotype (VTEC, MPE00906): BL = 32.0, CW = 4.0, IW = 2.0, ISW = 0.9, B11W = 4.7, B11H = 3.4; ♀ (VTEC, MPE03004): BL = 32.6, CW = 4.1, IW = 2.4, ISW = 1.0, B11W = 5.2, B11H = 3.9.

####### Variation.

No known variation.

####### Distribution.

Known from southeastern Ohio and northwestern West Virginia (Ohio: Athens, Hocking, Lawrence, Meigs, and Washington counties; West Virginia: Wood County, Suppl. material 7, Fig. 127). Distribution area: 5,823 km^2^; status: SRE.

####### Ecology.

Individuals of *N.ohionis* have been collected from mesic broadleaved forests composed of red maple, birch, tuliptree, oak, hickory, pawpaw, American beech, sycamore, buckeye, spicebush, sourwood, and hemlock, often from hillsides and beside walking trails, under leaf litter and logs.

####### Etymology.

Loomis and Hoffman (1948) give no justification for their naming, however it is reasonable to assume this species is named after its type locality, Ohio.

####### Type locality.

United States, Ohio, Athens County, Coolville, Hennen Ln., 39.2107°N, -81.8421°W.

####### Notes.

We designate a neotype for *N.ohionis* because a holotype was never designated by either Williams and Hefner (1928) or by Loomis and Hoffman (1948). Williams and Hefner (1928) illustrated a millipede found in Ohio and identified it as *Fontariacastanea* (McNeill, 1887). Loomis and Hoffman (1948) recognized that this illustration was not of *F.castanea*, but rather a yet-undescribed species of *Nannaria*, which they named *Nannariaohionis*. Chamberlin and Hoffman (1958, p. 38) stated that the “type” was at Miami University; however, searches of the collection by the current curator at our behest revealed no millipede specimens. Causey (1952: 8) mentions and illustrates a “paratype” of *N.ohionis* loaned to her by Hefner, but does not mention where the “paratype” was held. Because no type material was ever designated for *N.ohionis*, and due to the lack of any known location of material collected by Williams or Hefner, we felt a neotype designation was warranted.

###### Nannaria sheari sp. nov.

Taxon classificationAnimaliaPolydesmidaXystodesmidae

7ABBB4AF-FE4D-5FD6-8BFD-5E2F456842C3

http://zoobank.org/2A2E7202-5E4E-4915-9533-5FD5235834E8

**Figure 67.**
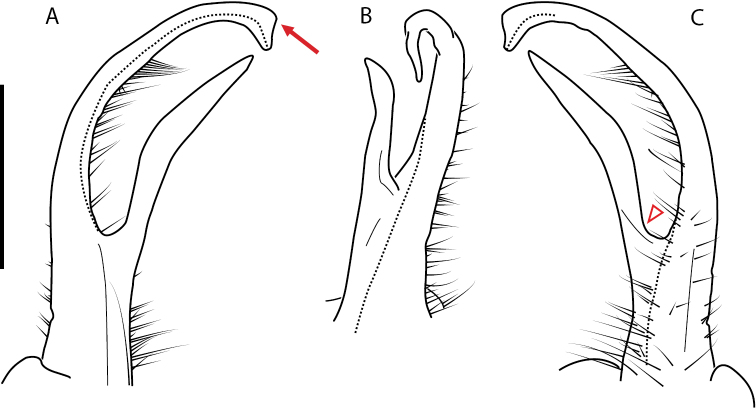
*Nannariasheari* sp. nov. paratype ♂ (VMNH, NAN0012) left gonopod **A** anterior view; red arrow indicates lateral flange **B** medial view **C** posterior view; red triangle indicates reduced, ridge-like prefemoral spine. Scale bar: 0.5 mm.

**Figure 68.**
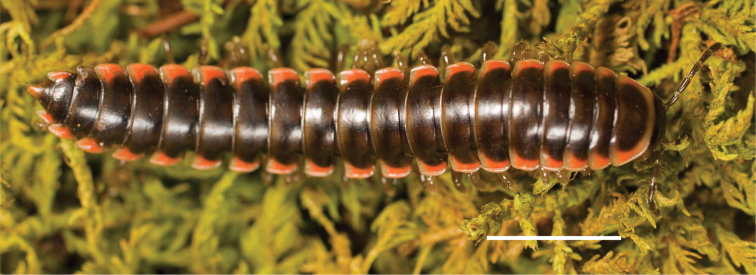
*Nannariasheari* sp. nov. holotype ♂ (VTEC, MPE01684) coloration. Scale bar: 4.0 mm.

Figs 67 , 68 Vernacular name: “William Shear’s Twisted-Claw Millipede”

####### Material examined.

***Holotype*:** United States – **West Virginia** • ♂; Mercer County, Brush Creek Preserve, along trail to waterfall; 37.4647°N, -81.0623°W; elev. 628 m; 14 June 2016; hand collected; J. Means, D. Hennen leg.; VTEC MPE01684.

***Paratypes*:** United States – **West Virginia** • 1 ♀; same collection data as holotype; VMNH MPE01685 • 1 ♂; Mercer County, Brush Creek Falls, Tullgren, sort of Rhododendron litter; 37.4671°N, -81.0597°W; 5 Oct.1967; W. Shear leg.; VMNH NAN0012.

####### Other material.

United States – **West Virginia** • 1 ♀; Mercer County, Speedway Hemlock Grove, oak-pine litter; 37.4564°N, -81.0105°W; 4 Apr. 1967; W. Shear leg.; VMNH NAN0002 • 2 ♂♂; same collection data as preceding; VMNH NAN0048 • 13 ♂♀; Mercer County, Speedway Roadside Park, route 20, 3 mi. north of Athens, hibernating aggregation of dead animals under log; 37.4646°N, -81.0112°W; 9 Mar. 1968; W. Shear leg.; VMNH NAN0020 • 3 ♂♂; Mercer County, Brush Creek Preserve, along trail to waterfall; 37.4647°N, -81.0623°W; elev. 628 m; 14 June 2016; hand collected; J. Means, D. Hennen leg.; VTEC MPE01665–7 • 1 ♂ and 1 ♀; Mercer County, Athens, Jackson’s Park; 37.4222°N, -81.0163°W; 30 Mar. 1968; W. Shear leg.; VMNH NAN0013 • 1 ♂; Mercer County, Athens, 218 W. Broadway; 37.4254°N, -81.0211°W; 29 Mar. 1973; W. Shear leg.; VMNH NAN0046. For detailed collection data see Suppl. material 7.

####### Diagnosis.

Adult males of *Nannariasheari* sp. nov. are distinct from other *Nannaria* and the nearby *N.aenigma*, based on the following combination of characters: ***Gonopods*.** Gonopodal acropodite continually curving medially, not relatively straight before curving medially at half-way point as in *N.castra* sp. nov. Distal zone curving medially, not dorsally as in *N.castra* sp. nov. Acropodite tip directed caudally (Fig. 67B), not medially as in *N.aenigma*. Acropodite tip with lateral flange (Fig. 67, red arrow). Acropodite simple in *N.sheari* sp. nov., lacking slight twist and swelling at midpoint as found in *N.castra* sp. nov., and lateral flange as found in *N.aenigma*. Telopodite basal zone with slight lateral bulge and wider than space between prefemoral process and acropodite, not thinner as in *N.castra* sp. nov. Prefemur with dorsomedially curving prefemoral process (Fig. 67B), not straight as in *N.aenigma*. Prefemoral spine fused with prefemoral process, reduced to shelf-like ridge at base of prefemoral process (Fig. 67C, red triangle). ***Color*.** Tergites with orange paranotal spots (Fig. 68). Dark brown background. Dorsum of collum smooth with orange and white margin.

####### Measurements.

♂ holotype (VTEC, MPE01684): BL = 30.7, CW = 3.5, IW = 1.9, ISW = 0.7, B11W = 4.1, B11H = 2.4. ♀ paratype (VMNH, MPE01685): BL = 30.2, CW = 3.2, IW = 1.9, ISW = 0.8, B11W = 4.5, B11H = 3.3.

####### Variation.

No known variation.

####### Distribution.

Known from a small area in and around Athens, West Virginia (West Virginia: Mercer County, Suppl. material 7; Fig. 126). Distribution area: 12 km^2^; status: MRE.

####### Ecology.

Individuals of *N.sheari* sp. nov. have been collected from mesic hardwood forests composed of oak, tuliptree, birch, maple, buckeye, rhododendron, and some pine and hemlock. Individuals were found under deciduous leaf litter by the side of hiking trails.

####### Etymology.

This species is named after its original collector, and a longtime mentor to the authors, Dr. William Shear. The specific name is a genitive noun derived as a patronym.

####### Type locality.

United States, West Virginia, Mercer County, Brush Creek Preserve, along trail to waterfall, 37.4647°N, -81.0623°W.

###### Nannaria suprema sp. nov.

Taxon classificationAnimaliaPolydesmidaXystodesmidae

FDECD9E7-422A-55F2-B0E1-EF3F14EE5859

http://zoobank.org/33E2F68E-ACCA-4370-B759-3ECA81A6DDA9

**Figure 69.**
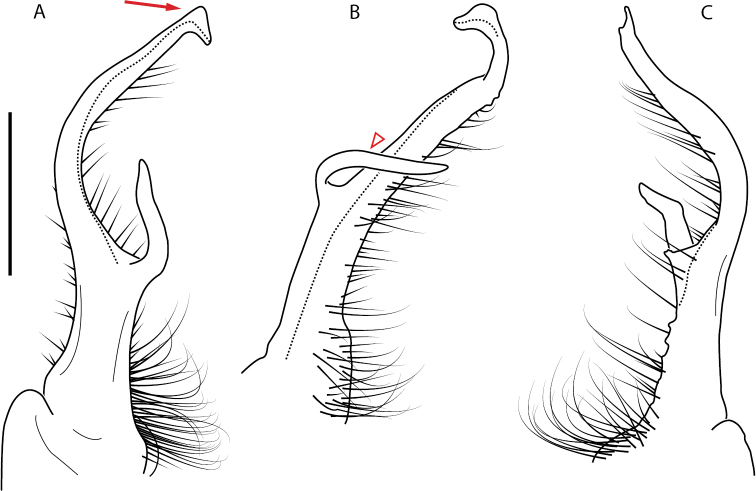
*Nannariasuprema* sp. nov. holotype ♂ (VTEC, MPE00075) left gonopod **A** anterior view; red arrow indicates small, triangular lateral flange **B** medial view; red triangle indicates ventrally curving prefemoral process **C** posterior view. Scale bar: 0.5 mm.

**Figure 70.**
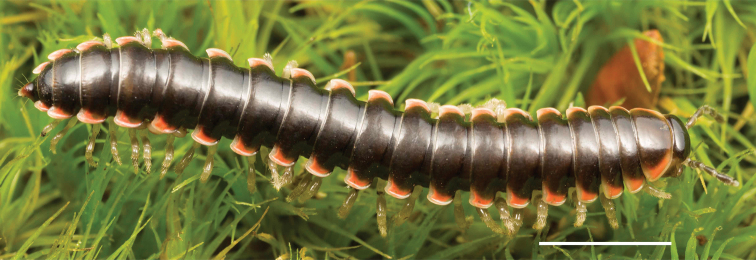
*Nannariasuprema* sp. nov. holotype ♂ (VTEC, MPE00075) coloration. Scale bar: 4.0 mm.

Figs 69 , 70 Vernacular name: “The High Elevation Twisted-Claw Millipede”

####### Material examined.

***Holotype*:** United States – **Virginia** • ♂; Smyth County, Mount Rogers Natural Rec Area, FR 84; 36.70672°N, -81.60284°W; elev. 1318 m; 25 June 2014; hand collected; J. Means, P. Marek and E. Francis leg.; VTEC MPE00075.

***Paratypes*:** United States – **Virginia** • 1 ♀; same collection data as holotype; VTEC MPE00066 • 1 ♀; same collection data as holotype; VMNH MPE00068.

####### Other material.

United States – **Tennessee** • 2 ♂♂; Johnson County, 2 NE Shady Valley McQueen Gap Rd., 1 jct. Hwy 133 Harp Mtn.; 36.5580°N, -81.9097°W; May 2002; A. Gagan leg.; VMNH NAN0459; SCAU – **Virginia** • 1 ♂; Grayson County, Grayson Highlands State Park, Haw Orchard Mountain, Twin Pinnacles trail; 36.6250°N, -81.5020°W; elev. 1530 m; 10 June 2017; hand collected; C. Harden leg.; VTEC MPE03436 • 1 ♂; Grayson County, Grayson Highlands State Pk Haw Orchard Mtn., DF site 1, behind water tank; 36.6251°N, -81.5008°W; 17 Sep. 1990; VMNH survey leg.; VMNH NAN0121 • 6 ♂♂; Grayson County, Grayson Highlands State Park, DF site 1, nr Visitor Center; 36.6242°N, -81.4979°W; 17 Oct. 1990; VMNH survey leg.; VMNH NAN0132 • 1 ♂; same collection data as preceding; 20 May 1991; VMNH NAN0357 • 7 ♂♂; Grayson County, Grayson Highlands State Park DF site 2, below picnic area, Haw Orchard Mountain; 36.6242°N, -81.4979°W; 17 Sep. 1990; VMNH survey leg.; VMNH NAN0122 • 11 ♂♂; same collection data as preceding; 17 Oct. 1990; VMNH NAN0127 • 1 ♂; Grayson County, Grayson Highland State Park, site 1, above water tank, Haw Orchard Mtn.; 36.6250°N, -81.5008°W; 2 June 1991; VMNH survey leg.; VMNH NAN0123 • 1 ♂; same collection data as preceding; 30 Aug. 1990; VMNH NAN0415 • 2 ♂♂; Grayson County, drift fence site 1 Grayson Highlands State Park; 36.6211°N, -81.4845°W; 2 Oct. 1990; VMNH survey leg.; VMNH NAN0413 • 3 ♂♂; Grayson County, Whitetop Mtn., DF site off FS 89, beechwoods; 36.6387°N, -81.6055°W; elev. 1524 m; 7 Sep. 1990; VMNH survey leg.; VMNH NAN0125 • 2 ♂♂; same collection data as preceding; 18 Nov. 1993; VMNH NAN0129 • 6 ♂♂; same collection data as preceding; 11 July 1993; VMNH NAN0131 • 2 ♂♂; same collection data as preceding; 15 Mar. – 23 Apr. 1994; VMNH NAN0139 • 1 ♂; Grayson County, S. slope Mt. Rogers; 36.6597°N, -81.5447°W; elev. 1372 m; 19 Apr. 1970; Karren leg.; VMNH NAN0134 • 1 ♂; same collection data as preceding; 19 May 1957; Highton, R. Hoffman leg.; VMNH NAN0135 • 1 ♂; same collection data as preceding; 27 Sep. 1969; group trip leg.; VMNH NAN0136 • 2 ♂♂; from the border of Johnson and Sullivan counties, top of Holston Mtn., 2 mi. NW of Shady Valley, U.S. Hyw. 421; 36.5394°N, -81.9535°W; 17 May 1974; R. Hoffman leg.; VMNH NAN0171 • 1 ♂; Grayson County, Grayson Highlands State Park at Massie’s Gap; 36.6242°N, -81.4979°W; elev. 1372 m; 23 Aug. 1984; A. Garland leg.; VMNH NAN0179 • 1 ♂; Smyth County, Sugar Grove, Raccoon Branch Wilderness campground, near beginning of Raccoon Branch Trail by campsite 8; 36.7462°N, -81.4247°W; elev. 855 m; hand collected; D. Hennen leg.; VTEC MPE02634 • 1 ♂; Smyth County, Va. Hy. 600, halfway between Konnarock and Elk Garden; 36.7003°N, -81.6109°W; elev. 1219 m; 10 May 1982; R. Hoffman et alia leg; VMNH NAN0128. For detailed collection data see Suppl. material 7.

####### Diagnosis.

Adult males of *Nannariasuprema* sp. nov. are distinct from other *Nannaria* and the sympatric *N.aenigma*, based on the following combination of characters: ***Gonopods*.** Acropodite simple and curving medially, without lateral flange as in *N.aenigma*. Distal zone short, directed dorsomedially (Fig. 69C), with large, triangular lateral flange (Fig. 69A, red arrow)—not serpentine as in *N.aenigma*. Telopodite basal zone thin with lateral bulge, not simple, straight as in *N.ambulatrix* sp. nov. and *N.aenigma*. Telopodite basal zone > 1/2 length of acropodite, not subequal to length as in *N.ambulatrix* sp. nov., or ca. 1/6 length as in and *N.aenigma*. Prefemur with serpentine, ventrally curving prefemoral process (Fig. 69B, red triangle), not medially curving as in *N.ambulatrix* sp. nov., or straight, acicular as in and *N.aenigma*. Prefemoral spine reduced, sharp. ***Color*.** Tergites with orange paranotal spots with lateral white trim (Fig. 70). Individuals variably with a dark brown to black background. Dorsum of collum smooth with orange margin with white trim.

####### Measurements.

♂ holotype (VTEC, MPE00075): BL = 30.1, CW = 3.4, IW = 2.2, ISW = 0.7, B11W = 4.4, B11H = 2.9; ♀ paratype (VTEC, MPE00066): BL = 31.8, CW = 4.0, IW = 2.2, ISW = 0.9, B11W = 5.3, B11H = 3.6.

####### Variation.

Some individuals from Grayson Highlands, Virginia, display a phenotypic variation wherein the acropodite tip is directed dorsally in the anterior view, rather than medially; though this is extremely rare.

####### Distribution.

Known from a small area in southwestern Virginia and northeastern Tennessee (Tennessee: Johnson and Sullivan counties; Virginia: Grayson and Smyth counties, Suppl. material 7; Fig. 126). Distribution area: 342 km^2^; status: MRE.

####### Ecology.

Individuals of *N.suprema* sp. nov. have been found in mesic hardwood forests dominated by oak, maple, rhododendron, hemlock, and red spruce; they are often found under logs and leaf litter.

####### Etymology.

This species is named for its occurrence at high elevations, including the peak of Mount Rogers, the highest point in Virginia. The specific name is derived from the Latin *supremus*, highest, and is a feminized adjective.

####### Type locality.

United States, Virginia, Smyth County, Mount Rogers Natural Rec Area, FR 84; 36.70672°N, -81.60284°W.

##### *blackmountainensis* clade

**Components.***Nannariaalpina* sp. nov., *N.blackmountainensis* sp. nov. and females from Turkey Foot Campground in Kentucky and Bamboo, North Carolina (Fig. 114, Supplemental Material 2). Members of the *blackmountainensis* clade share gonopodal characters, including a rectangular gonopod basal zone and a lateral flange on the acropodite apex, which becomes hooked in *N.blackmountainensis* sp. nov. While the two *N.blackmountainensis* sp. nov. specimens used in our genetic analysis were both from southeastern Kentucky, the VMNH collection includes specimens from a disparate population near Bamboo, North Carolina. This suggests that the female collected from Bamboo may either represent a third, basal species with similar morphology to *N.blackmountainensis* sp. nov., or an as-yet unseen species which is sympatric with the North Carolina *N.blackmountainensis* sp. nov.

**Distribution.** the *blackmountainensis* clade extends from eastern Kentucky, into southwestern Virginia, northeastern Tennessee, and northwestern North Carolina (Fig. 114).

###### Nannaria alpina sp. nov.

Taxon classificationAnimaliaPolydesmidaXystodesmidae

17529861-7A55-5462-921E-EE8CBCA13173

http://zoobank.org/D44E0F4B-C192-4706-8445-04E373AC9F66

**Figure 71.**
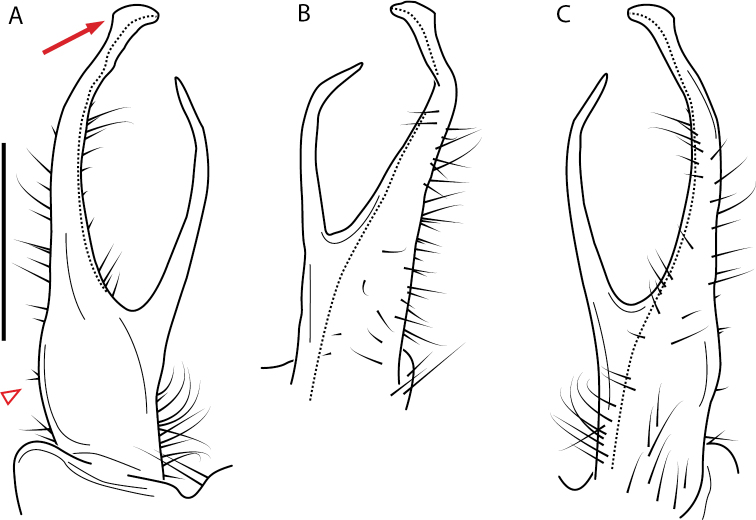
*Nannariaalpina* sp. nov. holotype ♂ left gonopod (VTEC, MPE03150) **A** anterior view; red arrow indicates triangular lateral flange; red triangle indicates lateral bulge **B** medial view **C** posterior view. Scale bar: 0.5 mm.

**Figure 72.**
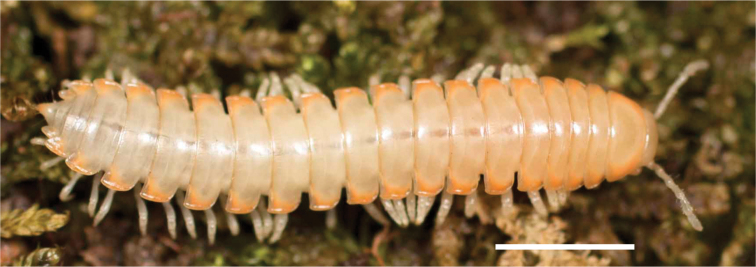
*Nannariaalpina* sp. nov. holotype ♂ (VTEC, MPE03150) coloration. Scale bar: 4.0 mm.

Figs 71 , 72 Vernacular name: “The Alpine Twisted-Claw Millipede”

####### Material examined.

***Holotype*:** United States – **Kentucky** • ♂; Pulaski County, Boone National Forest, Alpine Recreation Area; 36.9156°N, -84.5182°W; elev. 360 m; 27 Sept. 2017; J. Means, D. Hennen leg.; hand collected; VTEC MPE03150.

***Paratypes*:** United States – **Kentucky** • 1 ♂; same data as for holotype; VMNH MPE03199 • 3 ♀♀; same data as for holotype; VTEC MPE03200, 3201; VMNH, MPE03768. For detailed collection data see Suppl. material 7.

####### Diagnosis.

Adult males of *Nannariaalpina* sp. nov. are distinct from other *Nannaria* based on the following combination of characters. ***Gonopods*.** Acropodite straight, curving at 45° angle at apex, not gently curving throughout or with medial swelling as in *Nannariablackmountainensis* sp. nov. Acropodite tip with small, triangular lateral flange (Fig. 71A, red arrow), not with large, hooked lateral flange as in *N.blackmountainensis* sp. nov. Telopodite basal zone with slight lateral bulge (Fig. 71A, red triangle). Prefemur with laterally curving, acuminate prefemoral process; not straight, acicular prefemoral process as in *N.blackmountainensis* sp. nov. Gap between prefemoral process and acropodite greater than width of acropodite basal zone, not less than width of acropodite basal zone as in *N.blackmountainensis* sp. nov. Prefemur lacking prefemoral spine. ***Color*.** Tergites with pale orange paranotal spots (Fig. 72). White background (most likely due to teneral condition of the specimen). Dorsum of collum smooth with orange margin.

####### Measurements.

♂ holotype (VTEC, MPE03150): BL = 29.5, CW = 4.2, IW = 2.0, ISW = 0.9, B11W = 4.9, B11H = 3.1; ♀ paratype (VTEC, MPE03200): BL = 30.8, CW = 3.7, IW = 2.3, ISW = 0.9, B11W = 5.0, B11H = 3.5.

####### Variation.

No known variation.

####### Distribution.

Known only from the type locality, Alpine Recreation Area (Kentucky: Pulaski County; Suppl. material 7; Fig. 126). Distribution area: N/A; status: MRE.

####### Ecology.

Specimens of *Nannariaalpina* sp. nov. were collected from the side of a hiking path in a mesic broadleaf deciduous forest under ca. 3 cm of soil. Specimens were primarily found within molting chambers from a 1 m^2^ area.

####### Etymology.

This species is named for its type locality. The specific name is a feminine adjective derived from the Latin *alpinus* for alpine.

####### Type locality.

United States, Kentucky, Pulaski County, Boone National Forest, Alpine Recreation Area, 36.9156°N, -84.5182°W.

###### Nannaria blackmountainensis sp. nov.

Taxon classificationAnimaliaPolydesmidaXystodesmidae

058F8789-4B18-573E-8931-563DEA3F645E

http://zoobank.org/2ACF8697-BA97-4E11-BFBD-233FE0452FAB

**Figure 73.**
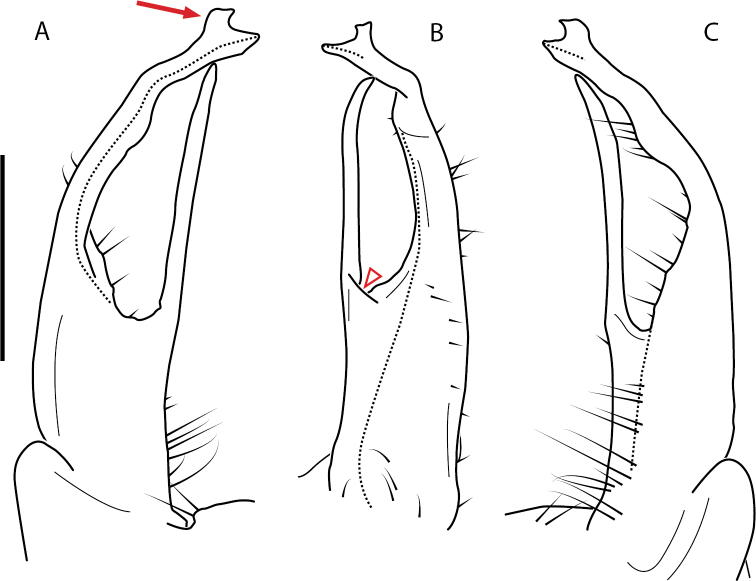
*Nannariablackmountainensis* sp. nov. holotype ♂ left gonopod (VTEC, SPC001090) **A** anterior view; red arrow indicates hooked lateral flange **B** medial view; red triangle indicates reduced prefemoral spine **C** posterior view. Scale bar: 0.5 mm.

**Figure 74.**
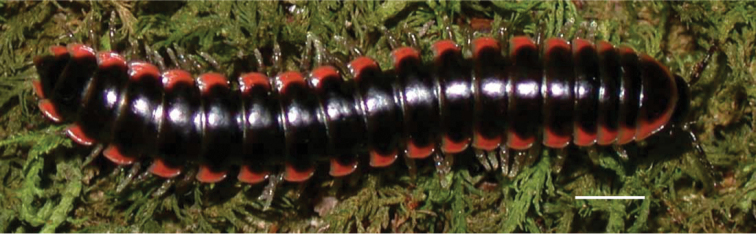
*Nannariablackmountainensis* sp. nov. paratype ♂ (VMNH, SPC001002) coloration. Scale bar: 4.0 mm.

Figs 73 , 74 Vernacular name: “The Black Mountain Twisted-Claw Millipede”

####### Material examined.

***Holotype*:** United States – **Kentucky** • ♂; Harlan Co., Black Mountain summit, radar station access road off Black Mountain Ridge Rd., about 2.4 rd. km E jct w/ KY-160; 36.9156°N, -82.8930°W; elev. 1250 m; 10 May 2011; hand collected; P. Marek, C. Hall and D. & M. Beamer leg.; VTEC SPC001090.

***Paratypes*:** United States – **Kentucky** • 1 ♂; Harlan Co., Stone Mountain State Natural Area, wooded slope above entrance ex US-421; 36.7607°N, -83.1400°W; elev. 570 m; 31 May 2006; hand collected; P. Marek leg.; VMNH SPC001002 • 1 ♀ same collection data as for preceding; VTEC SPC001009 • 1 ♂ Kentucky, Harlan Co., Cumberland Mountain (N Slope), Wagonroad Tunnel Trail; 36.7328°N, -83.2216°W; elev. 691 m; 25 July 2005; hand collected; P. Marek and C. Spruill leg.; FMNH SPC000652.

####### Other material.

United States – **Kentucky** • 1 ♂; Bell County, Pine Mountain State Park, trail to Honeymoon Falls; 36.7434°N, -83.7121°W; 26 Sept.1976; R. Hoffman leg.; VMNH NAN0184 • 1 ♂; Bell County, Pine Mtn. State Park; 36.6844°N, -83.8351°W; 10 May 1975; J. Ettman leg.; VMNH NAN0493 • 2 ♂♂; Bell County, Pine Mtn. State Park, Wildflower Garden Area; 36.7359°N, -83.7379°W; 1 Feb. 1976; J. Ettman leg.; NCSM NAN0545 • 1 ♀; Harlan County, Cumberland Mtn (N slope), Wagonroad Tunnel Trail (36.7389°N, -83.2197°W, elev. 528 m), 26 July 2005 (Coll: P. Marek, C. Spruill; VTEC SPC000794. SCAU – **North Carolina** • 1 ♂; Avery County, wooded hillside at Plumetree; 36.0269°N, -82.0080°W; 4 June 1964; L. Hubricht leg.; VMNH NAN0183 • 11 ♂♂♀♀; Avery County, 3.7 SSE Banner Elk, NC 105 & 184, Grandfather Mtn.; 36.1170°N, -81.8386°W; 8 Sept. 1973; R. Shelley; NCSM NAN0498 • 6 ♂♂; same collection data as for preceding; 1978; R. Shelley leg.; NCSM NAN0518 • 20 ♂♂♀♀; Avery County, Newland; 36.0873°N, -81.9273°W; 20 Aug. 1984; D. Massee leg.; NCSM NAN0522 • 1 ♂; Watauga County, Valle Crucis, NC 194, ca. 6S 1113; 36.2092°N, -81.7783°W; 11 Oct. 1975; J. Clamp leg.; NCSM NAN0515; SCAU – **Tennessee** • 1 ♂; Carter County, 2.5 miles S of Burbank; 36.1123°N, -82.1019°W; 2 May 1951; L. Hubricht leg.; VMNH, NAN0185 • 6 ♂; Carter County, Roan Mtn.; 36.1943°N, -82.0710°W; 9 Aug. 1941; Brooks leg.; VMNH, NAN0186 • 5 ♂♂; Carter County, N slope, Roane Mtn; 36.1079°N, -82.1280°W; 9 Aug. 1941; Dr. & Mrs. Brooks leg.; VMNH, NAN0294 • 1 ♂; Cumberland Mtns.; E. Cope leg.; VMNH, NAN0181; SCAU – **Virginia** • 1 ♀; Lee County, Poor Valley, Pennington Gap, VA. 621, ca.1.6 km E US. 421, on N facing slope; 36.7768°N, -83.0157°W, elev. 460 m; 10 June 2005; hand collected; P. Marek leg.; VTEC, SPC000540 • 1 ♂; Scott County, Powell Mtn. off FS 642, ca. 1 mi NW of Duffield, JNF; 36.7311°N, -82.8071°W; 13 Sept. 1994; C. Hobson, D. Stevenson leg.; VMNH NAN0182. For detailed collection data see Suppl. material 7.

####### Diagnosis.

Adult males of *Nannariablackmountainensis* sp. nov. are distinct from other *Nannaria* and the nearby *N.domestica* based on the following combination of characters: ***Gonopods*.** Gonopodal acropodite gently curving dorsomedially with pronounced medial swelling (Fig. 73A). Acropodite tip with large, hooked lateral flange (Fig. 73A, red arrow), not small, triangular lateral flange as in *N.domestica*. Tip terminating in small, dorsally directed rectangular point, not sharp, caudally directed as in *N.domestica*. Height of telopodite basal zone ca. 1/2 length of prefemoral process, not < 1/3 length as in *N.domestica*. Prefemoral process straight, acuminate, not laminate and serpentine as in *N.domestica*. Prefemoral spine reduced and fused to prefemoral process, forming small ridge (Fig. 15B, red triangle). ***Color*.** Tergites with hot orange/red paranotal spots (Fig. 74). Jet black background. Dorsum of collum smooth with orange margin.

####### Measurements.

♂ holotype (VTEC, SPC001090): BL = 31.2, CW = 3.6, IW = 2.3, ISW = 0.7, B11W = 4.7, B11H = 3.2. ♀ paratype (VTEC, SPC001009): BL = 35.4, CW = 4.1, IW = 2.8, ISW = 1.0, B11W = 5.5, B11H = 3.9.

####### Variation.

There exists a fair amount of variation amongst individuals of *N.blackmountainensis* sp. nov. from throughout the species’ range. Individuals from the northern border of Tennessee and North Carolina have medially curving prefemoral processes and sharp, pronounced prefemoral spines, while the only known specimen from near Crummies, Kentucky (SPC000652) has a small, triangular prefemoral spine. The southern populations of *N.blackmountainensis* may be discovered to be a separate species; however, such a discovery is contingent on the further collection of genetic material.

####### Distribution.

*Nannariablackmountainensis* sp. nov. has a disjunct distribution in the confluence of eastern Kentucky and Tennessee and western Virginia and North Carolina (Kentucky: Harlan and Bell counties; Virginia: Scott County; Tennessee: Cumberland and Carter counties; North Carolina: Avery and Watauga counties; Suppl. material 7; Fig. 126). Distribution area: 5,582 km^2^; status: SRE.

####### Ecology.

Individuals of *N.blackmountainensis* sp. nov. were collected from mesic forests of hemlock, tuliptree, maple, and oak, with an understory of jewelweed and stinging nettle.

####### Etymology.

This species is named for its type locality. The specific name is an adjective.

####### Type locality.

United States, Kentucky, Harlan Co., Black Mountain summit, radar station access road off Black Mountain Ridge Rd., about 2.4 rd. km E jct w/ KY-160, 36.9156°N, -82.8930°W.

##### *terricola* clade

**Components.***Nannariabobmareki* sp. nov., *N.dilatata* (Hennen & Shelley, 2015), *N.fracta* sp. nov., *N.solenas* sp. nov., *N.spruilli* sp. nov., *N.terricola*, and females from Little Coal River Campground, Boone County, West Virginia, Crane Hollow, Hocking County, Ohio, and Raven Run, Fayette County, Kentucky (Fig. 114; Suppl. material 2). Members of the *terricola* clade share gonopodal characters, including straight gonopods which do not cross in situ (with the exception of *N.dilatata*), the presence of a lateral basal bulge, and a medial flange near the acropodite apex. *Nannariadilatata* and the female specimens from Crane Hollow, Ohio, and Raven Run, Kentucky, form a clade which is sister to the rest of the *terricola* clade, and may represent a separately evolving lineage. The gonopods of *N.dilatata* are not as straight as in the other members of the *terricola* clade, and we look forward to the discovery of males from both the Crane Hollow and Raven Run populations. As with the *ohionis* and several other clades in the minor group, more sampling is needed to resolve the relationships within the *terricola* clade, but the *terricola* clade is one of the more morphologically homogenous clades in the minor species group.

**Distribution.** the *terricola* clade extends from southeastern Ohio, south into eastern Kentucky, West Virginia, southwestern Virginia, and central Tennessee (Fig. 114).

###### Nannaria bobmareki sp. nov.

Taxon classificationAnimaliaPolydesmidaXystodesmidae

3B7480FE-CEDF-5811-8E8F-F1CCBC194766

http://zoobank.org/485F1B62-722C-4887-B5D7-E47E34BAFF43

**Figure 75.**
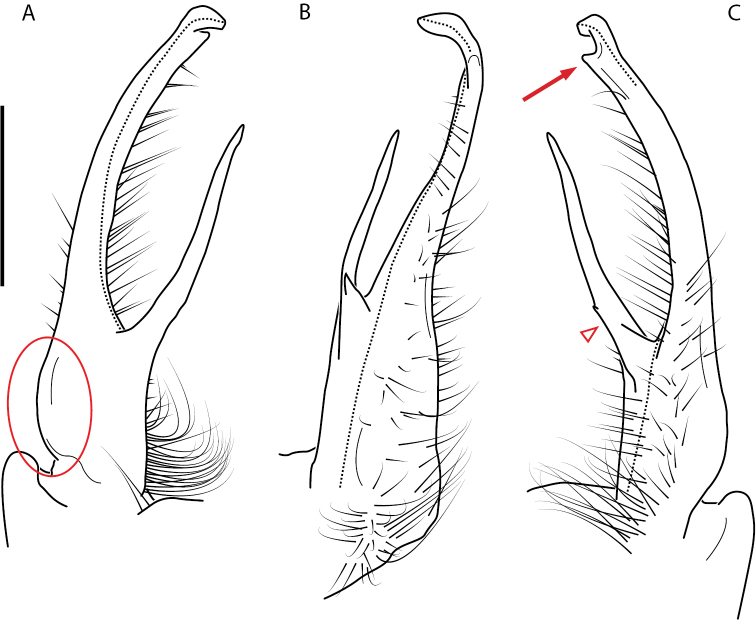
*Nannariabobmareki* sp. nov. ♂ holotype left gonopod (VTEC, SPC001019) **A** anterior view; red circle indicates slight basal swelling **B** medial view **C** posterior view; red arrow indicates shelf-like medial flange; red triangle indicates prefemoral spine partially fused with prefemoral process. Scale bar: 0.5 mm.

**Figure 76.**
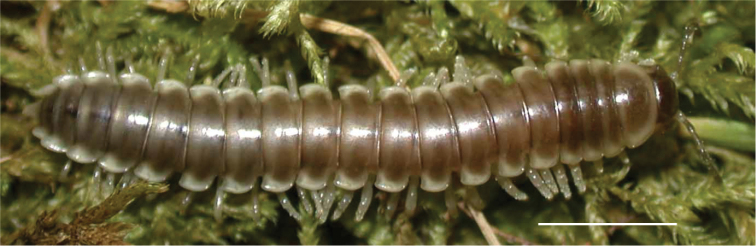
*Nannariabobmareki* sp. nov. ♂ holotype (VTEC, SPC001019) coloration. Scale bar: 4.0 mm.

**Figure 77.**
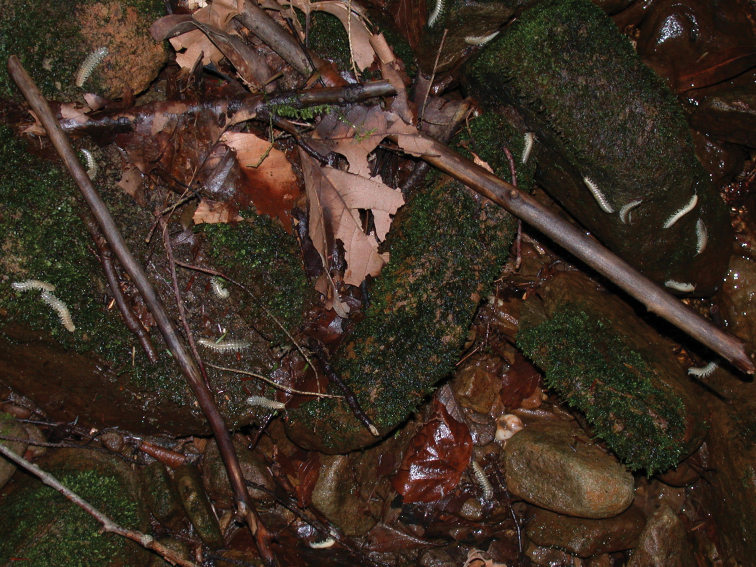
Small subset of *Nannariabobmareki* sp. nov. ♂ swarm observed in the Blanton Forest State Nature Preserve, Harlan Co., Kentucky.

Figs 75 , 76 , 77 Vernacular name: “Bob Marek’s Twisted-Claw Millipede”

Nannaria ‘Blanton’: Marek and Bond 2006: 721.

####### Material examined.

***Holotype***: United States – **Kentucky** • ♂; Leslie County, Cawood Recreation Site, about 5.4 rd km N jct KY-221 & US-421; 36.9364°N, -83.3729°W; elev. 417; 26 July 2006; hand collected; P. Marek and B. Marek leg.; VTEC SPC001019.

***Paratypes***: United States – **Kentucky** • 1 ♂; same collection data as for holotype; VMNH SPC001025 • 1 ♂; same collection data as for holotype; FMNH SPC001026 • 1 ♂; same collection data as for holotype; VTEC SPC001027 • 1 ♀; same collection data as for holotype; VTEC SPC001028.

####### Other material.

United States – **Kentucky** • 6 ♂♂ and 1 ♀; Harlan County Pine Mtn., Blanton Forest State Nature Preserve, High Fork Br., nr campground ranger station; 36.8594°N, -83.3823°W; elev. 411 m; 10 Aug. 2003; hand collected; P. Marek leg.; VTEC SPC000177–183 • 1 ♂; Harlan County, N slope Pine Mtn., James E Bickford Nature Preserve, Pine Mtn. Settlement School; 36.9473°N, -83.1807°W; elev. 587 m; 9 May 2011; hand colleted; P. Marek, C. Hall, D. Beamer, M. Beamer leg.; VTEC SPC001083. For detailed collection data see Suppl. material 7.

####### Diagnosis.

Adult males of *Nannariabobmareki* sp. nov. are distinct from other *Nannaria* and the nearby *N.aenigma*, based on the following combination of characters: ***Gonopods*.** Gonopodal acropodite acicular, bending abruptly medially at 90° at tip, not slightly curving medially before tip as in *N.fracta* sp. nov. or gently curving medially as in *N.aenigma*. Distal zone quadrate, not short, rounded, as in *N.fracta* sp. nov., or long and serpentine as in *N.aenigma*. Acropodite with small, shelf-like medial flange just before tip (Fig. 75C, red arrow). Prefemur with long, acicular prefemoral process with a pronounced, sharp prefemoral spine, partially fused to prefemoral process forming a ridge (Fig. 75C, red triangle), not free from prefemoral process as in *N.fracta* sp. nov. or lacking as in *N.aenigma*. Telopodite basal zone ca. 1/2 length of acropodite, not > 1/2 length of acropodite as in *N.fracta* sp. nov. or ca. 1/6 length of acropodite as in *N.aenigma*. Telopodite basal zone with slight medial swelling (Fig. 75A, red circle), not with pronounced medial swelling as in *N.fracta* sp. nov., or lacking as in *N.aenigma*. ***Color***: Tergites with white paranotal spots (Fig. 76). Dark brown background. Dorsum of collum smooth with white margin.

####### Measurements.

♂ holotype (VTEC, SPC001019): BL = 26.6, CW = 3.7, IW = 2.1, ISW = 0.9, B11W = 4.3, B11H = 2.8; ♀ paratype (VTEC, SPC001028): BL = 30.4, CW = 4.1, IW = 2.4, ISW = 1.2, B11W = 5.1, B11H = 3.6.

####### Variation.

No known variation.

####### Distribution.

Known from a small triangular area in southeastern Kentucky (Kentucky: Harlan and Leslie counties; Suppl. material 7; Fig. 126). Distribution area: 74 km^2^; status: MRE.

####### Ecology.

*Nannariabobmareki* sp. nov. is the only species of *Nannaria* which is known to engage in swarming behavior. PEM observed a swarm of an estimated 400 *N.bobmareki* sp. nov. individuals covering 4 m^2^ in the Blanton Forest State Nature Preserve in August of 2003 (Fig. 77). *Nannaria* are rarely found in high abundance, making swarming behavior especially notable. Additionally, *N.bobmareki* sp. nov. was encountered co-occurring with, but not participating in, a putative Müllerian mimicry ring composed of the xystodesmid species *Apheloriapolychroma* Marek, Means & Hennen, 2018 and *Brachoriaflammipes* Marek, 2010 (Marek 2010).

####### Etymology.

This species is named after its co-collector, Bob Marek. The specific name is a genitive noun derived as a patronym.

####### Type locality.

United States, Kentucky, Leslie County, Cawood Recreation Site, about 5.4 rd km N jct KY-221 & US-421, 36.9364°N, -83.3729°W.

###### Nannaria dilatata

Taxon classificationAnimaliaPolydesmidaXystodesmidae

(Hennen & Shelley, 2015)

F7825FCF-A44B-55C8-B556-5E4AB91BC1D1

**Figure 78.**
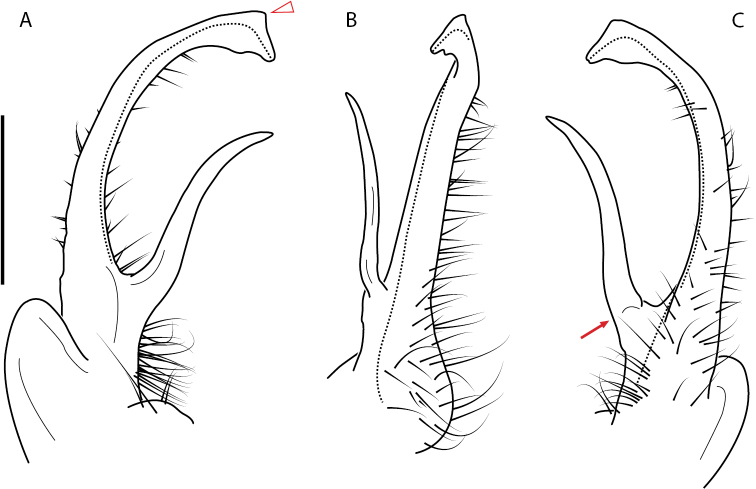
*Nannariadilatata* (Hennen & Shelley, 2015) paratype ♂ (NCSM, NCSM27945) left gonopod **A** anterior view; red triangle indicates acropodite tip lateral flange **B** medial view **C** posterior view; red arrow indicates reduced prefemoral spine. Scale bar: 0.5 mm.

**Figure 79.**
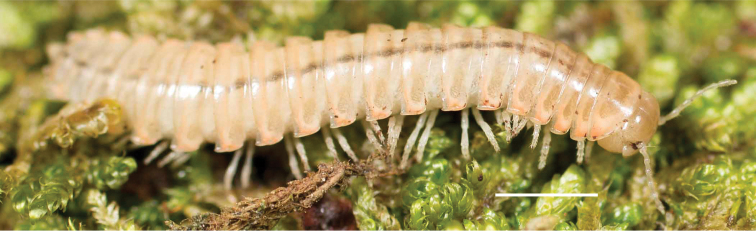
*Nannariadilatata* (Hennen & Shelley, 2015) non-type ♀ (VTEC, MPE02788) coloration. Scale bar: 4.0 mm.

Figs 78 , 79 Vernacular name: “The Mossy Rock Twisted-Claw Millipede”

Mimuloria dilatata dilatata Hennen & Shelley, 2015: 1–16, figs 18, 19.
Nannaria dilatata : Means et al. 2021: S69.

####### Material examined.

***Holotype***: United States – **Tennessee** • ♂; Marshall County, Henry Horton State Park, campground; [35.5875°N, -86.7035°W]; 9 May 1979; R. M. Shelley leg.; FSCA.

***Paratype***: United States – **Tennessee** • 1 ♀; same collection data as holotype; NCSM NCSM27945.

####### Other material.

United States – **Tennessee** • 1 ♀; Marshall County, Henry Horton State Park, to the right of the main office, under moss on top of large boulder; 35.5914°N, -86.7029°W; 13 May 2017; hand collected; D. Hennen, J. Means, V. Wong leg.; VTEC MPE02788. For detailed collection data see Suppl. material 7.

####### Diagnosis.

Adult males of *N.dilatata* are distinct from other *Nannaria*, including the nearby *N.hippopotamus* sp. nov. and *Nannaria* sp. nov. ‘Cratagae’ (*wilsoni* species group) based on the following combination of characters: **Gonopods**. Gonopodal acropodite gently curving anteromedially, not straight as in *N.hippopotamus* sp. nov. and *Nannaria* sp. nov. ‘Cratagae.’ Acropodite tip with prominent triangular lateral flange curving abruptly at a 90° angle towards tip (Fig. 78A, red triangle), not curving gently and rounded as in *N.hippopotamus* sp. nov. or lacking as in *Nannaria* sp. nov. ‘Cratagae.’ Acropodite with laminate medial flange just proximal to tip, not lacking as in *N.hippopotamus* sp. nov. and *Nannaria* sp. nov. ‘Cratagae.’ Acropodite simple, without medial swelling as in *N.hippopotamus* sp. nov. Telopodite basal zone ca. ¼ length of acropodite, not ca. ½ as in *N.hippopotamus* sp. nov. Prefemur with dorsomedially curving prefemoral process, not straight, acicular as in *N.hippopotamus* sp. nov. and *Nannaria* sp. nov. ‘Cratagae.’ Prefemoral spine reduced to small rounded lobe at base of prefemoral process (Fig. 78, red arrow), not sharp as in *N.hippopotamus* sp. nov. or lacking as in *Nannaria* sp. nov. ‘Cratagae.’ **Color**. Tergites with light orange paranotal spots and light pink stripes (Fig. 79). Light grey background. Dorsum of collum smooth with light pink margin.

####### Measurements.

♂ holotype (FSCA): BL = 30.9, CW = 4.1, IW = 2.1, ISW = 0.8, B11W = 4.7, B11H = 3.5; ♀ paratype (NCSM, NCSM27945): BL = 28.1, CW = 3.5, IW = 2.0, ISW = 0.9, B11W = 4.5, B11H = 2.9.

####### Variation.

Hennen and Shelley (2015) noted some slight variation between the type locality males and the male collected from Davidson Co., Tennessee, including the latter having a smaller prefemoral spine, a more gradual distal curve of the acropodite, and a reduced acropodite medial swelling.

####### Distribution.

Known only from central Tennessee (Tennessee: Davidson and Marshall counties, Fig. 129). Distribution area: N/A; status: MRE.

####### Ecology.

Hennen and Shelley (2015) provided no ecological notes in their description of *N.dilatata*, but the single individual collected by DAH for this revision was found under a damp mat of moss on a large boulder.

####### Etymology.

Hennen and Shelley (2015: 14) state “The specific name references the apical dilation on the outer/anterior acropodite surface.”

####### Type locality.

United States, Tennessee, Marshall County, Henry Horton State Park, campground.

####### Notes.

In the original publication, Hennen and Shelley (2015, 14) designated a male holotype (FSCA) and one male and two female paratypes (FSCA, NCSM), all collected by R. M. Shelley on May 9, 1979. Which paratypes were sent to either the FSCA or NCSM was not mentioned in the original publication; however, the NCSM had only one paratype (female, NCSM27945), implying that the other male and female paratypes are deposited at the FSCA.

###### Nannaria fracta sp. nov.

Taxon classificationAnimaliaPolydesmidaXystodesmidae

37325C79-13A6-599C-90A3-3B988C3133EA

http://zoobank.org/8D8D39A0-7084-4BA9-8F47-9D0C7C99F55B

**Figure 80.**
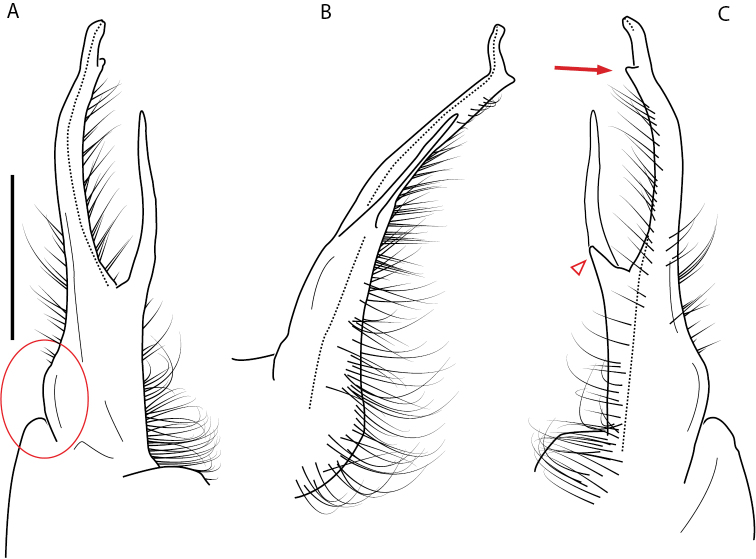
*Nannariafracta* sp. nov. paratype ♂ (VMNH, NAN0156) left gonopod **A** anterior view; red circle indicates pronounced basal swelling **B** medial view **C** posterior view; red arrow indicates shelf-like medial flange; red triangle indicates sharp, pronounced prefemoral spine. Scale bar: 0.5 mm.

**Figure 81.**
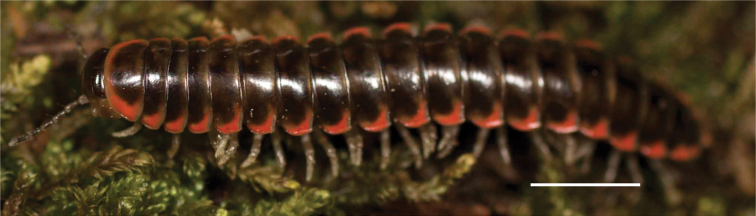
*Nannariafracta* sp. nov. paratype ♂ (VTEC, MPE03185) coloration. Scale bar: 4.0 mm.

Figs 80 , 81 Vernacular name: “The Breaks Interstate Park Twisted-Claw Millipede”

####### Material examined.

***Holotype***: United States – **Virginia** • ♂; Dickenson County, Haysi, Breaks Interstate Park, Laurel Branch Trail at intersection with Cold Spring Trail; 37.2897°N, -82.2999°W; elev. 565 m; 28 Sep. 2017; hand collected; J. Means, D. Hennen leg.; VTEC MPE031781.

***Paratypes***: United States – **Virginia** • ♂; same collection data as holotype; VTEC, MPE03179 • 1 ♂; same collection data as holotype; VMNH MPE03756 • 2 ♂♂; same collection data as holotype; VTEC MPE03183, 84; 1 ♂; Dickenson County, Breaks Interstate Park, in camping area; 37.2936°N, -82.3005°W; 16 Apr. 1983; D. Ogle leg.; VMNH NAN0156 • SCAU – **Kentucky** • 2 ♀♀; Pike County, Pikeville, Bob Amos Park, 424 Bob Amos Dr. WW Gearheart Hiking Trail; 37.4690°N, -82.5462°W; elev. 359 m; 28 Sep. 2017; hand collected; J. Means, D. Hennen leg.; VTEC MPE03185, 86.

####### Other material.

United States – **Virginia** • 1 ♂; Dickenson County, Breaks Interstate Park; 37.2936°N, -82.3005°W; 7 Sep. 1967; Neff, R. Hoffman leg.; VMNH NAN0154 • 1 ♂; Russell County, 1 mile NW of Lynn Spring; 37.1153°N, -81.9411°W; 20 Apr. 1962; R. Hoffman leg.; VMNH NAN0155 • 1 ♂; Washington County, Clinch Mountain Wildlife Management Area, deciduous forest above Big Tumbling Creek; 36.9931°N, -81.7368°W; 21 Sep. 2011; S. Roble leg.; VMNH NAN0157. For detailed collection data see Suppl. material 7.

####### Diagnosis.

Adult males of *Nannariafracta* sp. nov. are distinct from other *Nannaria* and the nearby *N.aenigma*, based on the following combination of characters: ***Gonopods*.** Gonopodal acropodite acicular, slightly curving medially before tip, not bending abruptly medially at 90° at tip as in *N.bobmareki* sp. nov. or gently curving medially as in *N.aenigma*. Distal zone short, rounded—not quadrate as in *N.bobmareki* sp. nov., or long and serpentine as in *N.aenigma*. Acropodite with small, shelf-like medial flange just before tip (Fig. 80C, red arrow). Prefemur with long, acicular prefemoral process with a pronounced, sharp prefemoral spine (Fig. 80C, red triangle), not fused with prefemoral process as in *N.bobmareki* sp. nov. or lacking as in *N.aenigma*. Telopodite basal zone > ½ length of acropodite, not ca. 1/2 length of acropodite as in *N.bobmareki* sp. nov. or ca. 1/6 length of acropodite as in *N.aenigma*. Telopodite basal zone basal zone with pronounced medial swelling (Fig. 80A, red circle), not with slight medial swelling as in *N.bobmareki* sp. nov., or lacking as in *N.aenigma*. ***Color***: Tergites with red paranotal spots (Fig. 81). Dark brown background. Dorsum of collum smooth with red margin.

####### Measurements.

♂ holotype (VTEC, MPE03178): BL = 28.9, CW = 3.7, IW = 1.8, ISW = 0.85, B11W = 4.4, B11H = 2.5; ♀ paratype (VTEC, MPE03185): BL = 35.0, CW = 4.5, IW = 2.7, ISW = 1.2, B11W = 5.6, B11H = 4.2.

####### Variation.

No known variation.

####### Distribution.

*Nannariafracta* sp. nov. has a linear distribution extending from eastern Kentucky into western Virginia (Kentucky: Pike County; Virginia: Dickenson, Russell, and Tazewell counties; Suppl. material 7, Fig. 126). Distribution area: 98 km^2^; status: MRE.

####### Ecology.

Individuals of *N.fracta* sp. nov. have been collected from mesic hardwood forests composed of beech, maple, tuliptree, hemlock, and rhododendron. Specimens taken from Bob Amos Park in Kentucky were found under 1–2 cm of hardpacked, dark soil on the side of a hiking path, while specimens collected from Breaks Interstate Park in Virginia were found in a very moist rhododendron cove along the bank of Laurel Branch Creek, under 1–2 cm of sandy, dark soil.

####### Etymology.

This species is named for Breaks Interstate Park, where it was originally collected by R. L. Hoffman in 1962. The specific name is an adjective derived from the Latin *fractura*, meaning break or fracture.

####### Type locality.

United States, Virginia, Dickenson County, Haysi, Breaks Interstate Park, Laurel Branch Trail at intersection with Cold Spring Trail, 37.2897°N, -82.2999°W.

###### Nannaria solenas sp. nov.

Taxon classificationAnimaliaPolydesmidaXystodesmidae

7992210F-5606-5662-8C8E-A9AA8A2A52D0

http://zoobank.org/D0A9A027-576C-4BB4-9B51-0EE1178377A7

**Figure 82.**
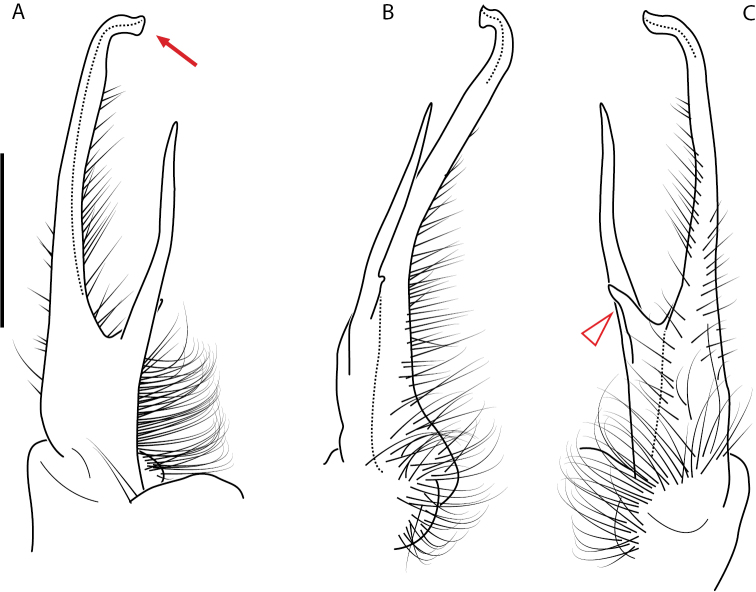
*Nannariasolenas* sp. nov. holotype ♂ (VTEC, MPE00128) left gonopod **A** anterior view; red arrow upturned acropodite tip **B** medial view **C** posterior view; red triangle indicates pronounced prefemoral spine. Scale bar: 0.5 mm.

**Figure 83.**
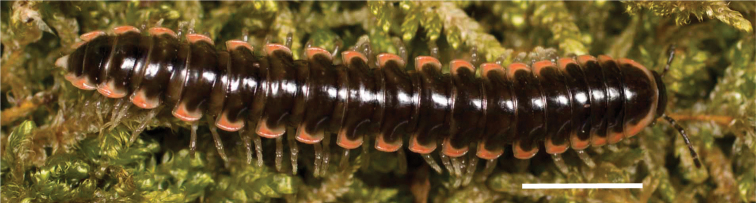
*Nannariasolenas* sp. nov. non-type ♂ (VTEC, MPE02420) coloration. Scale bar: 4.0 mm.

**Figure 84.**
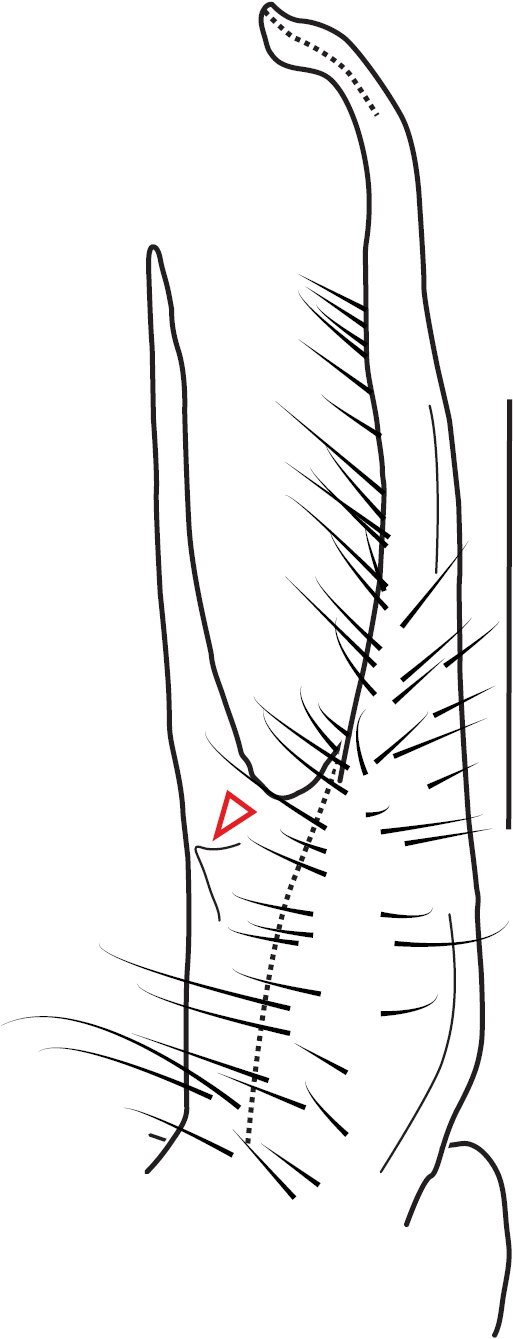
*Nannariasolenas* sp. nov. non-type ♂ (VTEC, MPE02420) from Crawfish Valley left gonopod; red triangle indicates reduced prefemoral spine.

Figs 82 , 83 , 84 Vernacular name: “The Pipestem Twisted-Claw Millipede”

####### Material examined.

***Holotype***: United States – **West Virginia** • ♂; Summers County, Pipestem Resort State Park, path to lake, ca. 12 km northeast of Athens; 37.5278°N, -80.9889°W; 835 m; 21 Aug. 2014; hand collected; J. Means, E. Francis leg.; VTEC MPE00128.

***Paratypes***: United States – **West Virginia** • 4 ♂♂; same collection data as holotype; VTEC MPE00130, 132–134 • 4 ♂♂; same collection data as holotype; VMNH, MPE00135, 137, 139, 142 • 3 ♀♀; same collection data as holotype; VMNH MPE00129, 131, 136 • 3 ♀♀; same collection data as holotype; VMNH MPE00138, 140, 141.

####### Other material.

United States – **Virginia** • 2 ♂♂; Hamilton’s Cave, ca. 4 mi. E Mechanicsburg; 37.1726°N, -80.8780°W; 11 Apr. 1967; Herpetology Class leg.; VMNH NAN0151 • 1 ♂; same collection data as preceding; 29 Apr. 1956; R. Hoffman leg.; VMNH NAN0177 • 19 ♂♂; Bland County, Hamilton’s Cave, 5 mi. ENE of Mechanicsburg; 37.1834°N, -80.8624°W; 20 Sep. 1967; Knight, Rushin, Liscombe, R. Hoffman leg.; VMNH NAN0322 • 5 ♂♂; Bland County, hillside outside Hamilton’s Cave, ca. 6 km east of Mechanicsville (presumably a typo of Mechanicsburg); 37.1468°N, -80.8727°W; 16 May 1980; R. Hoffman leg.; VMNH NAN0323 • 10 ♂♂; Bland County, Big Walker Mountain, on 3 mi/SE of Mechanicsville (presumably a typo of Mechanicsburg); 37.1307°N, -80.8916°W; 21 Oct. 1978; R. Hoffman leg.; VMNH NAN0220 • 10 ♂; Russell County, Pinnacles Nature Preserve, nr preserve tr.; 36.9533°N, -82.0550°W; elev. 625 m; 20 Aug. 2006; hand collected; P. Marek, C. Spruill leg.; VTEC MMC0201, 203–207, 209, 212, 214, 215 • 5 ♀♀; same collection data as preceding; VTEC MMC0200, 208, 210, 211, 213 • 1 ♂; Tazewell County, SE slope East River Mtn., near Cove Creek; 37.2060°N, -81.3070°W; 29 Mar. 1971; C. Chapman leg.; VMNH NAN0049 • 3 ♂♂; Tazewell County, Burkes Garden, e. slope of Beartown Mtn.; 37.0114°N, -81.6990°W; elev. 1219 m; 19 Feb. 1971; W. Shear leg.; VMNH NAN0328 • 1 ♂; Wythe County, Crawfish Valley, Channel Rock Hollow trail 1 mile from Strawberry Rd. end; 36.9526°N, -81.3247°W; elev. 772 m; 24 Mar. 2017; hand collected; C. Harden leg.; VTEC MPE02416 • 1 ♂; Wythe County, Crawfish Valley, Channel Rock Hollow trail 1.5 mile from Strawberry Rd. end; 36.9585°N, -81.3189°W; elev. 770 m; 24 Mar. 2017; hand collected; C. Harden leg.; VTEC MPE02428 • SCAU – **West Virginia** • 1 ♂; Fayette County, Fayette Station; 38.0672°N, -81.0834°W; 4 Oct. 1989; W. Arnold leg.; NCSM NAN0455 • 1 ♂; Mercer County, Athens, Jackson’s Park, Unity Rd.; 37.4267°N, -81.0402°W; 2 Oct. 1966; W. Shear leg.; VMNH NAN0031. For detailed collection data see Suppl. material 7.

####### Diagnosis.

Adult males of *Nannariasolenas* sp. nov. are distinct from other *Nannaria* and the sympatric *N.asta* sp. nov. and *N.aenigma*, based on the following combination of characters: ***Gonopods*.** Gonopodal acropodite straight, not gently curving throughout as in *N.asta* sp. nov. or *N.aenigma*. Distal zone short, rectangular, bent medially at 90° angle with acropodite with slight cephalically-directed upturn at terminal edge (Fig. 82A, red arrow), not rounded, directed caudally with lateral flange as in *N.asta* sp. nov., or sinuous as in *N.aenigma*. Acropodite simple with slight swelling on inner margin but lacking dimple on outer margin as in *N.asta* sp. nov., or hooked lateral flange as in *N.aenigma*. Prefemur with long, acicular prefemoral process, not stout as in *N.asta* sp. nov., or laterally curving as in *N.aenigma*. Prefemoral spine pronounced and tooth-like (Fig. 82C, red triangle), not cephalically-curving as in *N.asta* sp. nov. or lacking as in *N.aenigma*. Telopodite basal zone simple, ca. 1/2 length of acropodite, not with lateral bulge, < ½ length of acropodite as in *N.asta* sp. nov., or < 1/6 length of acropodite as in *N.aenigma*. ***Color*.** Tergites with orange paranotal spots (Fig. 83). Black background. Dorsum of collum smooth with orange and white margin.

####### Measurements.

♂ holotype (VTEC, MPE00128): BL = 30.8, CW = 4.1, IW = 2.1, ISW = 1.0, B11W = 5.0, B11H = 3.3; ♀ paratype (VMNH, MPE00140): BL = 34.7, CW = 4.3, IW = 2.5, ISW = 1.1, B11W = 5.5, B11H = 3.1.

####### Variation.

Individuals from Crawfish Valley, Wythe County, Virginia, have reduced, shelf-like prefemoral spines (Fig. 84, red triangle).

####### Distribution.

Known from southwestern Virginia and southeastern West Virginia, with an individual from central West Virginia (West Virginia: Fayette, Mercer, and Summers counties; Virginia: Bland, Giles, Russell, Tazewell, and Wythe counties, Suppl. material 7; Fig. 126). Distribution area: 3,009 km^2^; status: SRE.

####### Ecology.

Individuals of *Nannariasolenas* sp. nov. have been collected from mesic hardwood forests, dominated by oak, maple, and pine. Specimens from Crawfish Valley were collected at night while walking on top of grass in an overgrown road.

####### Etymology.

This species is named after its type locality, Pipestem Resort State Park in West Virginia. The specific name is a noun in apposition derived from the Greek *solínas*, meaning pipe.

####### Type locality.

United States, West Virginia, Summers County, Pipestem Resort State Park, path to lake, ca. 12 km northeast of Athens, 37.5278°N, -80.9889°W.

###### Nannaria spruilli sp. nov.

Taxon classificationAnimaliaPolydesmidaXystodesmidae

9A629F07-7F54-54F1-BCFB-637C85C9B829

http://zoobank.org/B60DEA74-0FFB-4C8E-AFC0-06769D39AA52

**Figure 85.**
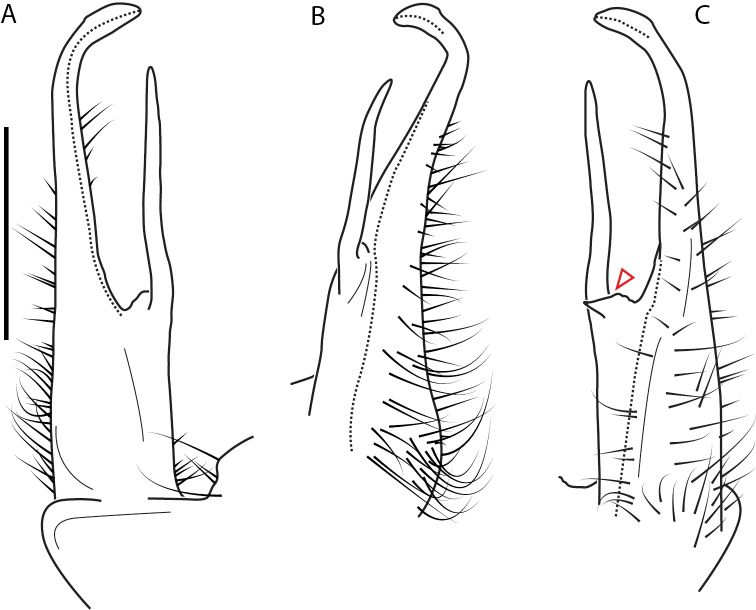
*Nannariaspruilli* sp. nov. holotype ♂ (VTEC, MMC0035) left gonopod **A** anterior view **B** medial view **C** posterior view; red arrow indicates small lateral flange; red triangle indicates reduced prefemoral spine. Scale bar: 0.5 mm.

Fig. 85 Vernacular name: “Chad Spruill’s Twisted-Claw Millipede”

####### Material examined.

***Holotype***: United States – **Virginia** • ♂; Wise County, Osborn Rock, FR238; 36.8949°N, -82.5902°W; elev. 1112 m; 17 Aug. 2006; hand collected; P. Marek & C. Spruill leg.; VTEC MMC0035.

***Paratype***: United States – **Virginia** • 1 ♀; same collection data as holotype; VTEC MMC0021 • 2 ♀♀; same collection data as holotype; VMNH MMC0027, 32. For detailed collection data see Suppl. material 7.

####### Diagnosis.

Adult males of *Nannariaspruilli* sp. nov. are distinct from other *Nannaria* and the nearby *N.aenigma*, based on the following combination of characters: ***Gonopods*.** Gonopodal acropodite straight, curving medially at nearly 90° angle at apex, not gently curving or with medial swelling as in *N.blackmountainensis* sp. nov. Acropodite tip blunt with small, lobed lateral flange (Fig. 85C, red arrow), not with large, hooked lateral flange as in *N.blackmountainensis* sp. nov., or thin, sinuous tip as in *N.aenigma*. Height of telopodite basal zone > 1/2 length of acropodite, not < ½ length as in *N.blackmountainensis* sp. nov., or ca. 1/6 length as in *N.aenigma*. Prefemur with straight, acicular prefemoral process and reduced prefemoral spine (Fig. 85C, red triangle). ***Color*.** Color in life unknown.

####### Measurements.

♂ holotype (VTEC, MMC0035): BL = 29.9, CW = 3.8, IW = 2.1, ISW = 0.8, B11W = 5.1, B11H = 3.1; ♀ paratype (VMNH, MMC0027; head taken for DNA): BL = N/A, CW = 4.1, IW = N/A, ISW = N/A, B11W = 5.4, B11H = 3.7.

####### Variation.

No known variation.

####### Distribution.

Known only from the type locality (Virginia: Wise County, Suppl. material 7; Fig. 126). Distribution area: N/A; status: MRE.

####### Ecology.

Individuals of *N.spruilli* sp. nov. were collected from mesic broadleaved forests from underneath deciduous leaf litter. *Nannariaspruilli* sp. nov. was encountered co-occurring with a Mullerian mimicry ring composed of the xystodesmid species *Apheloriapolychroma*, *Brachoriacedra* Keeton, 1959, and *Brachoriainsolita* Keeton, 1959 (Marek and Bond 2009).

####### Etymology.

This species was named after its co-collector, Chad Spruill. The specific name is a genitive noun derived as a patronym.

####### Type locality.

United States, Virginia, Wise County, Osborn Rock, FR238; 36.8949°N, -82.5902°W.

###### Nannaria terricola

Taxon classificationAnimaliaPolydesmidaXystodesmidae

(Williams & Hefner, 1928)

9155CABD-9608-5946-B002-D0A3AA3E9F56

**Figure 86.**
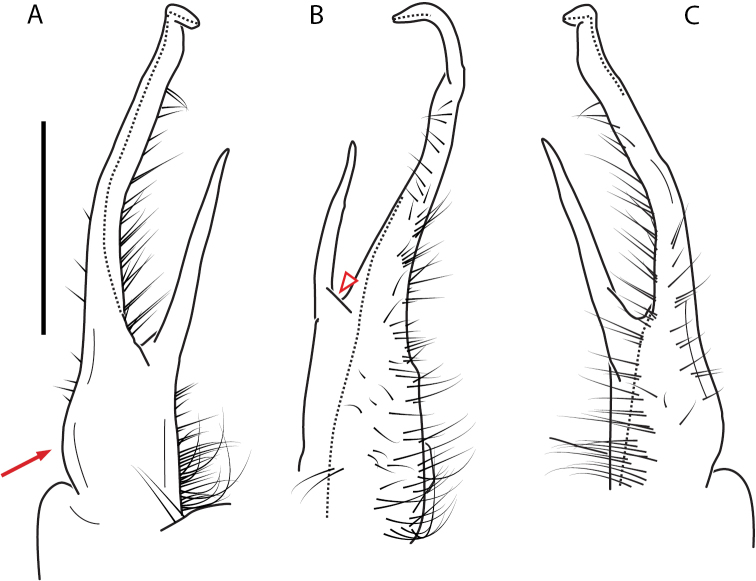
*Nannariaterricola* ♂ (VTEC, MPE02234) left gonopod **A** anterior view; red arrow indicates basal zone lateral bulge **B** medial view; red triangle indicates reduced, ridge-like prefemoral spine **C** posterior view. Scale bar: 0.5 mm.

**Figure 87.**
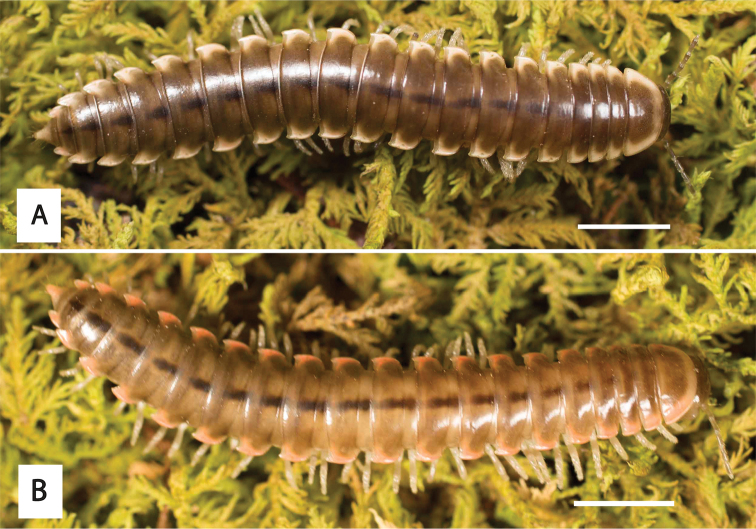
*Nannariaterricola* coloration **A** ♀ (VTEC, MPE01690) white paranota **B** ♀ (VTEC, MPE01691) pale orange paranota. Scale bars: 4.0 mm.

Figs 86 , 87 Vernacular name: “The Northern Ohio Twisted-Claw Millipede”

Fontaria terricola Williams & Hefner, 1928: 106, fig. 9c.
Nannaria terricola : Loomis and Hoffman 1948: 53. Chamberlin and Hoffman 1958: 42. Hoffman 1999: 368. Marek et al. 2014: 38. Means et al. 2021: S73.

####### Material examined.

***Syntypes***: United States – **Ohio** • 6 ♀♀; labeled as cotypes, Butler County, Oxford, Hueston’s Woods; [39.5800°N, -84.7600°W]; NMNH #2269.

####### Other material.

United States – **Ohio** • 1 ♂; Adams County, West Union, Edge of Appalachia Preserve, Abner Hollow Trail, not far up the trail, after the uphill walk; 38.7213°N, -83.4335°W; elev. 231 m; 16 June 2016; hand collected; J. Means, D. Hennen leg.; VTEC MPE03714 • 5 ♀♀; same collection data as preceding; VTEC MPE01721–24, 1738 • 9 ♂♂; Butler County, Oxford (39.5069°N, -84.7452°W), 1928, Coll: R. Hefner leg.; VMNH NAN0329 • 3 ♂; Harrison County, Hopedale, in old field; 40.3252°N, -80.9013°W; 29 Apr. 1979; R. Urbanek leg.; VMNH NAN0325 • 2 ♀♀; Hocking County, Crane Hollow Nature Preserve, in hollow behind Ellis House; 39.4913°N, -82.5797°W; elev. 290 m; 15 June 2016; hand collected; J. Means, D. Hennen leg.; VTEC MPE01690, 1691 • 3 ♂♂; Logan County, Bellefontaine, Fred Corker Park; 40.3640°N, -83.7330°W; elev. 435 m; 3 Nov. 2016; hand collected; J. Brown leg.; VTEC MPE02234, 2235, 2238 • 2 ♀♀; same collection data as preceding; VTEC MPE02236, 2237 • 1 ♂; Stark County, Stark Wilderness Center; 40.6720°N, -81.6420°W; 1 May 1971; W. Shear leg.; VMNH NAN0047. For detailed collection data see Suppl. material 7.

####### Diagnosis.

Adult males of *Nannariaterricola* are distinct from other *Nannaria*, the sympatric *N.ohionis*, and the nearby *N.shenandoa*, based on the following combination of characters: ***Gonopods*.** Gonopodal acropodite very slightly curving medially before apex, nearly straight, not strongly curving medially as in *N.shenandoa*, or obviously curving medially as in *N.ohionis*. Distal zone and tip short, simple, bent at 90° angle to acropodite and curving dorsally—not bending medially as in *N.ohionis*, or large, with flanges, curving posterolaterally as in *N.shenandoa*. Telopodite basal zone ca. 1/2 length of acropodite, not ca. 1/3 as in *N.ohionis*, or ca. 1/4 as in *N.shenandoa*. Telopodite basal zone with lateral bulge (Fig. 86A, red arrow), lacking in both *N.ohionis* and *N.shenandoa*. Prefemur with straight acicular prefemoral process, not curving laterally as in *N.shenandoa*. Prefemoral process arising from prefemur, not from top of prefemoral spine as in *N.ohionis*. Prefemoral spine reduced to small ridge, fused with prefemoral process (Fig. 86B, red triangle), not large, projecting, acicular as in *N.ohionis*. ***Color*.** Tergites with either white or pale orange paranotal spots (Fig. 87). Tan to dark brown background. Dorsum of collum smooth with either white or pale orange caudal margin, depending on color morph.

####### Measurements.

♀ syntype (NMNH, #2269): BL = 21.7 CW = 3.1, IW = 1.8, ISW = 0.9, B11W = 4.0, B11H = 3.1.

####### Variation.

No known variation.

####### Distribution.

Known from throughout central and southern Ohio (Ohio: Adams, Butler, Harrison, Hocking, Logan, Preble, and Stark counties; Suppl. material 7; Fig. 127). Distribution area: 33,812 km^2^; status: WRE.

####### Ecology.

Individuals of *N.terricola* have been collected from mesic hardwood forests composed of sycamore, maple, beech, cherry, hemlock, oak, birch, pawpaw, spicebush, and alder. They were often found under leaf litter and logs on hillsides, occasionally under 1–2 cm of dark soil.

####### Etymology.

Williams and Hefner (1928) gave no explanation for the name *terricola* in their description of the species, but it is assumed that it is derived from the Latin *terricolus*, terrestrial.

####### Type locality.

United States, Ohio, Butler County, Oxford, Hueston’s Woods; [39.5800°N, -84.7600°W].

####### Notes.

In the original publication, Williams and Hefner (1928: 106, 107) did not designate type specimens, but mentioned that adults were collected, implying the existence of a type series. Upon investigation of the type material deposited at the NMNH we found that six female syntypes existed (NMNH #2269). Where the male specimen which Williams and Hefner (1928) illustrated and described currently resides is unknown.

##### *ambulatrix* clade

**Components.***Nannariaambulatrix* sp. nov., *N.asta* sp. nov., *N.botrydium* sp. nov., *N.tsuga*, sp. nov., and female specimens from Warriors Path State Park, Tennessee and Brumley Gap, Virginia (Fig. 114, Suppl. material 2). Members of the *ambulatrix* clade share gonopodal characters, including a thin gonopod with a straight and tall basal zone, a prominent prefemoral spine, and a lobed or triangular lateral flange near the acropodite apex. For the minor species group, the *ambulatrix* clade is uniquely cohesive morphologically, genetically (average PP = 0.95) and geographically.

**Distribution.** the *ambulatrix* clade extends from southwestern Virginia into northeastern Tennessee (Fig. 114).

###### Nannaria ambulatrix sp. nov.

Taxon classificationAnimaliaPolydesmidaXystodesmidae

30E2A3CF-2A6A-535B-9604-ACCDEC4FE38A

http://zoobank.org/F4DFC180-0793-4435-9E2A-31BD35809325

**Figure 88.**
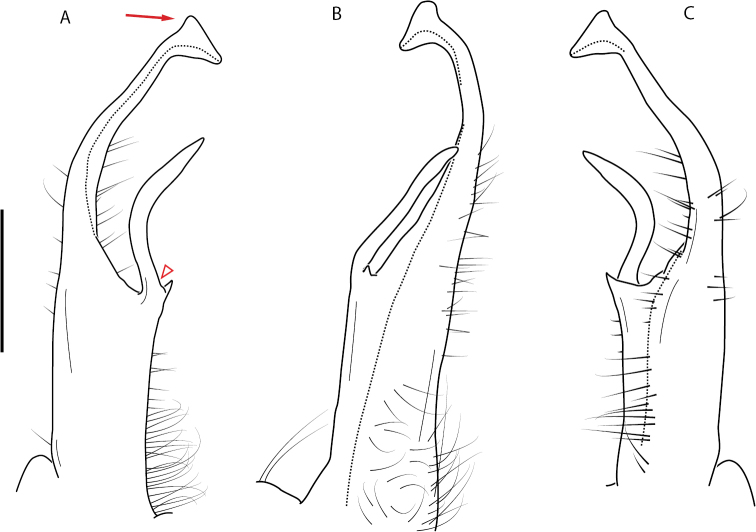
*Nannariaambulatrix* sp. nov. holotype ♂ left gonopod (MPE00178) **A** anterior view; red arrow indicates large, triangular lateral flange; red triangle indicates prefemoral process arising dorsomedially from prefemoral spine **B** medial view **C** posterior view. Scale bar: 0.5 mm.

**Figure 89.**
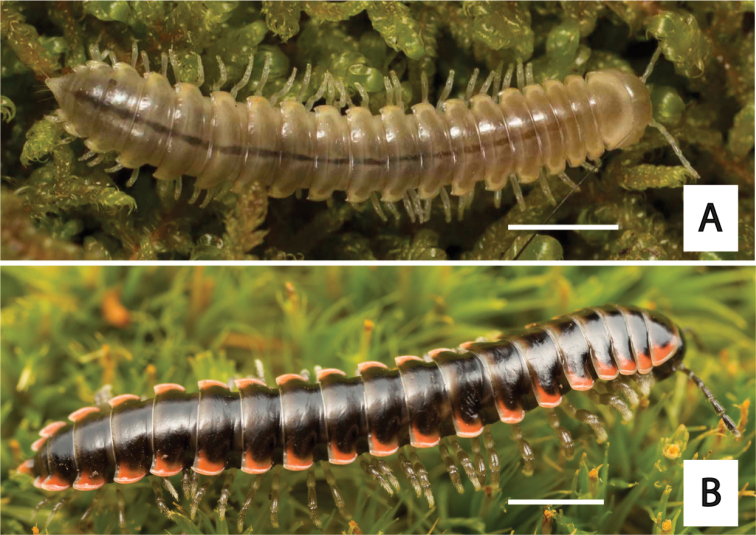
*Nannariaambulatrix* sp. nov. coloration **A** non-type ♀ (VTEC, MPE03795) white/tan paranota **B** paratype ♂ (VTEC, MPE00167) orange paranota. Scale bars: 4.0 mm.

Figs 88 , 89 Vernacular name: “The Big Walker Twisted-Claw Millipede”

####### Material examined.

***Holotype*:** United States – **Virginia** • ♂; Smyth County, west slope of Big Walker Mtn., VA-16; 36.9112°N, -81.5317°W; elev. 1054 m; 9 Sept. 2014, J. Means leg.; hand collected; VTEC MPE00178.

***Paratypes*:** United States – **Virginia** • 1 ♀; same data as for holotype; VTEC, MPE00174 • 2 ♂; same data as for holotype; VTEC MPE00166, 167 • 2 ♂ and 1 ♀; same data as for holotype; VMNH, MPE00169, 179, 180.

####### Other material.

United States – **Virginia** • 1 ♂ Washington County, Mendota, steep hillside beside intersection of CR 614 and CR 620, near Holston River; 36.7146°N, -82.2806°W; elev. 381 m; 12 Feb. 2018; hand collected J. Means, D. Hennen, P. Marek leg.; VTEC MPE03789 • 1 ♂; Washington County, Mendota, Anderson Rd.; 36.7219°N, -82.2771°W; elev. 464 m; 11 Feb. 2017; J. Means, D. Hennen, V. Wong leg.; hand collected; VTEC MPE02286 • 1 ♂ Washington County, Mendota, VA-620, ca.1 road km NE jct w/VA-614; 36.7192°N, -82.2775°W; elev. 287 m; 12 Feb. 2018; J. Means, D. Hennen, P. Marek leg.; hand collected; VTEC MPE04240 • 3 ♀; same collection data as for preceding; VTEC MPE03797, 3799, 4270 • 5 ♂♂; same collection data as for preceding; VTEC MPE03794, 3800-3803 • 2 ♀♀; Washington County, Mendota, Fugate Gap; 36.7285°N, -82.3026°W; elev. 609 m; 12 Feb. 2018; hand collected; J. Means, D. Hennen, P. Marek leg.; VTEC MPE03795, 3804 • 1 ♂ Washington County, DF site off VA. 620, 1 km E of Mendota; 36.7108°N, -82.2904°W; 11 June 1998; VMNH Survey leg.; VMNH NAN0137 • 1 ♂; Washington County, DF site off VA. 620, ca. 2 mi NE of Mendota; 36.7313°N, -82.2760°W; 8 Aug.–2 Dec. 1997;VMNH Survey leg.; VMNH NAN0307 • 1 ♂; Washington County, Abram’s Fall, ca. 2 mi SW of Benhams, end of VA. 614; 36.6580°N, -82.2439°W; 15 June 1960; R. Hoffman leg.; VMNH NAN0308 • 1 ♂; Smyth County, Hungry Mother State Park, inside Hemlock Haven conference center; 36.8914°N, -81.5249°W; 5 Aug. 2009; J. Beard leg.; VMNH NAN0124 • 1 ♂; Smyth County, Big Walker Mountain, ca. 3 mi west of Hungry Mother State Park on VA. 16, west slope; 36.9104°N, -81.5333°W; 9 Sept. 1956; R. Hoffman leg.; VMNH NAN0310. For detailed collection data see Suppl. material 7.

####### Diagnosis.

Adult males of *Nannariaambulatrix* sp. nov. are distinct from other *Nannaria* and the co-occurring *N.aenigma*, based on the following combination of characters: ***Gonopods*.** Gonopodal acropodite very slightly curving medially before apex, nearly straight (Fig. 88). Distal zone short, directed dorsomedially, with large, triangular lateral flange (Fig. 88A, red arrow), not simple as in *N.terricola*, or serpentine as in *N.aenigma*. Telopodite basal zone large, subequal to length of acropodite, not ca. 1/2 length as in *N.terricola*, or ca. 1/6 length as in *N.aenigma*. Telopodite basal zone straight, without lateral bulge as in *N.terricola*. Prefemur with medially curving prefemoral process, not acicular, straight as in *N.terricola* and *N.aenigma*. Prefemoral process arising dorsomedially from small, sharp, prefemoral spine (Fig. 88A, red triangle), not arising from prefemur or with spine reduced to a shelf-like ridge as in *N.terricola*. ***Color*.** Tergites with either white/light yellow or orange paranotal spots (Fig. 89). Gray to black background. Dorsum of collum smooth with either white/light yellow or orange margin, depending on color morph.

####### Measurements.

♂ holotype (VTEC, MPE00178): BL = 28.0, CW = 4.3, IW = 2.2, ISW = 1.0, B11W = 5.0, B11H = 3.4; ♀ paratype (VTEC, MPE00174): BL = 32.3, CW = 4.3, IW = 2.5, ISW = 0.9, B11W = 5.6, B11H = 3.7.

####### Variation.

Specimens of *Nannariaambulatrix* sp. nov. collected from the top of Clinch Mountain (36.7285°N, -82.3026°W, Elev. 845 m) were noticeably smaller than those collected from other localities, suggesting a possible adaptation in the higher elevation population to a drier and/or less suitable habitat. This is the only instance of a stark difference in size between populations of the same species that we have observed in *Nannaria*.

####### Distribution.

Known only from southwestern Virginia (Russell, Smyth and Washington counties; Suppl. material 7; Fig. 126). The distribution of *N.ambulatrix* sp. nov. follows the Brumley and Little Brushy Mountain ranges. Distribution area: 488 km^2^; status: MRE.

####### Ecology.

*Nannariaambulatrix* sp. nov. individuals were collected from mesic broadleaf deciduous forests, often in groups occupying 1–2 m^2^ patches, suggesting that the species is distributed in a highly aggregated manner.

####### Etymology.

This species is named for its type locality (Big Walker Mountain), and was informally proposed by R.L. Hoffman. The specific name is a noun in apposition derived from the Latin *ambulo*, to walk, and *trix*, ‘a female doer of an action.’

####### Type locality.

United States, Virginia, Smyth County, west slope of Big Walker Mtn., VA-16, 36.9112°N, -81.5317°W.

###### Nannaria asta sp. nov.

Taxon classificationAnimaliaPolydesmidaXystodesmidae

54CA8E7C-1ED3-5D75-B237-423F77AA4F02

http://zoobank.org/79F53717-C02F-4A97-825F-F2F7F5C8D474

**Figure 90.**
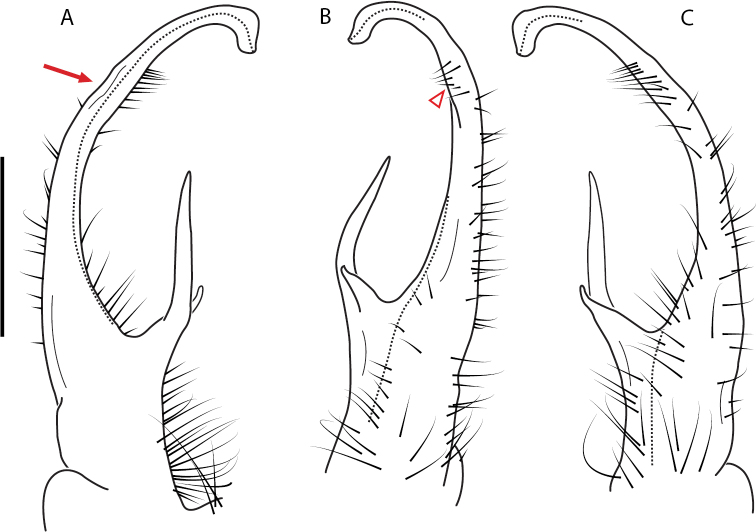
*Nannariaasta* sp. nov. holotype ♂ left gonopod (VTEC, MPE02419) **A** anterior view; red arrow indicates outer margin dimple **B** medial view; red triangle indicates slightly swollen inner margin **C** posterior view. Scale bar: 0.5 mm.

**Figure 91.**
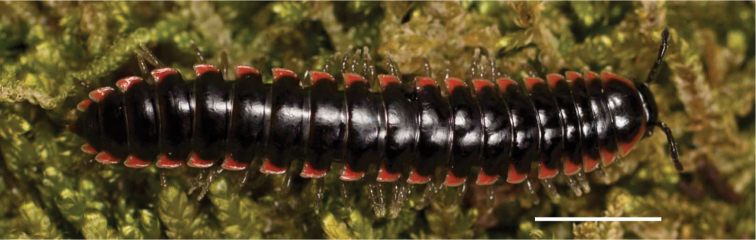
*Nannariaasta* sp. nov. holotype ♂ (VTEC, MPE02419) coloration. Scale bar: 4.0 mm.

Figs 90 , 91 Vernacular name: “The Crawfish Valley Twisted-Claw Millipede”

####### Material examined.

***Holotype*:** United States – **Virginia** • ♂; Wythe County, Crawfish Valley, Channel Rock, ca. 1.5 mi down trail from Strawberry Rd. end; 36.9585°N, -81.3189°W; elev. 770 m; 24 Mar. 2017; hand collected; C. Harden leg.; VTEC MPE02419.

***Paratypes*:** United States – **Virginia** • 3 ♂♂; Wythe Co. Crawfish Valley; 36.9811°N, -81.2934°W; elev. 715 m; 24 Mar. 2017; C. Harden leg.; VTEC MPE02421, VMNH, MPE02429, 2430. For detailed collection data see Suppl. material 7.

####### Diagnosis.

Adult males of *Nannariaasta* sp. nov. are distinct from other *Nannaria* and the sympatric *N.solenas* sp. nov. and *N.aenigma* based on the following combination of characters: ***Gonopods*.** Gonopodal acropodite gently curving medially, not straight as in *N.solenas* sp. nov. Distal zone curving dorsomedially, tip rounded, directed caudally with small lateral flange (Fig. 90A), not rectangular, directed medially at 90° angle with acropodite and slight cephalically-directed upturn at terminal edge as in *N.solenas* sp. nov., and not sinuous, without flange as in *N.aenigma*. Acropodite with slight swelling on inner margin (Fig. 90B, red triangle) and dimple on outer margin (Fig. 90A, red arrow). Prefemur with stout, acicular prefemoral process, arising dorsomedially from projected, cephalically-curving prefemoral spine, not arising from prefemur as in *N.solenas* sp. nov. Telopodite basal zone with small lateral bulge, lacking in *N.solenas* sp. nov. ***Color*.** Tergites with orange paranotal spots (Fig. 91). Black background. Dorsum of collum smooth with orange margin.

####### Measurements.

♂ holotype (VTEC, MPE02419): BL = 26.2, CW = 4.1, IW = 2.1, ISW = 0.9, B11W = 4.9, B11H = 3.1.

####### Variation.

No known variation.

####### Distribution.

Known only from southwestern Virginia (Wythe and Bland counties; Suppl. material 7; Fig. 126). The distribution of *N.asta* sp. nov. is restricted to the Crawfish Valley area and south of Wytheville, Virginia. Distribution area: 171 km^2^; status: MRE.

####### Ecology.

The majority of *Nannariaasta* sp. nov. individuals were found at night, walking along the side of a path on top of predominantly pine litter, a notably odd behavior for *Nannaria*, which typically remain beneath the leaf litter and are more cryptic in their behavior.

####### Etymology.

The specific epithet is an arbitrary combination of letters derived from the Infraorder Astacidea (which includes crayfish). It is to be treated as a noun in apposition.

####### Type locality.

United States, Virginia, Wythe County, Crawfish Valley, Channel Rock, ca. 1.5 mi down trail from Strawberry Rd. end, 36.9585°N, -81.3189°W.

###### Nannaria botrydium sp. nov.

Taxon classificationAnimaliaPolydesmidaXystodesmidae

B07EB7F2-D5DA-5209-B208-D789AA5F0E57

http://zoobank.org/AA57E2AC-54E7-4B80-B9E0-44EE3F1038EC

**Figure 92.**
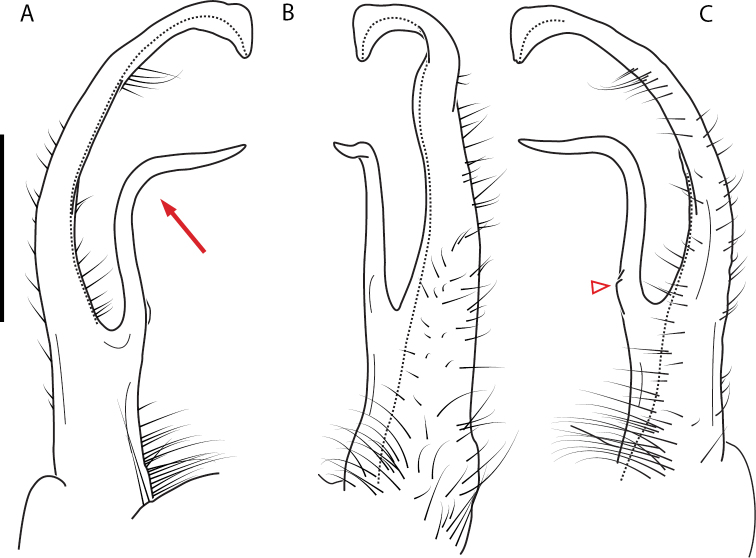
*Nannariabotrydium* sp. nov. holotype ♂ (VTEC, MPE00993) left gonopod **A** anterior view; red arrow indicates 90° bend in prefemoral process **B** medial view **C** posterior view; red triangle indicates reduced, prefemoral spine fused with prefemoral process. Scale bar: 0.5 mm.

**Figure 93.**
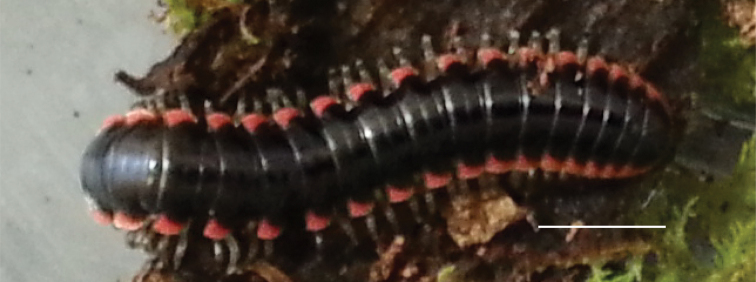
*Nannariabotrydium* sp. nov. holotype ♂ (VTEC, MPE00993) coloration. Scale bar: 4.0 mm.

Figs 92 , 93 Vernacular name: “The Grapefield Road Twisted-Claw Millipede”

####### Material examined.

***Holotype*:** United States – **Virginia** • ♂; Bland County, 2.6 km west of Hicksville; 37.1892°N, -81.1633°W; elev. 738 m; 12 Feb. 2016; hand collected; D. Hennen, P. Marek leg.; VTEC MPE00993.

***Paratypes*:** United States – **Virginia** • 1 ♂; Bland County, 2.7 km west of Hicksville; 37.1913°N, -81.165°W; elev. 754 m; 12 Feb. 2016; hand collected; D. Hennen, P. Marek leg.; VMNH MPE00992 • 1 ♀; Bland County, 4.2 km northwest of Hicksville; 37.19314°N, -81.18221°W; elev. 900 m; 2 Mar. 2016; hand collected; J. Means, P. Marek leg.; VTEC MPE01014.

####### Other material.

United States – **Virginia** • 1 ♂; Bland County; 37.1940°N, -81.1733°W, elev. 823 m; 2 Mar. 2016; hand collected; J. Means, P. Marek, Tyler leg.; VTEC MPE01012 • 1 ♂; Bland County; 37.1937°N, -81.1739°W; elev. 815 m; 2 Mar. 2016; hand collected; J. Means, P. Marek, Tyler leg.; VTEC MPE01013 • 2 ♂♂; Bland County; 37.1930°N, -81.1827°W; elev. 905 m; 2 Mar. 2016; hand collected; J. Means, P. Marek, Tyler leg.; VTEC MPE01009, 1011 • 1 ♂; Bland County; 37.1830°N, -81.1607°W; elev. 675 m; 7 Mar. 2016; hand collected; P. Marek, V. Wong, Tyler leg.; VTEC MPE01016 • 1 ♂ and 1 ♀; Bland County; 37.1234°N, -81.1355°W; elev. 889 m; 7 Apr. 2016; hand collected; J. Means, P. Marek, A. Prewitt, Tyler, Renea leg.; VTEC MPE03721, 3722 • 4 ♂; Tazewell County, Summit of East River Mtn., above Bluefield, West Virginia; 37.2112°N, -81.3164°W; elev. 914–1067 m; 9 Sep.1972; W. Shear leg.; VMNH NAN0015 • 2 ♂♂; Tazewell County, SE slope of East River Mountain near Cove Creek; 37.2067°N, -81.3072°W; 29 Mar. 1973; C. Chapman leg.; VMNH NAN0043; SCAU – **West Virginia** • 1 ♂; Mercer County, just over the West Virginia state line on 460, ca. 2 mi. SW of Glen Lyn on 219/8; 37.3452°N, -80.9105°W; elev. 570 m; 12 Nov. 2017; hand collected; J. Means; VTEC MPE03925 • 1 ♂; Randolph County, Cheat Bridge, TNC Preserve, Showers Fk at US 250; 38.6166°N, -79.8707°W; elev. 1174 m; 8 Oct. 2000; W. Arnold leg.; VMNH NAN0081. For detailed collection data see Suppl. material 7.

####### Diagnosis.

Adult males of *Nannariabotrydium* sp. nov. are distinct from other *Nannaria* and the sympatric *N.aenigma*, based on the following combination of characters: ***Gonopods*.** Gonopodal acropodite gently curving medially before apex, with lateral flange on tip, not thin and undulating as in *N.aenigma*. Acropodite tip directed caudally. Acropodite without medial swelling as in *N.tenuis* sp. nov. Telopodite basal zone > 1/3 length of acropodite, not ca. 1/6 as in *N.aenigma*. Prefemur with long, thin prefemoral process, bent 90° and directed medially at half-way point (Fig. 92A, red arrow)—not nearly linear as in *N.tenuis* sp. nov., or straight as in *N.aenigma*. Prefemoral spine reduced and fused for entire length with prefemoral process (Fig. 92C, red triangle), not lacking as in *N.aenigma* and *N.tenuis* sp. nov. ***Color*.** Tergites with bright red paranotal spots (Fig. 93). Black background. Dorsum of collum smooth with red margin.

####### Measurements.

♂ holotype (VTEC, MPE00993): BL = 27.0, CW = 3.8, IW = 1.9, ISW = 0.8, B11W = 4.7, B11H = 3.0; ♀ paratype (VTEC, MPE01014): BL = 26.7, CW = 3.2, IW = 1.9, ISW = 0.9, B11W = 3.9, B11H = 2.8.

####### Variation.

No known variation.

####### Distribution.

Known only from a small area (< 10 km^2^) west of Hicksville, Virginia (Virginia: Bland and Tazewell counties; Suppl. material 5; Fig. 126. A single male is recorded from Randolph County, West Virginia (ca. 200 km away from type locality), but as this is such a disjunct record, the authors are treating this specimen as an *N.botrydium* sp. nov. with an incorrect locality label (marked with an asterisk in Fig. 127). Distribution area: 10 km^2^; status: MRE.

####### Ecology.

Individuals of *N.botrydium* sp. nov. were collected from mesic hardwood forests composed of oak, maple, and rhododendron, found under leaf litter and occasionally 1–2 cm in dark soil.

####### Etymology.

This species is named after Grapefield Road, which runs near the type locality. The specific name is a Latinized diminutive of the Greek *botrys*, meaning a cluster of grapes.

####### Type locality.

United States, Virginia, Bland County, 2.6 km west of Hicksville, 37.1892°N, -81.1633°W.

###### Nannaria tsuga sp. nov.

Taxon classificationAnimaliaPolydesmidaXystodesmidae

67371BE7-4D3A-5673-B12B-63684DB966D9

http://zoobank.org/96611C3A-FC68-48FB-8EA4-9B241C3C3D53

**Figure 94.**
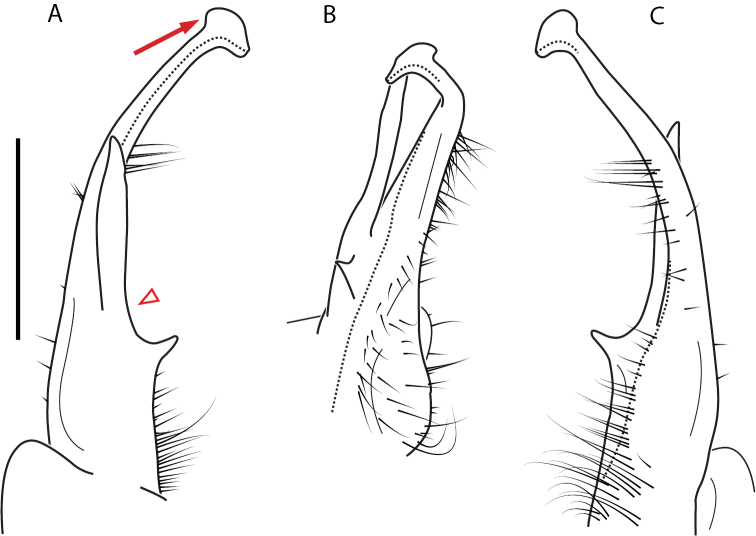
*Nannariatsuga* sp. nov. holotype ♂ (VTEC, MPE04047) left gonopod **A** anterior view; red arrow indicates large, lobed lateral flange; red triangle indicates prefemoral process arising distally from prefemoral spine **B** medial view **C** posterior view. Scale bar: 0.5 mm.

**Figure 95.**
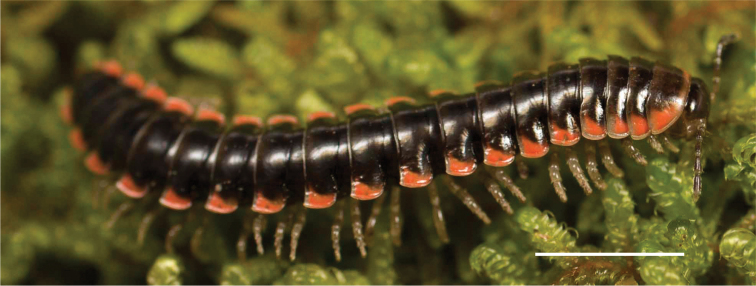
*Nannariatsuga* sp. nov. holotype ♂ (VTEC, MPE04047) coloration. Scale bar: 4.0 mm.

Figs 94 , 95 Vernacular name: “The Hemlock Grove Twisted-Claw Millipede”

####### Material examined.

***Holotype*:** United States – **Tennessee** • ♂; Sullivan County, Bristol, Steele Creek Park, along Hemlock Hollow Trail; 36.5703°N, -82.2356°W; elev. 506 m; 11 June 2018; hand collected; D. Hennen leg.; VTEC MPE04047.

***Paratypes*:** United States – **Tennessee** • 2 ♂♂; same collection data as holotype; VTEC MPE04048, 4049 • 1 ♂; same collection data as holotype; VMNH MPE04311 • 1 ♀; same collection data as holotype; VTEC MPE04043 • 1 ♀; same collection data as holotype; VMNH MPE04050. For detailed collection data see Suppl. material 7.

####### Diagnosis.

Adult males of *Nannariatsuga* sp. nov. are distinct from other *Nannaria* and the nearby *N.aenigma*, based on the following combination of characters: ***Gonopods*.** Gonopodal acropodite gently curving medially before apex, not nearly straight as in *N.ambulatrix* sp. nov. Distal zone short, directed dorsomedially, with large, lobed lateral flange (Fig. 94A, red arrow), not with triangular lateral flange as in *N.ambulatrix* sp. nov., or without flange and serpentine as in *N.aenigma*. Telopodite basal zone height < 1/2 length of acropodite, not enlarged, ca. 1/2 length as in *N.ambulatrix* sp. nov., or reduced, ca. 1/6 length as in *N.aenigma*. Prefemur with straight, laminate prefemoral process, separated widely from projected, with blunt prefemoral spine (Fig. 94A, red triangle), prefemoral process not arising dorsomedially from small, sharp prefemoral spine as in *N.ambulatrix* sp. nov. ***Color*.** Tergites with orange paranotal spots (Fig. 95). Black background. Dorsum of collum smooth with orange caudal margin.

####### Measurements.

♂ holotype (VTEC, MPE04047): BL = 33.9, CW = 4.3, IW = 2.0, ISW = 1.0, B11W = 5.2, B11H = 3.0; ♀ paratype (VTEC, MPE04043): BL = 31.1, CW = 3.8, IW = 2.6, ISW – 1.1, B11W = 4.9, B11H = 3.8.

####### Variation.

No known variation.

####### Distribution.

Known only from the type locality (Tennessee: Sullivan County, Suppl. material 7; Fig. 126). Distribution area: N/A; status: MRE.

####### Ecology.

Individuals of *Nannariatsuga* sp. nov. were collected from a mesic mixed hardwood and hemlock forest, composed of, in addition to hemlock, oak, beech, and buckeye. Individuals were found under moist litter on a hillside.

####### Etymology.

This species is named for the habitat in which it was discovered, a hemlock grove. The specific name is derived from the name *Tsuga* Carrière, the genus containing hemlock trees, and is a noun in apposition.

####### Type locality.

United States, Tennessee, Sullivan County, Bristol, Steele Creek Park, along Hemlock Hollow Trail, 36.5703°N, -82.2356°W.

##### *minor* clade

**Components.***Nannariacryomaia* sp. nov., *N.daptria* sp. nov., *N.hippopotamus* sp. nov., *N.honeytreetrailensis* sp. nov., *N.kassoni* sp. nov., *N.minor*, *N.rhysodesmoides* (Hennen & Shelley, 2015), and multiple female specimens from northeastern Tennessee (Fig. 114; Suppl. material 2). The *minor* clade is fairly morphologically diverse, though all members have either a small medial flange near the acropodite tip, or a medial swelling before the acropodite apex. No specimens of *N.rhysodesmoides* were collected for this study; based on the close geographic proximity of *N.rhysodesmoides* to *N.cryomaia* sp. nov. and *N.kassoni* sp. nov., as well as the shared gonopodal characters of a sharp acropodite tip and the presence of a lateral flange on the acropodite apex (as seen in *N.kassoni* sp. nov.), we place *N.rhysodesmoides* within the *minor* clade. We had expected *N.kassoni* sp. nov., and *N.cryomaia* sp. nov., to form a clade, due to their close geographic proximity, and similarly expanded medial flange; however, they appear to be more closely related to other, less morphologically similar members of the *minor* clade. *Nannariacryomaia* sp. nov. is found only ~ 30 km from *N.kassoni* sp. nov., and yet is ~ 170 km away from *N.minor*, to which it is more closely related. Phylogeographic analyses may reveal that there have been multiple distributional expansions and retractions of the *minor* clade, leading to the interesting patterns of relatedness observed in this group.

**Distribution.** the *N.minor* clade extends from southwestern Virginia into northeastern Tennessee (Fig. 114).

###### Nannaria cryomaia sp. nov.

Taxon classificationAnimaliaPolydesmidaXystodesmidae

326C6E1A-2401-5310-AEF1-27FE38EC668E

http://zoobank.org/B5C5C054-0EAE-48AB-8422-CC516979A45E

**Figure 96.**
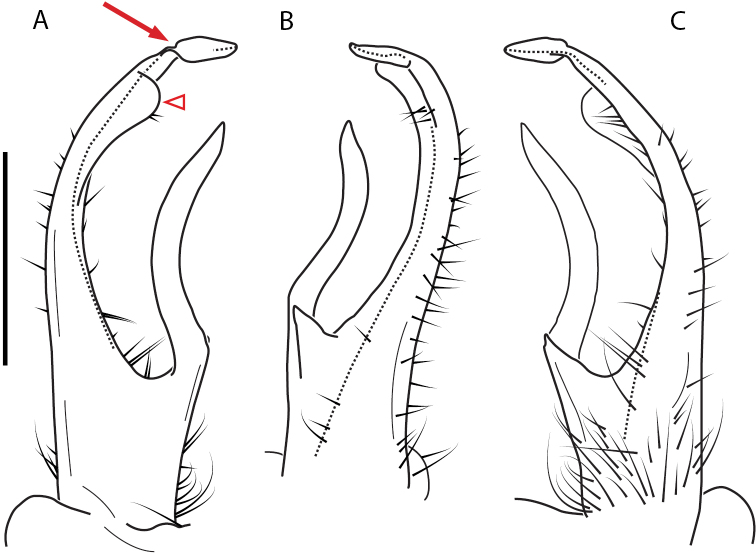
*Nannariacryomaia* sp. nov. holotype ♂ (VTEC, MPE02642) left gonopod **A** anterior view; red arrow indicates apical constriction; red triangle indicates medial flange **B** medial view **C** posterior view. Scale bar: 0.5 mm.

**Figure 97.**
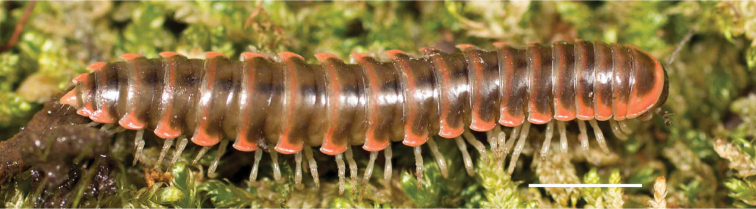
*Nannariacryomaia* sp. nov. paratype ♀ (VTEC, MPE02793) coloration. Scale bar: 4.0 mm.

Figs 96 , 97 Vernacular name: “The Frozen Head Twisted-Claw Millipede”

####### Material examined.

***Holotype*:** United States – **Tennessee** • ♂; Morgan County, campground at Frozen Head State Park; 36.1321°N, -84.4978°W; elev. 423 m; 12 May 2017; hand collected; J. Means, D. Hennen, V. Wong leg.; VTEC MPE02642.

***Paratypes*:** United States – **Tennessee** • 1 ♂; same collection data as holotype; VMNH MPE03765 • 2 ♀; same collection data as holotype; VTEC MPE02793, 94 • 2 ♀; same colletion data as holotype; VMNH MPE02795, 96.

####### Other material.

United States – **Tennessee** • 1 ♂; Morgan County, Frozen Head State Park; 36.1321°N, -84.4978°W; 29 May 1980; R. Shelley, MSM leg.; NCSM NAN0452. For detailed collection data see Suppl. material 7.

####### Diagnosis.

Adult males of *Nannariacryomaia* sp. nov. are distinct from other *Nannaria* and the nearby *Nannaria* sp. nov. ‘Cratagae’ (*wilsoni* species group) based on the following combination of characters: ***Gonopods*.** Gonopodal acropodite curving medially before apex, not straight as in *Nannaria* sp. nov. ‘Cratagae.’ Apex with distinct constriction (Fig. 96A, red arrow), tip expanded distally, with small lobed lateral and medial flanges, not with acuminate, triangular lateral flange as in *N.kassoni* sp. nov. Apex without ventrally directed bend as in *Nannaria* sp. nov. ‘Cratagae.’ Acropodite with expanded medial flange (Fig. 96A, red triangle). Telopodite basal zone height > 1/3 length of acropodite, not ca. 1/3 as in *N.kassoni* sp. nov., and not ca. 1/4 length as in *Nannaria* sp. nov. ‘Cratagae.’ Prefemur with medially curving prefemoral process, paralleling curve of acropodite, not straight, acicular as in *Nannaria* sp. nov. ‘Cratagae.’ Prefemur with short, triangular prefemoral spine, not pronounced, curving as in *N.kassoni* sp. nov. ***Color*.** Tergites with faint orange stripes (Fig. 97). Dark brown background. Dorsum of collum smooth with orange margin.

####### Measurements.

♂ holotype (VTEC, MPE02642): BL = 24.9, CW = 3.9, IW = 2.4, ISW = 0.9, B11W = 4.7, B11H = 3.2; ♀ paratype (VMNH, MPE02796): BL = 26.6, CW = 3.9, IW = 2.2, ISW = 1.0, B11W = 5.2, B11H = 3.7.

####### Variation.

No known variation.

####### Distribution.

Known only from the type locality (Tennessee: Morgan County; Suppl. material 7; Fig. 126). Distribution area: N/A; status: MRE.

####### Ecology.

Individuals of *Nannariacryomaia* sp. nov. were collected from a mesic hardwood forest dominated by oak, ironwood, beech, and hemlock, under moist leaf litter.

####### Etymology.

This species is named for its type locality, Frozen Head State Park in the Crab Orchard Mountains of eastern Tennessee. The specific name is a latinized noun in apposition derived from the Greek *kryos*-, meaning frozen, and *maia*- for a kind of crab.

####### Type locality.

United States, Tennessee, Morgan County, campground at Frozen Head State Park; 36.1321°N, -84.4978°W.

###### Nannaria daptria sp. nov.

Taxon classificationAnimaliaPolydesmidaXystodesmidae

08D2FCD1-A27F-507A-83AB-9A90B590BA62

http://zoobank.org/78C0F60F-71A6-4C4B-BD7B-6BD036C30277

**Figure 98.**
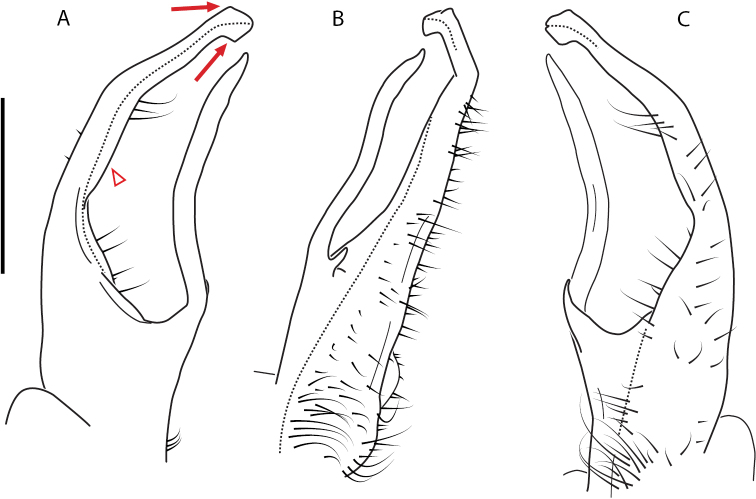
*Nannariadaptria* sp. nov. holotype ♂ (VTEC, MPE04156) left gonopod **A** anterior view; red arrows indicate medial and lateral flanges; red triangle indicates medial swelling **B** medial view **C** posterior view. Scale bar: 0.5 mm.

**Figure 99.**
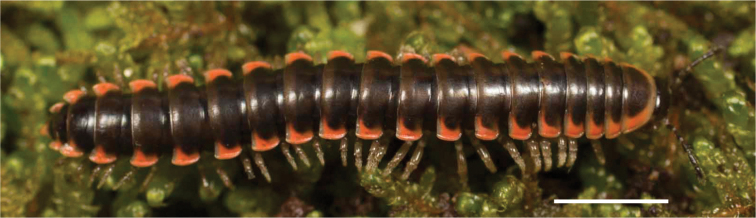
*Nannariadaptria* sp. nov. holotype ♂ (VTEC, MPE04156) coloration. Scale bar: 4.0 mm.

Figs 98 , 99 Vernacular name: “The Picnicking Twisted-Claw Millipede”

####### Material examined.

***Holotype*:** United States – **Tennessee** • ♂; Tennessee, Greene County, Paint Creek Corridor, Overlook Picnic Area, forest across road; 35.9777°N, -82.8478°W; elev. 500 m; 16 June 2018; hand collected; D. Hennen leg.; VTEC MPE04156. For detailed collection data see Suppl. material 7.

####### Diagnosis.

Adult males of *Nannariadaptria* sp. nov. are distinct from other *Nannaria* and the nearby *N.scutellaria*, based on the following combination of characters: ***Gonopods*.** Gonopodal acropodite gently curving medially throughout, not straight with 90° medial bend as in *N.scutellaria*. Tip blunt, with small, triangular lateral and medial flanges (Fig. 98A, red arrows), not simple as in *N.minor*. Acropodite swollen before apex (Fig. 98A, red triangle). Acropodite lacking tooth-like medial flange of *N.minor*, or acicular medial flange of *N.scutellaria*. Prefemur with long, thin, medially curving prefemoral process, not laterally curving as in *N.minor* and *N.scutellaria*. Prefemoral spine prominent, claw-like, curving cephalically, not small as in *N.minor*. Telopodite basal zone > 1/3 length of acropodite, not < 1/3 as in *N.scutellaria*. ***Color*.** Tergites with orange paranotal spots (Fig. 99). Light brown to black background. Dorsum of collum smooth with orange and white margin.

####### Measurements.

♂ holotype (VTEC, MPE04156): BL = 30.6, CW = 3.7, IW = 2.0, ISW = 0.8, B11W = 4.8, B11H = 3.4.

####### Variation.

No known variation.

####### Distribution.

Known only from the type locality (Tennessee: Greene County, Suppl. material 7; Fig. 126). Distribution area: N/A; status: MRE.

####### Ecology.

*Nannariadaptria* sp. nov. was collected from mesic, broadleaved forests composed of oak, maple, rhododendron, and hemlock, found under deciduous leaf litter in sandy soil.

####### Etymology.

This species was named for the relaxing lunch that directly preceded its discovery. The specific name is a noun in apposition derived from the Greek *daptria*, meaning ‘eater.’

####### Type locality.

United States, Tennessee, Greene County, Paint Creek Corridor, Overlook Picnic Area, forest across road; 35.9777°N, -82.8478°W.

###### Nannaria hippopotamus sp. nov.

Taxon classificationAnimaliaPolydesmidaXystodesmidae

57CAA5C1-803E-59B9-88AC-EA5926F98E8C

http://zoobank.org/AFE8AC4E-F463-4AA3-A9F6-4D0B4C16DCC9

**Figure 100.**
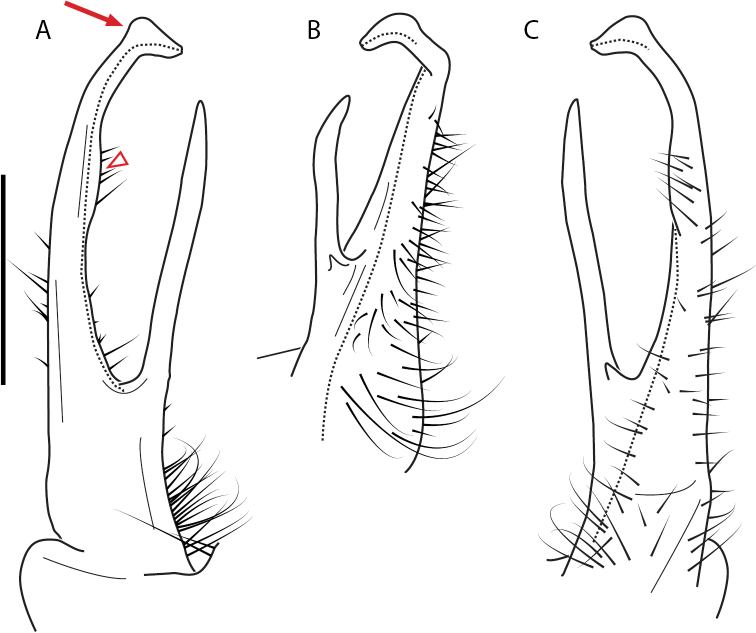
*Nannariahippopotamus* sp. nov. paratype ♂ (NCSM, NAN0469) left gonopod **A** anterior view; red arrow indicates lateral flange; red triangle indicates acropodite medial swelling **B** medial view **C** posterior view. Scale bar: 0.5 mm.

**Figure 101.**
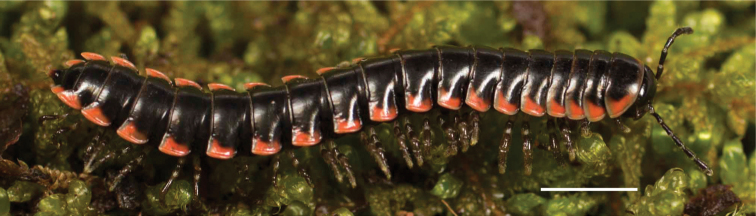
*Nannariahippopotamus* sp. nov. non-type ♂ (VTEC, MPE04141) coloration. Scale bar: 4.0 mm.

Figs 100 , 101 Vernacular name: “The Horse Creek Twisted-Claw Millipede”

####### Material examined.

***Holotype*:** United States – **Tennessee** • ♂; Greene County, Cherokee National Forest, Horse Creek Rec Area, picnic area on east side of Horse Creek; 36.1059°N, -82.6545°W; elev. 581 m; 16 June 2018; hand collected; D. Hennen leg.; VTEC MPE04150.

***Paratypes*:** United States – **Tennessee** • 3 ♀; same collection data as holotype; VTEC MPE04151–53 • 3 ♀♀; same collection data as holotype; VMNH MPE04154, 55, 4233 • 1 ♂; same collection data as holotype; 16 Oct. 1978; R. Shelley, W. Jones leg.; NCSM NAN0469.

####### Other material.

United States – **Tennessee** • 1 ♂; Greene County, 8.5 air km south of Horse Creek, Cherokee National Forest, Old Forge Recreation Area; 36.0878°N, -82.6811°W; elev. 623 m; 15 June 2018; hand collected; D. Hennen leg.; VTEC MPE04141 • 1 ♀; same collection data as preceding; VTEC MPE04142. For detailed collection data see Suppl. material 7.

####### Diagnosis.

Adult males of *Nannariahippopotamus* sp. nov. are distinct from other *Nannaria*, and the nearby *N.scutellaria*, based on the following combination of characters: ***Gonopods*.** Gonopodal acropodite straight, curving medially at apex. Acropodite tip with prominent lobed lateral flange (Fig. 100A, red arrow), curving dorsomedially, not simple as in *N.terricola*, or with tooth-like medial flange as in *N.scutellaria*. Acropodite swollen medially before apex (Fig. 100A, red triangle). Telopodite basal zone ca. 1/2 length of acropodite, not ca. 1/4 as in *N.scutellaria*. Telopodite basal zone with very slight lateral bulge, not prominent as in *N.terricola*. Prefemur with straight, acicular prefemoral process, not curving ventrally as in *N.scutellaria*. Prefemoral spine small, sharp, not fused with prefemoral process as in *N.terricola*. ***Color*.** Tergites with orange paranotal spots (Fig. 101). Black background. Dorsum of collum smooth with orange margin.

####### Measurements.

♂ holotype (VTEC, MPE04150): BL = 30.4, CW = 4.2, IW = 2.3, ISW = 1.0, B11W = 4.9, B11H = 3.3; ♀ paratype (VTEC, MPE04152): BL = 31.2, CW – 4.1, IW = 2.3, ISW = 1.0, B11W = 5.3, B11H = 4.0.

####### Variation.

No known variation.

####### Distribution.

Known only from two campsites in eastern Tennessee (Tennessee: Greene County; Suppl. material 5; Fig. 126). Distribution area: N/A; status: MRE.

####### Ecology.

Individuals of *Nannariahippopotamus* sp. nov. have been found under moist leaf litter in mesic broadleaved forests dominated by sweetgum, hickory, hemlock, oak, rhododendron, and maple.

####### Etymology.

This species is named for its type locality, Horse Creek, a national forest recreation area hardwood cove. The specific name is a noun in apposition derived from the Greek *hippos*-, meaning horse, and *potamós*, meaning river.

####### Type locality.

United States, Tennessee, Greene County, Cherokee National Forest, Horse Creek Rec Area, picnic area on east side, 36.1059°N, -82.6545°W.

###### Nannaria honeytreetrailensis sp. nov.

Taxon classificationAnimaliaPolydesmidaXystodesmidae

CD5A0D95-79AD-59AE-9EDD-AEBA7A393396

http://zoobank.org/90CC3A0F-C6AF-41BA-A4A7-CB7D94721204

**Figure 102.**
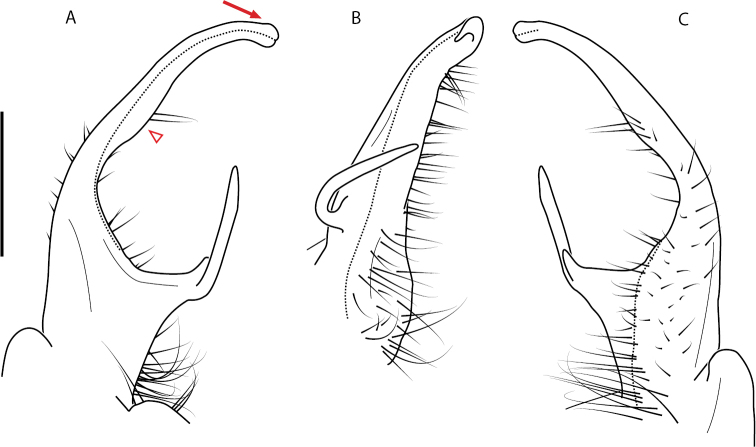
*Nannariahoneytreetrailensis* sp. nov. holotype ♂ (VTEC, MMC0334) left gonopod **A** anterior view; red arrow indicates medial flange; red triangle indicates medial swelling **B** medial view **C** posterior view. Scale bar: 0.5 mm.

**Figure 103.**
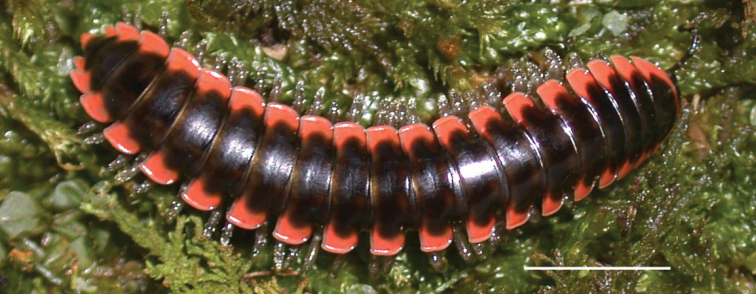
*Nannariahoneytreetrailensis* sp. nov. holotype ♂ (VTEC, MMC0334) coloration. Scale bar: 4.0 mm.

Figs 102 , 103 Vernacular name: “The Honey Tree Trail Twisted-Claw Millipede”

####### Material examined.

***Holotype*:** United States – **Virginia** • ♂; Lee County, Cumberland Gap National Historic Park, Honey Tree Trail, 0.2 km E junction with Gibson Gap Trail; 36.6078°N, -83.6322°W; 29 Sep. 2006; P. Marek leg.; VTEC MMC0334.

####### Diagnosis.

Adult males of *Nannariahoneytreetrailensis* sp. nov. are distinct from other *Nannaria* and the nearby *N.scutellaria*, based on the following combination of characters: ***Gonopods***: Gonopodal acropodite gently curving medially throughout, not straight with 90° medial bend as in *N.scutellaria*. Tip blunt, with small, triangular lateral flange (Fig. 102A, red arrow), lacking triangular medial flange of *N.daptria* sp. nov. Acropodite swollen basal to apex (Fig. 102A, red triangle), lacking acicular medial flange of *N.scutellaria*. Prefemur with short, acuminate, ventrally curving prefemoral process, not long, medially curving as in *N.daptria* sp. nov., or laterally curving as in *N.scutellaria*. Prefemoral process arising dorsolaterally from prominent, blunt prefemoral spine, not claw-like, as in *N.daptria* sp. nov. Telopodite basal zone height > 1/3 length of acropodite, not < 1/3 as in *N.scutellaria*. ***Color***: Tergites with large, hot orange/pink paranotal spots (Fig. 103). Black background. Dorsum of collum smooth with hot orange/pink margin.

####### Measurements.

♂ holotype (VTEC, MMC0334): BL = 35.5, CW = 4.5, IW = 2.6, ISW = 0.9, B11W = 5.5, B11H = 3.8.

####### Variation.

No known variation.

####### Distribution.

*Nannariahoneytreetrailensis* sp. nov. is known only from the type locality (Virginia: Lee County; Suppl. material 7, Fig. 126). Distribution area: N/A; status: MRE.

####### Ecology.

The *N.honeytreetrailensis* sp. nov. individual was collected from a mesic hardwood forest, on the side of a hiking path.

####### Etymology.

This species is named for its type locality. The specific name is a feminine adjective derived from the type locality.

####### Type locality.

United States, Virginia, Lee County, Cumberland Gap National Historic Park, Honey Tree Trail, 0.2 km E junction with Gibson Gap Trail, 36.6078°N, -83.6322°W.

###### Nannaria kassoni sp. nov.

Taxon classificationAnimaliaPolydesmidaXystodesmidae

652F7631-CCD3-54F4-91F9-5A92CBDF6784

http://zoobank.org/72902CE6-1FAF-46D7-8937-338D97E684BE

**Figure 104.**
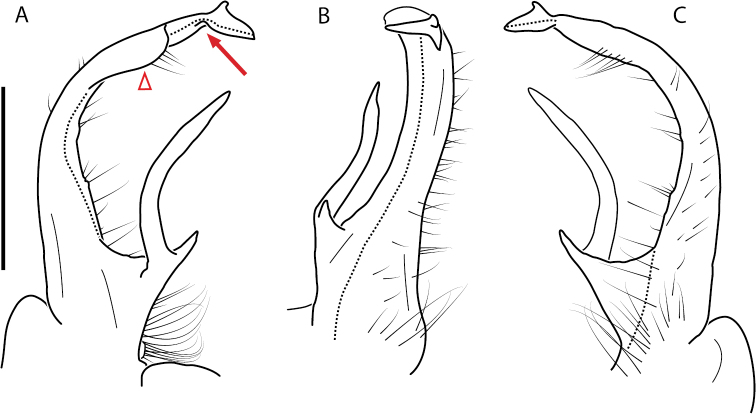
*Nannariakassoni* sp. nov. holotype ♂ (VTEC, MPE00544) left gonopod **A** anterior view; red arrow indicates apical constriction; red triangle indicates medial flange **B** medial view **C** posterior view. Scale bar: 0.5 mm.

**Figure 105.**
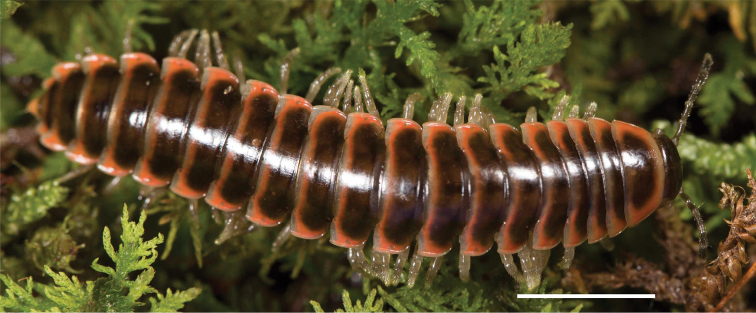
*Nannariakassoni* sp. nov. holotype ♂ (VTEC, MPE00544) coloration. Scale bar: 4.0 mm.

Figs 104 , 105 Vernacular name: “Kasson’s Twisted-Claw Millipede”

####### Material examined.

***Holotype*:** United States – **Tennessee** • ♂; Campbell County, Caryville, along Cove Creek Trail off Bruce Gap Rd., near Route I-75 and the peak of Log Mountain; 36.3072°N, -84.2260°W; elev. 320 m; 3 June 2015; hand collected; M. Kasson leg.; VTEC MPE00544.

####### Other material.

United States – **Tennessee** • 1 ♂; Campbell County, 10 miles NE of LaFollette; 36.4716°N, -83.9848°W; 11 May 1951; L. Hubricht leg.; VMNH NAN0192. For detailed collection data see Suppl. material 7.

####### Diagnosis.

Adult males of *Nannariakassoni* sp. nov. are distinct from other *Nannaria* and the nearby *Nannaria* sp. nov. ‘Cratagae’ (*wilsoni* species group) based on the following combination of characters: ***Gonopods*.** Gonopodal acropodite curving medially before apex, not straight as in *Nannaria* sp. nov. ‘Cratagae.’ Apex with distinct constriction (Fig. 104A, red arrow), tip sharp, with acuminate, triangular lateral flange, not blunt with small lobed lateral and medial flanges as in *N.cryomaia* sp. nov. Acropodite with expanded, laminate, medial flange (Fig. 104A, red triangle). Prefemur with medially curving prefemoral process, paralleling curve of acropodite, not straight, acicular as in *Nannaria* sp. nov. ‘Cratagae.’ Prefemoral process arising dorsolaterally from pronounced curving prefemoral spine, not arising from prefemur with short, triangular prefemoral spine as in *N.cryomaia* sp. nov. Telopodite basal zone height ca. 1/3 length of acropodite, not > 1/3 as in *N.cryomaia* sp. nov., and not ca. 1/4 length as in *Nannaria* sp. nov. ‘Cratagae.’ ***Color*.** Tergites with orange stripes (Fig. 105). Dark brown background. Dorsum of collum smooth with orange margin.

####### Measurements.

♂ holotype (VTEC, MPE00544): BL = 27.0, CW = 3.8, IW = 2.2, ISW = 1.0, B11W = 4.7, B11H = 2.6.

####### Variation.

No known variation.

####### Distribution.

Known from two locations near La Follette, Tennessee (Tennessee: Campbell County; Suppl. material 7, Fig. 126). Distribution area: N/A; status: MRE.

####### Ecology.

Individuals of *N.kassoni* sp. nov. were collected from mesic broadleaved forests, ranging from ca. 300 m elevation adjacent to a floodplain to ca. 750 m elevation near the peak of Log Mountain.

####### Etymology.

This species is named after its collector, Dr. Matt Kasson. The specific name is a genitive noun derived as a patronym.

####### Type locality.

United States, Tennessee, Campbell County, Caryville, along Cove Creek Trail off Bruce Gap Rd., near Route I-75 and the peak of Log Mountain, 36.3072°N, -84.2260°W.

###### Nannaria minor

Taxon classificationAnimaliaPolydesmidaXystodesmidae

Chamberlin, 1918

7512BB3F-62E3-53CD-B861-DECE345F8953

**Figure 106.**
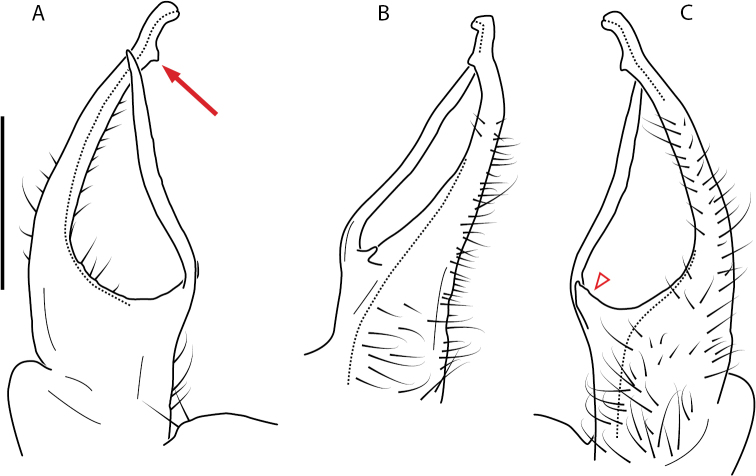
*Nannariaminor* ♂ (VTEC, MPE01249) left gonopod **A** anterior view; red arrow indicates tooth-like medial flange **B** medial view **C** posterior view; red triangle indicates cephalically curved prefemoral spine. Scale bar: 0.5 mm.

**Figure 107.**
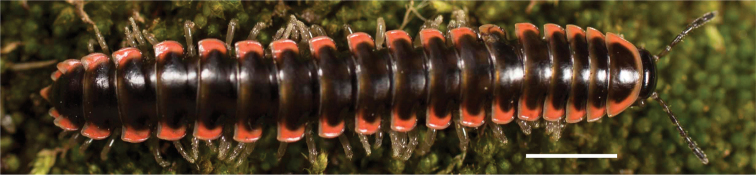
*Nannariaminor* ♂ (VTEC, MPE01249) coloration. Scale bar: 4.0 mm.

Figs 106 , 107 Vernacular name: “The First Twisted-Claw Millipede”

Nannaria minor Chamberlin, 1918: 124. Attems 1938: 199. Brimley 1938: 499. Chamberlin and Hoffman 1958: 41. Hoffman 1964: 11. Wray 1967: 152. Hoffman 1999: 367. Shelley 2000: 196. Marek et al. 2014: 37. Means et al. 2021: 16, S71.

####### Material examined.

United States – **North Carolina** • 1 ♂; Ashe County, West Jefferson, Mount Jefferson State Natural Area, lower loop of Rhododendron Trail; 36.4032°N, -81.4646°W; elev. 1388 m); 10 Oct. 2018; hand collected; D. Hennen leg.; VTEC MPE04388 • 1 ♀; same collection data as preceding; VTEC MPE04389 • 3 ♂♂; Mitchell County, Little Switzerland, Grassy Creek Falls; 35.8561°N, -82.0876°W; elev. 995 m; hand collected; D. Hennen leg.; VTEC MPE04391–93 • 2 ♀♀; same collection data as preceding; VTEC, MPE04394, 4395 • 6 ♂♂; Mitchell County, Crabtree Meadows Rec. Area, B.R.P.; 35.8996°N, -82.2147°W; elev. 1036–1097 m; 30 Oct. 1971; R. Hoffman, Knight leg.; VMNH NAN0200 • 1 ♂; Mitchell County, 1000’ S of Sam’s Gap, on U.S. Hy. 23; 35.9385°N, -82.5632°W; 2 Aug. 1962; R. Hoffman leg.; VMNH NAN0202 • 1 ♂; Mitchell County, Bakersville, around garage at home; 36.0156°N, -82.1587°W; 13 Feb. 1976; D. Terrel leg.; NCSM NAN0554 • 1 ♂; Mitchell County, summit of Roan Mtn., in spruce-fir forest; 36.1039°N, -82.1219°W; elev. 1829 m; 24 July 1975; R. Shelley, J. Clamp leg.; NCSM NAN0502 • 4 ♂♂♀♀; Watauga County, 2.8 WNW Blowing Rock, Julian Price Memorial Park, off Blue Ridge Pkwy.; 36.1422°N, -81.7456°W; 9 Sep. 1973; R. Shelley leg.; NCSM NAN0520; SCAU – **Tennessee** • 1 ♀; Carter County, northeast of Hampton, Watauga Point Rec Area, trail from parking lot; 36.3199°N, -82.0834°W; elev. 632 m; 12 June 2018; hand collected; D. Hennen leg.; VTEC MPE04060 • 1 ♂; Carter County, Hampton, Cherokee National Forest, Laurel Creek Falls Trail; 36.2652°N, -82.1235°W; elev. 768 m; 12 June 2018; hand collected; D. Hennen leg.; VTEC MPE04074 • 3 ♀♀; Carter County, Roan Mountain, Roan Mountain State Park, Blue 2 Trail; 36.1674°N, -82.0984°W; elev. 872 m; 12 June 2018; D. Hennen leg.; VTEC MPE04080–82 • 3 ♂♂; Carter County, The Laurels Rec Area, Cherokee National Forest; 36.2476°N, -82.2696°W; elev. 584 m; 13 June 2018; D. Hennen leg.; VTEC MPE04089, 4090, 4226 • 2 ♀♀; same collection data as preceding; VTEC, MPE04091, 4092 • 1 ♂; Carter County, south of Alizabethton, at pull off on Route 362; 36.2651°N, -82.2300°W; elev. 620 m; 25 May 2016; hand collected; J. Means, D. Hennen leg.; VTEC MPE01249 • 8 ♀♀; same collection data as preceding; VTEC MPE01252, 1253, 1296, 1302, 1303, 1313, 1314, 2061 • 1 ♂; Carter County, Doe R. Bluff, 1 mile NW of Hampton; 36.2944°N, -82.1855°W; 3 May 1951; L. Hubricht leg.; VMNH NAN0293 • 1 ♂; Loudon County, Lenoir City, basement of house; 35.7972°N, -84.2561°W; 10 Dec. 1971; W. Tolbert leg.; VMNH NAN0144 • 1 ♀; Unicoi County, Unicoi, Limestone Cove Rec Area; 36.1742°N, -82.2982°W; elev. 697 m; hand collected; D. Hennen leg.; VTEC MPE04103 • 3 ♀♀; Unicoi County, Erwin, Cherokee National Forest, Rock Creek Falls Rec Area, Rock Creek Falls Trail; 36.1389°N, -82.3468°W; elev. 704 m; 13 June 2018; hand collected; D. Hennen leg.; VTEC MPE04107, 4108, 4231 • 2 ♂♂; Unicoi County, 10.7 air km from Erwin, Cherokee National Forest, beginning of trail to Sill Creek Falls, off Clark Creek Road; 36.1281°N, -82.5340°W; elev. 559; 14 June 2018; hand collected; D. Hennen leg.; VTEC MPE04127, 4128 • 3 ♀♀; Unicoi County, Flag Pond, Rocky Fork State Park, Rocky Fork Trail; 36.0482°N, -82.5615°W; elev. 740 m; hand collected; D. Hennen leg.; VTEC MPE4133–35 • 1 ♂; Unicoi County, 3 SW Erwin, Unaka Springs Rd., 1 mi jct. Chestoa Rd.; 36.0931°N, -82.4396°W; 1 Apr. 2002; A. Gagan leg.; NCSM NAN0476 • 2 ♀♀; Unicoi County, Erwin, Cherokee National Forest, Rock Creek Falls Rec Area, Rock Creek Falls Trail; 36.1389°N, -82.3468°W; 13 June 2018; hand collected; D. Hennen leg.; VTEC MPE04108, 4231 • 1 ♀; Washington County, Johnson City, Buffalo Mountain Park, White Rock Lower Loop; 36.2770°N, -82.3461°W; elev. 717 m; 13 June 2018; D. Hennen leg.; VTEC MPE04100. For detailed collection data see Suppl. material 7.

####### Diagnosis.

Adult males of *Nannariaminor* are distinct from other *Nannaria*, and the nearby *N.aenigma*, based on the following combination of characters: ***Gonopods*.** Gonopodal acropodite gently curving medially throughout with extremely short, blunt distal zone, not with serpentine distal zone as in *N.aenigma*. Acropodite with small, tooth-like medial flange near apex (Fig. 106A, red arrow), not with lobed medial flange as in *N.mcelroyorum* sp. nov. Prefemur with long, thin, acicular prefemoral process slightly curving laterally, not sinuous, cephalically directed as in *N.mcelroyorum* sp. nov. Prefemoral spine small, curving cephalically (Fig. 106B, red triangle), without secondary hump as in *N.mcelroyorum* sp. nov. Telopodite basal zone height < 1/2 length of acropodite, not ca. 1/2 length as in *N.mcelroyorum* sp. nov., or ca. 1/6 length as in *N.aenigma*. ***Color*.** Tergites with hot pink/orange paranotal spots (Fig. 107). Light brown to black background. Dorsum of collum smooth with orange margin.

####### Measurements.

♂ (VTEC, MPE01249): BL = 31.8, CW = 3.9, IW = 2.1, ISW = 0.9, B11W = 4.6, B11H = 3.1; ♀ (VTEC, MPE01253): BL = 31.2, CW = 3.7, IW = 2.1, ISW = 1.0, B11W = 5.1, B11H = 3.5.

####### Variation.

Notable variation found in *N.minor* is the length and general directionality of the prefemoral process; a specimen collected from the summit of Roan Mountain in North Carolina (NAN0502) has a short and somewhat curving prefemoral process, while specimens from Sam’s Gap in Tennessee (NAN0202) have long prefemoral processes that do not cross the acropodite in the anterior view, and the specimen illustrated by Hoffman (1964: 32) has a medially directed prefemoral tip.

####### Distribution.

Known from the Southern Appalachians, primarily along the northern half of the North Carolina and Tennessee border (North Carolina: Ashe, Madison, Mitchell, Watauga, and Yancey counties; Tennessee: Carter, Loudon, and Unicoi counties; Virginia: Carroll County, Suppl. material 7; Fig. 126). Distribution area: 5,671 km^2^; status: SRE.

####### Ecology.

Individuals of *Nannariaminor* have been collected from mesic deciduous forests, often dominated by maple, oak, hemlock, and rhododendron, typically under 1–2 cm soil and/or leaf litter.

####### Etymology.

Chamberlin (1918) gave no etymology for his name, *N.minor*, though it is reasonable to assume that he named both the genus and the species for its small size in relation to other xystodesmids. Why Chamberlin would feel the need to reiterate the small nature of the species is unknown. One possibility is that Chamberlin may have chosen the specific name after the Latin word *minor* for ‘smaller’ to differentiate it from a second species he described in the same paper *N.media* Chamberlin, 1918 (now *Borariastricta*) which was slightly larger than *N.minor*, but not as large as the third species he described, *N.infesta* (now *Howellariainfesta*). Thus, *minor* was the smallest of the three species, *media* (from the Latin *medi*- meaning middle) was the middle-sized species, and *infesta* was the largest. If this hypothesis is true it is a mystery to the authors why Chamberlin broke the pattern with *infesta*, though the fungal infestation of the *infesta* type may have offered up too good of a species epithet to pass up.

####### Type locality.

United States, Tennessee, Burbank.

####### Notes.

In the original publication, Chamberlin (1918, 125) mentions that two specimens were collected by R. Thaxter: a male, which Chamberlin designates as the type, and a female.

###### Nannaria rhysodesmoides

Taxon classificationAnimaliaPolydesmidaXystodesmidae

(Hennen & Shelley, 2015)

B8D91701-E813-590A-B3A2-9E03FEA6CB39

**Figure 108.**
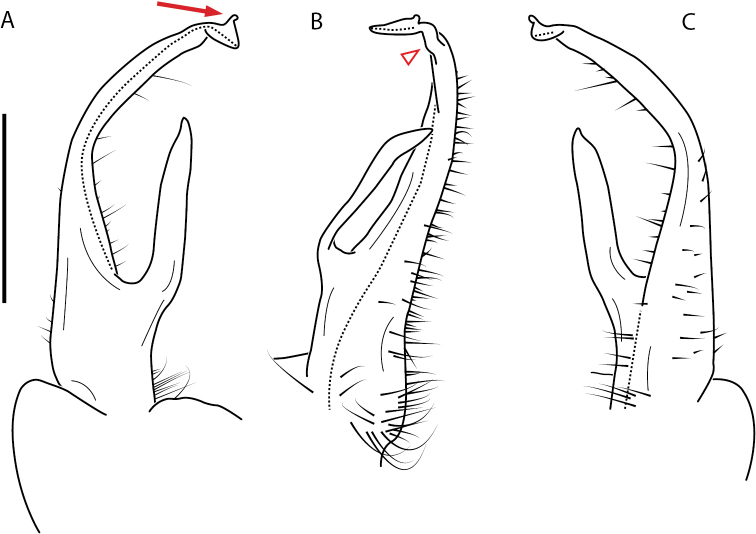
*Nannariarhysodesmoides* holotype ♂ (FSCA) left gonopod **A** anterior view, red arrow indicates small, pointed lateral flange **B** medial view; red triangle indicates slight sinuation of the acropodite apex **C** posterior view. Scale bar: 0.5 mm.

Fig. 108 Vernacular name: “The Rhysodesmus-Like Twisted-Claw Millipede”

Mimuloria rhysodesmoides Hennen & Shelley, 2015: 1–16.
Nannaria rhysodesmoides : Means et al. 2021: S72.

####### Material examined.

***Holotype*:** United States – **Virginia** • ♂; Putnam County, Cookeville; [36.1628°N, -85.5016°W]; 12 Apr. 1958; H.E. Evans leg.; FSCA.

***Paratypes*:** United States – **Virginia** • 6 ♂♂; same collection data as holotype; NCSM 27913. For detailed collection data see Suppl. material 7.

####### Diagnosis.

Adult males of *Nannariarhysodesmoides* are distinct from other *Nannaria* and the nearby *N.blackmountainensis* sp. nov. based on the following combination of characters: ***Gonopods*.** Gonopodal acropodite simple, gently curving dorsomedially, not with pronounced medial swelling as in *N.blackmountainensis* sp. nov. Acropodite tip with small, triangular lateral flange (Fig. 108A, red arrow), not large, hooked lateral flange as in *N.blackmountainensis* sp. nov. Apex slightly sinuate (Fig. 108B, red triangle). Acropodite tip terminating in small, sharp, dorsally directed point, not blunt, squared off as in *N.blackmountainensis* sp. nov. Height of telopodite basal zone < 1/2 length of prefemoral process, not ca. 1/2 length of prefemoral process as in *N.blackmountainensis* sp. nov. Prefemoral process straight, wide, approaching laminate, not thin and acuminate as in *N.blackmountainensis* sp. nov. Prefemoral spine completely reduced not forming small ridge as in *N.blackmountainensis* sp. nov. ***Color*.** The preserved holotype suggests that *N.rhysodesmoides* may have metatergal stripes connecting the paranotal spots, though living specimens have not be observed by the authors.

####### Measurements.

♂ paratype (NCSM27913; ♂ holotype too fragmented for measurement): BL = 22.3, CW = 2.9, IW = 1.8, ISW = 0.8, B11W = 4.4, B11H = 2.4.

####### Variation.

No known variation.

####### Distribution.

Known only from the type locality (Tennessee: Putnam County, Suppl. material 7; Fig. 129). Distribution area: N/A; status: MRE.

####### Ecology.

Hennen and Shelley (2015) do not mention ecological notes in their description of the species, most likely due to a lack of ecological information from the original collector.

####### Etymology.

Hennen and Shelley (2015) chose the specific name based on the presence of a lateral flange on the gonopod (Fig. 108A, red arrow), a character also seen in the genus *Rhysodesmus* Cook, 1895.

####### Type locality.

United States, Virginia, Putnam County, Cookeville.

####### Notes.

In the original publication, Hennen and Shelley (2015: 15) designate a male holotype and five male paratypes (FSCA) collected by H. E. Evans on 12 April 1958, and note that one male paratype from this collection was retained at the NCSM.

##### Species with uncertain clade membership

###### Nannaria conservata

Taxon classificationAnimaliaPolydesmidaXystodesmidae

Chamberlin, 1940

53C97763-8713-5BEC-9283-F0C97ACF76F5

**Figure 109.**
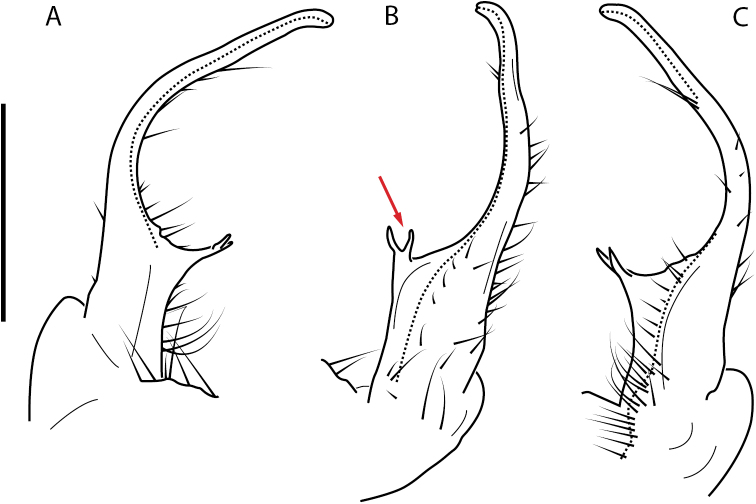
*Nannariaconservata* ♂ (VMNH, NAN0338) left gonopod **A** anterior view **B** medial view; red arrow indicates reduced prefemoral spine paralleling prefemoral process **C** posterior view. Scale bar: 0.5 mm.

Fig. 109 Vernacular name: “The Duke Forest Twisted-Claw Millipede”

Nannaria conservata Chamberlin 1940: 56. Chamberlin and Hoffman 1958: 40. Wray 1967: 152. Shelley 1974: 111. Shelley 1975: 180, figs 1–6. Shelley 1978: 66, figs 67–69. Hoffman 1999: 366. Shelley 2000: 196. Marek et al. 2014: 36. Means et al. 2021: S69.

####### Material examined.

United States – **North Carolina** • 1 ♂; Franklin County, near Moccasin Creek, 1.5 miles SW of Pilot; 35.8625°N, -78.2683°W; 2 May 1959; L. Hubricht leg.; VMNH, NAN0336 • 7 ♂♂♀♀; Person County, 2 mi SSE Surl, off end of SR 1718 in window wells, Sheets residence; 36.3157°N, -78.8887°W; 3 Apr. 1999; A. & S. Braswell, R. Sheets leg.; NCSM NAN0547 • 1 ♀; Wake County, pond near William B Umstead State Park; 35.8366°N, -78.7623°W; elev. 118 m; 17 Apr. 2016; hand collected; J. Means leg.; VTEC MPE02117 • 8 ♂♂; Wake County, Raleigh, 209 Lynwood Lane; 35.8404°N, -78.6376°W; 15 Mar. 1975; M. Cooper leg.; VMNH NAN0337, 338 • 12 ♂♂♀♀; same collection data as preceding; 29 Jan. 1975; M. Cooper leg.; NCSM, NAN0549, 550, 552 • 2 ♂; Wake County, Raleigh, 1611 Oberlin Rd.; 35.8036°N, -78.6582°W; 23 Jan. 1990; D. Stephan leg.; NCSM NAN0546 • 1 ♂; Wake County, Raleigh, jct. Athens Dr. & Avent Ferry Rd.; 35.7674°N, -78.7066°W; 10 Mar. 1990; R. Shelley leg.; NCSM NAN0550. For detailed collection data see Suppl. material 7.

####### Diagnosis.

Adult males of *Nannariaconservata* are distinct from other *Nannaria* and the nearby *N.hardeni* sp. nov. based on the following combination of characters: ***Gonopods*.** Gonopodal acropodite gently curving dorsomedially before apex, not medially as in *N.hardeni* sp. nov.; tip simple, blunt. Telepodite basal zone height < 1/2 length of acropodite, not > 1/2 as in *N.hardeni* sp. nov. Prefemoral process acicular, reduced, not subequal to height of telopodite basal zone and crossing acropodite as in *N.hardeni* sp. nov. Prefemoral spine acicular and paralleling prefemoral process (Fig. 109B, red arrow), not blunt and widely separated from prefemoral process as in *N.hardeni* sp. nov. ***Color*.** In his redescription of *N.conservata*, Shelley (1975) describes its color as “olive-brown with a pink epiproct and paranota…” The single female individual collected for this revision conformed to Shelley’s description, but was placed in 100% EtOH in the field not photographed alive.

####### Measurements.

♂ (VMNH, NAN0388): BL = 21.2, CW = 2.8, IW = 1.6, ISW = 0.8, B11W = 3.2, B11H = 2.3.

####### Variation.

There is no notable variation between individuals of *N.conservata*, with the exception of an abnormal male with three gonopods which Shelley (1975) illustrated and discussed.

####### Distribution.

Known only from a small area around Raleigh and Durham, North Carolina (North Carolina: Person, Orange, Wake and Franklin counties; Suppl. material 7; Fig. 128). Distribution area: 1,820 km^2^; status: SRE.

####### Ecology.

As no fresh identifiable material was collected for this revision, the authors cannot comment on habitat preference. However, Shelley (1975) noted that specimens were never found in moist areas, and instead were discovered under pine litter and in sandy, dry soils. As Shelley (1975) noted, this is unusual for a majority of xystodesmid species.

####### Etymology.

Chamberlin (1940) gives no etymology for *N.conservata*, but the specific name is seemingly a femininization of the Latin *conservo*, meaning conserve. This may be in reference to the conservation efforts which take place within its type locality, Duke Forest.

####### Note on clade membership.

The authors were unsuccessful at collecting specimens of *Nannariaconservata* for the present study, so DNA sequences, molecular phylogenetic relationship, and taxonomic affinities between *N.conservata* and the rest of the *minor* species group remain unknown. A female specimen from Raleigh, Wake County, North Carolina was collected and sequenced, and was placed within the *paupertas* clade. The only species of *Nannaria* known from Wake County is *N.conservata*. However, as *N.conservata* shares none of the gonopodal characters that unite the *paupertas* clade, and in light of the possibility that the female from Wake County represents an undescribed species, the authors refrain from placing *N.conservata* within the *paupertas* clade and leave the taxonomic affinity of *N.conservata* to be addressed in a future study.

####### Type locality.

United States, North Carolina, Durham County, Durham.

####### Notes.

In the original publication, Chamberlin (1940: 57) did not designate a type, but he describes only a male specimen which is therefore the holotype by monotypy. Chamberlin did not mention the collector of this specimen, but Shelley (1975: 180) reported that the type specimen was collected by N. B. Causey on November 12, 1939.

###### Nannaria oblonga

Taxon classificationAnimaliaPolydesmidaXystodesmidae

(Koch, 1847)

07444855-538E-52A6-8D6F-A30699B51E02

**Figure 110.**
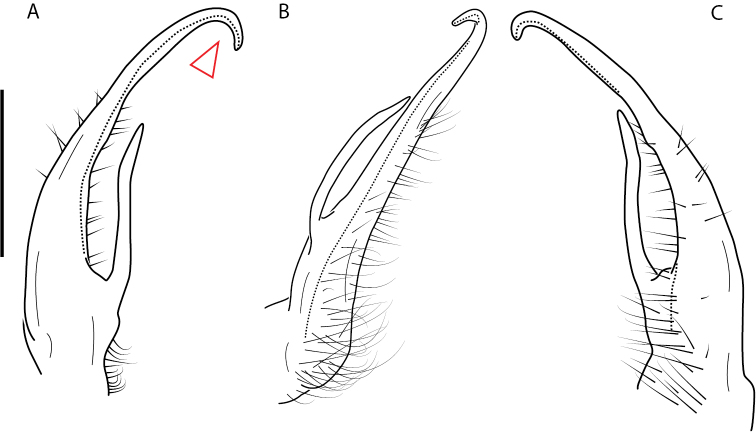
*Nannariaoblonga* syntype ♂ (MFN) left gonopod **A** anterior view; red triangle indicates crochet-like acropodite tip **B** medial view **C** posterior view. Scale bar: 0.5 mm.

Fig. 110 Vernacular Name: “The Pennsylvania Twisted-Claw Millipede”

Fontaria oblonga C.L. Koch, 1847: 142. C.L. Koch 1863: 73–74, fig. 64. Attems 1898: 263. Attems 1938: 167. Chamberlin and Hoffman 1958: 55.
Nannaria oblonga : Hoffman 1999: 367. Marek et al. 2014: 37. Means et al. 2021: S71.

####### Material examined.

**Syntype**: United States – **Pennsylvania** • ♂; from an unknown locality in “Pennsylvania”; MFN.

####### Diagnosis.

Adult males of *Nannariaoblonga* are distinct from other *Nannaria*, the possibly sympatric *N.fowleri*, and the nearby *N.shenandoa*, based on the following combination of characters: ***Gonopods*.** Gonopodal acropodite gently curving medially before apex, not strongly curved as in *N.shenandoa*. Distal zone short, simple, curving smoothly posteriorly, with a crochet-like appearance (Fig. 110A, red triangle). Distal zone not directed medially as in *N.fowleri*, or elongated, with flanges as in *N.shenandoa*. Telopodite basal zone ca. 1/3 length of acropodite, not ca. 1/4 as in *N.shenandoa*. Prefemur with prefemoral process thin, ca. 1/2 length of acropodite, curving medially to parallel acropodite near tip, not elongated, 1/3 length of acropodite and paralleling acropodite majority of length as in *N.fowleri*, or curving laterally as in *N.shenandoa*. Prefemoral spine lacking, not pronounced as tooth-like structure as in *N.fowleri*. ***Color*.**Koch (1847) describes *N.oblonga* as being dark brown with white paranota.

####### Measurements.

The type is too fragmented for accurate measurement.

####### Variation.

No known variation.

####### Distribution.

Known only from the type locality (Pennsylvania, Fig. 127). Distribution area: N/A; status: MRE.

####### Ecology.

Koch (1847) does not mention any ecological notes in his description of the species.

####### Etymology.

Koch (1847) does not give an etymology for the name *oblonga* in his 1863 publication, he goes into more detail about the syntype, and describes the species as “länglich,” meaning elongated or oblong in German. It is therefore reasonable to assume that the name is in reference to the general oblong shape of *Nannaria*.

####### Note on clade membership.

No specimens of *Nannariaoblonga* were collected for this study, and while *N.oblonga* shares some gonopodal characters with *N.tenuis* sp. nov. (such as the caudally-directed acropodite tip and a lack of a prefemoral spine) *N.oblonga* is geographically disjunct from the *ignis* clade, to which *N.tenuis* sp. nov. belongs. Due to a lack of molecular data and a paucity of shared morphological characters, clade membership is uncertain.

####### Type locality.

United States, Pennsylvania; MFN.

####### Notes.

In the original publication, Koch (1847: 142) did not designate a type or mention the number of specimens he examined and later on, Koch (1863) described both a male and female specimen in greater detail. The specimen in the MFN collection was labeled “Holotypus.” Without knowing the number of specimens Koch examined for his original description of the species, we cannot be sure if it is a holotype by monotypy, and hence refrain from designating a lectotype.

###### Nannaria rutherfordensis

Taxon classificationAnimaliaPolydesmidaXystodesmidae

Shelley, 1975

6B8F3025-DBF8-53D7-BC0F-FACADEAE17C9

**Figure 111.**
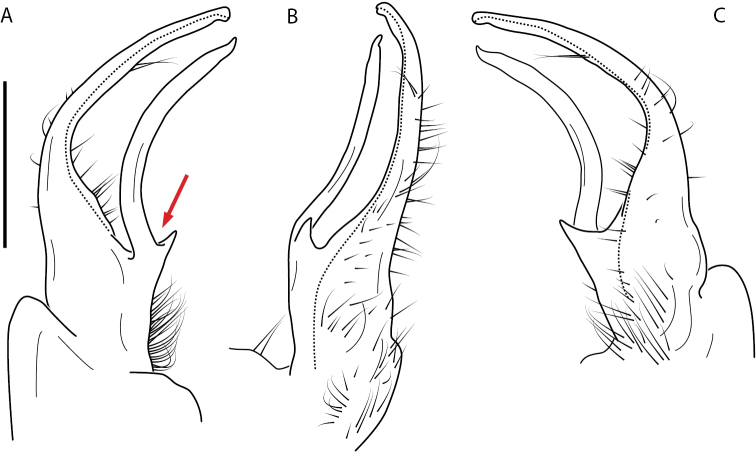
*Nannariarutherfordensis* holotype ♂ (NMNH, USNMENT 01491821) left gonopod **A** anterior view, red arrow indicates pronounced, sharp prefemoral spine **B** medial view **C** posterior view. Scale bar: 0.5 mm.

Fig. 111 Vernacular name: “The Rutherfordton Twisted-Claw Millipede”

Nannaria rutherfordensis Shelley, 1975: 184, figs 7–9. Hoffman 1999: 367. Shelley 2000, 197. Marek et al. 2014: 38. Means et al. 2021: S72.

####### Material examined.

***Holotype*:** United States – **North Carolina** • ♂; Rutherford County, Rutherfordton, Bus. 74, 0.2 W Byp. 74; [35.3108°N, -81.9303°W]; 15 Oct. 1973; R. M. Shelley leg.; NMNH USNMENT 01491821. For detailed collection data see Suppl. material 7.

####### Diagnosis.

Adult males of *Nannariarutherfordensis* are distinct from other *Nannaria* and the nearby *N.minor* based on the following combination of characters: ***Gonopods*.** Gonopodal acropodite simple, gently curving medially with extremely short, blunt distal zone, not with small, tooth-like medial flange near apex as in *N.minor*. Prefemur with long, thin, medially curving prefemoral process, not acicular slightly curving laterally as in *N.minor*. Prefemoral process arising from sharp, projected prefemoral spine (Fig. 111A, red arrow), not from small prefemoral spine which curves cephalically as in *N.minor*. Telopodite basal zone height < 1/2 length of acropodite. ***Color*.**Shelley (1975) described *N.rutherfordensis* as having white paranota with a concolorous stripe along the anterior edge of the collum.

####### Measurements.

♂ holotype (NMNH, USNMENT 01491821): BL = 28.0, CW = 3.2, IW = 2.2, ISW = 0.9, B11W = 4.3, B11H = 2.8.

####### Variation.

No known variation.

####### Distribution.

Known only from the type locality (North Carolina: Rutherford County, Suppl. material 7, Fig. 126). Distribution area: N/A; status: MRE.

####### Ecology.

Shelley (1975, pp. 186) notes that the holotype was found under leaves by the side of a log in an “urban patch of hardwoods, a few feet from the pavement...” The authors JCM and DAH were unable to recollect *N.rutherfordensis* on multiple trips to the type locality and surrounding area. The type locality had unfortunately recently been cleared for development and was found dry and lacking vegetation on each visit. The authors infer this species may be locally extirpated.

####### Etymology.

Shelley (1975) provides no etymology for his naming in his description of the species; however, it is reasonable to assume that *N.rutherfordensis* is named after its type locality.

####### Note on clade membership.

No specimens of *Nannariarutherfordensis* were collected for this study. While it shares the gonopodal character of a prefemoral process that parallels the curved acropodite with *N.kassoni* sp. nov. (*minor* clade), this character is also found in *N.fowleri* (*fowleri* clade) and *N.tasskelsoae* sp. nov. (*tasskelsoae* clade), which are 352 km and 266 km north, respectively. In combination with the relative geographic isolation of the species, being the most southern species in North Carolina, clade membership is uncertain.

####### Type locality.

United States, North Carolina, Rutherford County, Rutherfordton, Bus. 74, 0.2 W Byp. 74; [35.3108°N, -81.9303°W].

####### Notes.

In the original publication, Shelley (1975: 184) designates a male holotype (NCSM 2053) which he collected on October 15, 1973. Interestingly, despite the type being deposited by Shelley at the NCSM, at some point before the writing of Hoffman’s (1999) checklist the specimen was moved to the NMNH and relabeled with the code USNMENT 01491821 by an unknown party.

###### Nannaria sigmoidea

Taxon classificationAnimaliaPolydesmidaXystodesmidae

(Hennen & Shelley, 2015)

BBAFCC7B-1C0D-525B-B168-0C002B8A6BBF

**Figure 112.**
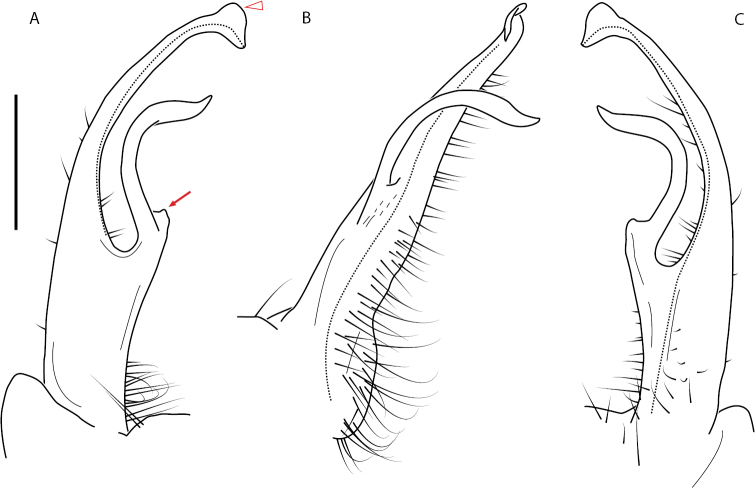
*Nannariasigmoidea* holotype ♂ (NCSM NCSM27946) left gonopod **A** anterior view; red arrow indicates lateral flange; red arrow indicates reduced prefemoral spine **B** medial view **C** posterior view. Scale bar: 0.5 mm.

Vernacular name: “The Sigmoid Gonopod Twisted-Claw Millipede” Fig. 112

Mimuloria dilatata sigmoidea Hennen & Shelley, 2015: 1–15, figs 20, 21.
Nannaria sigmoidea : Means et al. 2021: S72.

####### Material examined.

***Holotype*:** United States – **Tennessee** • ♂; Meigs County, c.a. 7.2 km S Decatur, along country road 4274, 0.8 km from junction of Tennessee highway 58; [35.4378°N, -84.9140°W]; 14 Oct. 1978; R. M. Shelley, W. B. Jones leg.; NCSM NCSM27946. For detailed collection data see Suppl. material 7.

####### Diagnosis.

Adult males of *N.sigmoidea* are distinct from other *Nannaria*, including the nearby *N.blackmountainensis* sp. nov. and *Nannaria* sp. nov. ‘Cratagae’ (*wilsoni* species group), based on the following combination of characters: ***Gonopods*.** Gonopodal acropodite simple and gently curving anteromedially, not straight as in *Nannaria* sp. nov. ‘Cratagae’ or with medial swelling as in *N.blackmountainensis* sp. nov. Acropodite with prominent, rounded lateral flange near tip (Fig. 112A, red arrow), not hooked as in *N.blackmountainensis* sp. nov. or lacking as in *Nannaria* sp. nov. ‘Cratagae.’ Acropodite tip directed posteriorly, not dorsally as in *N.blackmountainensis* sp. nov. Telopodite basal zone subequal to length of prefemoral process, not ca. 1/2 length as in *N.blackmountainensis* sp. nov. or ca. 1/3 length as in *Nannaria* sp. nov. ‘Cratagae.’ Prefemur with prefemoral process bent at 90° angle at halfway point, directed medioventrally, not straight, acicular as in *N.blackmountainensis* sp. nov. and *Nannaria* sp. nov. ‘Cratagae.’ Prefemoral spine reduced to small, blunt lobe at base of prefemoral process (Fig. 112A, red triangle), not fused as a small ridge as in *N.blackmountainensis* sp. nov. or lacking as in *Nannaria* sp. nov. ‘Cratagae.’ ***Color*.** Color in life unknown.

####### Measurements.

♂ holotype (NCSM, NCSM27946): BL = 36.1, CW = 5.0, IW = 3.0, ISW = 1.1, B11W = 6.0, B11H = 3.8.

####### Variation.

No known variation.

####### Distribution.

Known only from the type locality (Tennessee: Meigs County; Suppl. material 7; Fig. 129). Distribution area: N/A; status: MRE.

####### Ecology.

Hennen and Shelley (2015) note that only that the type specimens were collected from the east side of the Tennessee River.

####### Etymology.

Hennen and Shelley (2015: 15) stated that “[t]he subspecific name denotes the overall sigmoidal curvature of the prefemoral process, which also passes through numerous vertical planes.”

####### Note on clade membership.

No specimens of *Nannariasigmoidea* were collected for this study. *Nannariasigmoidea* shares gonopodal characters with members of the *ambulatrix* clade, such as a prominent lateral flange on the acropodite tip, a prefemoral process bent at 90°, and crossing of the acropodites in the medial view (found in *N.ambulatrix* sp. nov. and *N.botrydium* sp. nov.). However, due to the wide geographic separation between *N.sigmoidea* and the *ambulatrix* clade (> 270 km), and the presence of these characters in a member of the *minor* clade (*N.kassoni* sp. nov.), the phylogenetic placement of *N.sigmoidea* in a clade is uncertain.

####### Type locality.

United States, Tennessee, Meigs County, c.a. 7.2 km S Decatur, along country road 4274, 0.8 km from junction of Tennessee highway 58.

####### Notes.

In the original publication, Hennen and Shelley (2015: 15) designate a male holotype collected by R. M. Shelley and W. B. Jones on October 14, 1978.

**Figure 113.**
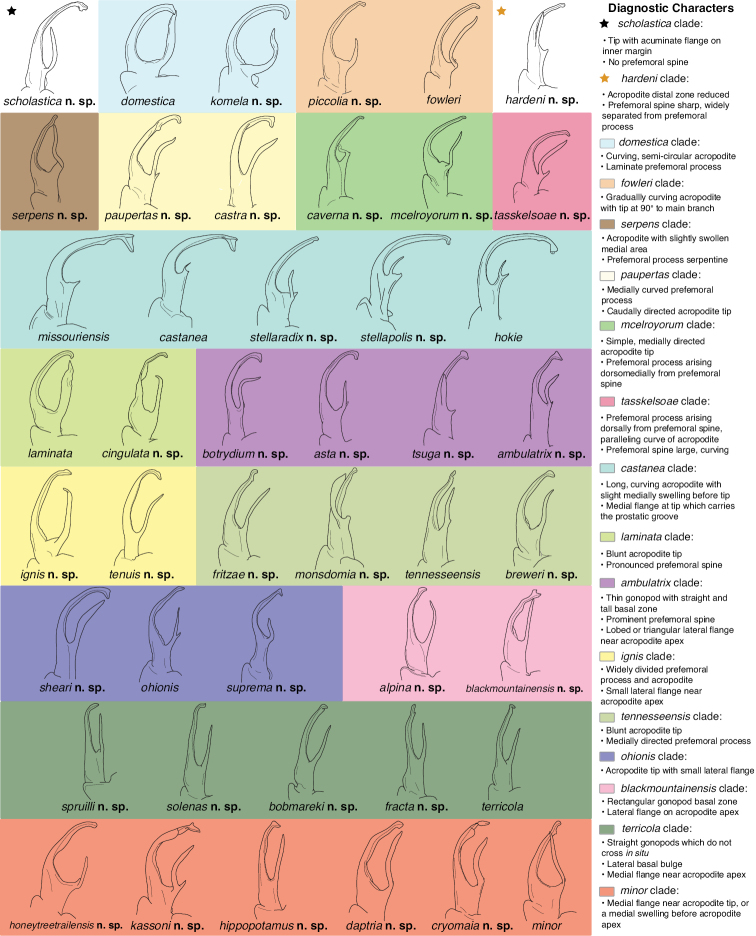
The 17 *minor* species group clades represented by males. Black star: *Nannariascholastica*, sky blue: *N.domestica* clade, orange: *N.fowleri* clade, burnt orange star: *N.hardeni*, brown: *N.serpens* clade, light yellow: *N.paupertas* clade, green: *N.mcelroyorum* clade, cranberry pink: *N.tasskelsoae* clade, turquoise: *N.castanea* clade, lime green: *N.laminata* clade, purple: *N.ambulatrix* clade, yellow: *N.ignis* clade, camo green: *N.tennesseensis* clade, blue: *N.ohionis* clade, light pink: *N.blackmountainensis* clade, forest green: *N.terricola* clade, red: *N.minor* clade.

**Figure 114.**
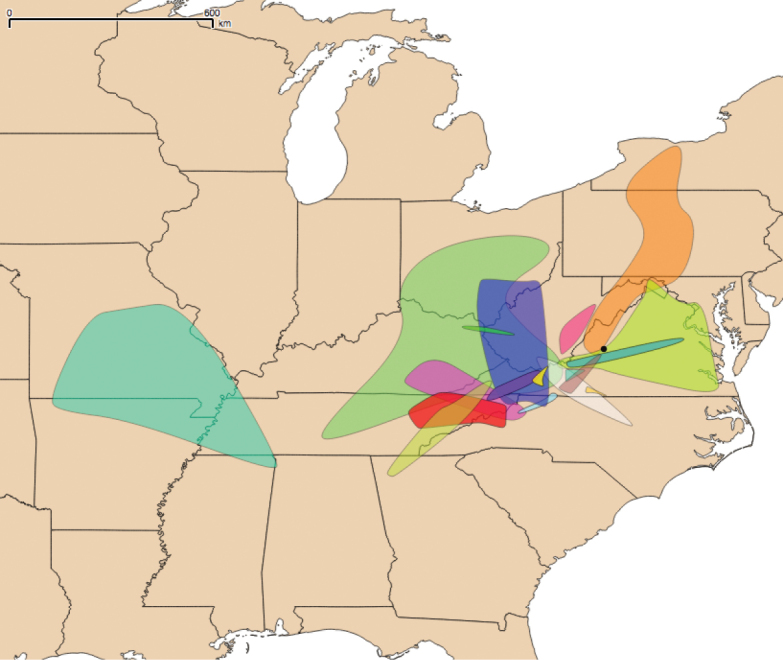
Geographic distributions of the 18 *minor* group clades. Black dot: *Nannariascholastica*, sky blue: *N.domestica* clade, orange: *N.fowleri* clade, white: *N.paupertas* clade, dark green: *Nannaria* ‘Murphy’ clade, green: *N.mcelroyorum* clade, burnt orange: *N.hardeni*, brown: *N.serpens* clade, turquoise: *N.castanea* clade, neon green: *N.laminata* clade, cranberry pink: *N.tasskelsoae* clade, camo green: *N.tennesseensis* clade, yellow: *N.ignis* clade, blue: *N.ohionis* clade, pink: *N.blackmountainensis* clade, light green: *N.terricola* clade, purple: *N.ambulatrix* clade, red: *N.minor* clade.

##### *Nannariaminor* species group *incertae sedis*

The following taxa are of uncertain taxonomic delimitation and placement within the *Nannariaminor* species group. These hypothesized species are morphologically distinct but do not meet our criteria for species delimitation, and so are not formally described. For example, these specimens represent historical natural history collection material and lack intact genetic material. These groupings, therefore, do not define novel species, but provide putative species and a basis for future investigations of the species diversity of *Nannaria*.

###### Nannaria

Taxon classificationAnimaliaPolydesmidaXystodesmidae

“Attica” incertae sedis

BCFF5CF2-1729-5CFD-83E0-535029DEB623

Fig. 115

####### Material examined.

United States – **West Virginia** • 1 ♂; Mercer County, Athens; 37.4222°N, -81.0163°W; 27 Mar. 1966; W. Shear leg.; VMNH NAN0051 • 1 ♂; Summers County, Lick Creek; 37.4845°N, -80.9114°W; 15 May 1956; W. Shear leg.; VMNH, NAN0052. For detailed collection data see Suppl. material 7.

####### Hypothesized placement.

**Figure 115.**
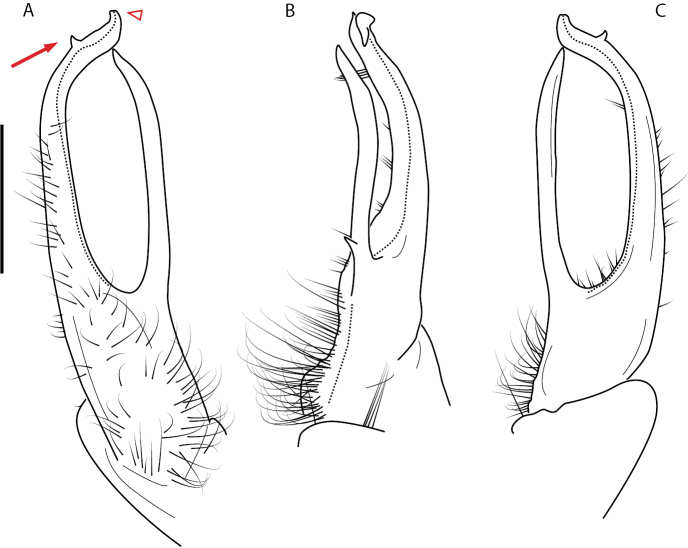
*Nannaria* “Attica” *incertae sedis* ♂ (VMNH, NAN0051) right gonopod, mirrored for consistency **A** anterior view, red arrow indicates small, triangular lateral flange; red triangle indicates cephalically directed acropodite tip **B** medial view **C** posterior view. Scale bar: 0.5 mm.

*Nannaria* “Attica” specimens are hypothesized to be closely related to *N.hippopotamus* sp. nov. based on the following combination of gonopodal characters: Acropodite straight with sudden bend at tip. Acropodite with lateral flange (Fig. 115A, red arrow). *Nannaria* “Attica” specimens differ from *N.hippopotamus* sp. nov. based on the following combination of gonopodal characters: Acropodite lateral flange small and triangular (Fig. 115A, red arrow), not with prominent lobed lateral flange as in *N.hippopotamus* sp. nov. Acropodite tip curving cephalically (Fig. 115A, red triangle). Acropodite lacking medial swelling as found in *N.hippopotamus* sp. nov. (Fig. 100A, red triangle). Prefemoral process curving laterally.

###### Nannaria

Taxon classificationAnimaliaPolydesmidaXystodesmidae

“Bina” incertae sedis

763017F9-B97C-5C3D-9BA3-9A20D4491203

Fig. 116

####### Material examined.

United States – **North Carolina** • 3 ♂♂; Ashe County, 3 miles NW of Lansing; 36.5299°N, -81.5493°W; 13 Oct. 1963; R. Hoffman leg.; VMNH NAN0133 • 7 ♂♂; Ashe County, 2 mi. E of Grayson; 36.5361°N, -81.6477°W; 17 May 1974; R. Hoffman leg.; VMNH NAN0162 • 1 ♂; Ashe County, Bina; 36.4836°N, -81.4991°W; 17 May 1974; R. Hoffman leg.; VMNH NAN0163 • SCAU – **Tennessee** • 1 ♂; Johnson County, Backbone Rock Rec. Area 4 mi. S of Damascus; 36.5938°N, -81.8140°W; 2 June 1974; R. Hoffman, L. Knight leg.; VMNH NAN0140 • 14 ♂♂; same collection data as preceding; 4 Sep. 1969; R. Hoffman leg.; VMNH NAN0161. For detailed collection data see Suppl. material 7.

####### Hypothesized placement.

**Figure 116.**
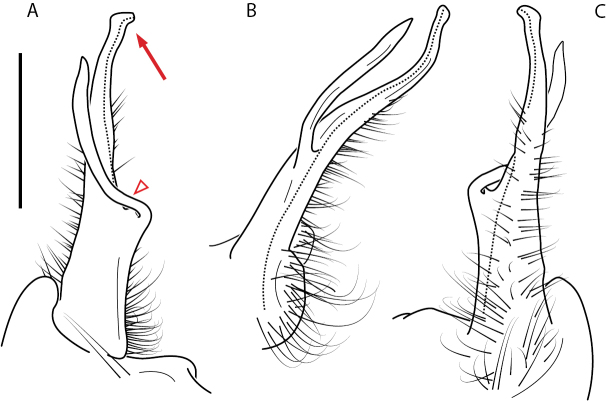
*Nannaria* “Bina” *incertae sedis* ♂ (VMNH, NAN0163) left gonopod **A** anterior view, red arrow indicates reduced distal region; red triangle indicates laterally curving prefemoral process **B** medial view **C** posterior view. Scale bar: 0.5 mm.

*Nannaria* “Bina” specimens are hypothesized to be closely related to *N.monsdomia* sp. nov. based on the following combination of gonopodal characters: Acropodite straight with extremely short distal region (Fig. 116A, red arrow). Prefemoral process curving laterally at base, crossing acropodite dorsally (Fig. 116A, red triangle). *Nannaria* “Bina” specimens differ from *N.monsdomia* sp. nov. based on the following combination of gonopodal characters: Prefemoral spine reduced to toothlike protrusion, not large, lobed as in *N.monsdomia* sp. nov. Prefemoral process thin, not enlarged near tip as in *N.monsdomia* sp. nov.

###### Nannaria

Taxon classificationAnimaliaPolydesmidaXystodesmidae

“Claytor Lake” incertae sedis

830F4582-291E-5449-B7B6-5AFDA97424B3

Fig. 117

####### Material examined.

United States – **Virginia** • 1 ♂; Pulaski County, Claytor Lake St. Pk.; 37.0559°N, -80.6274°W; 10 May 1963; D. Marvin leg.; VMNH NAN0233 • 1 ♂; Pulaski County, 2 mi SW of Snowville; 37.0119°N, -80.5870°W; June; R. Hoffman leg.; VMNH NAN0236 • 13 ♂♂; Pulaski County, RAAP Dublin facility, woodlot near bldg. 1717; 37.1970°N, -80.5459°W; 1 May 1998; S. Garriock leg.; VMNH NAN0239 • 1 ♂; Pulaski County, RAAP – Dublin, 100 m NE of bldg. 1019; 37.1970°N, -80.5459°W; 1 July 1997; S. Garriock leg.; VMNH NAN0240 • 53 ♂♂♀♀; Pulaski County, Radford Army Ammunition Plant, Dublin Facility DF 6 “Sally Pond”; 37.1056°N, -80.6855°W; 14 Apr. 1998; S. Garriock leg.; VMNH NAN0340. For detailed collection data see Suppl. material 7.

####### Hypothesized placement.

**Figure 117.**
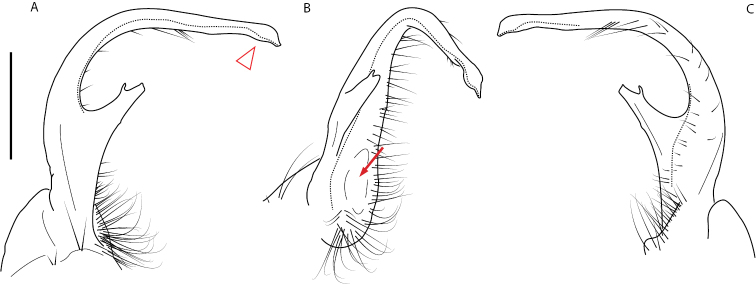
*Nannaria* “Claytor Lake” *incertae sedis* ♂ (VMNH, NAN0233) left gonopod **A** anterior view; red triangle indicates medially directed tip **B** medial view, red arrow indicates basal depression, setae surrounding depression removed for clarity **C** posterior view. Scale bar: 0.5 mm.

*Nannaria* “Claytor Lake” specimens are hypothesized to be closely related to *N.hokie* and belong to the *castanea* clade based on the following combination of gonopod characters: Acropodite distal zone elongated and presence of a small medial flange near the acropodite tip. *Nannaria* “Claytor Lake” can be separated from other *castanea* clade species based on the following combination of gonopodal characters: Acropodite tip robust and directed medially (Fig. 117A, red triangle), not directed caudally as in other members of the *castanea* clade. Telopodite basal zone with distinct concave depression (Fig. 117B, red arrow), not found in other members of the *castanea* clade.

###### Nannaria

Taxon classificationAnimaliaPolydesmidaXystodesmidae

“Hanging Rock” incertae sedis

4904BD24-4F18-5008-A2DB-CFB047DF8648

Fig. 118

####### Material examined.

United States – **Virginia** • 1 ♂; Scott County, woods off Rt. 72, ca 3 mi NE of Dungannon, 500 ft. beyond entrance to Hanging Rock USFS Rec Area; 36.8589°N, -82.4340°W; 14 Nov. 2007; R. Hoffman leg.; VMNH NAN0360. For detailed collection data see Suppl. material 7.

####### Hypothesized placement.

**Figure 118.**
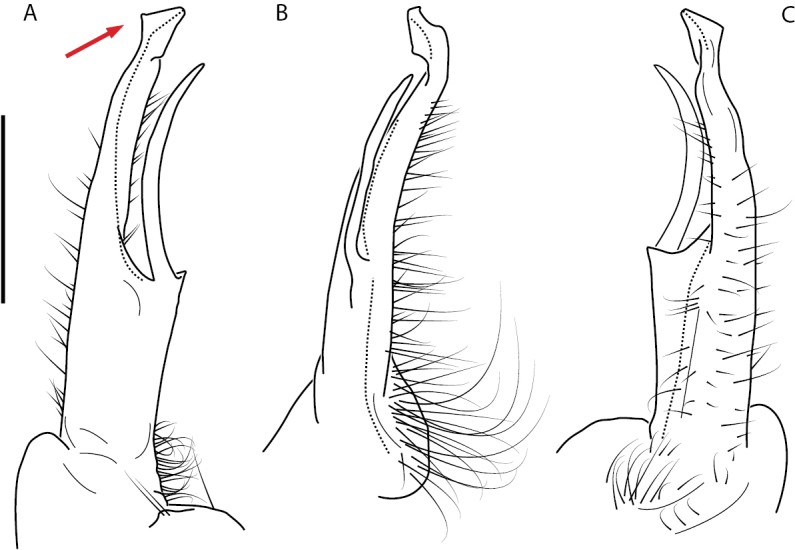
*Nannaria* “Hanging Rock” *incertae sedis* ♂ (VMNH, NAN0360) left gonopod **A** anterior view; red arrow indicates pointed lateral flange **B** medial view **C** posterior view. Scale bar: 0.5 mm.

*Nannaria* “Hanging Rock” specimens are hypothesized to be closely related to *Nannaria* “Bina” based on the following combination of gonopod characters: With predominantly straight acropodite and short distal zone. Prefemoral process arising medially from pronounced, tooth-like prefemoral spine. *Nannaria* “Hanging Rock” can be separated from “Bina” based on the following combination of characters: Acropodite tip pointed, not rounded as in “Bina”. Acropodite with pointed lateral flange near apex (Fig. 118A, red arrow), not rounded as in “Bina”. Prefemoral process curving towards but not crossing acropodite in anterior view.

###### Nannaria

Taxon classificationAnimaliaPolydesmidaXystodesmidae

“Happy Valley” incertae sedis

CFB68CE3-049A-57AC-9184-30DFEB28C37B

Fig. 119

####### Material examined.

United States – **North Carolina** • 1 ♂ and 2 ♀♀; Caldwell County, 5 NW Lenoir, nr. Jct. US321 & NC 268, on paved drive nr. woods; 35.9819°N, -81.5641°W; 19 Nov. 1979; E. Newton leg.; NCSM NAN0499. For detailed collection data see Suppl. material 7.

####### Hypothesized placement.

**Figure 119.**
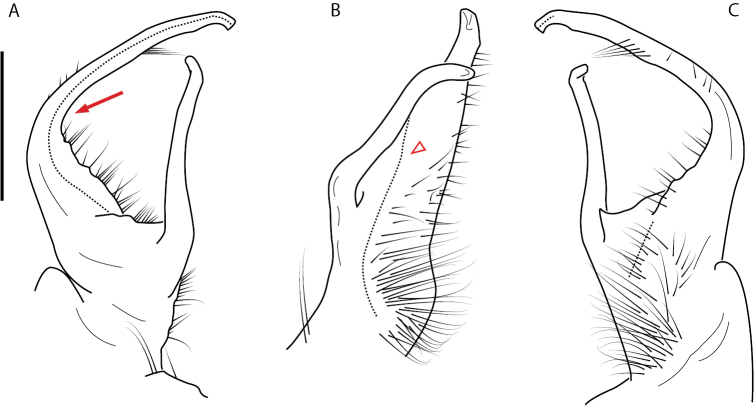
*Nannaria* “Happy Valley” *incertae sedis* ♂ (NCSM, NAN0499) left gonopod **A** anterior view; red arrow indicates abrupt medial bend of acropodite **B** medial view; red triangle indicates wide basal zone **C** posterior view. Scale bar: 0.5 mm.

*Nannaria* “Happy Valley” specimens are hypothesized to be closely related to *Nannariaminor* based on geographic proximity and the following combination of gonopodal characters: With wide distance between bases of acropodite and prefemoral process. Presence of medial flange near apex. Short, blunt distal zone. *Nannaria* “Happy Valley” specimens differ from *N.minor* based on the following combination of gonopodal characters: Acropodite medial flange much reduced in “Happy Valley”. Prefemoral process directed ventrally at tip, not anteriorly as in *N.minor*. Medial curve of acropodite abrupt, forming a nearly 90° angle (Fig. 119A, red arrow), not smooth and gradual as in *N.minor*. Acropodite basal zone extremely wide in medial view (Fig. 119B, red triangle).

###### Nannaria

Taxon classificationAnimaliaPolydesmidaXystodesmidae

“Moores Springs” incertae sedis

1549AAE6-0362-5943-80BF-A4819C22CED4

Fig. 120

####### Material examined.

United States – **North Carolina** • 1 ♂; Stokes County, 0.2 NNW Moores Springs, Moores Springs Cpgd.; 36.4211°N, -80.2891°W; 4 Apr. 2002; J. Beane et al. leg.; VMNH NAN0201 • 1 ♂; Stokes County, 2.5 miles S of Danbury; 36.3730°N, -80.1893°W; 1 Oct. 1950; L. Hubricht leg.; VMNH NAN0203. For detailed collection data see Suppl. material 7.

####### Hypothesized placement.

**Figure 120.**
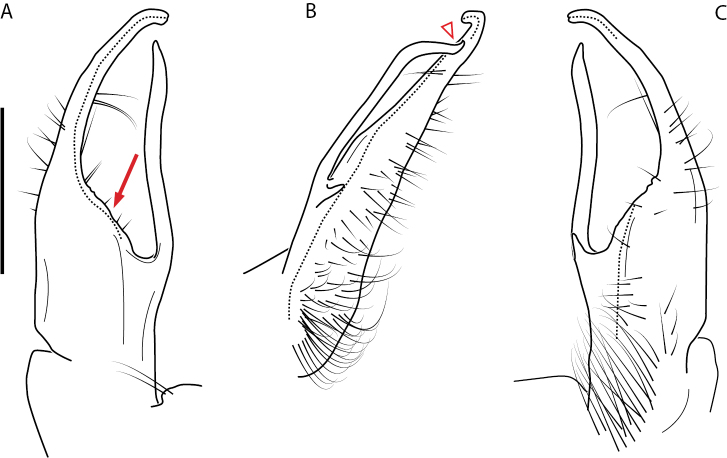
*Nannaria* “Moores Springs” *incertae sedis* ♂ (VMNH, NAN0201) left gonopod **A** anterior view; red arrow indicates raised basal ridge **B** medial view; red triangle indicates ventrally directed prefemoral process tip **C** posterior view. Scale bar: 0.5 mm.

*Nannaria* “Moores Springs” specimens are hypothesized to be closely related to *Nannariaminor* based on the following combination of gonopodal characters: Acropodite tip simple, blunt, directed medially with short distal zone. Prefemoral process thin, approaching length of acropodite. Wide basal zone when viewed anteriorly. *Nannaria* “Moores Springs” specimens differ from *N.minor* based on the following gonopodal characters: Telopodite basal zone with raised ridge (Fig. 120A, red arrow), lacking in *N.minor*. Acropodite simple, lacking medial flange found in *N.minor*. Prefemoral process tip directed ventrally (Fig. 120B, red triangle), not directed laterally as in *N.minor*.

###### Nannaria

Taxon classificationAnimaliaPolydesmidaXystodesmidae

“Natchez Trace” incertae sedis

11427A53-F4F1-56C1-985C-AEAD012324C7

Fig. 121

####### Material examined.

United States – **Tennessee** • 3 ♂♂ and 4 ♀♀; Henderson County, Natchez Trace St. Pk., Fern Nature Trail; 35.7966°N, -88.2646°W; 26 May 1980; R. Shelley leg.; NCSM NAN0540. For detailed collection data see Suppl. material 7.

####### Hypothesized placement.

**Figure 121.**
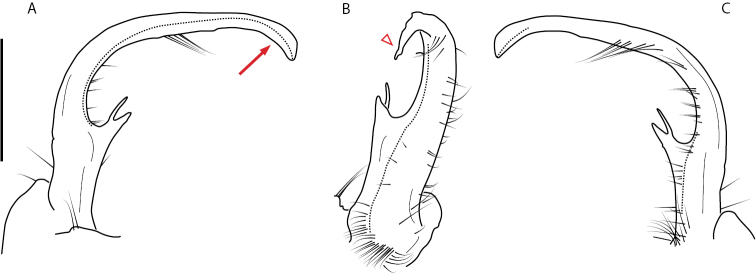
*Nannaria* “Natchez Trace” *incertae sedis* ♂ (NCSM, NAN0540) left gonopod **A** anterior view; red arrow indicates smoothly curving prostatic groove **B** medial view, slightly rotated to show laterally compressed acropodite tip, red triangle **C** posterior view. Scale bar: 0.5 mm.

*Nannaria* “Natchez Trace” specimens are hypothesized to be closely related to *Nannaria* “Claytor Lake” based on the following combination of gonopodal characters: Acropodite robust, curving medially with extended distal zone. Acropodite tip simple, laterally compressed (Fig. 121B, red triangle), directed posteromedially. *Nannaria* “Natchez Trace” specimens differ from *Nannaria* “Claytor Lake” based on the following combination of gonopodal characters: Prefemoral process pronounced, acicular, not reduced as in “Claytor Lake.” Seminal groove continually curving towards tip, without small undulation as in “Claytor Lake” (Fig. 121A, red arrow).

###### Nannaria

Taxon classificationAnimaliaPolydesmidaXystodesmidae

“Peden” incertae sedis

0F4B7B94-117B-5D12-96F4-0061D5AEA467

Fig. 122

####### Material examined.

United States – **North Carolina** • 1 ♂; Alleghany County, 10 mi W Spart, 0.1 mi NE on 1308 jct 1303; 36.4987°N, -81.3080°W; 17 May 1980; R. Shelley, MSM leg.; NCSM NAN0496. For detailed collection data see Suppl. material 7.

####### Hypothesized placement.

**Figure 122.**
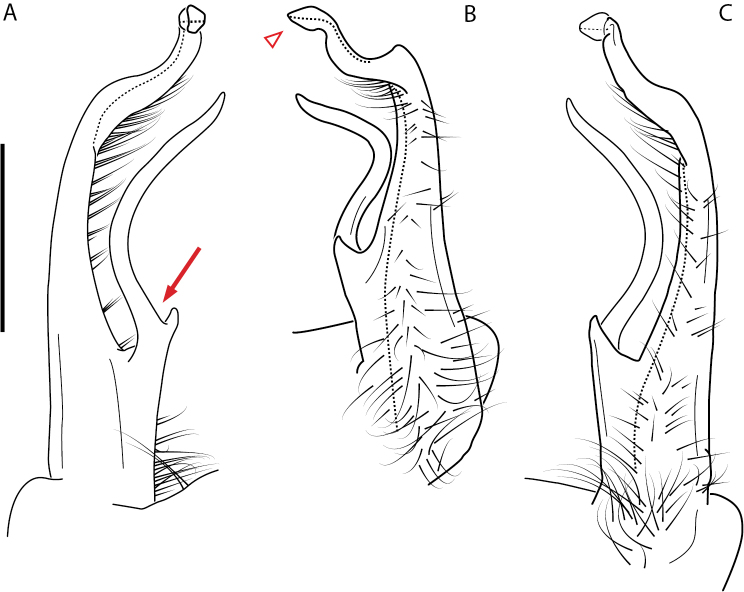
*Nannaria* “Peden” *incertae sedis* ♂ (NCSM, NAN0496) left gonopod **A** anterior view; red arrow indicates prefemoral process arising medially from pronounced prefemoral spine **B** medial view; red triangle indicates dorsally directed acropodite tip **C** posterior view. Scale bar: 0.5 mm.

*Nannaria* “Peden” specimens are hypothesized to be closely related to *Nannariatasskelsoae* sp. nov. based on the following combination of gonopodal characters: Telopodite prefemoral process arising medially from pronounced, tooth-like prefemoral spine (Fig. 122A, red arrow). Prefemoral process paralleling acropodite throughout majority of length. *Nannaria* “Peden” specimens differ from *N.tasskelsoae* sp. nov. based on the following combination of gonopodal characters: Acropodite tip serpentine, resembling snake prepared to strike, directed dorsally (Fig. 122B, red triangle), not directed medially as in *N.tasskelsoae* sp. nov.

###### Nannaria

Taxon classificationAnimaliaPolydesmidaXystodesmidae

“Repass” incertae sedis

C2AEDC53-29C5-5598-BB5A-459EF7DB2988

Fig. 123

####### Material examined.

United States – **Virginia** • 1 ♂; Russell County, Clinch Mtn., 1 mile SE of Repass; 36.9786°N, -81.7926°W; elev. 1128 m; 5 July 1962; R. Hoffman leg.; VMNH NAN0130 • 1 ♂; Russell County, along Laurel Bed Lake, SW of Saltville, Clinch Mountain; 36.9558°N, -81.8137°W; 26 Apr. 1975; D. Ogle leg.; VMNH NAN0143. For detailed collection data see Suppl. material 7.

####### Hypothesized placement.

**Figure 123.**
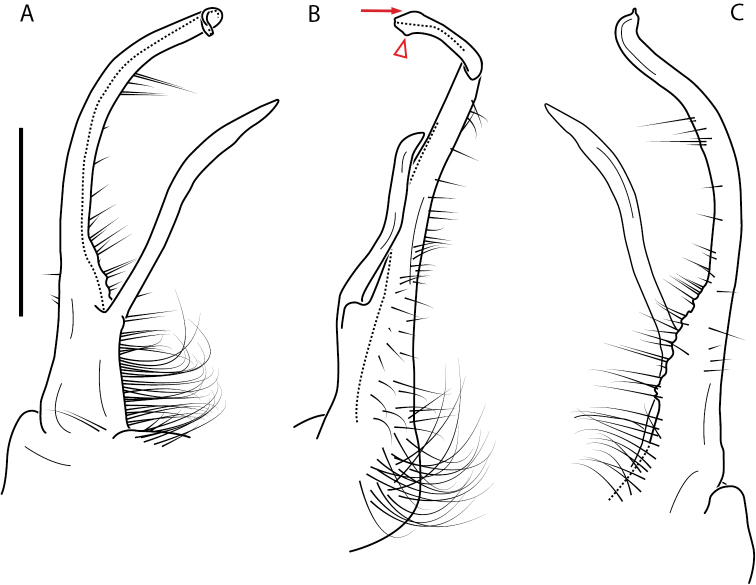
*Nannaria* “Repass” *incertae sedis* ♂ (VMNH, NAN0130) left gonopod **A** anterior view **B** medial view; red arrow and red triangle indicate lateral and medial acropodite tip flanges, respectively **C** posterior view. Scale bar: 0.5 mm.

*Nannaria* “Repass” specimens are hypothesized to be closely related to *Nannariabotrydium* sp. nov. based on close geographic proximity and the following combination of gonopodal characters: Acropodite thin, gently curving medially. Acropodite tip with small lateral flange (Fig. 123B, red arrow). Prefemoral process bending medially at midpoint. *Nannaria* “Repass” differs from *N.botrydium* sp. nov. based on the following gonopodal characters: Acropodite tip bending dorsally with small, triangular medial flange (Fig. 123B, red triangle), not bending caudally as in *N.botrydium* sp. nov. Prefemoral process bending medially at midpoint to a lesser degree than as in *N.botrydium* sp. nov.

###### Nannaria

Taxon classificationAnimaliaPolydesmidaXystodesmidae

“Ridgeway” incertae sedis

7B2A2C3C-F19A-5C48-9C7D-55A7821FD0FC

Fig. 124

####### Material examined.

United States – **Virginia** • 1 ♂; Henry County, Ridgeway; 36.5766°N, -79.8586°W; 27 Nov. 1961; R. Hoffman leg.; VMNH NAN0269. For detailed collection data see Suppl. material 7.

####### Hypothesized placement.

**Figure 124.**
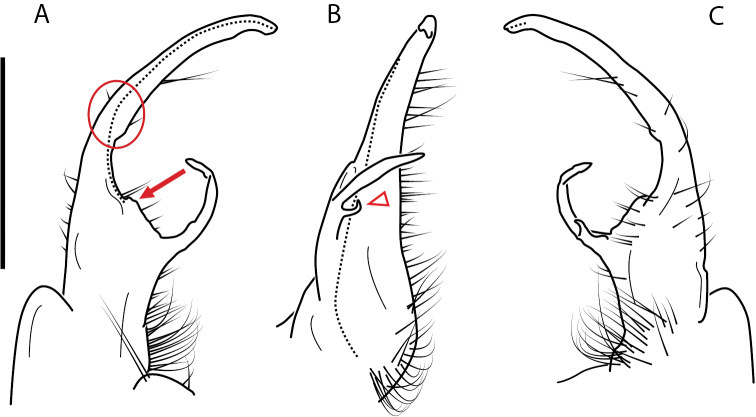
*Nannaria* “Ridgeway” *incertae sedis* ♂ (VMNH, NAN0269) left gonopod **A** anterior view, red arrow indicates raised basal ridge, red oval indicates acropodite medial swelling **B** medial view; red triangle indicates hook-liked prefemoral spine **C** posterior view. Scale bar: 0.5 mm.

*Nannaria* “Ridgeway” specimens are hypothesized to be closely related to *Nannaria* “Moores Springs” based on close geographic proximity and the following combination of gonopodal characters. Acropodite simple, directed medially. Telopodite basal zone with raised ridge (Fig. 124A, red arrow). Prefemoral spine pronounced and claw-like (Fig. 124B, red triangle). *Nannaria* “Ridgeway” specimens differ from “Moores Springs” based on the following combination of gonopodal characters: Prefemoral process small, curving ventrolaterally, not long, nearly straight as in “Moores Springs.” Acropodite with slight medial swelling (Fig. 124A, red oval), lacking in “Moores Springs.”

###### Nannaria

Taxon classificationAnimaliaPolydesmidaXystodesmidae

“Sagittata” incertae sedis

90DB9729-5DA5-5436-A75C-CC42F564C42D

Fig. 125

####### Material examined.

United States – **West Virginia** • 1 ♂; Raleigh County, Grandview State Park NE of Beckley; 37.8302°N, -81.0635°W; 22 Sep. 1962; R. Hoffman leg.; VMNH NAN0316. For detailed collection data see Suppl. material 7.

####### Hypothesized placement.

**Figure 125.**
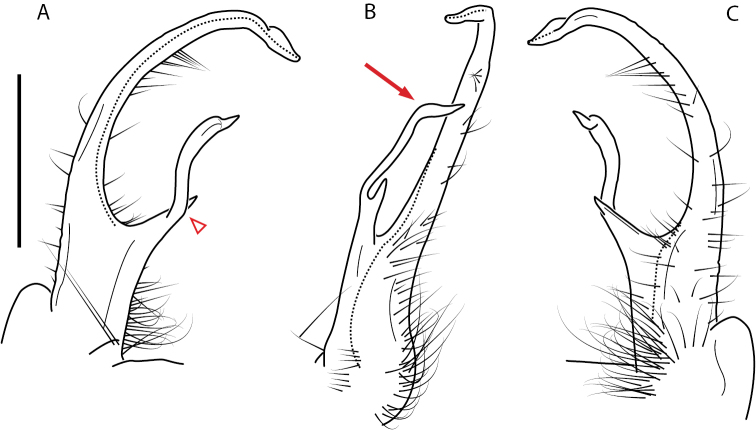
*Nannaria* “Sagittata” *incertae sedis* ♂ (VMNH, NAN0316) left gonopod **A** anterior view, red triangle indicates prefemoral process arising medially from prefemoral spine **B** medial view; red arrow indicates ventrally directed prefemoral process **C** posterior view. Scale bar: 0.5 mm.

*Nannaria* “Sagittata” specimens are hypothesized to be closely related to *Nannariatasskelsoae* sp. nov. based on close geographic proximity and the following combination of gonopodal characters. Acropodite gently curving medially before bending posteriorly at tip. Prefemoral process arising from pronounced prefemoral spine. *Nannaria* “Sagittata” specimens differ from *N.tasskelsoae* sp. nov. based on the following gonopodal characters: Acropodite tip serpentine and pointed, not rectangular and blunt as in *N.tasskelsoae* sp. nov. Prefemoral process serpentine and directed ventrally at tip, crossing acropodite in medial view (Fig. 125B, red arrow), not simple, curving medially as in *N.tasskelsoae* sp. nov. Prefemoral process arising medially from prefemoral spine, not dorsally as in *N.tasskelsoae* sp. nov. (Fig. 125A, red triangle).

**Figure 126.**
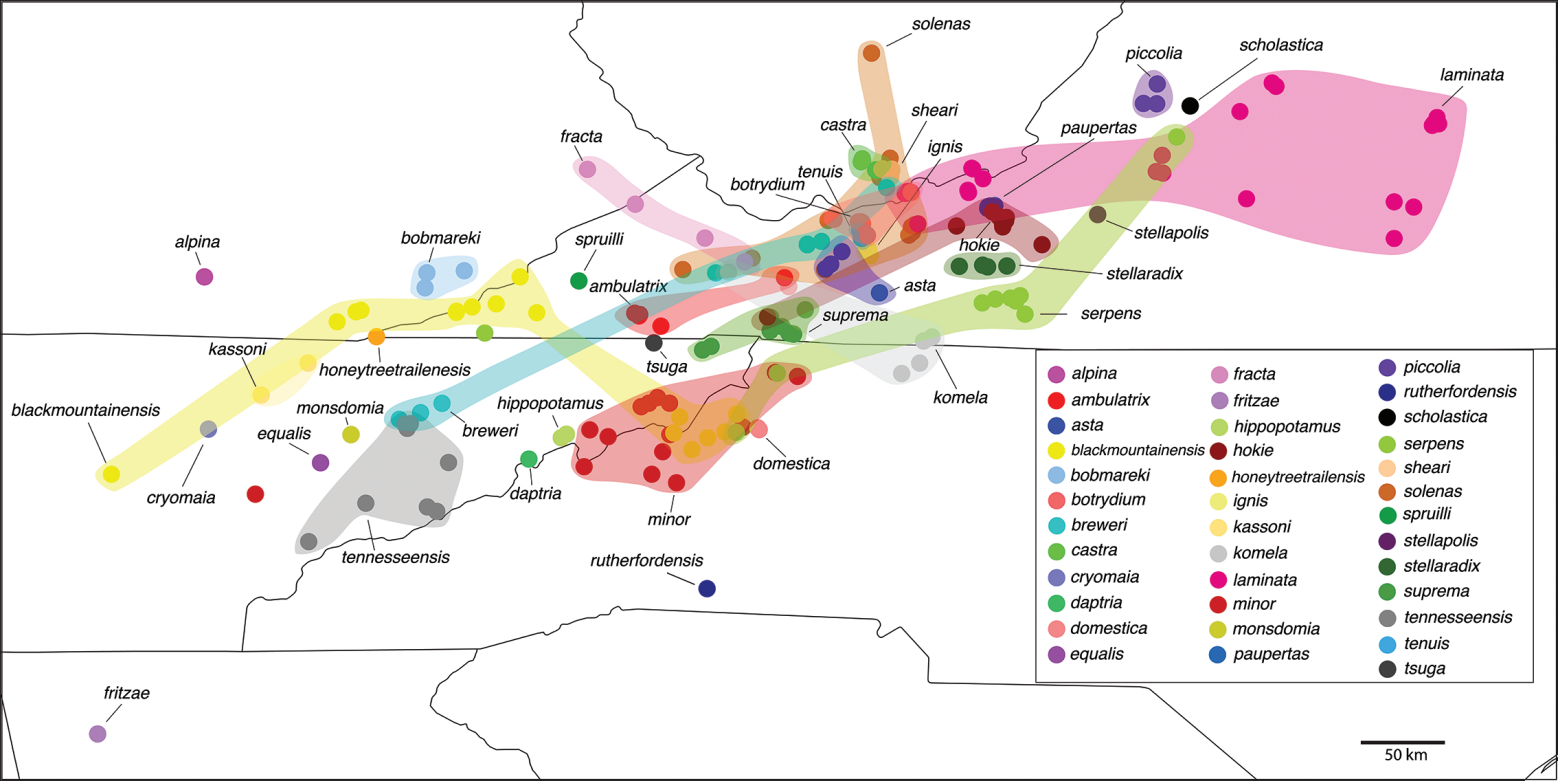
Distribution map of *Nannariaminor* species group collection localities in the central Appalachians.

**Figure 127.**
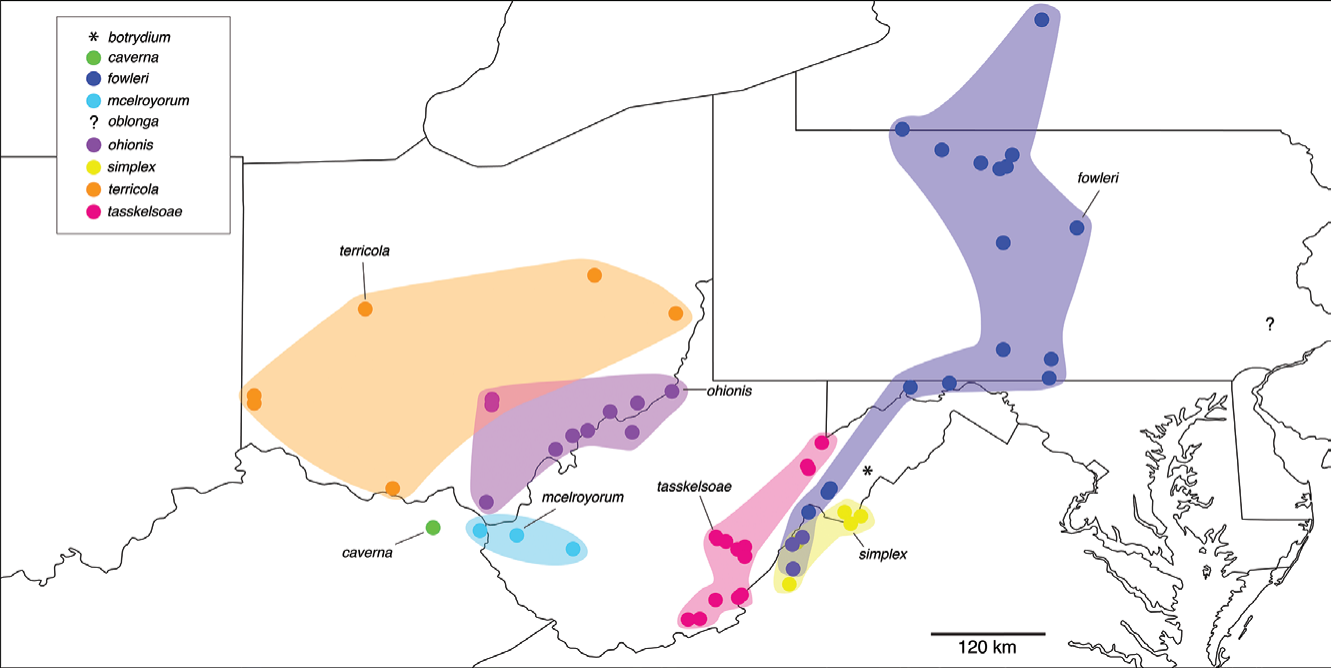
Distribution map of *Nannariaminor* species group collection localities in the northern Appalachians.

**Figure 128.**
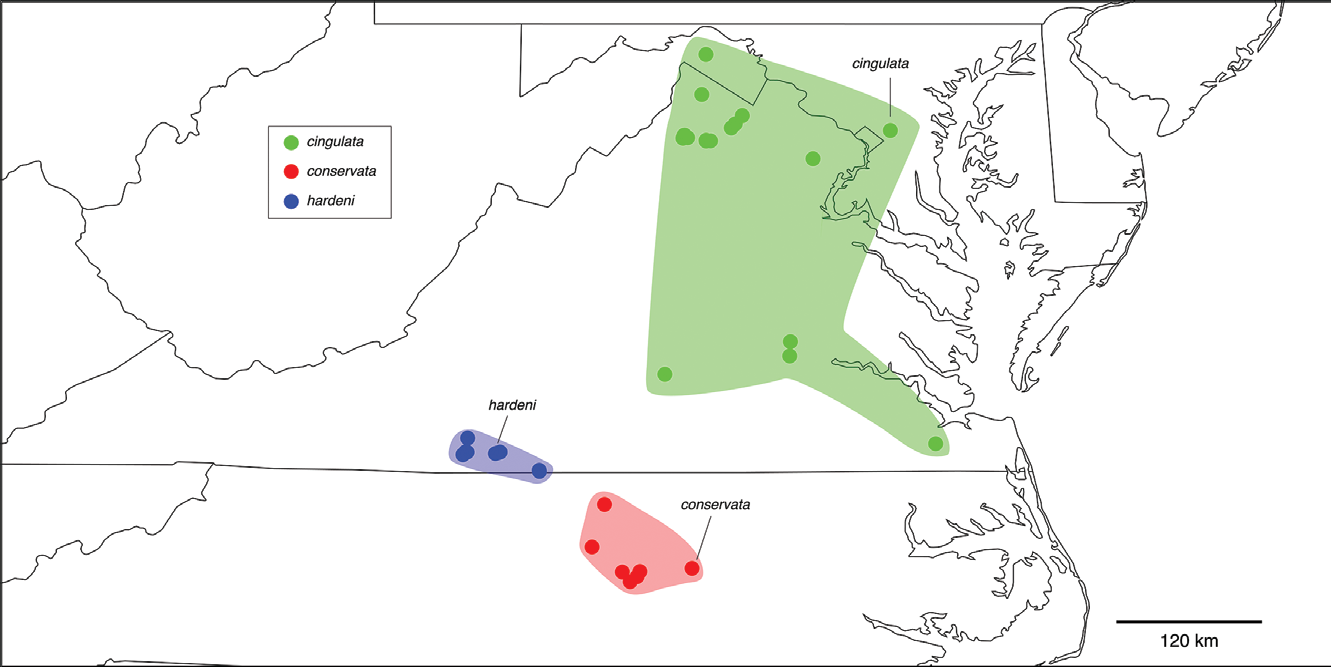
Distribution map of *Nannariaminor* species group collection localities in the Eastern Piedmont and coast.

**Figure 129.**
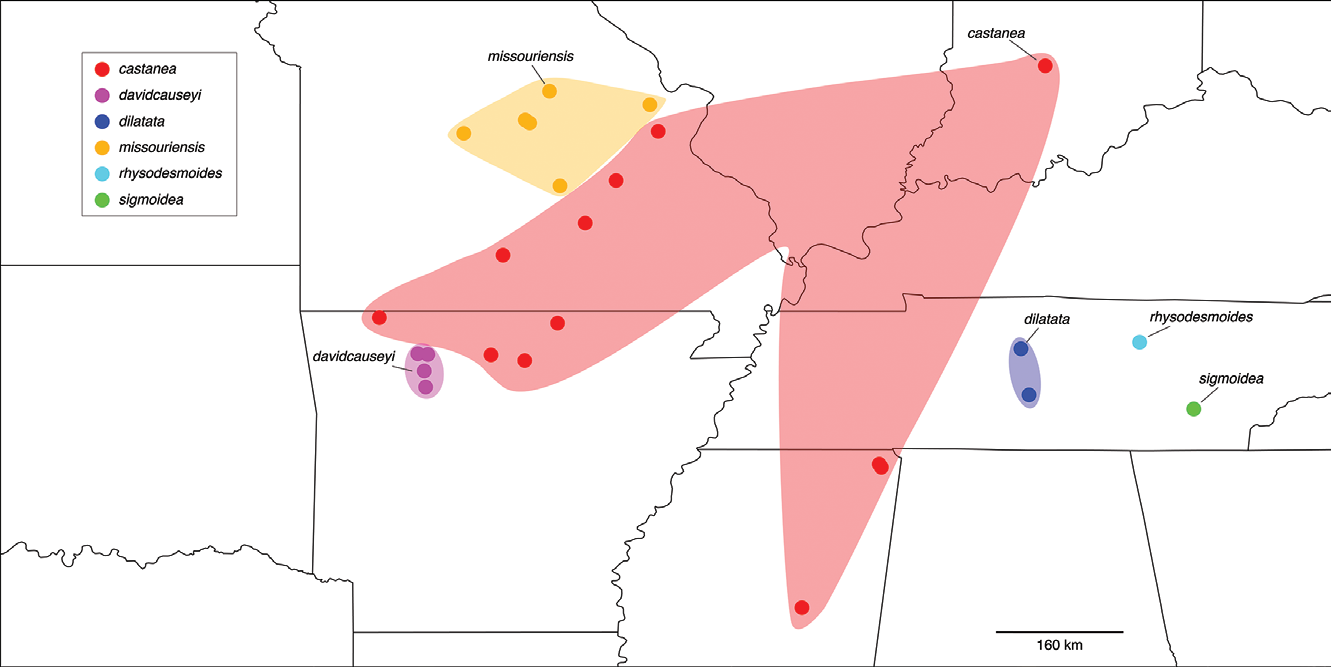
Distribution map of *Nannariaminor* species group collection localities west of the Appalachians.

## Discussion

We revised the genus *Nannaria* through a combination of molecular and morphological data, and provided the first molecular framework for this alpha-taxonomically poorly known and widespread group. Our molecular phylogeny supported previous molecular phylogenetic evidence that the tribe Nannariini is monophyletic within the Xystodesmidae, with *O.pulchella* sister to the remaining members of the tribe in the genus *Nannaria* (Suppl. material 3, Means et al. 2021). We described 35 new species, and confirmed that *Nannaria* contains two clades: the *wilsoni* and the *minor* species groups. The *wilsoni* species group occurs primarily in the central and southern Appalachian Mountains, while the *minor* species group is more widespread and occurs throughout the eastern and central U.S. and Canada. Within the *minor* species group, we found a considerable degree of morphological variation, even between closely related species, and a paucity of characters uniting species within clades (Fig. 113). We found several instances of close morphological similarity between distantly related species that are likely a result of convergence (e.g., *N.ignis* sp. nov. and *N.minor*). Means and Marek (2017) showed that morphological convergence between distantly related species within the family Xystodesmidae repeatedly evolved and was common, and could confuse taxonomy. Our findings suggest that morphology should not be used as the sole criteria for the taxonomic placement of species within *Nannaria*, and instead molecular phylogenetics ideally utilizing multiple genes should be used in combination with morphological and geographical data.

### *Nannariaminor* and *wilsoni* species groups

Within *Nannaria* there are two primary stem group clades, the *minor* and *wilsoni* species groups, both containing considerable undescribed species diversity. Within the *minor* species group, there are 18 distinct clades (Suppl. material 3), which vary in terms of the morphological similarity between their member species. Both the *minor* and *wilsoni* species groups were recovered as monophyletic (Suppl. material 3, *pp* = 1), suggesting an evolutionary divide between the two groups. This divide has been unrecognized in the past literature, perhaps due to the lack of an obvious geographical separation and/or morphological synapomorphies which could be used to consistently differentiate species of either group. Upon examination of morphological characters in context of the molecular phylogeny, however, we have found characters that are useful for distinguishing *wilsoni* versus *minor* species groups. First, the *minor* species group exhibit a prefemoral spine (Fig. 5, ps), spare *N.scholastica* sp. nov., and a small subset of species (*N.castra* sp. nov., *N.missouriensis*, *N.oblonga*, and *N.rhysodesmoides*). None of the *wilsoni* species group taxa exhibit this character. Second, the majority of *minor* species group taxa have large basal zones, in some species reaching over half of the length of the telopodite, while *wilsoni* species group taxa have reduced basal zones, rarely reaching a third of the length of the acropodite. Third, *minor* species group taxa have uniformly unmodified cyphopod receptacles, while many *wilsoni* species group taxa have highly modified cyphopod receptacles (discussed in an upcoming revision of the *wilsoni* species group). Hoffman (1949) suggested that the distance between the bases of the last pair of legs and the shape of the “preanal scale” (= hypoproct) could be used to separate *N.simplex* (*minor* species group) and *N.ericacea* (*wilsoni* species group); however, these differences are not found consistently throughout either group and are therefore not useful for differentiating the *minor* versus *wilsoni* groups. Future species-level identifications of the genus should use the morphology of the gonopods or DNA barcoding with COI (Suppl. material 4C).

### The utility of morphology in the *minor* species group

**Figure 130.**
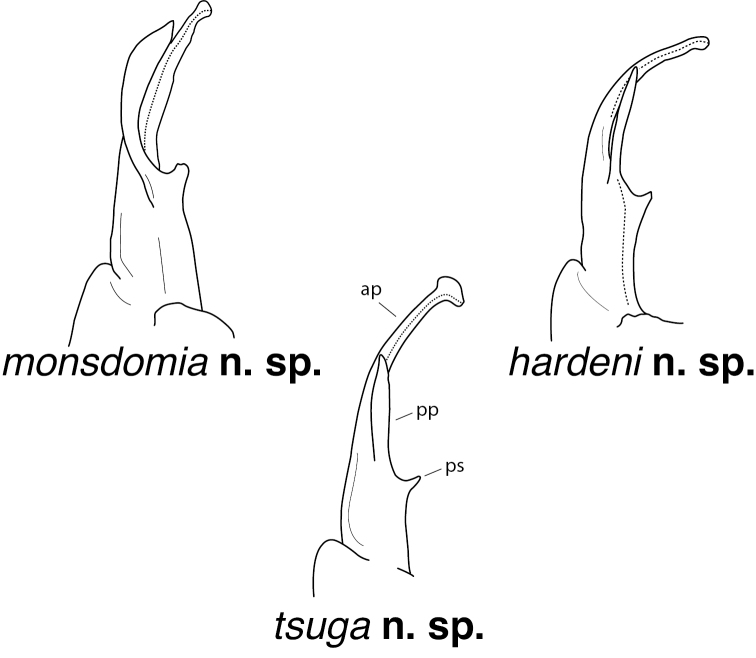
Example of morphological convergence between three distantly related species of *Nannaria*. In all three species, the prefemoral process (pp) arises separate from the prefemoral spine (ps), and crosses the acropodite (ap), despite the three species belonging to separate clades.

Previous descriptions of species in the genus *Nannaria* have been based solely on morphological characters, almost exclusively consisting of the male genitalia; however, morphological character scoring (Suppl. material 5) revealed little variation in non-genitalic somatic characters. Through molecular phylogenetic analysis of nearly 100 *minor* species group specimens, we have revealed the existence of 18 clades within the group, and demonstrated that morphological characters, while useful for species level diagnosis, are inadequate for determining evolutionary relationships among species. For example, the distinctive prefemoral process which was separated from the prefemoral spine, and arising from the prefemur and overlapping the acropodite in the anterior view is present in the species *N.monsdomia* sp. nov., *N.hardeni* sp. nov. and *N.tsuga* sp. nov. (Fig. 130). However, phylogenetic evidence indicated that *N.monsdomia* sp. nov., *N.hardeni* sp. nov. and *N.tsuga* sp. nov. all belong to separate clades, spread throughout the tree (Fig. 7), which suggest that this prefemoral process configuration evolved multiple times in the *minor* species group. The same is true for the telopodite basal zone, prefemoral spine, prefemur, and acropodite flange. We presumed that species with tall, thin basal zones would be closely related, such as in the case of *N.ambulatrix* sp. nov. and *N.tenuis* sp. nov.; however, these species are distantly related. Likewise, we expected that the prefemoral process and spine had diagnostic potential, yet both structures seem to vary considerably between closely related species, such as the prominent prefemoral spine in *N.paupertas* sp. nov., and the completely absent/reduced spine of its sister species, *N.castra* sp. nov. The prefemoral process and spine may aid in stabilizing the acropodite during mating, and perhaps allow for the repositioning of a spermatophore during copulation, as in the Parajulidae (Mathews and Bultman 1993); however, the function of the prefemoral structures have never been directly evaluated in *Nannaria* and therefore remain unknown. The position and shape of acropodite flanges, such as the can opener-like medial flange of *N.minor* (Fig. 106A) and *N.fritzae* sp. nov. (Fig. 55A) also seem to be inadequate diagnostic characters. Even multiple combinations of the basal zone, prefemoral spine, prefemur, and acropodite flange taken together do not indicate close evolutionary relationships: for example, the distinctive combination of a prominent lateral flange and thin acropodite basal zone found in the *N.ambulatrix* clade can also be found in the *N.minor* (e.g., *N.hippopotamus* sp. nov., Fig. 100), *N.ohionis* (e.g., *N.suprema* sp. nov., Fig. 69), *N.ignis* (e.g., *N.tenuis* sp. nov., Fig. 63), and *N.paupertas* clades (e.g., *N.castra* sp. nov., Fig. 20). In contrast, the *N.castanea* clade is the most morphologically definable group, with all members possessing long, thin acropodites, pronounced prefemoral spines and acropodite tip lateral flanges. There are no known species with long, thin, *N.castanea*-like acropodites that are not members of this clade.

Although sufficient for species-level identification within the *Nannaria*, morphology is a poor indicator of evolutionary relationship above the species level. These results support previous work by Means and Marek (2017) and Means et al. (2021), which showed that morphological homoplasy in gonopod morphology is common within the Xystodesmidae, and suggested that future investigations of evolutionary relationships between millipede taxa should include molecular evidence as an independent dataset.

### The phylogeographic history of the *minor* species group

From the molecular analysis presented here, the Appalachian Mountains—and more specifically, the Blue Ridge Mountains and Valley and Ridge physiographic regions—contain the greatest diversity of *Nannaria* species (Figs 125–127). Sixteen of the eighteen clades recovered in the phylogeny have members in Virginia, making this state the center of Nannariini diversity (Figs 126–129). It should be noted, however, that collecting efforts have been most intense in Virginia, and it is likely that many species of *Nannaria* remain undiscovered in other central Appalachian states, especially West Virginia, Kentucky, and Tennessee. Though it is difficult, without fossil evidence, to make statements about the age or movements of *Nannaria* species over evolutionary time, the placement of the putative relictual *N.scholastica* sp. nov. as the sister to the rest of the *minor* species group would suggest that the group originated in western Virginia before spreading throughout the eastern United States. With members of the *N.castanea* clade found west of the Mississippi, it is likely that *Nannaria* originated before the last ice age, and may have experienced multiple range contractions and expansions due to shifting environmental conditions. Although there seem to be congruent distributions between unrelated floral and faunal taxa, such as with plethodontid salamanders (Kozak 2017), the seemingly repeated patterns shown by phylogenetic analyses indicate continuous abiotic processes during the Paleogene (splitting, expansion, and retraction) mediated by cooling and warming that may have led to “pseudocongruent” distributions seen in other co-distributed taxa (Manos and Meireiles 2015).

Insight into the abiotic processes that led to the morphological and genetic patterns observed in *Nannaria* are necessary to help understand the diversification of the group. As a highly dispersal-limited group, environmental conditions would need to be relatively stable for long periods of time for populations to move great distances rapidly, for example in the *N.castanea* clade, where genetic variation between the eastern and western lineages is less than may be expected based on the nearly 800 km distance between the two lineages (Figs 126, 129). It is unclear why the *wilsoni* species group is limited to the Appalachian Mountains and does not occur west of the Mississippi River as in the *minor* species group (Fig. 1). The *minor* species group taxa are found in a wider variety of habitats than the *wilsoni* species group taxa, which appear to be tightly constrained to mesic forested areas. Whether the wider distribution of the *minor* species group is due to an apomorphic physiological adaptation, such as a resistance to desiccation, or some other means is unknown. Alternatively, the *wilsoni* species group may be a younger clade and had less time to expand its range; that the *wilsoni* species group is an older clade and experienced greater extinctions is a possibility as well but unlikely. Future studies of *Nannaria* should seek to understand abiotic factors of their habitat to illuminate the patterns of diversification in the group, and help clarify to what extent ecological factors influence speciation over temporal or other factors influencing evolutionary history.

### *Nannaria* habitat preferences and crypsis

The majority of *Nannaria* specimens were collected in mesic deciduous forests, often near streams, rivers, or other lotic habitats. In his description of *N.ericacea*, a member of the *wilsoni* species group, Hoffman (1949) concluded that both *N.ericacea* and *N.wilsoni* were often found in ericaceous habitats, i.e., habitats dominated by an abundance of plants in the family Ericaceae. *Rhododendron* groves seem to be an excellent indicator of suitable *Nannaria* habitat, perhaps due to their high moisture requirements and tolerance of water-saturated soils. Adult *Nannaria* are rarely encountered under the *Rhododendron* canopy, preferring instead the oak and maple groves interspersing or abutting stands of *Rhododendron*; while immature *Nannaria* are frequently encountered directly under *Rhododendron*. This suggests that the dark and impenetrable understory created by *Rhododendron* may be an ideal area for *Nannaria* to molt safely and avoid desiccation during the process of tanning and hardening the cuticle; though the thick, waxy leaves are likely inedible, thereby forcing adults to move outward from *Rhododendron* coves. Hemlock stands (*Tsuga* spp.) are a second good indicator of *Nannaria* presence, likely due to a similar need for a cool, wet environment. Within forested habitats, *Nannaria* were frequently found beneath the soil at the edges of rocks, tree basal zones, and roots, which may provide shelter and a stable, moist environment. Additionally, *Nannaria* appeared to prefer sloped hillsides, with little understory, and sandy or loamy soil that drains well and most likely facilitates burrowing.

Unlike the majority of xystodesmid species that are brightly colored and active on the surface (Marek and Bond 2009), *Nannaria* were often found burrowed 1–2 cm beneath the soil and are drab in coloration, with a lack of strongly contrasting pattern elements. Burrowing, and behavioral concealment, is likely related to their drab coloration as part of an overall cryptic strategy (Ruxton et al. 2018). Dominant color patterns of *Nannaria*, e.g., small red or white spots on either paranota with a dark background and lack of additional pattern elements such as spots on the prozonite (as in some *Apheloriapolychroma* with four large yellow spots [Marek et al. 2018]), lend themselves to a cryptic appearance. Red light has been shown to be a dominant color in forest sub-canopies and red body coloration appears to be camouflaged in these habitats (Wilson et al. 2007). In addition, the dominant color pattern of *Nannaria* (red, white, rarely orange) may be non-functional and an ancestral polymorphism that was retained, and perhaps later minimized to small spots. Twenty-three specimens had metatergal stripes, but these were often faint and rarely found on fully tanned adults, suggesting that the stripes may disappear with tanning and hardening of the cuticle in most individuals. Whitehead and Shelley (1992) speculated that the general lack of pigmentation in *Nannaria* may constitute a form of mimicry with immature apheloriines, which as a result of a lack of a tanned fully sclerotized cuticle appear white, though this hypothesis remains untested. We suggest that the lack of pigmentation is due to their small size, perhaps retention of ancestral color pattern elements, and cryptic lifestyle, where bright coloration would be less beneficial. The similarly-sized *Oenomaeapulchella* has stripes and not small paranotal spots as in *Nannaria* (Fig. 3). Specimens of *Oenomaeapulchella*, like *Nannaria* species, do not generally produce a noticeable odor of benzaldehyde advertising their production of hydrogen cyanide. A larger comparative analysis of cyanide glands and defensive chemicals of *O.pulchella*, *Nannaria* species, and related members of the Xystodesmidae may illuminate the role that color and associated chemical defense plays in the Nannariini.

## Conclusions

Since the description of the genus nearly a century ago, *Nannaria* has received little taxonomic attention beyond the occasional species description. Here, we provide the first revision of the Nannariini using molecular evidence from both genera (*Oenomaea* and *Nannaria*). We reveal the existence of two distinct clades—the *minor* and *wilsoni* species groups, and describe 35 new species within the highly species diverse and widespread *minor* species group. In an upcoming revision of the *wilsoni* species group, led by DAH, 17 new species will be described. With 52 new species of these cryptically colored and often difficult-to-find polydesmidan millipedes, the *Nannaria* represent a taxon that is among the least known α-taxonomically of any animal group in North America. From this analysis, we provide evidence of 35 undescribed species within the *minor* species group and 11 additional putative species. We have shown that *Nannaria* has many instances of morphological convergence confounding morphology-based species concepts. This study shows a 10% mean difference amongst species in the COI barcoding region, and establishes a large barcoding database of the genus that provides groundwork for future investigations of speciation and diversity within the *Nannaria*. Over 70% of species within the *Nannariaminor* species group are micro-range endemics, meaning they have distributions of less than 1000 km^2^ (Harvey 2002; Means and Marek 2017). The highest density of known species diversity occurs in Virginia, primarily in the Ridge and Valley ecoregion. This topographically complex area is an exceptional repository of high species richness and relative rarity of taxa (Stein et al. 2000). Deforestation, surface mining, and habitat loss disproportionately threaten these groups with narrow endemism, strict habitat requirements, and low dispersal rates such as *Nannaria*. The loss of micro-range endemics and other rare species permanently hinders our ability to conserve biodiversity, reconstruct phylogenetic history, and study diversity of the life on the planet.

## Supplementary Material

XML Treatment for Nannaria

XML Treatment for Nannaria scholastica

XML Treatment for Nannaria domestica

XML Treatment for Nannaria komela

XML Treatment for Nannaria fowleri

XML Treatment for Nannaria piccolia

XML Treatment for Nannaria simplex

XML Treatment for Nannaria castra

XML Treatment for Nannaria paupertas

XML Treatment for Nannaria caverna

XML Treatment for Nannaria mcelroyorum

XML Treatment for Nannaria hardeni

XML Treatment for Nannaria serpens

XML Treatment for Nannaria castanea

XML Treatment for Nannaria davidcauseyi

XML Treatment for Nannaria hokie

XML Treatment for Nannaria missouriensis

XML Treatment for Nannaria stellapolis

XML Treatment for Nannaria stellaradix

XML Treatment for Nannaria cingulata

XML Treatment for Nannaria laminata

XML Treatment for Nannaria tasskelsoae

XML Treatment for Nannaria breweri

XML Treatment for Nannaria equalis

XML Treatment for Nannaria fritzae

XML Treatment for Nannaria monsdomia

XML Treatment for Nannaria tennesseensis

XML Treatment for Nannaria ignis

XML Treatment for Nannaria tenuis

XML Treatment for Nannaria ohionis

XML Treatment for Nannaria sheari

XML Treatment for Nannaria suprema

XML Treatment for Nannaria alpina

XML Treatment for Nannaria blackmountainensis

XML Treatment for Nannaria bobmareki

XML Treatment for Nannaria dilatata

XML Treatment for Nannaria fracta

XML Treatment for Nannaria solenas

XML Treatment for Nannaria spruilli

XML Treatment for Nannaria terricola

XML Treatment for Nannaria ambulatrix

XML Treatment for Nannaria asta

XML Treatment for Nannaria botrydium

XML Treatment for Nannaria tsuga

XML Treatment for Nannaria cryomaia

XML Treatment for Nannaria daptria

XML Treatment for Nannaria hippopotamus

XML Treatment for Nannaria honeytreetrailensis

XML Treatment for Nannaria kassoni

XML Treatment for Nannaria minor

XML Treatment for Nannaria rhysodesmoides

XML Treatment for Nannaria conservata

XML Treatment for Nannaria oblonga

XML Treatment for Nannaria rutherfordensis

XML Treatment for Nannaria sigmoidea

XML Treatment for Nannaria

XML Treatment for Nannaria

XML Treatment for Nannaria

XML Treatment for Nannaria

XML Treatment for Nannaria

XML Treatment for Nannaria

XML Treatment for Nannaria

XML Treatment for Nannaria

XML Treatment for Nannaria

XML Treatment for Nannaria

XML Treatment for Nannaria

## Supplementary materials

Supplementary material 1 Suppl. material 1 Data type List of genes Explanation note List of gene regions explored for use in taxonomic analyses of *Nannaria*. https://binary.pensoft.net/file/531234This dataset is made available under the Open Database License (http://opendatacommons.org/licenses/odbl/1.0/). The Open Database License (ODbL) is a license agreement intended to allow users to freely share, modify, and use this Dataset while maintaining this same freedom for others, provided that the original source and author(s) are credited.Means JC, Hennen DA, Marek PM

Supplementary material 2 Suppl. material 2 Data type List of taxa & NCBI accession numbers Explanation note List of taxa used in molecular analyses, organized alphabetically by genus and then species. Acc# refer to the NCBI Genbank database. All specimens available from the corresponding author by request and stored in the Virginia Tech Insect Collection, Blacksburg, Virginia, USA. https://binary.pensoft.net/file/531235This dataset is made available under the Open Database License (http://opendatacommons.org/licenses/odbl/1.0/). The Open Database License (ODbL) is a license agreement intended to allow users to freely share, modify, and use this Dataset while maintaining this same freedom for others, provided that the original source and author(s) are credited.Means JC, Hennen DA, Marek PM

Supplementary material 3 Suppl. material 3 Data type Phylogenetic Explanation note Molecular phylogeny of *Nannaria*. https://binary.pensoft.net/file/531236This dataset is made available under the Open Database License (http://opendatacommons.org/licenses/odbl/1.0/). The Open Database License (ODbL) is a license agreement intended to allow users to freely share, modify, and use this Dataset while maintaining this same freedom for others, provided that the original source and author(s) are credited.Means JC, Hennen DA, Marek PM

Supplementary material 4 Suppl. material 4 Data type Phylogenetic Explanation note Individual gene phylogenies of *Nannaria*. https://binary.pensoft.net/file/531237This dataset is made available under the Open Database License (http://opendatacommons.org/licenses/odbl/1.0/). The Open Database License (ODbL) is a license agreement intended to allow users to freely share, modify, and use this Dataset while maintaining this same freedom for others, provided that the original source and author(s) are credited.Means JC, Hennen DA, Marek PM

Supplementary material 5 Suppl. material 5 Data type Morphological scoring matrix Explanation note Morphological scoring matrix for *Nannariaminor* species group. https://binary.pensoft.net/file/531238This dataset is made available under the Open Database License (http://opendatacommons.org/licenses/odbl/1.0/). The Open Database License (ODbL) is a license agreement intended to allow users to freely share, modify, and use this Dataset while maintaining this same freedom for others, provided that the original source and author(s) are credited.Means JC, Hennen DA, Marek PM

Supplementary material 6 Suppl. material 6 Data type Key to morphological matrix Explanation note Key to morphological characters used to score *Nannaria*. https://binary.pensoft.net/file/531239This dataset is made available under the Open Database License (http://opendatacommons.org/licenses/odbl/1.0/). The Open Database License (ODbL) is a license agreement intended to allow users to freely share, modify, and use this Dataset while maintaining this same freedom for others, provided that the original source and author(s) are credited.Means JC, Hennen DA, Marek PM

Supplementary material 7 Suppl. material 7 Data type occurrences Explanation note Materials examined. https://binary.pensoft.net/file/531240This dataset is made available under the Open Database License (http://opendatacommons.org/licenses/odbl/1.0/). The Open Database License (ODbL) is a license agreement intended to allow users to freely share, modify, and use this Dataset while maintaining this same freedom for others, provided that the original source and author(s) are credited.Means JC, Hennen DA, Marek PM

Supplementary material 8 Suppl. material 8 Data type Species group distributions Explanation note A .kmz file of *Nannariaminor* species group distributions for use in programs such as Google Earth (Alphabet Inc., Mountain View, California). https://binary.pensoft.net/file/531241This dataset is made available under the Open Database License (http://opendatacommons.org/licenses/odbl/1.0/). The Open Database License (ODbL) is a license agreement intended to allow users to freely share, modify, and use this Dataset while maintaining this same freedom for others, provided that the original source and author(s) are credited.Means JC, Hennen DA, Marek PM
