# Supplementary material for: A revision of the minor species group in the millipede genus Nannaria Chamberlin, 1918 (Diplopoda, Polydesmida, Xystodesmidae)
Source: Zookeys. 2021 Apr 13;1030:1–180. doi: 10.3897/zookeys.1030.62544 (PMC8060247; doi:10.3897/zookeys.1030.62544)
Supplement: Supplementary material 1 — Suppl. material 1 [file zookeys-1030-001-s001.docx]

| **Supplementary Material 1.** List of gene regions explored for use in taxonomic analyses of *Nannaria*. | | | |
| --- | --- | --- | --- |
| **Gene Region** | **Present** | **Amplification success** | **Sequencing success** |
| 16S | Y | > 90% | > 90% |
| alpha-enolase | Y | < 15% | - |
| Arginine Kinase | Y | > 90% | < 50% |
| CAD | N | - | - |
| CHP | N | - | - |
| CO2 | N | - | - |
| Cytb | N | - | - |
| EF1g | Y | > 90% | < 50% |
| EV12A | N | - | - |
| FAM43A | N | - | - |
| fbox | Y | > 90% | > 90% |
| hyp gene | N | - | - |
| MID1 | N | - | - |
| PEPCK | Y | < 50% | - |
| PP2A | N | - | - |
| PTP | N | - | - |
| ribonuclease P | N | - | - |
| RPB1 | Y | > 90% | > 90% |
| RyR | N | - | - |
| sdccag1 | N | - | - |
| SKiip | Y | < 15% | - |
| SPTA1 | Y | > 90% | < 50% |
| tartan | N | - | - |
| TP1 | N | - | - |
| UROD | Y | < 50% | - |
| Wingless | N | - | - |
| Zinc finger | N | - | - |
