# Supplementary material for: A revision of the minor species group in the millipede genus Nannaria Chamberlin, 1918 (Diplopoda, Polydesmida, Xystodesmidae)
Source: Zookeys. 2021 Apr 13;1030:1–180. doi: 10.3897/zookeys.1030.62544 (PMC8060247; doi:10.3897/zookeys.1030.62544)
Supplement: Supplementary material 2 — Suppl. material 2 [file zookeys-1030-001-s002.docx]

| **Supplementary Material 2**. List of taxa used in molecular analyses, organized alphabetically by genus and then species. Acc# refer to the NCBI GenBank database. All specimens available from the corresponding author by request and stored in the Virginia Tech Insect Collection, Blacksburg, Virginia, USA | | | | | | | | | | |
| --- | --- | --- | --- | --- | --- | --- | --- | --- | --- | --- |
| **Taxon** | **Specimen number** | **Latitude** | **Longitude** | **State, County** | **Acc# 16S** | **Acc# EF1a** | **Acc# 28S** | **Acc# COI** | **Acc# RNApol2** | **ACC# fbox** |
| **Xystodesmidae** |  |  |  |  |  |  |  |  |  |  |
| **Rhysodesmini** |  |  |  |  |  |  |  |  |  |  |
| *Howellaria deuturkiana* (Causey, 1942) | MPE00504 | 35.08157 | -83.23599 | North Carolina, Macon | **MF948809** | **MF953766** | **MF953664** | **MF953683** | **-** | **-** |
| *Caralinda causeyae* Shelley, 1983 | MPE02387 | 30.74989 | -83.5575 | Georgia, Brooks | **MN658224** | **MN719671** | **-** | **MN699734** | **-** | **-** |
| *Cherokia georgiana* (Bollman, 1889) | SPC000354 | 35.06337 | -83.43687 | North Carolina, Macon | **DQ490695** | **MF953802** | **MF948851** | **MF953726** | **-** | **-** |
| *Dicellarius atlanta* (Chamberlin, 1946) | SPC000428 | 33.25058 | -83.92334 | Georgia, Butts | **DQ490648** | **KR136044** | **KR135890** | **KR135992** | **-** | **-** |
| *Erdelyia saucra* Hoffman, 1962 | MPE03313 | 35.12418 | -83.53927 | North Carolina, Macon | **MN658233** | **MN719686** | **-** | **MN699743** | **-** | **-** |
| *Gonoessa furcata* Shelley, 1984 | MPE03304 | 32.17443 | -86.36858 | Alabama, Montgomery | **MN658246** | **MN719684** | **MN817826** | **MN699757** | **-** | **-** |
| *Gyalostethus monticolens* (Chamberlin, 1951) | SPC001008 | 36.76070 | -83.14000 | Kentucky, Harlan | **EU127868** | **MF953824** | **MF948875** | **MF953755** | **-** | **-** |
| *Pachydesmus incursus* Chamberlin, 1939 | SPC000380 | 33.13690 | -81.43390 | South Carolina, Barnwell | **DQ490696** | **MF953804** | **MF948852** | **MF953728** | **-** | **-** |
| *Pleuroloma flavipes* Rafinesque, 1820 | SPC000338 | 36.92891 | -83.19141 | Kentucky, Bell | **DQ490692** | **MF953800** | **MF948849** | **MF953724** | **-** | **-** |
| *Rhysodesmus texicolens* Chamberlin, 1938 | MPE02388 | 25.98839 | -97.56482 | Texas, Cameron | **MN658265** | **-** | **-** | **MN699795** | **-** | **-** |
| *Stenodesmus tuobitus* (Chamberlin, 1910) | MTX0199 | 32.93085 | -108.0137 | New Mexico, Grant | **KR135967** | **MF953771** | **KR135918** | **KR136021** | **-** | **-** |
|  |  |  |  |  |  |  |  |  |  |  |
| **Euryurini** |  |  |  |  |  |  |  |  |  |  |
| *Eurymerodesmus mundus* Chamberlin, 1931 | Emun2 |  |  |  | **MN658235** | **MN719644** | **MN817823** | **MN699745** | **-** | **-** |
|  |  |  |  |  |  |  |  |  |  |  |
| **Nannarini** |  |  |  |  |  |  |  |  |  |  |
| *Nannaria* | MPE00156 | 37.45819 | -79.62956 | Virginia, Bedford | **MT302730** | **MT319918** | **MT319825** | **MT311374** | **MT503348** | **MT349970** |
| *Nannaria* | MPE00234 | 36.96624 | -80.41797 | Virginia, Montgomery | **MT302731** | **MT319921** | **MT319829** | **MT311378** | **MT503352** | **MT349974** |
| *Nannaria* | MPE00585 | 38.65502 | -80.07136 | Virginia, Randolph | **MT302702** | **MT319927** | **-** | **MT311383** | **MT503360** | **MT349984** |
| *Nannaria* | MPE00792 | 37.40352 | -80.34148 | Virginia, Craig | **MT302710** | **MT319928** | **MT319836** | **MT311384** | **-** | **MT349985** |
| *Nannaria* | MPE01236 | 37.9421 | -78.47794 | Virginia, Albemarle | **MT302724** | **MT319932** | **MT319844** | **MT311394** | **MT503375** | **MT350001** |
| *Nannaria* | MPE01465 | 35.02885 | -83.28239 | North Carolina, Macon | **MN817798** | **MN719660** | **MN817839** | **MN699770** | **MT503379** | **MT350005** |
| *Nannaria* | MPE01690 | 39.49131 | -82.57975 | Ohio, Hocking | **MT302670** | **MT319936** | **-** | **-** | **MT503382** | **MT350009** |
| *Nannaria* | MPE02059 | 36.68667 | -80.44151 | Virginia, Patrick | **MT302732** | **MT319940** | **MT319847** | **MT311400** | **MT503387** | **MT350015** |
| *Nannaria* | MPE02060 | 38.3605 | -79.20483 | Virginia, Augusta | **MT302741** | **-** | **-** | **MT311401** | **MT503388** | **MT350016** |
| *Nannaria* | MPE02108 | 38.30893 | -79.3603 | Virginia, Augusta | **MT302742** | **MT319944** | **MT319851** | **-** | **MT503392** | **MT350020** |
| *Nannaria* | MPE02109 | 38.59485 | -79.19827 | West Virginia, Pendleton | **MT302740** | **MT319945** | **MT319852** | **MT311405** | **MT503393** | **MT350021** |
| *Nannaria* | MPE02110 | 37.85263 | -79.85123 | Virginia, Alleghany | **MT302739** | **MT319946** | **MT319853** | **-** | **MT503394** | **MT350022** |
| *Nannaria* | MPE02117 | 35.83665 | -78.76237 | North Carolina, Wake | **-** | **MT319948** | **MT319854** | **MT311407** | **-** | **-** |
| *Nannaria* | MPE02242 | 38.18027 | -81.83841 | West Virginia, Boone | **MT302675** | **MT319949** | **MT319858** | **MT311412** | **MT503401** | **MT350029** |
| *Nannaria* | MPE02500 | 37.44932 | -80.52018 | Virginia, Giles | **MT302729** | **-** | **MT319865** | **MT311419** | **MT503410** | **MT350038** |
| *Nannaria* | MPE02801 | 37.46674 | -83.91641 | Kentucky, Jackson | **MT302661** | **MT319959** | **MT319869** | **MT311424** | **MT503419** | **MT350047** |
| *Nannaria* | MPE02831 | 37.23214 | -80.08678 | Virginia, Roanoke | **MT302648** | **-** | **-** | **MT311425** | **MT503420** | **MT350048** |
| *Nannaria* | MPE02879 | 36.3973 | -81.05179 | North Carolina, Wilkes | **MT302715** | **-** | **MT319871** | **MT311427** | **MT503422** | **MT350050** |
| *Nannaria* | MPE02891 | 36.56056 | -80.74694 | North Carolina, Surry | **MT302722** | **-** | **MT319872** | **MT311428** | **MT503423** | **MT350051** |
| *Nannaria* | MPE02900 | 37.94297 | -79.13828 | Virginia, Augusta | **MT302743** | **MT319961** | **MT319873** | **-** | **MT503424** | **MT350052** |
| *Nannaria* | MPE02937 | 36.50222 | -82.4825 | Tennessee, Sullivan | **MT302692** | **-** | **-** | **MT311429** | **MT503425** | **MT350053** |
| *Nannaria* | MPE02983 | 36.7941 | -82.09048 | Virginia, Washington | **-** | **-** | **MT319874** | **MT311430** | **MT503426** | **MT350054** |
| *Nannaria* | MPE03145 | 37.8915 | -84.3972 | Kentucky, Fayette | **MT302671** | **MT319968** | **MT319881** | **-** | **MT503432** | **MT350061** |
| *Nannaria* | MPE03311 | 35.12418 | -83.53927 | North Carolina, Macon | **-** | **MT319971** | **MT319885** | **-** | **MT503436** | **MT350065** |
| *Nannaria* | MPE03317 | 34.83647 | -83.77109 | Georgia, Towns | **MT302752** | **MT319972** | **MT319886** | **-** | **MT503437** | **MT350066** |
| *Nannaria* | MPE03430 | 35.43156 | -82.24359 | North Carolina, Rutherford | **-** | **MT319973** | **MT319887** | **-** | **MT503438** | **MT350067** |
| *Nannaria* | MPE03469 | 37.34528 | -80.91056 | West Virginia, Mercer | **MT302689** | **-** | **MT319888** | **MT311437** | **MT503439** | **MT350068** |
| *Nannaria* | MPE03487 | 37.79563 | -79.44273 | Virginia, Rockingham | **-** | **MT319975** | **MT319890** | **MT311439** | **-** | **MT350071** |
| *Nannaria* | MPE03492 | 37.90211 | -79.58829 | Virginia, Rockbridge | **MT302738** | **MT319976** | **MT319891** | **-** | **MT503441** | **MT350072** |
| *Nannaria* | MPE03639 | 38.06589 | -77.38031 | Virginia, Caroline | **MT302711** | **MT319977** | **MT319893** | **MT311441** | **MT503443** | **MT350074** |
| *Nannaria* | MPE03655 | 36.1913 | -81.60234 | North Carolina, Watauga | **MT302657** | **MT319979** | **-** | **MT311443** | **MT503445** | **-** |
| *Nannaria* | MPE03674 | 38.30719 | -79.76994 | Virginia, Highland | **MT302718** | **-** | **MT319895** | **MT311444** | **MT503446** | **MT350076** |
| *Nannaria* | MPE04027 | 36.75215 | -80.60795 | Virginia, Carroll | **MT302704** | **-** | **-** | **MT311447** | **-** | **MT350079** |
| *Nannaria* | MPE04051 | 36.38979 | -82.36777 | Tennessee, Washington | **MT302682** | **-** | **MT319898** | **MT311449** | **MT503450** | **MT350081** |
| *Nannaria* | MPE04087 | 37.37952 | -80.25034 | Virginia, Craig | **MT302709** | **-** | **-** | **MT311452** | **MT503452** | **MT350084** |
| *Nannaria* | MPE04175 | 36.4329 | -82.07241 | Tennessee, Carter | **MT302713** | **-** | **MT319907** | **MT311457** | **MT503458** | **MT350091** |
| *Nannaria* | MPE04200 | 37.5833 | -80.16049 | Virginia, Craig | **MT302726** | **MT319984** | **MT319908** | **-** | **MT503459** | **MT350092** |
| *Nannaria* | SPC000674 | 37.68827 | -80.3644 | West Virginia, Monroe | **MT302728** | **MT319986** | **-** | **-** | **-** | **MT350095** |
| *Nannaria* | SPC000689 | 38.119 | -80.154 | West Virginia, Pocahontas | **MT302697** | **-** | **-** | **MT311459** | **MT503462** | **MT350096** |
| *N. aenigma* Means et al., 2020 | MPE00268 | 37.01488 | -81.4101 | Virginia, Tazewell | **MT302733** | **MT319923** | **MT319832** | **-** | **MT503355** | **MT349977** |
| *N. aenigma* Means et al., 2020 | MPE01008 | 37.19245 | -81.17036 | Virginia, Bland | **MN817805** | **MN719649** | **MN817838** | **MN699769** | **MT503367** | **MT349992** |
| *N. aenigma* Means et al., 2020 | MPE01060 | 37.04938 | -81.1161 | Virginia, Bland | **MT302737** | **-** | **MT319841** | **MT311389** | **MT503369** | **MT349994** |
| *N. aenigma* Means et al., 2020 | MPE01648 | 36.94777 | -81.82424 | Virginia, Washington | **MT302734** | **MT319934** | **-** | **MT311396** | **MT503381** | **MT350007** |
| *N. aenigma* Means et al., 2020 | MPE02106 | 36.8304 | -81.95938 | Virginia, Washington | **MT302735** | **MT319942** | **MT319849** | **MT311403** | **MT503390** | **MT350018** |
| *N. alpine* Means, Hennen, & Marek, 2020 | MPE03150 | 36.91564 | -84.51827 | Kentucky, Pulaski | **MT302658** | **-** | **MT319882** | **MT311436** | **MT503433** | **MT350062** |
| *N. ambulatrix* Means, Hennen, & Marek, 2020 | MPE00178 | 36.91126 | -81.53172 | Virginia, Smyth | **-** | **-** | **MT319826** | **MT311375** | **MT503349** | **MT349971** |
| *Nannaria* sp. nov. ‘Amicolola’ | MPE01230 | 34.57534 | -84.17575 | Georgia, Lumpkin | **MT302751** | **-** | **-** | **MT311393** | **MT503374** | **MT350000** |
| *N. astavalla* Means, Hennen, & Marek, 2020 | MPE02419 | 36.9586 | -81.31893 | Virginia, Wythe | **-** | **MT319952** | **MT319862** | **MT311416** | **MT503407** | **MT350035** |
| *N. austricola* Hoffman, 1950 | SPC000352 | 35.06337 | -83.43687 | North Carolina, Macon | **MT302749** | **MT319985** | **MT319909** | **-** | **MT503460** | **MT350093** |
| *N*. *blackmountainensis* Means, Hennen, & Marek, 2020 | SPC000652 | 36.73285 | -83.22161 | Kentucky, Harlan | **MT302660** | **-** | **MT319910** | **MT311458** | **MT503461** | **MT350094** |
| *N*. *blackmountainensis* Means, Hennen, & Marek, 2020 | SPC001090 | 36.91565 | -82.90308 | Kentucky, Harlan | **MT302659** | **-** | **MT319912** | **MT311462** | **MT503465** | **MT350098** |
| *N*. *bobmareki* Means, Hennen, & Marek, 2020 | MMC0138 | 36.87201 | -82.48473 | Virginia, Scott | **MT302685** | **-** | **-** | **MT311368** | **MT503343** | **MT349964** |
| *N*. *bobmareki* Means, Hennen, & Marek, 2020 | SPC001019 | 36.93645 | -83.37294 | Kentucky, Leslie | **MT302668** | **MT319988** | **MT319911** | **MT311461** | **MT503464** | **MT350097** |
| *N. botrydia* Means, Hennen, & Marek, 2020 | MPE01009 | 37.19309 | -81.18272 | Virginia, Bland | **MT302687** | **MT319929** | **-** | **MT311388** | **MT503368** | **MT349993** |
| *N*. *breweri* Means, Hennen, & Marek, 2020 | MPE02191 | 36.21672 | -83.40559 | Tennessee, Hamblen | **MT302683** | **-** | **MT319856** | **MT311410** | **MT503399** | **MT350027** |
| *N*. *breweri* Means, Hennen, & Marek, 2020 | SPC001167 | 36.21605 | -83.4057 | Tennessee, Hamblen | **MT302684** | **MT319989** | **-** | **MT311463** | **MT503466** | **MT350099** |
| *N. castanea* (McNeill, 1887) | MPE02789 | 34.8051 | -88.30634 | Mississippi, Tishomingo | **MN817796** | **MN719677** | **MN817829** | **MN699760** | **MT503416** | **MT350044** |
| *N. castanea* (McNeill, 1887) | MPE02797 | 36.43071 | -93.75764 | Arkansas, Carroll | **MN817797** | **MN719678** | **MN817831** | **MN699761** | **MT503417** | **MT350045** |
| *N. castra* Means, Hennen, & Marek, 2020 | MPE03470 | 37.51472 | -81.12972 | West Virginia, Mercer | **MT302705** | **-** | **MT319889** | **-** | **-** | **MT350069** |
| *N. caverna* Means, Hennen, & Marek, 2020 | MPE03139 | 38.37383 | -83.1142 | Kentucky, Carter | **MT302653** | **MT319967** | **MT319880** | **MT311435** | **-** | **MT350060** |
| *N. cingulata* Means, Hennen, & Marek, 2020 | MPE01881 | 39.07173 | -77.912 | Virginia, Clarke | **MT302698** | **MT319939** | **-** | **MT311399** | **MT503385** | **MT350013** |
| *N. cingulata* Means, Hennen, & Marek, 2020 | MPE02324 | 38.93326 | -78.32075 | Virginia, Shenandoah | **MT302699** | **MT319950** | **MT319860** | **MT311414** | **MT503403** | **MT350031** |
| *Nannaria*  sp. nov. ‘Cratagae’ | MPE01222 | 35.66289 | -85.34983 | Tennessee, Van Buren | **MT302723** | **MT319931** | **-** | **-** | **MT503373** | **MT349999** |
| *N. cryomaia* Means, Hennen, & Marek, 2020 | MPE02642 | 36.13206 | -84.4978 | Tennessee, Morgan | **-** | **MT319957** | **-** | **-** | **MT503414** | **MT350042** |
| *N*. *honeytreetrailensis* Means, Hennen, & Marek, 2020 | MMC0334 | 36.6078 | -83.6322 | Virginia, Lee | **MT302693** | **MT319915** | **MT319821** | **MT311370** | **MT503345** | **MT349966** |
| *N. daptria* Means, Hennen, & Marek, 2020 | MPE04156 | 35.97775 | -82.8478 | Tennessee, Greene | **MT302681** | **-** | **MT319906** | **-** | **MT503457** | **MT350090** |
| *N. dilatata* (Hennen & Shelley, 2015) | MPE02788 | 35.59143 | -86.70294 | Tennessee, Marshall | **-** | **MN719676** | **MN817830** | **MN699762** | **MT503415** | **MT350043** |
| *N. domestica* Shelley, 1975 | MPE00305 | 36.1386 | -81.6694 | North Carolina, Watauga | **MN817799** | **MN719647** | **MN817832** | **MN699763** | **MT503357** | **MT349980** |
| *N. ericacea* Hoffman, 1949 | MPE00276 | 37.17295 | -80.40787 | Virginia, Montgomery | **MN817800** | **MN719646** | **MN817833** | **-** | **MT503356** | **MT349979** |
| *N. ericacea* Hoffman, 1949 | MPE01850 | 37.86851 | -79.87131 | Virginia, Alleghany | **MT302725** | **MT319938** | **-** | **MN699764** | **MT503384** | **MT350012** |
| *N. fowleri* Chamberlin, 1947 | MPE00433 | 38.69476 | -79.51469 | West Virginia, Pendleton | **MN817801** | **MN719648** | **MN817834** | **MN699765** | **MT503358** | **MT349981** |
| *N. fowleri* Chamberlin, 1947 | MPE03017 | 39.9079 | -77.47775 | Pennsylvania, Franklin | **MT302716** | **MT319962** | **MT319875** | **-** | **MT503427** | **MT350055** |
| *N. fowleri* Chamberlin, 1947 | MPE03071 | 41.10344 | -77.24355 | Pennsylvania, Clinton | **MT302717** | **MT319963** | **MT319876** | **MT311431** | **MT503428** | **MT350056** |
| *N. fracta* Means, Hennen, & Marek, 2020 | MPE03178 | 37.28973 | -82.29994 | Virginia, Dickenson | **MT302667** | **MT319969** | **MT319883** | **-** | **MT503434** | **MT350063** |
| *N. fracta* Means, Hennen, & Marek, 2020 | MPE03184 | 37.46902 | -82.54621 | Kentucky, Pike | **MT302677** | **MT319970** | **MT319884** | **-** | **MT503435** | **MT350064** |
| *N. fritzae* Means, Hennen, & Marek, 2020 | MPE02359 | 34.56287 | -85.06811 | Georgia, Floyd | **MT302690** | **MT319951** | **MT319861** | **MT311415** | **MT503405** | **MT350033** |
| *N. hardeni* Means, Hennen, & Marek, 2020 | MPE02278 | 36.5577 | -79.3515 | Virginia, Danville City | **MT302652** | **-** | **MT319859** | **MT311413** | **MT503402** | **MT350030** |
| *N. hippopotama* Means, Hennen, & Marek, 2020 | MPE04150 | 36.10593 | -82.65458 | Tennessee, Greene | **MT302680** | **-** | **MT319905** | **-** | **MT503456** | **MT350089** |
| *N. hokie* Means et al., 2020 | MPE00275 | 37.17295 | -80.40787 | Virginia, Montgomery | **MT302645** | **MT319924** | **MT319833** | **MT311381** | **-** | **MT349978** |
| *N. hokie* Means et al., 2020 | MPE00880 | 37.22507 | -80.42761 | Virginia, Montgomery | **MN817806** | **-** | **MN817840** | **MN699771** | **MT503362** | **MT349987** |
| *N. ignis* Means, Hennen, & Marek, 2020 | MPE00912 | 37.02572 | -81.09017 | Virginia, Wythe | **MT302707** | **-** | **MT319839** | **MT311386** | **MT503365** | **MT349990** |
| *N. ignis* Means, Hennen, & Marek, 2020 | MPE01063 | 37.03837 | -81.10909 | Virginia, Bland | **MT302706** | **-** | **MT319842** | **MT311390** | **MT503370** | **MT349995** |
| *N. ignis* Means, Hennen, & Marek, 2020 | MPE01198 | 37.04789 | -81.11555 | Virginia, Bland | **MT302688** | **-** | **-** | **MT311392** | **MT503372** | **MT349998** |
| *N. kassoni* Means, Hennen, & Marek, 2020 | MPE00544 | 36.30728 | -84.22605 | Tennessee, Campbell | **MT302703** | **MT319925** | **MT319834** | **MT311382** | **MT503359** | **MT349982** |
| *N. komela* Means, Hennen, & Marek, 2020 | MPE03523 | 36.60622 | -80.77205 | Virginia, Carroll | **MT302721** | **-** | **MT319892** | **MT311440** | **MT503442** | **MT350073** |
| *N. laminata* Hoffman, 1949 | MPE02124 | 37.42098 | -80.50938 | Virginia, Giles | **MT302701** | **-** | **-** | **MT311408** | **MT503396** | **MT350024** |
| *N. laminata* Hoffman, 1949 | MPE02392 | 37.91395 | -79.02071 | Virginia, Augusta | **MN817802** | **-** | **MN817835** | **MN699766** | **MT503406** | **MT350034** |
| *N. laminata* Hoffman, 1949 | MPE02528 | 37.47176 | -80.56202 | West Virginia, Monroe | **MT302700** | **-** | **MT319866** | **MT311420** | **MT503411** | **MT350039** |
| *N. mcelroyorum* Means, Hennen, & Marek, 2020 | MPE02240 | 38.18027 | -81.83841 | West Virginia, Boone | **MT302656** | **-** | **MT319857** | **MT311411** | **MT503400** | **MT350028** |
| *N. mcelroyorum* Means, Hennen, & Marek, 2020 | MPE03113 | 38.30473 | -82.35124 | West Virginia, Wayne | **MT302654** | **MT319965** | **MT319878** | **MT311433** | **MT503430** | **MT350058** |
| *N. mcelroyorum* Means, Hennen, & Marek, 2020 | MPE03125 | 38.34754 | -82.68667 | Kentucky, Boyd | **MT302655** | **MT319966** | **MT319879** | **MT311434** | **MT503431** | **MT350059** |
| *N. minor* Chamberlin, 1918 | MPE01249 | 36.26516 | -82.23001 | Tennessee, Carter | **MN817803** | **-** | **MN817836** | **MN699767** | **MT503377** | **MT350003** |
| *N. minor* Chamberlin, 1918 | MPE01313 | 36.26516 | -82.23001 | Tennessee, Carter | **MT302663** | **MT319933** | **MT319845** | **MT311395** | **MT503378** | **MT350004** |
| *N. minor* Chamberlin, 1918 | MPE04060 | 36.31997 | -82.08347 | Tennessee, Carter | **MT302665** | **MT319982** | **MT319899** | **MT311450** | **-** | **MT350082** |
| *N. minor* Chamberlin, 1918 | MPE04080 | 36.16749 | -82.0984 | Tennessee, Carter | **MT302664** | **-** | **MT319900** | **MT311451** | **MT503451** | **MT350083** |
| *N. minor* Chamberlin, 1918 | MPE04100 | 36.27708 | -82.34615 | Tennessee, Washington | **-** | **-** | **MT319901** | **MT311453** | **MT503453** | **MT350085** |
| *N. minor* Chamberlin, 1918 | MPE04103 | 36.17428 | -82.29823 | Tennessee, Unicoi | **MT302662** | **-** | **MT319902** | **MT311454** | **MT503454** | **MT350086** |
| *N. minor* Chamberlin, 1918 | MPE04107 | 36.13898 | -82.34686 | Tennessee, Unicoi | **MT302678** | **-** | **MT319903** | **MT311455** | **-** | **MT350087** |
| *N. minor* Chamberlin, 1918 | MPE04133 | 36.04829 | -82.56155 | Tennessee, Unicoi | **MT302679** | **MT319983** | **MT319904** | **MT311456** | **MT503455** | **MT350088** |
| *N. missouriensis* Chamberlin, 1928 | MPE02800 | 38.67069 | -90.7515 | Missouri, St. Charles | **MT302644** | **MT319958** | **-** | **MT311423** | **MT503418** | **MT350046** |
| *N. monsdomia* Means, Hennen, & Marek, 2020 | MPE02188 | 36.10326 | -83.76422 | Tennessee, Knox | **MT302691** | **-** | **MT319855** | **MT311409** | **MT503398** | **MT350026** |
| *N. morrisoni* Hoffman, 1948 | MPE02015 | 38.14746 | -78.74435 | Virginia, Albemarle | **MN817804** | **MN719665** | **MN817837** | **MN699768** | **MT503386** | **MT350014** |
| *N. morrisoni* Hoffman, 1948 | MPE02107 | 38.14746 | -78.74435 | Virginia, Albemarle | **MT302747** | **MT319943** | **MT319850** | **MT311404** | **MT503391** | **MT350019** |
| *N. morrisoni* Hoffman, 1948 | MPE02115 | 37.83889 | -79.02139 | Virginia, Nelson | **MT302745** | **MT319947** | **-** | **MT311406** | **MT503395** | **MT350023** |
| *N. morrisoni* Hoffman, 1948 | MPE02491 | 37.75472 | -79.185 | Virginia, Amherst | **MT302746** | **MT319954** | **MT319864** | **MT311418** | **MT503409** | **MT350037** |
| *N. morrisoni* Hoffman, 1948 | MPE02595 | 38.38023 | -78.50368 | Virginia, Greene | **MT302748** | **MT319955** | **MT319867** | **MT311421** | **MT503412** | **MT350040** |
| *N. morrisoni* Hoffman, 1948 | MPE02872 | 37.84252 | -79.11655 | Virginia, Nelson | **MT302744** | **MT319960** | **MT319870** | **MT311426** | **MT503421** | **MT350049** |
| *N. ohionis* Loomis & Hoffman, 1948 | MPE00906 | 39.2107 | -81.8421 | Ohio, Athens | **-** | **-** | **MT319838** | **-** | **MT503363** | **MT349988** |
| *N. ohionis* Loomis & Hoffman, 1948 | MPE00907 | 39.4307 | -81.5003 | Ohio, Washington | **MN817807** | **-** | **MN817841** | **-** | **MT503364** | **MT349989** |
| *N. ohionis* Loomis & Hoffman, 1948 | MPE03643 | 39.24205 | -81.29918 | West Virginia, Wood | **MT302712** | **MN719688** | **MT319894** | **MN699772** | **MT503444** | **MT350075** |
| *N. paupertata* Means, Hennen, & Marek, 2020 | MPE00108 | 37.26783 | -80.48522 | Virginia, Montgomery | **MT302708** | **MT319917** | **MT319823** | **MT311372** | **MT503346** | **MT349968** |
| *N. piccolia* Means, Hennen, & Marek, 2020 | MPE03809 | 37.80658 | -79.61522 | Virginia, Rockbridge | **MT302719** | **MT319980** | **MT319896** | **MT311445** | **MT503447** | **MT350077** |
| *N. scholastica* Means, Hennen, & Marek, 2020 | MPE03485 | 37.79563 | -79.44273 | Virginia, Rockingham | **MT302720** | **MT319974** | **-** | **MT311438** | **MT503440** | **MT350070** |
| *N. scutellaria* Causey, 1942 | MPE01474 | 35.63609 | -83.49378 | Tennessee, Sevier | **MN817808** | **-** | **MN817842** | **MN699773** | **MT503380** | **MT350006** |
| *N. scutellaria* Causey, 1942 | MPE02105 | 35.4938 | -83.15572 | North Carolina, Jackson | **-** | **MT319941** | **MT319848** | **MT311402** | **MT503389** | **MT350017** |
| *N. serpentiba* Means, Hennen, & Marek, 2020 | MPE00202 | 36.7761 | -80.54463 | Virginia, Carroll | **MT302649** | **MT319919** | **MT319827** | **MT311376** | **MT503350** | **MT349972** |
| *N. serpentiba* Means, Hennen, & Marek, 2020 | MPE00817 | 36.81324 | -80.34959 | Virginia, Floyd | **MT302650** | **-** | **MT319837** | **MT311385** | **MT503361** | **MT349986** |
| *N. serpentiba* Means, Hennen, & Marek, 2020 | MPE02610 | 36.7182 | -80.32461 | Virginia, Patrick | **MT302651** | **MT319956** | **MT319868** | **MT311422** | **MT503413** | **MT350041** |
| *N. sheari* Means, Hennen, & Marek, 2020 | MPE01684 | 37.46476 | -81.06235 | Virginia, Mercer | **-** | **MT319935** | **-** | **MT311397** | **-** | **MT350008** |
| *N. shenandoa* Hoffman, 1949 | MPE00231 | 39.43698 | -79.98525 | West Virginia, Marion | **MT302750** | **MT319920** | **MT319828** | **MT311377** | **MT503351** | **MT349973** |
| *N. shenandoa* Hoffman, 1949 | MPE03104 | 37.77483 | -83.68251 | Kentucky, Powell | **-** | **MT319964** | **MT319877** | **MT311432** | **MT503429** | **MT350057** |
| *N. solina* Means, Hennen, & Marek, 2020 | MMC0201 | 36.95333 | -82.05507 | Virginia, Russell | **MT302673** | **MT319914** | **MT319820** | **MT311369** | **MT503344** | **MT349965** |
| *N. solina* Means, Hennen, & Marek, 2020 | MPE00128 | 37.52641 | -80.98794 | West Virginia, Mercer | **MT302674** | **-** | **MT319824** | **MT311373** | **MT503347** | **MT349969** |
| *N. solina* Means, Hennen, & Marek, 2020 | MPE02428 | 36.9586 | -81.31893 | Virginia, Wythe | **MT302672** | **MT319953** | **MT319863** | **MT311417** | **MT503408** | **MT350036** |
| *N. spruilli* Means, Hennen, & Marek, 2020 | MMC0035 | 36.89499 | -82.59027 | Virginia, Wise | **MT302676** | **MT319913** | **MT319819** | **MT311367** | **MT503342** | **MT349963** |
| *N. stellapolis* Means, Hennen, & Marek, 2020 | MPE00252 | 37.23098 | -79.95028 | Virginia, Roanoke | **MT302646** | **-** | **MT319831** | **MT311380** | **MT503354** | **MT349976** |
| *N. stellaradix* Means, Hennen, & Marek, 2020 | MPE00239 | 36.96624 | -80.41797 | Virginia, Montgomery | **MT302647** | **MT319922** | **MT319830** | **MT311379** | **MT503353** | **MT349975** |
| *N. suprema* Means, Hennen, & Marek, 2020 | MPE00075 | 36.70672 | -81.60284 | Virginia, Smyth | **MT302714** | **MT319916** | **-** | **MT311371** | **-** | **MT349967** |
| *N*. *tasskelsoae* Means, Hennen, & Marek, 2020 | MPE00578 | 38.11011 | -80.27332 | West Virginia, Hillsboro | **MT302694** | **MT319926** | **MT319835** | **-** | **-** | **MT349983** |
| *N*. *tasskelsoae* Means, Hennen, & Marek, 2020 | MPE04023 | 38.2498 | -80.4455 | West Virginia, Greenbrier | **MT302696** | **-** | **-** | **MT311446** | **MT503448** | **MT350078** |
| *N*. *tasskelsoae* Means, Hennen, & Marek, 2020 | SPC000710 | 38.249 | -80.44375 | West Virginia, Greenbrier | **MT302695** | **MT319987** | **-** | **MT311460** | **MT503463** | **-** |
| *N. tennesseensis* (Bollman, 1889) | MPE01237 | 36.1515 | -83.51701 | Tennessee, Jefferson | **MN817809** | **MN719652** | **MT319822** | **MN699774** | **MT503376** | **MT350002** |
| *N. tenua* Means, Hennen, & Marek, 2020 | MPE00925 | 37.14073 | -81.13944 | Virginia, Bland | **-** | **-** | **MT319840** | **MT311387** | **MT503366** | **MT349991** |
| *N. tenua* Means, Hennen, & Marek, 2020 | MPE01111 | 37.12343 | -81.13559 | Virginia, Bland | **MT302686** | **-** | **MT319843** | **MT311391** | **-** | **MT349996** |
| *N. terricola* (Williams & Hefner, 1928) | MPE01691 | 39.49131 | -82.57975 | Ohio, Hocking | **MT302669** | **MT319937** | **-** | **-** | **-** | **MT350010** |
| *N. terricola* (Williams & Hefner, 1928) | MPE01738 | 38.72137 | -83.43355 | Ohio, Adams | **MN817810** | **MN719664** | **MN817843** | **MN699775** | **MT503383** | **MT350011** |
| *N. tsuga* Means, Hennen, & Marek, 2020 | MPE04047 | 36.57037 | -82.23569 | Tennessee, Sullivan | **MT302666** | **MT319981** | **MT319897** | **MT311448** | **MT503449** | **MT350080** |
| *N. wilsoni* Hoffman, 1949 | MPE01149 | 37.38917 | -80.50528 | Virginia, Giles | **MT302727** | **MT319930** | **-** | **-** | **MT503371** | **MT349997** |
| *N. wilsoni* Hoffman, 1949 | MPE02132 | 37.38927 | -80.50575 | Virginia, Giles | **MN817811** | **MN719667** | **MN817844** | **MN699776** | **MT503397** | **MT350025** |
| *Oenomaea pulchella* (Bollman, 1889) | MPE02350 | 36.25194 | -83.08472 | Tennessee, Hawkins | **-** | **MN719668** | **MN817846** | **-** | **-** | **-** |
| *Oenomaea pulchella* (Bollman, 1889) | MPE02353 | 36.25194 | -83.08472 | Tennessee, Hawkins | **-** | **-** | **-** | **-** | **MT503404** | **MT350032** |
