# Supplementary figures and images for: A revision of the minor species group in the millipede genus Nannaria Chamberlin, 1918 (Diplopoda, Polydesmida, Xystodesmidae)
Source: Zookeys. 2021 Apr 13;1030:1–180. doi: 10.3897/zookeys.1030.62544 (PMC8060247; doi:10.3897/zookeys.1030.62544)

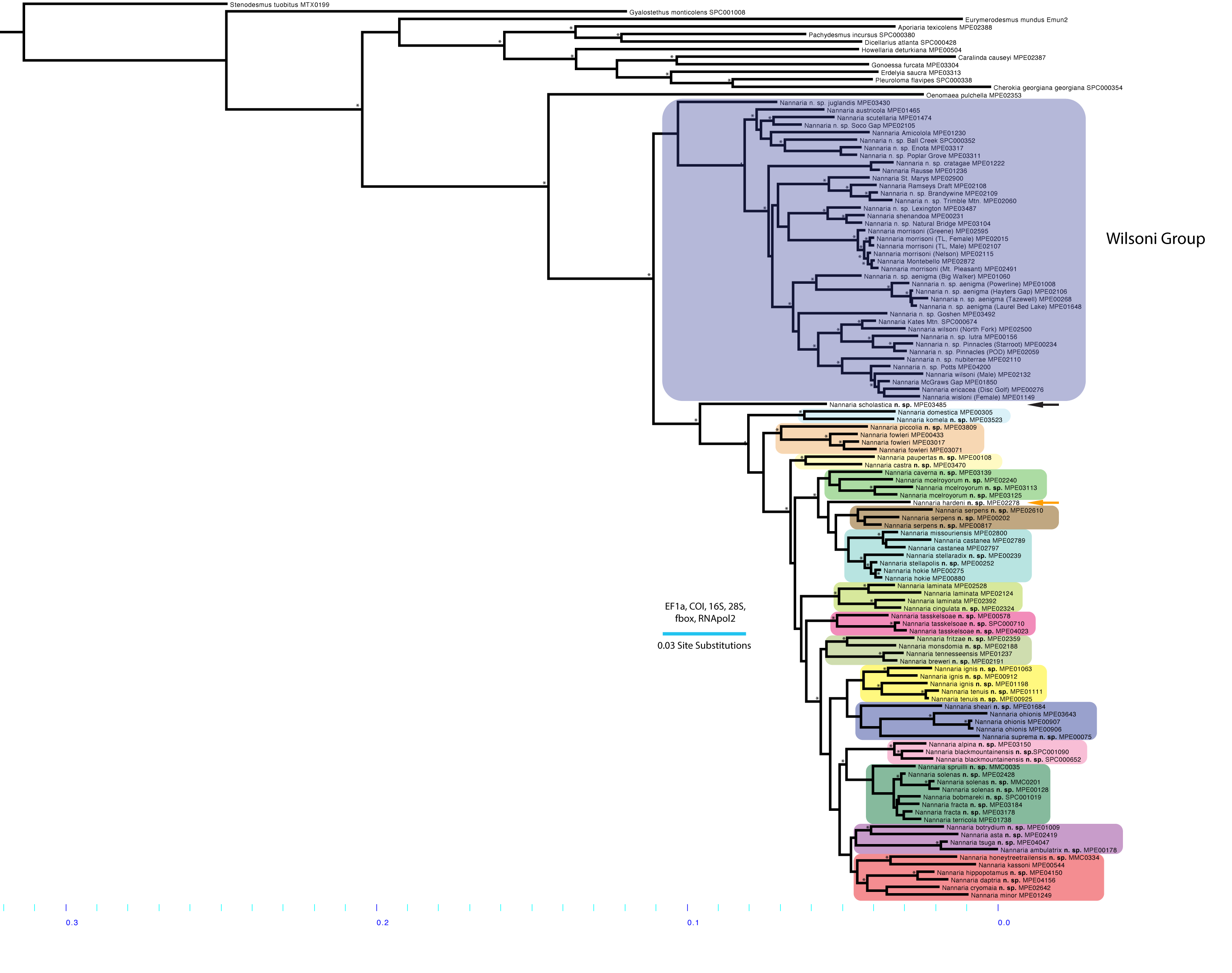

Supplement: Supplementary material 3 — Suppl. material 3 [file zookeys-1030-001-s003.tif]

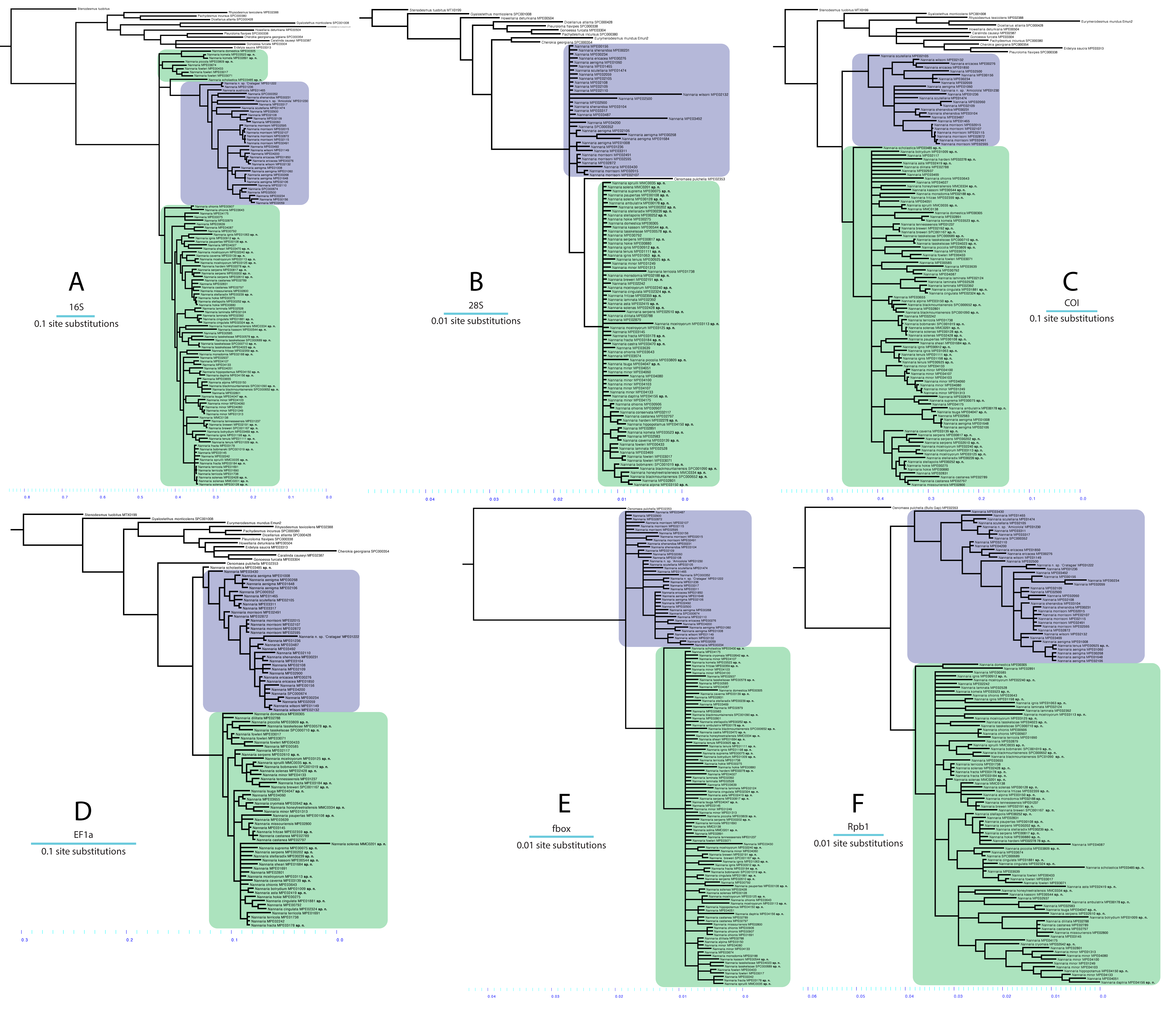

Supplement: Supplementary material 4 — Suppl. material 4 [file zookeys-1030-001-s004.tif]
