# Supplementary material for: A revision of the minor species group in the millipede genus Nannaria Chamberlin, 1918 (Diplopoda, Polydesmida, Xystodesmidae)
Source: Zookeys. 2021 Apr 13;1030:1–180. doi: 10.3897/zookeys.1030.62544 (PMC8060247; doi:10.3897/zookeys.1030.62544)
Supplement: Supplementary material 5 — Suppl. material 5 [file zookeys-1030-001-s005.docx]

| **Supplementary Material 5.1** Morphological scoring matrix for *Nannaria minor* species group, characters 1-12. See Supplementary Material 6 for character key. | | | | | | | | | | | | |
| --- | --- | --- | --- | --- | --- | --- | --- | --- | --- | --- | --- | --- |
| **Species** | **C 1** | **C 2** | **C 3** | **C 4** | **C 5** | **C 6** | **C 7** | **C 8** | **C 9** | **C 10** | **C 11** | **C 12** |
| *alpina* | 2 | 1 | 1 | 1 | 1 | 1 | 5 | 3 | 1 | 2 | 2 | 3 |
| *ambulatrix* | 1 | 2 | 2 | 3 | 2 | 3 | 4 | 2 | 1 | 3 | 1 | 1 |
| *astavalla* | 2 | 2 | 2 | 2 | 2 | 3 | 3 | 1 | 1 | 2 | 2 | 2 |
| *blackmountainensis* | 2 | 2 | 3 | 3 | 4 | 1 | 1 | 1 | 1 | 2 | 1 | 1 |
| *bobmareki* | 2 | 2 | 2 | 3 | 4 | 1 | 1 | 1 | 1 | 2 | 2 | 1 |
| *botrydia* | 2 | 2 | 3 | 3 | 3 | 1 | 4 | 2 | 1 | 3 | 1 | 1 |
| *breweri* | 2 | 2 | 3 | 3 | 4 | 1 | 5 | 2 | 1 | 4 | 1 | 2 |
| *castanea* | 2 | 2 | 2 | 2 | 2 | 2 | 1 | 2 | 1 | 3 | 1 | 1 |
| *castra* | 2 | 1 | 1 | 1 | 1 | 1 | 2 | 2 | 1 | 4 | 2 | 3 |
| *caverna* | 2 | 2 | 2 | 2 | 2 | 3 | 4 | 3 | 1 | 1 | 1 | 1 |
| *cingulata* | 2 | 2 | 2 | 3 | 2 | 1 | 4 | 3 | 1 | 1 | 2 | 1 |
| *conservata* | 2 | 2 | 2 | 2 | 2 | 1 | 1 | 2 | 1 | 3 | 2 | 3 |
| *cryomaia* | 2 | 2 | 2 | 2 | 2 | 2 | 4 | 2 | 1 | 1 | 1 | 1 |
| *daptria* | 2 | 2 | 2 | 2 | 2 | 1 | 5 | 2 | 1 | 2 | 1 | 1 |
| *davidcauseyi* | 2 | 2 | 2 | 3 | 2 | 1 | 1 | 1 | 1 | 2 | 1 | 1 |
| *dilitata* | 2 | 2 | 2 | 3 | 5 | 1 | 5 | 2 | 1 | 3 | 1 | 3 |
| *domestica* | 2 | 2 | 2 | 3 | 4 | 1 | 2 | 3 | 3 | 1 | 1 | 2 |
| *equalis* | 1 | 2 | 2 | 2 | 2 | 1 | 2 | 1 | 2 | 2 | 1 | 1 |
| *fowleri* | 2 | 2 | 2 | 3 | 2 | 3 | 4 | 2 | 1 | 1 | 1 | 1 |
| *fracta* | 1 | 2 | 2 | 2 | 2 | 1 | 1 | 1 | 1 | 2 | 2 | 1 |
| *fritzae* | 2 | 2 | 2 | 3 | 2 | 1 | 1 | 2 | 1 | 3 | 1 | 1 |
| *hardeni* | 2 | 2 | 2 | 2 | 2 | 1 | 1 | 1 | 2 | 2 | 1 | 1 |
| *hippopotama* | 1 | 2 | 2 | 3 | 2 | 1 | 1 | 1 | 1 | 2 | 2 | 1 |
| *hokie* | 2 | 2 | 2 | 2 | 2 | 2 | 1 | 2 | 1 | 3 | 1 | 1 |
| *honeytreetrailensis* | 2 | 2 | 2 | 2 | 3 | 3 | 3 | 1 | 1 | 1 | 1 | 3 |
| *ignis* | 2 | 2 | 2 | 2 | 2 | 1 | 4 | 3 | 1 | 2 | 1 | 3 |
| *kassoni* | 2 | 2 | 2 | 2 | 2 | 3 | 5 | 2 | 1 | 3 | 1 | 2 |
| *komela* | 2 | 2 | 3 | 3 | 4 | 1 | 4 | 3 | 1 | 1 | 1 | 3 |
| *laminata* | 2 | 2 | 2 | 3 | 2 | 1 | 2 | 1 | 1 | 2 | 2 | 2 |
| *mcelroyorum* | 2 | 2 | 2 | 2 | 2 | 3 | 4 | 1 | 1 | 1 | 1 | 2 |
| *minor* | 2 | 2 | 2 | 3 | 2 | 1 | 1 | 3 | 2 | 2 | 1 | 1 |
| *missouriensis* | 2 | 1 | 2 | 2 | 2 | 5 | 6 | 4 | 1 | 5 | 1 | 1 |
| *monsdomia* | 1 | 2 | 2 | 2 | 3 | 2 | 2 | 2 | 2 | 2 | 1 | 1 |
| *oblonga* | 2 | 1 | 1 | 1 | 1 | 1 | 5 | 1 | 1 | 2 | 1 | 1 |
| *ohionis* | 2 | 2 | 2 | 2 | 2 | 2 | 1 | 1 | 1 | 2 | 1 | 3 |
| *paupertata* | 2 | 2 | 2 | 2 | 2 | 3 | 4 | 2 | 1 | 1 | 1 | 1 |
| *piccola* | 2 | 2 | 2 | 3 | 2 | 3 | 3 | 3 | 1 | 2 | 1 | 3 |
| *rhysodesmoides* | 2 | 1 | 1 | 1 | 1 | 1 | 2 | 1 | 1 | 2 | 1 | 1 |
| *rutherfordensis* | 2 | 2 | 2 | 2 | 2 | 3 | 4 | 2 | 1 | 2 | 2 | 1 |
| *scholastica* | 2 | 1 | 1 | 1 | 1 | 1 | 1 | 3 | 1 | 2 | 1 | 1 |
| *serpentiba* | 2 | 2 | 2 | 2 | 2 | 3 | 4 | 3 | 1 | 2 | 1 | 2 |
| *sheari* | 2 | 2 | 3 | 3 | 4 | 1 | 2 | 2 | 1 | 4 | 2 | 3 |
| *sigmoidea* | 2 | 2 | 2 | 2 | 3 | 3 | 4 | 2 | 1 | 1 | 1 | 1 |
| *simplex* | 2 | 2 | 2 | 3 | 2 | 1 | 4 | 1 | 1 | 2 | 1 | 1 |
| *solina* | 2 | 2 | 3 | 3 | 4 | 1 | 1 | 1 | 1 | 2 | 1 | 1 |
| *spruilli* | 1 | 2 | 2 | 3 | 2 | 1 | 1 | 1 | 1 | 2 | 1 | 1 |
| *stellapolis* | 2 | 2 | 2 | 2 | 4 | 2 | 5 | 2 | 1 | 3 | 2 | 1 |
| *stellaradix* | 2 | 2 | 2 | 2 | 2 | 3 | 5 | 2 | 1 | 1 | 1 | 1 |
| *suprema* | 2 | 2 | 2 | 3 | 2 | 1 | 4 | 1 | 1 | 1 | 2 | 2 |
| *tasskelsoae* | 2 | 2 | 2 | 3 | 2 | 3 | 2 | 2 | 1 | 2 | 1 | 1 |
| *tennesseensis* | 2 | 2 | 3 | 3 | 4 | 1 | 1 | 1 | 1 | 2 | 1 | 1 |
| *tenua* | 2 | 2 | 3 | 3 | 5 | 1 | 4 | 2 | 1 | 4 | 1 | 3 |
| *terricola* | 1 | 2 | 3 | 3 | 4 | 1 | 1 | 1 | 1 | 2 | 2 | 1 |
| *tsuga* | 2 | 2 | 2 | 2 | 3 | 1 | 2 | 1 | 2 | 2 | 1 | 1 |

| **Supplementary Material 5.2** Morphological scoring matrix for *Nannaria* minor species group, characters 13-24. See Supplementary Material 6 for character key. | | | | | | | | | | | | |
| --- | --- | --- | --- | --- | --- | --- | --- | --- | --- | --- | --- | --- |
| **Species** | **C 13** | **C 14** | **C 15** | **C 16** | **C 17** | **C 18** | **C 19** | **C 20** | **C 21** | **C 22** | **C 23** | **C 24** |
| *alpina* | 2 | 2 | 1 | 2 | 1 | 1 | 2 | 1 | 2 | 2 | 2 | 3 |
| *ambulatrix* | 3 | 3 | 1 | 2 | 1 | 1 | 2 | 1 | 2 | 2 | 2 | 3 |
| *astavalla* | 1 | 3 | 1 | 1 | 1 | 1 | 3 | 2 | 2 | 2 | ? | ? |
| *blackmountainensis* | 2 | 3 | 1 | 1 | 1 | 1 | 4 | 1 | 2 | 2 | 2 | 1 |
| *bobmareki* | 2 | 2 | 1 | 2 | 1 | 2 | 1 | 1 | 2 | 2 | 2 | 3 |
| *botrydia* | 1 | 3 | 1 | 2 | 1 | 1 | 3 | 2 | 2 | 2 | 2 | 2 |
| *breweri* | 1 | 3 | 1 | 2 | 1 | 1 | 3 | 1 | 2 | 2 | 2 | 4 |
| *castanea* | 2 | 3 | 1 | 1 | 1 | 4 | 5 | 2 | 3 | 1 | 2 | 2 |
| *castra* | 2 | 5 | 1 | 1 | 1 | 1 | 5 | 2 | 2 | 2 | 2 | 2 |
| *caverna* | 3 | 2 | 1 | 2 | 1 | 1 | 1 | 1 | 2 | 2 | 2 | 2 |
| *cingulata* | 1 | 2 | 2 | 1 | 1 | 1 | 3 | 1 | 2 | 2 | 2 | 2 |
| *conservata* | 1 | 3 | 1 | 2 | 1 | 1 | 1 | 1 | 2 | 2 | 2 | 3 |
| *cryomaia* | 1 | 3 | 1 | 2 | 1 | 1 | 1 | 1 | 2 | 2 | 2 | 2 |
| *daptria* | 2 | 3 | 1 | 1 | 1 | 2 | 2 | 1 | 2 | 2 | ? | ? |
| *davidcauseyi* | 2 | 3 | 1 | 2 | 4 | 4 | 5 | 1 | 2 | 1 | ? | ? |
| *dilitata* | 1 | 3 | 1 | 2 | 3 | 1 | 2 | 1 | 2 | 2 | 2 | 2 |
| *domestica* | 1 | 3 | 1 | 2 | 1 | 1 | 2 | 5 | 2 | 1 | 2 | 3 |
| *equalis* | 2 | 2 | 1 | 1 | 1 | 1 | 3 | 1 | 2 | 2 | ? | ? |
| *fowleri* | 1 | 3 | 1 | 1 | 1 | 1 | 1 | 1 | 2 | 2 | 2 | 2 |
| *fracta* | 3 | 1 | 1 | 2 | 1 | 2 | 1 | 4 | 2 | 2 | 2 | 2 |
| *fritzae* | 2 | 3 | 1 | 2 | 4 | 1 | 1 | 1 | 2 | 2 | ? | ? |
| *hardeni* | 3 | 3 | 1 | 2 | 1 | 1 | 1 | 1 | 2 | 2 | 2 | 2 |
| *hippopotama* | 2 | 2 | 1 | 1 | 1 | 1 | 3 | 1 | 2 | 2 | 2 | 2 |
| *hokie* | 1 | 3 | 1 | 2 | 4 | 4 | 5 | 2 | 2 | 2 | 2 | 3 |
| *honeytreetrailensis* | 1 | 3 | 1 | 1 | 1 | 1 | 2 | 1 | 2 | 2 | ? | ? |
| *ignis* | 1 | 3 | 1 | 2 | 1 | 1 | 3 | 1 | 2 | 2 | ? | ? |
| *kassoni* | 1 | 3 | 1 | 2 | 3 | 1 | 2 | 1 | 2 | 1 | ? | ? |
| *komela* | 1 | 3 | 1 | 2 | 1 | 1 | 1 | 2 | 2 | 2 | 2 | 3 |
| *laminata* | 1 | 3 | 1 | 1 | 1 | 1 | 1 | 2 | 2 | 1 | ? | ? |
| *mcelroyorum* | 2 | 3 | 1 | 2 | 2 | 1 | 1 | 1 | 2 | 2 | 2 | 3 |
| *minor* | 1 | 3 | 1 | 2 | 4 | 1 | 1 | 1 | 2 | 2 | 2 | 2 |
| *missouriensis* | 1 | 3 | 1 | 2 | 4 | 4 | 5 | 2 | 3 | 1 | 2 | 3 |
| *monsdomia* | 3 | 2 | 1 | 2 | 1 | 1 | 3 | 1 | 2 | 2 | 2 | 2 |
| *oblonga* | 1 | 3 | 1 | 1 | 1 | 1 | 1 | 2 | 2 | 1 | ? | ? |
| *ohionis* | 1 | 3 | 1 | 2 | 1 | 1 | 1 | 1 | 2 | 1 | 2 | 3 |
| *paupertata* | 1 | 3 | 1 | 1 | 3 | 1 | 1 | 5 | 2 | 2 | 2 | 2 |
| *piccola* | 1 | 3 | 1 | 2 | 1 | 1 | 1 | 1 | 2 | 2 | 2 | 2 |
| *rhysodesmoides* | 2 | 3 | 1 | 2 | 1 | 1 | 2 | 5 | 2 | 1 | 1 | ? |
| *rutherfordensis* | 1 | 3 | 1 | 1 | 1 | 1 | 1 | 1 | 2 | 2 | ? | ? |
| *scholastica* | 1 | 3 | 1 | 2 | 1 | 4 | 1 | 5 | 2 | 1 | 2 | 2 |
| *serpentiba* | 1 | 3 | 1 | 1 | 2 | 1 | 1 | 1 | 2 | 2 | 2 | 3 |
| *sheari* | 1 | 3 | 1 | 2 | 1 | 1 | 5 | 2 | 2 | 2 | 2 | 2 |
| *sigmoidea* | 3 | 3 | 1 | 2 | 1 | 1 | 3 | 2 | 2 | 2 | ? | ? |
| *simplex* | 1 | 3 | 1 | 1 | 1 | 1 | 1 | 1 | 2 | 2 | ? | ? |
| *solina* | 2 | 2 | 1 | 2 | 1 | 1 | 1 | 4 | 2 | 2 | 2 | 3 |
| *spruilli* | 3 | 2 | 1 | 2 | 1 | 1 | 3 | 1 | 2 | 2 | 2 | 2 |
| *stellapolis* | 2 | 3 | 1 | 2 | 1 | 1 | 2 | 2 | 2 | 2 | ? | ? |
| *stellaradix* | 1 | 3 | 1 | 2 | 3 | 1 | 2 | 1 | 2 | 2 | 2 | 2 |
| *suprema* | 3 | 3 | 1 | 2 | 1 | 1 | 2 | 2 | 2 | 2 | 2 | 2 |
| *tasskelsoae* | 1 | 3 | 1 | 2 | 3 | 3 | 3 | 1 | 2 | 2 | 2 | 2 |
| *tennesseensis* | 3 | 4 | 1 | 2 | 1 | 1 | 2 | 4 | 2 | 2 | 2 | 2 |
| *tenua* | 1 | 3 | 1 | 1 | 1 | 1 | 3 | 2 | 2 | 2 | 2 | 3 |
| *terricola* | 2 | 2 | 1 | 2 | 1 | 1 | 1 | 5 | 2 | 2 | 2 | 2 |
| *tsuga* | 1 | 3 | 1 | 2 | 1 | 1 | 3 | 1 | 2 | 2 | 2 | 3 |

| **Supplementary Material 5.3** Morphological scoring matrix for *Nannaria* minor species group, characters 25-36. See Supplementary Material 6 for character key. | | | | | | | | | | | | |
| --- | --- | --- | --- | --- | --- | --- | --- | --- | --- | --- | --- | --- |
| **Species** | **C 25** | **C 26** | **C 27** | **C 28** | **C 29** | **C 30** | **C 31** | **C 32** | **C 33** | **C 34** | **C 35** | **C 36** |
| *alpina* | 1 | 2 | 3 | 2 | 1 | 2 | 2 | 2 | 2 | 1 | 1 | 1 |
| *ambulatrix* | 1 | 1 | 3 | 2 | 1 | 2 | 2 | 2 | 2 | 1 | 1 | 1 |
| *astavalla* | ? | ? | ? | 2 | 1 | 2 | 2 | 2 | 2 | 1 | 1 | 1 |
| *blackmountainensis* | 1 | 3 | 3 | 2 | 1 | 2 | 2 | 2 | 2 | 1 | 1 | 1 |
| *bobmareki* | 1 | 5 | 3 | 2 | 1 | 2 | 2 | 2 | 2 | 1 | 1 | 1 |
| *botrydia* | 1 | 2 | 3 | 2 | 1 | 2 | 2 | 2 | 2 | 1 | 1 | 1 |
| *breweri* | 1 | 5 | 3 | 2 | 1 | 2 | 2 | 2 | 2 | 1 | 1 | 1 |
| *castanea* | 1 | 2 | 3 | 2 | 1 | 2 | 2 | 2 | 2 | 1 | 1 | 1 |
| *castra* | 1 | 5 | 3 | 2 | 1 | 2 | 2 | 2 | 2 | 1 | 1 | 1 |
| *caverna* | 1 | 5 | 3 | 2 | 1 | 2 | 2 | 2 | 2 | 1 | 1 | 1 |
| *cingulata* | 1 | 5 | 3 | 2 | 1 | 2 | 2 | 2 | 2 | 1 | 1 | 1 |
| *conservata* | 1 | 2 | 3 | 2 | 1 | 2 | 2 | 2 | 2 | 1 | 1 | 1 |
| *cryomaia* | 1 | 1 | 3 | 2 | 1 | 2 | 2 | 2 | 2 | 1 | 1 | 1 |
| *daptria* | ? | ? | ? | 2 | 1 | 2 | 2 | 2 | 2 | 1 | 1 | 1 |
| *davidcauseyi* | ? | ? | ? | 2 | 1 | 2 | 2 | 2 | 2 | 1 | 1 | 1 |
| *dilitata* | 1 | 3 | 3 | 2 | 1 | 2 | 2 | 2 | 2 | 1 | 1 | 1 |
| *domestica* | 1 | 3 | 3 | 2 | 1 | 2 | 2 | 2 | 2 | 1 | 1 | 1 |
| *equalis* | ? | ? | ? | 2 | 1 | 2 | 2 | 2 | 2 | 1 | 1 | 1 |
| *fowleri* | 1 | 1 | 3 | 2 | 1 | 2 | 2 | 2 | 2 | 1 | 1 | 1 |
| *fracta* | 1 | 3 | 3 | 2 | 1 | 2 | 2 | 2 | 2 | 1 | 1 | 1 |
| *fritzae* | ? | ? | ? | 2 | 1 | 2 | 2 | 2 | 2 | 1 | 1 | 1 |
| *hardeni* | 1 | 1 | 3 | 2 | 1 | 2 | 2 | 2 | 2 | 1 | 1 | 1 |
| *hippopotama* | 1 | 5 | 3 | 2 | 1 | 2 | 2 | 2 | 2 | 1 | 1 | 1 |
| *hokie* | 1 | 2 | 3 | 2 | 1 | 2 | 2 | 2 | 2 | 1 | 1 | 1 |
| *honeytreetrailensis* | ? | ? | ? | 2 | 1 | 2 | 2 | 2 | 2 | 1 | 1 | 1 |
| *ignis* | ? | ? | ? | 2 | 1 | 2 | 2 | 2 | 2 | 1 | 1 | 1 |
| *kassoni* | ? | ? | ? | 2 | 1 | 2 | 2 | 2 | 2 | 1 | 1 | 1 |
| *komela* | 1 | 5 | 3 | 2 | 1 | 2 | 2 | 2 | 2 | 1 | 1 | 1 |
| *laminata* | ? | ? | ? | 2 | 1 | 2 | 2 | 2 | 2 | 1 | 1 | 1 |
| *mcelroyorum* | 1 | 3 | 3 | 2 | 1 | 2 | 2 | 2 | 2 | 1 | 1 | 1 |
| *minor* | 1 | 1 | 3 | 2 | 1 | 2 | 2 | 2 | 2 | 1 | 1 | 1 |
| *missouriensis* | 1 | 1 | 3 | 2 | 1 | 2 | 2 | 2 | 2 | 1 | 1 | 1 |
| *monsdomia* | 1 | 3 | 3 | 2 | 1 | 2 | 2 | 2 | 2 | 1 | 1 | 1 |
| *oblonga* | ? | ? | ? | 2 | 1 | 2 | 2 | 2 | 2 | 1 | 1 | 1 |
| *ohionis* | 1 | 1 | 3 | 2 | 1 | 2 | 2 | 2 | 2 | 1 | 1 | 1 |
| *paupertata* | 1 | 1 | 3 | 2 | 1 | 2 | 2 | 2 | 2 | 1 | 1 | 1 |
| *piccola* | 1 | 1 | 3 | 2 | 1 | 2 | 2 | 2 | 2 | 1 | 1 | 1 |
| *rhysodesmoides* | ? | ? | ? | 2 | 1 | 2 | 2 | 2 | 2 | 1 | 1 | 1 |
| *rutherfordensis* | ? | ? | ? | 2 | 1 | 2 | 2 | 2 | 2 | 1 | 1 | 1 |
| *scholastica* | 1 | 1 | 3 | 2 | 1 | 2 | 2 | 2 | 2 | 1 | 1 | 1 |
| *serpentiba* | 1 | 3 | 3 | 2 | 1 | 2 | 2 | 2 | 2 | 1 | 1 | 1 |
| *sheari* | 1 | 1 | 3 | 2 | 1 | 2 | 2 | 2 | 2 | 1 | 1 | 1 |
| *sigmoidea* | ? | ? | ? | 2 | 1 | 2 | 2 | 2 | 2 | 1 | 1 | 1 |
| *simplex* | ? | ? | ? | 2 | 1 | 2 | 2 | 2 | 2 | 1 | 1 | 1 |
| *solina* | 1 | 5 | 3 | 2 | 1 | 2 | 2 | 2 | 2 | 1 | 1 | 1 |
| *spruilli* | 1 | 5 | 3 | 2 | 1 | 2 | 2 | 2 | 2 | 1 | 1 | 1 |
| *stellapolis* | ? | ? | ? | 2 | 1 | 2 | 2 | 2 | 2 | 1 | 1 | 1 |
| *stellaradix* | 1 | 1 | 3 | 2 | 1 | 2 | 2 | 2 | 2 | 1 | 1 | 1 |
| *suprema* | 1 | 1 | 3 | 2 | 1 | 2 | 2 | 2 | 2 | 1 | 1 | 1 |
| *tasskelsoae* | 1 | 5 | 3 | 2 | 1 | 2 | 2 | 2 | 2 | 1 | 1 | 1 |
| *tennesseensis* | 1 | 1 | 3 | 2 | 1 | 2 | 1 | 2 | 2 | 1 | 1 | 1 |
| *tenua* | 1 | 5 | 3 | 2 | 1 | 2 | 2 | 2 | 2 | 1 | 1 | 1 |
| *terricola* | 1 | 2 | 3 | 2 | 1 | 2 | 1 | 2 | 2 | 1 | 2 | 1 |
| *tsuga* | 1 | 1 | 3 | 2 | 1 | 2 | 2 | 2 | 2 | 1 | 1 | 1 |

| **Supplementary Material 5.4** Morphological scoring matrix for *Nannaria* minor species group, characters 37-47. See Supplementary Material 6 for character key. | | | | | | | | | | | |
| --- | --- | --- | --- | --- | --- | --- | --- | --- | --- | --- | --- |
| **Species** | **C 37** | **C 38** | **C 39** | **C 40** | **C 41** | **C 42** | **C 43** | **C 44** | **C 45** | **C 46** | **C 47** |
| *alpina* | 2 | 1 | 1 | 1 | 1 | 2 | 1 | 2 | 1 | 2 | 1 |
| *ambulatrix* | 2 | 1 | 1 | 1 | 1 | 2 | 1 | 2 | 1 | 2 | 1 |
| *astavalla* | 2 | 1 | 1 | 1 | 1 | 2 | 1 | 2 | 1 | 2 | 1 |
| *blackmountainensis* | 2 | 1 | 1 | 1 | 1 | 2 | 1 | 2 | 1 | 2 | 1 |
| *bobmareki* | 2 | 1 | 1 | 1 | 1 | 2 | 1 | 2 | 1 | 2 | 1 |
| *botrydia* | 2 | 1 | 1 | 1 | 1 | 2 | 1 | 2 | 1 | 2 | 1 |
| *breweri* | 2 | 1 | 1 | 1 | 1 | 2 | 1 | 2 | 1 | 2 | 1 |
| *castanea* | 2 | 1 | 1 | 1 | 1 | 2 | 1 | 2 | 1 | 2 | 1 |
| *castra* | 2 | 1 | 1 | 1 | 1 | 2 | 1 | 2 | 1 | 2 | 1 |
| *caverna* | 2 | 1 | 1 | 1 | 1 | 2 | 1 | 2 | 1 | 2 | 1 |
| *cingulata* | 2 | 1 | 1 | 1 | 1 | 2 | 1 | 2 | 1 | 2 | 1 |
| *conservata* | 2 | 1 | 1 | 1 | 1 | 2 | 1 | 2 | 1 | 2 | 1 |
| *cryomaia* | 2 | 1 | 1 | 1 | 1 | 2 | 1 | 2 | 1 | 2 | 1 |
| *daptria* | 2 | 1 | 1 | 1 | 1 | 2 | 1 | 2 | 1 | 2 | 1 |
| *davidcauseyi* | 2 | 1 | 1 | 1 | 1 | 2 | 1 | 2 | 1 | 2 | 1 |
| *dilitata* | 2 | 1 | 1 | 1 | 1 | 2 | 1 | 2 | 1 | 2 | 1 |
| *domestica* | 2 | 1 | 1 | 1 | 1 | 2 | 1 | 2 | 1 | 2 | 1 |
| *equalis* | 2 | 1 | 1 | 1 | 1 | 2 | 1 | 2 | 1 | 2 | 1 |
| *fowleri* | 2 | 1 | 1 | 1 | 1 | 2 | 1 | 2 | 1 | 2 | 1 |
| *fracta* | 2 | 1 | 1 | 1 | 1 | 2 | 1 | 2 | 1 | 2 | 1 |
| *fritzae* | 2 | 1 | 1 | 1 | 1 | 2 | 1 | 2 | 1 | 2 | 1 |
| *hardeni* | 2 | 1 | 1 | 1 | 1 | 2 | 1 | 2 | 1 | 2 | 1 |
| *hippopotama* | 2 | 1 | 1 | 1 | 1 | 2 | 1 | 2 | 1 | 2 | 1 |
| *hokie* | 2 | 1 | 1 | 1 | 1 | 2 | 1 | 2 | 1 | 2 | 1 |
| *honeytreetrailensis* | 2 | 1 | 1 | 1 | 1 | 2 | 1 | 2 | 1 | 2 | 1 |
| *ignis* | 2 | 1 | 1 | 1 | 1 | 2 | 1 | 2 | 1 | 2 | 1 |
| *kassoni* | 2 | 1 | 1 | 1 | 1 | 2 | 1 | 2 | 1 | 2 | 1 |
| *komela* | 2 | 1 | 1 | 1 | 1 | 2 | 1 | 2 | 1 | 2 | 1 |
| *laminata* | 2 | 1 | 1 | 1 | 1 | 2 | 1 | 2 | 1 | 2 | 1 |
| *mcelroyorum* | 2 | 1 | 1 | 1 | 1 | 2 | 1 | 2 | 1 | 2 | 1 |
| *minor* | 2 | 1 | 1 | 1 | 1 | 2 | 1 | 2 | 1 | 2 | 1 |
| *missouriensis* | 2 | 1 | 1 | 1 | 1 | 2 | 1 | 2 | 1 | 2 | 1 |
| *monsdomia* | 2 | 1 | 1 | 1 | 1 | 2 | 1 | 2 | 1 | 2 | 1 |
| *oblonga* | 2 | 1 | 1 | 1 | 1 | 2 | 1 | 2 | 1 | 2 | 1 |
| *ohionis* | 2 | 1 | 1 | 1 | 1 | 2 | 1 | 2 | 1 | 2 | 1 |
| *paupertata* | 2 | 1 | 1 | 1 | 1 | 2 | 1 | 2 | 1 | 2 | 1 |
| *piccola* | 2 | 1 | 1 | 1 | 1 | 2 | 1 | 2 | 1 | 2 | 1 |
| *rhysodesmoides* | 2 | 1 | 1 | 1 | 1 | 2 | 1 | 2 | 1 | 2 | 1 |
| *rutherfordensis* | 2 | 1 | 1 | 1 | 1 | 2 | 1 | 2 | 1 | 2 | 1 |
| *scholastica* | 2 | 1 | 1 | 1 | 1 | 2 | 1 | 2 | 1 | 2 | 1 |
| *serpentiba* | 2 | 1 | 1 | 1 | 1 | 2 | 1 | 2 | 1 | 2 | 1 |
| *sheari* | 2 | 1 | 1 | 1 | 1 | 2 | 1 | 2 | 1 | 2 | 1 |
| *sigmoidea* | 2 | 1 | 1 | 1 | 1 | 2 | 1 | 2 | 1 | 2 | 1 |
| *simplex* | 2 | 1 | 1 | 1 | 1 | 2 | 1 | 2 | 1 | 2 | 1 |
| *solina* | 2 | 1 | 1 | 1 | 1 | 2 | 1 | 2 | 1 | 2 | 1 |
| *spruilli* | 2 | 1 | 1 | 1 | 1 | 2 | 1 | 2 | 1 | 2 | 1 |
| *stellapolis* | 2 | 1 | 1 | 1 | 1 | 2 | 1 | 2 | 1 | 2 | 1 |
| *stellaradix* | 2 | 1 | 1 | 1 | 1 | 2 | 1 | 2 | 1 | 2 | 1 |
| *suprema* | 2 | 1 | 1 | 1 | 1 | 2 | 1 | 2 | 1 | 2 | 1 |
| *tasskelsoae* | 2 | 1 | 1 | 1 | 1 | 2 | 1 | 2 | 1 | 2 | 1 |
| *tennesseensis* | 2 | 1 | 1 | 1 | 1 | 2 | 1 | 2 | 1 | 2 | 1 |
| *tenua* | 2 | 1 | 1 | 1 | 1 | 2 | 1 | 2 | 1 | 2 | 1 |
| *terricola* | 2 | 1 | 1 | 1 | 1 | 2 | 1 | 2 | 1 | 2 | 1 |
| *tsuga* | 2 | 1 | 1 | 1 | 1 | 2 | 1 | 2 | 1 | 2 | 1 |
