# Supplementary material for: A revision of the minor species group in the millipede genus Nannaria Chamberlin, 1918 (Diplopoda, Polydesmida, Xystodesmidae)
Source: Zookeys. 2021 Apr 13;1030:1–180. doi: 10.3897/zookeys.1030.62544 (PMC8060247; doi:10.3897/zookeys.1030.62544)
Supplement: Supplementary material 6 — Suppl. material 6 [file zookeys-1030-001-s006.docx]

**Supplementary Material 6**

Key to morphological characters used to score *Nannaria*. 1-22 male gonopodal characters, specific to *Nannaria*; 23-27 female cyphopodal characters; 28-47 somatic characters. Adapted from Marek and Bond, 2006.

1. ♂ Acropodites (1) do not cross midline in situ; (2) cross midline in situ.
2. ♂ Prefemoral basal spine (1) absent; or (2) present.
3. ♂ Prefemoral basal spine (1) absent; (2) separate from prefemoral process for some part of length; or (3) entire length fused with acropodite prefemoral process.
4. ♂ Prefemoral basal spine size (1) absent; (2) large, pronounced; or (3) small, reduced.
5. ♂ Prefemoral basal spine shape (1) absent; (2) pointed, sharp; (3) pointed, blunt; (4) rectangular, shelf-like; or (5) reduced to rounded bulge.
6. ♂ Prefemoral process arising from (1) prefemur; (2) top of prefemoral spine; (3) prefemoral spine, dorsomedially; or (4) prefemoral spine, ventromedially.
7. ♂ Prefemoral process shape (1) acicular; (2) laminate; (3) stout; (4) sinuous; or (5) simple, curving.
8. ♂ Prefemoral process curve, when viewed anteriorly (1) straight; (2) medially or (3) laterally.
9. ♂ Prefemoral process crosses acropodite, when viewed anteriorly (1) does not cross; (2) crosses dorsolaterally; or (3) crosses ventrolaterally.
10. ♂ Prefemoral process tip directed (1) ventrally; (2) cephalically; (3) medially; or (4) dorsally.
11. ♂ Telopodite basal zone lateral bulge (1) absent; or (2) present.
12. ♂ Telopodite basal zone width, when viewed anteriorly, relative to space between acropodite and prefemoral process at greatest divide (1) wider; (2) subequal; or (3) thinner.
13. ♂ Telopodite basal zone height, when viewed anteriorly, relative to length of acropodite (1) < half; (2) subequal; or (3) > half.
14. ♂ Acropodite arc (1) straight throughout; (2) straight, with abrupt bend at tip; (3) gradual curve; (4) sinuous; or (5) straight, with bend at midpoint.
15. ♂ Acropodite cingulum (1) absent; or (2) present.
16. ♂ Acropodite swelling (1) swollen before apex; or (2) not swollen before apex.
17. ♂ Acropodite medial flange shape (1) absent; (2) lobed; (3) laminate; or (4) tooth-like.
18. ♂ Acropodite tip medial flange shape (1) absent; (2) triangular; (3) lobed; or (4).
19. ♂ Acropodite tip lateral flange shape (1) absent; (2) triangular; (3) lobed; (4) hooked; or (5) laminate.
20. ♂ Acropodite tip directed, when viewed anteriorly (1) medially; (2) caudally; (3) laterally; (4) cephalically; (5) dorsally; or (6) ventrally.
21. ♂ Acropodite distal zone length (1) absent; (2) short, less than 0.4x length of acropodite; or (3) long, greater than or equal to 0.5x length of acropodite.
22. ♂ Acropodite tip shape (1) sharp; (2) blunt.
23. ♀ Cyphopodal receptacle (1) absent; or (2) present.
24. ♀ Cyphopodal receptacle size at widest part (1) absent; (2) shorter than prefemur length (3) subequal to prefemur length (4) wider than prefemur length.
25. ♀ Cyphopodal valves, symmetry (1) symmetric; or (2) asymmetric.
26. ♀ Cyphopodal valves, orientation (1) ventrally; (2) anteroventrally; (3) posteroventrally; (4) twisted posterior; (5) laterally.
27. ♀ Cyphopodal receptacle cuticle surface (1) absent; (2) smooth; or (3) sculptured.
28. Gnathochilarium lateral emargination (1) absent; or (2) present.
29. Antennomere one distal cuticle conformation (1) cylindrical, not wrapped around cones; or (2) wrapped around cones.
30. Collum ridges (1) absent; or (2) present.
31. Caudolateral corners, paranota I-X shape (1) acute, projecting caudally; (2) rounded cephalically.
32. Caudolateral corners, paranota I-XIX shape (1) acute, projecting caudally on all segments; (2) rounded cephalically on segments I-X only; or (3) rounded cephalically on segments I-XIX.
33. Metatergal pores, metatergites IX+X (1) absent; or (2) present.
34. Metatergal linear bump-pores (1) absent; or (2) present.
35. Lateral wrinkles, Tergites IX+X (1) tightly wrinkled; or (2) loosely wrinkled.
36. Repugnatorial pores, paranota IX+X orientation (1) laterally; or (2) dorsally.
37. Metatergal dorsal slits (1) absent; or (2) present.
38. Metatergal dorsal microsculpture mesh shape (1) isodiametric; or (2) anisodiametric.
39. Paranota-dorsum, segments IX+X angle (1) 130°; or (2) 180°.
40. Paranotal segments IX+X width (1) thin; or (2) thick;
41. ♂ Gonapophyses shape (1) cylindrical; or (2) goblet-like.
42. Sternal knobs, 4^th^ leg pair (1) absent; or (2) present.
43. Pleural process, segments IX+X (1) absent or (2) present;
44. Sternal triangular spines, segments IX+X (1) absent; or (2) present;
45. Sternal median bulge, segments IX+X (1) absent; or (2) present;
46. Setae, sterna IX+X (1) absent; or (2) present;
47. Ventral excavation, sterna IX-X (1) absent; or (2) present.
